# A light-driven multi-state heterojunction transistor for optoelectronic ternary logic circuits

**DOI:** 10.1038/s41467-026-74238-y

**Published:** 2026-06-19

**Authors:** Chungryeol Lee, Dongho Choi, Sanghoon Park, Jeong-ik Park, Taehyun Nam, Minjae Jang, Seunghyup Yoo, Sung Gap Im

**Affiliations:** 1 Department of Chemical and Biomolecular Engineering, Korea Advanced Institute of Science and Technology (KAIST), Daejeon, South Korea; 2 School of Electrical Engineering, Korea Advanced Institute of Science and Technology (KAIST), Daejeon, South Korea; 3 Graduate School of Semiconductor Technology, Korea Advanced Institute of Science and Technology (KAIST), Daejeon, South Korea

**Keywords:** Electronic devices, Electronic devices

## Abstract

A multi-state transistor that converts optical inputs into discrete logic outputs provides a device-level framework for optoelectronic computing beyond the binary paradigm. Here, we present a light-driven ternary heterojunction transistor (T-HTR), featuring three distinct states – off, on, and a light-tunable intermediate state – within a single device. The multi-state switching is achieved by a p-type/n-type heterojunction with a controlled charge injection barrier at the source electrode. This design enables the device to be maintained in the off-state in the absence of intermediate logic operations, thereby minimizing static power consumption. An optoelectronic ternary logic inverter based on the T-HTR is capable of full-swing pull-up and pull-down operations with a light-tunable intermediate state, realizing the functions of standard, positive, and negative ternary inverters within a single device. We further demonstrate a pixel-level sensory circuit as a proof-of-concept for in-sensor computing, enabling multifunctional image processing tasks, such as pixel integration and intersection, through dynamic switching of logic functions.

## Abstract (web summary)

Most optoelectronic circuits remain constrained by conventional binary logic. Here, Lee et al. report a light-driven multi-state transistor that converts optical inputs into ternary logic states, enabling reconfigurable logic and in-sensor image processing within a single device architecture.

## Introduction

Optoelectronic logic circuits have attracted significant attention due to their potential to enhance computational efficiency and to provide a versatile processing architecture. By harnessing optical signals, these circuits facilitate high-speed parallel processing, spatial signal computation, and contactless data transmission, rendering them energy-efficient alternatives to conventional electronic circuits^1,2^. Such advantages establish optoelectronic logic circuits as key enablers in emerging computing schemes, such as reconfigurable logic^3–5^, in-sensor computing^6,7^, and neuromorphic vision systems^8,9^.

A fundamental strategy in optoelectronic logic circuits involves modulating the field-effect characteristics of semiconductors with light, thereby diversifying the switching behavior of individual transistors. Such optical modulation is typically achieved under controlled illumination rather than ambient light, using localized or integrated light sources to ensure stable and selective device operation. This approach facilitates light-based information processing and flexible logic execution within a minimal circuit footprint, thereby alleviating the need for complex circuit architectures or large-scale hardware configurations. Despite their potential, most existing optoelectronic circuits remain confined within conventional digital logic frameworks, where individual transistors operate solely as binary switches, toggling between on and off states^10–13^. Consequently, diverse optical signals—such as intensity or wavelength variations—are reduced to mere binary representations, limiting their role to an on-or-off switch. This structural constraint not only limits data throughput but also suppresses the full potential of optoelectronic logic circuits, preventing them from leveraging the variable characteristics of light to enable more efficient and sophisticated computational operations.

A promising solution is to introduce multi-valued logic into optoelectronic circuits^14^. Multi-valued logic refers to computation using more than two logic states, enabling higher data processing efficiency and improved integration density within the same design rule. For example, ternary logic is known to reduce the system complexity by ~63% compared to conventional binary logic systems^15^. By providing a distinct intermediate logic state, the ternary logic system can establish a framework that more effectively exploits the variable characteristics of light signals beyond conventional binary on/off switches. Here, this intermediate state is not merely an additional logic level but a light-driven, dynamically adjustable state that can transition toward either the on- or off-states or remain stable between them. Thus, this intermediate state serves as a bridge, preserving optical information that would otherwise be lost in binary logic systems. Therefore, optoelectronic ternary logic circuits ensure that the diverse properties of light can be reflected directly in computational operations, rather than being constrained to rigid binary classifications. In particular, a transistor capable of intrinsically supporting multiple logic states can enable circuit functions to be implemented more compactly, as operations that would otherwise require several binary switching elements may be realized within a single multi-state device.

The realization of optoelectronic multi-valued logic circuits requires the development of transistors capable of exhibiting three distinct switching states. Such transistors have been demonstrated using heterojunction transistors with various channel materials, including organic semiconductors^16–18^, graphene^19–21^, metal oxides^22–24^, and two-dimensional (2D) transition metal dichalcogenides (TMDs)^25–27^. A key characteristic of heterojunction transistors is the presence of negative transconductance, where the drain current (${I}_{{{{\rm{D}}}}}$) peaks and subsequently decreases within a specific gate voltage (${V}_{{{{\rm{G}}}}}$) range, thereby constructing an intermediate state^28^. Several light-driven multi-valued logic devices have extended this concept by exploiting optically tunable negative transconductance in heterojunction transistors. For example, InSe-based transistors have exhibited configurable photo-responses enabling ternary logic operation^29^, where trap-assisted processes enabled negative transconductance and optically switchable multi-level states. Similarly, optically reconfigurable ternary logic operation has been demonstrated in C8-BTBT/PhC2-BQQDI heterojunction transistors, exhibiting improved voltage-transfer characteristics and enhanced static-noise margins under UV illumination^30,31^. Additional efforts have focused on photonic modulation of negative transconductance in In_2_O_3_/PDPP3T heterojunction transistors, enabling binary-to-ternary logic conversion under monochromatic optical inputs^32^. While these studies successfully demonstrated that light can serve as an additional control knob for multi-state logic, the negative-transconductance mechanism is inherently ${V}_{{{{\rm{G}}}}}$-dependent, and therefore the heterojunction transistor cannot establish a stable logic state by itself. This requires an additional complementary transistor to construct a complementary inverter, imposing a critical design constraint. Moreover, under illumination, the coexistence of photocurrent and gate-induced field-effect current further aggravates the instability of the intermediate state. This overlap also limits the dynamic range of the photocurrent, reducing its precision in optical-state encoding and leading to significant leakage current in the intermediate regime that may ultimately result in high static power consumption^30–33^. Overcoming these limitations requires a transistor that intrinsically exhibits a ${V}_{{{{\rm{G}}}}}$-independent intermediate state, whose level is finely tunable by light. To date, however, no such device has been experimentally demonstrated.

Here, we present a ternary heterojunction transistor (T-HTR) that enables the formation of a stable intermediate state between the on- and off-states, characterized by a photocurrent, which is independent of ${V}_{{{{\rm{G}}}}}$ (i.e., zero transconductance). The proposed T-HTR features a vertical p-type/n-type heterojunction structure with an injection barrier layer beneath the source electrode. This configuration enables efficient charge separation at the vertically stacked p-type/n-type heterojunction, directing photogenerated carriers to the source and drain, while suppressing charge injection from the source, thereby establishing a constant photocurrent in the intermediate state. By harnessing the T-HTR, we demonstrate an optoelectronic ternary logic inverter (T-inverter) with the intermediate logic dynamically reconfigured between logic 2 and logic 0 along with the input light intensity, which enables the implementation of a standard ternary inverter (STI), positive ternary inverter (PTI), and negative ternary inverter (NTI) modes within a single transistor configuration. Optoelectronic ternary logic gates, such as OR and AND gates, are also implemented, where both input voltage and light serve as control parameters to modulate the logic outputs, consistently matching the expected truth table behavior. Finally, we demonstrate a pixel-level sensory circuit consisting of 2 by 2 T-HTRs as a proof-of-concept for in-sensor computing, which reliably performs multifunctional image processing tasks, such as pixel integration and intersection. We believe that the T-HTR developed in this study provides a device-level foundation for future optoelectronics, bridging optical input encoding and multi-state logic within a single transistor, thereby enabling compact in-sensor computing systems based on multi-state optoelectronic devices.

## Results

### Design of the ternary heterojunction transistor

**Fig. 1:**
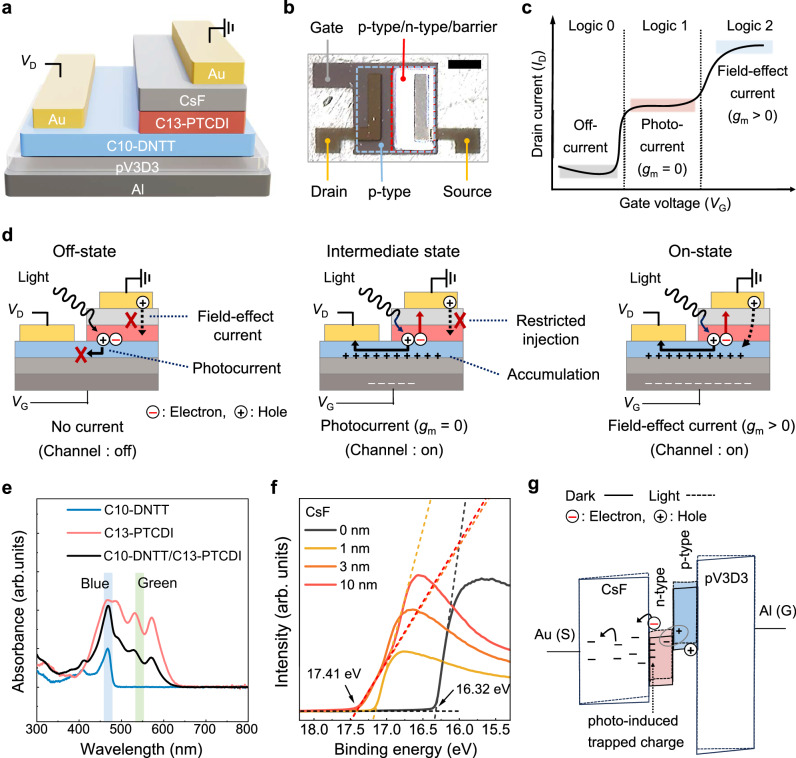
Design of the ternary heterojunction transistor (T-HTR). **a** The schematic illustration and **b** optical microscopy image of the T-HTR (scale bar: 500 µm). **c** The schematic illustration of transfer characteristics and **d** operational mechanism of the T-HTR consisting of three switching states. **e** Ultraviolet-visible (UV/Vis) absorption spectra of the n-type (C13-PTCDI), p-type (C10-DNTT) and n-type/p-type bilayer organic semiconductors. **f** Ultraviolet photoelectron spectroscopy (UPS) spectra of Au contact with thermally deposited cesium fluoride (CsF) as a function of CsF thickness. **g** Energy band diagrams of the source-side stack in the dark (solid lines) and under illumination (dashed lines). The photo-induced trapped charge at the CsF/p-type interface enhances the internal electric field in CsF.

The T-HTR architecture is designed by harnessing a p-n heterojunction structure, wherein the p-type acts as the main semiconductor and the n-type functions as the sub-semiconductor, respectively, to enable complementary electronic functionalities within the device structure (Fig. 1a, b). The main semiconductor refers to the semiconductor that forms an interface with the gate dielectric and extends across the entire channel, whereas the sub-semiconductor is positioned between the main semiconductor and the source electrode, forming a vertical p-type/n-type heterojunction. The continuous main-semiconductor from source to drain serves as a channel for field-effect current, allowing holes injected from the source to flow through the p-type main-semiconductor channel toward the drain, when a sufficient ${V}_{{{{\rm{G}}}}}$ is applied. On the other hand, the p-type/n-type vertical heterojunction promotes exciton dissociation under light illumination, facilitating the transport of photogenerated electrons through the n-type sub-semiconductor to the source, while photogenerated holes through the p-type main-semiconductor to the drain. An injection barrier layer is deliberately introduced between the sub-semiconductor and the source contact. The injection barrier layer induces asymmetric charge transport by blocking hole injection from the source while facilitating electron transport, according to the energy-level alignment. Therefore, when hole injection from the source is suppressed at low ${V}_{{{{\rm{G}}}}}$, the photogenerated carriers predominantly contribute to the photocurrent, thereby inducing a stable photocurrent. As a result, the proposed light-driven T-HTRs achieve three distinct output states within a single architecture—the off-state, intermediate state, and on-state—as schematically illustrated in Fig. 1c and Fig. 1d. In the off-state, the absence of a conductive channel inhibits charge transport, resulting in negligible current flow. In the intermediate state, the injection barrier plays a pivotal role by selectively allowing photocurrent while suppressing field-effect current, thereby establishing a ${V}_{{{{\rm{G}}}}}$-independent, stable intermediate state, characterized by a constant photocurrent with zero transconductance (ZTC, ${g}_{{{{\rm{m}}}}}$ = 0). Finally, in the on-state, a sufficiently high ${V}_{{{{\rm{G}}}}}$ facilitates hole injection at the source, leading to a high field-effect current (${g}_{{{{\rm{m}}}}}$ > 0).

In this study, 2,9-Didecyldinaphtho[2,3-b:2’,3’-f]thieno[3,2-b]thiophene (C10-DNTT, 20 nm) and N,N’-Ditridecyl-3,4,9,10-perylenetetracarboxylic diimide (C13-PTCDI, 20 nm) were utilized as the p-type and n-type channel materials, respectively, both of which are well-established high-mobility organic semiconductors, exhibiting hole and electron mobilities exceeding 1 cm^2 ^V^−1^ s^−1^ (Supplementary Fig. 1). They are also compatible with sequential vacuum evaporation, thereby facilitating the construction of p-type/n-type heterojunction structure^34^. A thickness of 20 nm for both semiconductors was selected, as it closely matches the reported exciton diffusion length in these small-molecule systems^35^ and ensures a continuous crystalline morphology for efficient charge transport^36–38^, while avoiding contact-resistance penalties associated with thicker films^39^. Ultraviolet-visible (UV-vis) spectroscopy reveals that C10-DNTT exhibits an absorption peak around 460 nm (blue light), whereas C13-PTCDI shows absorption peaks not only near 460 nm but also around 535 nm (green light) (Fig. 1e). When the two materials are stacked to form a C13-PTCDI/C10-DNTT bilayer heterojunction, the absorption spectrum becomes broader and complementary, covering both regions while exhibiting a slightly lower overall absorbance than the simple sum of the individual layers due to Fresnel reflections and interference at the interfaces^40^. A poly(1,3,5-trimethyl-1,3,5-trivinyl cyclotrisiloxane) (pV3D3, 50 nm) layer deposited in vapor-phase via initiated chemical vapor deposition (iCVD) was utilized as the gate dielectric layer^41^. The dielectric constant of the pV3D3 film was determined to be approximately 2.2, from which the dielectric thickness in the T-HTR was determined to be 50 nm (Supplementary Fig. 2). The chemical structures of the organic semiconductors and the polymer dielectric used in this study are presented in Supplementary Fig. 3. Au was used as the source and drain contacts. Cesium fluoride (CsF), often used as an electron transport layer in photovoltaic applications due to its strong interfacial dipole moment, was introduced as the injection barrier layer^42–44^.

To investigate the energy level alignment at the source electrode of the T-HTR, ultraviolet photoelectron spectroscopy (UPS) was conducted on Au with thermally deposited CsF as a function of CsF thickness (Fig. 1f). The apparent work function of the Au surface decreases from 4.9 to 3.8 eV as the CsF thickness increases from 0 to 10 nm. Notably, the most pronounced shift occurs within the first nanometer, corresponding to a drop from 4.9 eV to approximately 4.1 eV, followed by a gradual saturation at larger thicknesses. This reduction reflects the formation of strong interfacial dipoles at the buried Au/CsF interface that modify the electrostatic potential across the metal/dipole stack and effectively lower the vacuum level at the outer CsF surface. Such dipole-induced shifts have been widely reported for alkali halide interlayers and arise from partial charge transfer and polarization at the metal/insulator interface. The subsequent saturation behavior is consistent with the cancellation of densely packed dipoles and the attainment of an electrostatic equilibrium at the surface^45,46^. In this study, a 60 nm-thick CsF layer was employed to enhance the injection barrier functionality beyond dipole formation. The CsF exhibits a large bandgap of approximately 5.29 eV along with a valence band maximum of around 7.1 eV (Supplementary Fig. 4).

To elucidate the charge-transport mechanism through this thick CsF layer, we fabricated vertical Au/C10-DNTT (20 nm)/C13-PTCDI (20 nm)/CsF (0–90 nm)/Au stacks that replicate the source-side configuration of the T-HTR. The current density (${J}_{{{{\rm{i}}}}}$)–electric field (${E}_{{{{\rm{i}}}}}$) characteristics of this stacked device show a systematic decrease in ${J}_{{{{\rm{i}}}}}$ with increasing CsF thickness, confirming that a thicker barrier effectively suppresses carrier transmission (Supplementary Fig. 5). Notably, when the experimental data were analyzed in Poole-Frenkel-type and Schottky-type representations, the Poole-Frenkel representation shows better linearity across the investigated CsF thickness range. These results suggest that charge transport through the thick, wide-bandgap CsF layer is better described as field-assisted trap-mediated transport, rather than injection-limited emission^47,48^. In this mechanism, a thicker CsF layer reduces the average electric field and extends the trap-mediated path, thereby lowering the overall transmission probability.

To further quantify the semiconductor energy levels used for band alignment, UPS analysis was also performed on the organic semiconductors. The ionization energies extracted from the UPS spectra were 4.92 eV for C10-DNTT and 6.42 eV for C13-PTCDI (Supplementary Fig. 6). Subsequently, the optical band gaps were obtained from UV-vis absorption measurements using Tauc analysis (Supplementary Fig. 7), yielding values of 2.59 and 2.06 eV for C10-DNTT and C13-PTCDI, respectively. By combining the UPS-derived ionization energies with the optical band gaps, the electron affinities were determined to be 2.33 eV for C10-DNTT and 4.36 eV for C13-PTCDI.

The energy band diagram of the T-HTR from gate to source illustrates how the thick CsF layer governs the source-contact behavior in the light-induced intermediate state (Fig. 1g). Under illumination, exciton dissociation at the p-n heterojunction generates minority electrons that drift toward the CsF side. In this configuration, both hole injection from the source and light-induced minority-electron transport from the n-type layer are, in principle, possible via the trap-assisted process; however, the band alignment strongly favors electron transport while suppressing hole injection. Specifically, the apparent work function of the Au/CsF contact ( ~ 3.8 eV) is shallower than the lowest unoccupied molecular orbital (LUMO) level of C13-PTCDI (4.36 eV), whereas the much deeper highest occupied molecular orbital (HOMO) level of the C13-PTCDI (6.42 eV) imposes a large barrier. Notably, photogenerated holes can still be transported through the p-type channel toward the drain, contributing to photocurrent, even though gate-induced hole injection from the source remains energetically suppressed. Consequently, field-effect current remains suppressed, whereas photocurrent can readily flow through the trap-assisted pathway, giving rise to the stable intermediate conduction state. Meanwhile, a fraction of minority electrons that drift toward the CsF side is likely captured by interfacial states at the C13-PTCDI/CsF junction. This establishes a photo-induced dipole and alters the potential distribution across the series dielectric stack at a fixed ${V}_{{{{\rm{G}}}}}$ via Gauss’s law, increasing the electric field in the CsF layer (steeper band slope) while partially screening the upstream pV3D3 layer (flatter band slope). This enhanced electric field in CsF lowers the effective barrier for trap-assisted conduction, leading to a large threshold voltage (${V}_{{{{\rm{TH}}}}}$) shift in devices incorporating a CsF injection barrier under illumination, as will be discussed later.

The band configurations for the off- and on-states are also provided in Supplementary Fig. 8, together with the intermediate-state band diagram. In the off state, the channel is fully depleted; even under illumination, photogenerated carriers cannot contribute meaningfully to conduction because they cannot percolate through the depleted channel. In the on state, under strong gate bias, the electric field effectively reduces the hole-injection barrier through CsF and enables efficient hole transport into the p-type channel, with photocurrent further augmenting the total conduction. To clearly illustrate the role of the CsF/n-type/p-type stack at the source side of the T-HTR, band diagrams for devices with reversed source/drain configuration are also provided in Supplementary Fig. 8 for direct comparison.

### Electrical properties of the transistor

**Fig. 2:**
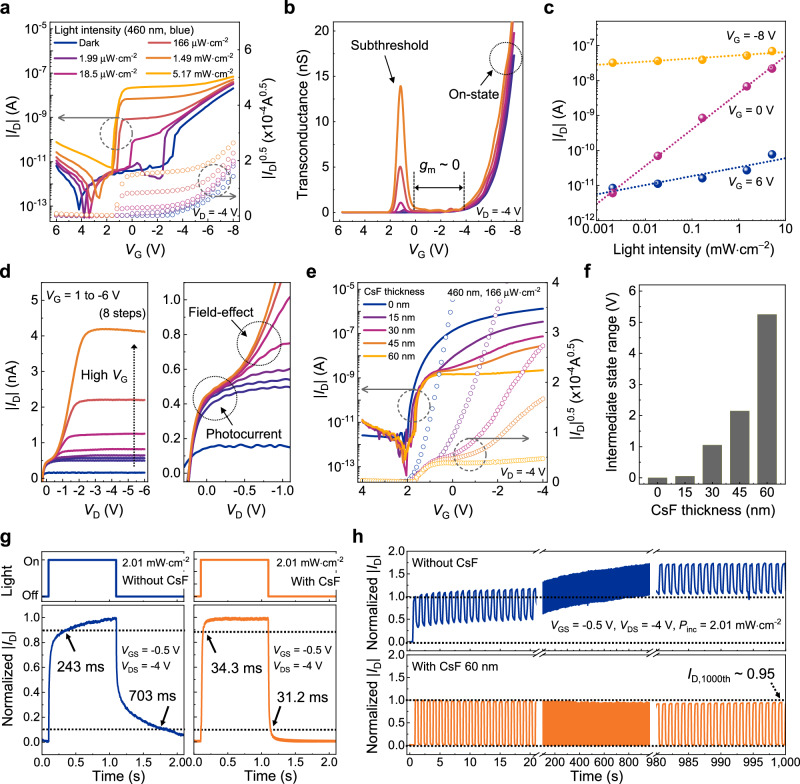
Electrical characterization of the T-HTR. **a** The transfer characteristics, **b** transconductance (${g}_{{{{\rm{m}}}}}$), and **c** extracted drain current (${I}_{D}$) at the off-, intermediate-, and on-states of the T-HTR under 460 nm illumination with respect to light intensity. The drain voltage (${V}_{{{{\rm{D}}}}}$) is fixed at −4 V. **d** Output characteristics of the T-HTR (left), with an expanded view of the low ${V}_{{{{\rm{D}}}}}$ region (right) under 166 μW∙cm^-2^ of 460 nm illumination. **e** The transfer characteristics of the T-HTR and **f** extracted range of the intermediate state with respect to CsF thickness. **g** Time-resolved photocurrent response under pulsed 460 nm illumination in the intermediate state of T-HTR without (left) and with (right) the CsF injection barrier. **h** Normalized ${I}_{D}$ under 1000 repeated 460 nm light pulses (0.5 s duration per cycle) in the intermediate state of the T-HTR without (top) and with (bottom) the CsF injection barrier.

The transfer characteristics of the T-HTR were investigated (Fig. 2a). Under dark conditions, the T-HTR exhibits typical on and off switching behavior. On the other hand, under 460 nm illumination, a constant photocurrent is observed between the on and off states, defining the intermediate state. As the light intensity increases, the ${V}_{{{{\rm{TH}}}}}$ decreases gradually, which is attributed to the internal photovoltage generated by interfacial dipoles and the accumulation of photogenerated minority carriers beneath the injection-barrier layer^49–52^. These trapped charges locally bend the energy bands, effectively lowering the gate bias required for channel formation (Supplementary Fig. 9). Notably, this ${V}_{{{{\rm{TH}}}}}$ reduction eventually saturates to the value observed in the control device without the CsF barrier, indicating that the presence of the injection barrier elevates the initial ${V}_{{{{\rm{TH}}}}}$ of the T-HTR by suppressing hole injection (Supplementary Fig. 10). The transconductance profile exhibits a well-defined ZTC region situated between the subthreshold region and the on-state (Fig. 2b). The sharp transconductance peak in the subthreshold region corresponds to the transition from channel depletion to channel accumulation, while the subsequent near-zero flattening of the transconductance in the ZTC region indicates the suppression of field-effect current. It should be noted that this behavior does not correspond to a negative transconductance regime. While negative transconductance involves a decrease in ${I}_{{{{\rm{D}}}}}$ with increasing ${V}_{{{{\rm{G}}}}}$ (${g}_{{{{\rm{m}}}}}$ < 0), the T-HTR maintains positive ${g}_{{{{\rm{m}}}}}$ (${g}_{{{{\rm{m}}}}}$ > 0) that simply approaches zero, reflecting the suppression of field-effect current and the formation of a ${V}_{{{{\rm{G}}}}}$-independent intermediate state. Also, double-sweep measurements of transfer and transconductance curves exhibit negligible hysteresis, confirming that the near-zero-${g}_{{{{\rm{m}}}}}$ region arises from the engineered injection barrier rather than trap-induced instability (Supplementary Fig. 11). As the light intensity increases from dark to 5.17 mW cm^-2^, the photocurrent in the intermediate state (at ${V}_{{{{\rm{G}}}}}$ = 0 V) increases from 3.8 pA to 21.9 nA. Note that the dynamic photocurrent range in the intermediate state is close to the on/off current ratio of the T-HTR, indicating that light exposure can induce on/off switching of the transistor, enabling reconfigurable optoelectronic ternary logic circuits. This also enables the device to be maintained in the off-state in the absence of intermediate logic operations, thereby minimizing static power consumption to as low as ~3.2 pW cm^-2^ in the intermediate state, which is significantly lower than that of conventional ternary logic transistors (Supplementary Table 1). Such low static power consumption at the intermediate state was reproducibly obtained across 12 devices, with the dark-state ${V}_{{{{\rm{TH}}}}}$ tightly distributed around -2.26 ± 0.21 V (Supplementary Fig. 12). When the photocurrent is plotted as a function of light intensity, it exhibits a linear relationship in the intermediate state (Fig. 2c). Such a good linearity is attributed to the injection barrier layer, which suppresses efficiently the light-induced additional carrier injection from the source, thereby ensuring a linear proportion of photocurrent to the amount of the photogenerated carriers^53–56^. To examine the wavelength-dependent operation of the device, the T-HTR was illuminated with monochromatic light at 460, 535, and 627 nm. Distinct photocurrent responses are observed, consistent with the absorption characteristics of the C13-PTCDI/C10-DNTT heterojunction (Supplementary Fig. 13). This spectral dependence demonstrates the optical tunability of the T-HTR and suggests the feasibility of wavelength-selective logic operations. The dynamic photocurrent range in the off- and on-states is far lower ( < 10^1^) compared to that of the intermediate state ( ~ 10^4^). This is because the photocurrent is ultimately limited by channel depletion of the main semiconductor in the off-state, whereas field-effect current dominates in the on-state. The output characteristics of the T-HTR under 166 μW cm^-2^ of 460 nm light exhibit a well-defined saturation region for the intermediate state (${V}_{{{{\rm{G}}}}}$ = 1 to −2 V) and on-state (${V}_{{{{\rm{G}}}}}$ = −3 to −6 V), demonstrating stable current output at high drain voltage ( | ${V}_{{{{\rm{D}}}}}$ | > 3 V) (Fig. 2d). In the intermediate state, the T-HTR exhibits a single photocurrent-driven regime across the entire ${V}_{{{{\rm{D}}}}}$ range. By contrast, in the on-state, two distinct current regimes emerge at low ${V}_{{{{\rm{D}}}}}$: photocurrent dominates for |${V}_{{{{\rm{D}}}}}$ | < 0.5 V, while field-effect current prevails for |${V}_{{{{\rm{D}}}}}$ | > 0.5 V. This operational transition produces S-shaped curves with a distinct inflection point, reflecting the contact resistance imposed by the injection barrier layer. On the other hand, the output characteristics of the T-HTR under dark conditions support the analysis above, showing a single current regime with S-shaped curves throughout the entire ${V}_{{{{\rm{G}}}}}$ range (Supplementary Fig. 14).

To validate the operating mechanism and the role of each layer in the T-HTR, transfer characteristics were examined under three modified device configurations: (1) eliminating the n-type/p-type vertical heterojunction by removing the sub-semiconductor; (2) allowing for facile charge injection from source by reducing the injection barrier; and (3) disrupting the transport of photogenerated charge carriers by reversal of the source and drain (electrons toward the p-type and holes toward the n-type) (Supplementary Fig. 10). None of these configurations yields a stable intermediate state, indicating that the following conditions are essential for establishing a distinct intermediate state between the on and off-states: (1) efficient exciton dissociation through the n-type/p-type vertical heterojunction, (2) suppression of field-effect current by the injection barrier when a low ${V}_{{{{\rm{G}}}}}$ is applied, and (3) proper source-drain orientation to facilitate the transport of the photogenerated charge carriers (electrons toward the n-type and holes toward the p-type).

The influence of the injection barrier thickness on the intermediate state was investigated (Fig. 2e). As the CsF thickness increases from 15 to 60 nm, the transition into the intermediate state becomes more distinct. The range of the intermediate state—defined as the ${V}_{{{{\rm{G}}}}}$ window between the initial ${V}_{{{{\rm{G}}}}}$, where transconductance starts to decrease from its peak, and the end ${V}_{{{{\rm{G}}}}}$, where transconductance increases back to a level comparable to the initial peak value—broadens from less than 1 V for 15 nm-thick CsF to more than 4 V for 60 nm-thick CsF (Fig. 2f). Such expansion results from the progressively restricted effective charge injection from the source with increasing barrier thickness, which shifts the onset voltage of field-effect current to higher ${V}_{{{{\rm{G}}}}}$.

To further elucidate how the injection barrier thickness affects the device figure of merit, we quantitatively analyzed its influence on the operating speed and power consumption. As the CsF thickness increases, the on-current gradually decreases due to the restricted charge injection from the source. The reduction in on-current lowers the transconductance and consequently the unity-gain cut-off frequency^57^:

1 $${f}_{{{{\rm{T}}}}}={g}_{{{{\rm{m}}}}}/{2\pi C}_{{{{\rm{gg}}}}}$$

where ${g}_{{{{\rm{m}}}}}$ is the transconductance and ${C}_{{{{\rm{gg}}}}}$ is the total gate capacitance. Meanwhile, the power consumption also decreases with decreasing on-current, resulting in a clear trade-off between operating speed and power efficiency (Supplementary Fig. 15). As the CsF thickness increases from 15 to 60 nm, the estimated cut-off frequency decreases from 10^2^–10^3 ^Hz to below 1 Hz, whereas the on-state (dynamic) power density drops from the mW cm^-2^ scale to the sub-μW cm^-2^ scale. The relatively low cut-off frequency estimated in the present devices is mainly attributed to the shadow-mask-defined geometry commonly used for proof-of-concept device demonstrations, which results in large device dimensions and substantial gate-overlap induced parasitic capacitance.

The influence of the injection barrier on response speed was also investigated by applying a 460 nm light pulse (2.01 mW cm^-2^) for 1 s and measuring the photocurrent in the intermediate state (Fig. 2g), which captures the transient response of photogenerated carriers under a constant electrical bias. Accordingly, this transient optical response is distinct from the cut-off frequency discussed above, which reflects the electrical switching limit under gate modulation. Without the CsF injection barrier, the T-HTR exhibits a slow rising time (time to reach from 10 to 90% of the saturation photocurrent) of 243 ms and a prolonged falling time (time to decay from 90 to 10% of the saturation current) of 703 ms upon light switching. Such sluggish response behavior is consistent with a high density of interfacial trap sites at the evaporated Au contact, where energetic metal deposition is known to generate localized electronic states that capture and slowly release photocarriers, thereby limiting the transient dynamics^58–60^. In contrast, with the 60 nm-thick CsF injection barrier, the rising time is reduced to 34.3 ms, and the falling time is shortened to 31.2 ms, resulting in a much sharper transient response with well-defined on/off switching behavior. This improvement suggests that the CsF layer effectively suppresses the dominant trap-mediated relaxation pathways at the metal/semiconductor interface. We note, however, that the reversible ${V}_{{{{\rm{TH}}}}}$ modulation observed under illumination indicates the possible presence of a small number of rapidly responding interfacial states or dipole-forming sites at the CsF/semiconductor boundary. These states may transiently accommodate photocarriers without inducing long-lived storage, consistent with the negligible hysteresis and rapid signal recovery observed.

Given that the incorporation of CsF renders the device contact-resistance dominated, the possible influence of channel transport on the transient response was further examined by varying the channel length of the T-HTR from 100 to 400 µm (Supplementary Fig. 16). The rising and falling times exhibit negligible dependence on channel length, indicating that the temporal response is not channel-limited but governed by a contact-dominated RC delay introduced by the source-side injection barrier. This behavior can be described by a simple RC model^61^:

2 $$\tau \sim ({R}_{{{{\rm{C}}}}}+{R}_{{{{\rm{ch}}}}})\times {C}_{{{{\rm{eff}}}}}$$

where ${R}_{{{{\rm{C}}}}}$ is the contact resistance, ${R}_{{{{\rm{ch}}}}}$ is the channel resistance, and ${C}_{{{{\rm{eff}}}}}$ is the effective capacitance. Because the barrier yields much higher ${R}_{{{{\rm{C}}}}}$ than ${R}_{{{{\rm{ch}}}}}$, the transient time becomes effectively independent of channel length.

To further evaluate the impact of the injection barrier on operational stability, T-HTRs with and without CsF were subjected to 1000 cycles of 460 nm light pulses (2.0 mW cm^-2^), each with a 1 s period and equal 0.5 s on and off times per cycle (Fig. 2h). Without the CsF injection barrier, the T-HTR exhibits a gradual increase in both baseline current and the photocurrent, with the baseline current reaching 1.73 times its initial value, suggesting cumulative charge trapping at the semiconductor/source interface. Conversely, the T-HTR with CsF maintains a stable photocurrent amplitude throughout all cycles, with only a 5% decrease from the initial value, demonstrating improved operational stability.

### Design versatility of the transistor

**Fig. 3:**
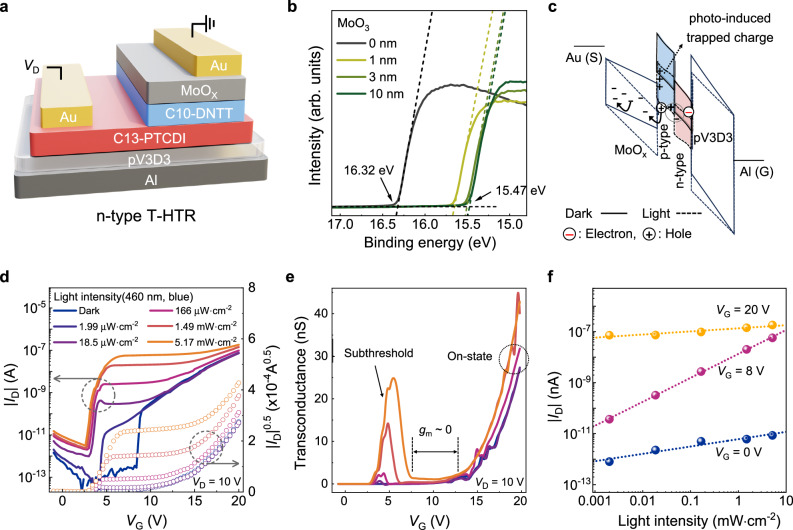
Versatility of the T-HTR architecture. **a** A schematic illustration of the T-HTR with a molybdenum oxide (MoO_x_) injection barrier. **b** Ultraviolet photoelectron spectroscopy (UPS) spectra of the Au contact with thermally deposited MoO_x_ as a function of MoO_x_ thickness. **c** Schematic energy band diagrams of the source-side stack in the dark (solid lines) and under illumination (dashed lines). The photo-induced trapped charge density at the MoO_x_/n-type interface enhances the internal electric field in MoO_x_. **d** The transfer characteristics, **e** transconductance (${g}_{m}$), and **f** extracted drain current (${I}_{D}$) at the off-, intermediate-, and on-states of the T-HTR under 460 nm illumination with respect to light intensity.

While CsF has been demonstrated as an effective injection barrier in the T-HTR, as shown in Fig. 1a, the device architecture allows for alternative material choices that can similarly modulate charge injection and transport characteristics in the switched type of main and sub-semiconductors. In particular, molybdenum oxide (MoO_x_), a well-established hole injection layer for photovoltaic applications due to its deep Fermi level, can be employed in place of CsF to increase the work function of the adjacent electrode, promoting hole transport while preventing electron transport as well^62–64^. With the MoO_x_ injection barrier layer, the T-HTR is designed with an n-type main semiconductor and a p-type sub-semiconductor (Fig. 3a). In this configuration, the MoO_x_ injection barrier beneath the source inhibits electron transport from the source to the n-type semiconductor while permitting hole transport from the p-type semiconductor to the source. Notably, the MoO_x_-based T-HTR functions as an n-type transistor due to the reversed polarity of the semiconductor configuration (n-type for the main semiconductor and p-type for the sub-semiconductor). This highlights that the T-HTR can be configured as either a p-type or an n-type transistor by modifying the semiconductor arrangement and selecting an appropriate injection barrier material, thereby broadening its potential applications.

To examine the energy level alignment at the source electrode of the T-HTR, UPS was conducted on Au with thermally deposited MoO_x_ as a function of MoO_x_ thickness (Fig. 3b). As the MoO_x_ thickness increases from 0 to 10 nm, the apparent work function of the Au progressively increases from 4.9 to 5.75 eV, with the steepest rise occurring within the first nanometer, reflecting the formation of strong interfacial dipoles at the Au/MoO_x_ interface. The band diagram of the T-HTR from gate to source reveals how the MoO_x_ layer governs the source-contact behavior in the light-induced intermediate state, which is analogous to the CsF-based T-HTR (Fig. 3c). Exciton dissociation at the p–n heterojunction generates minority holes that drift toward the MoOₓ interface under illumination. Although both photogenerated holes from the p-type semiconductor and electrons from the source are in principle available, the energy alignment strongly favors hole transport while suppressing electron injection: the apparent work function of Au/MoO_x_ (~5.75 eV) aligns closely with the HOMO level of C10-DNTT (4.92 eV), whereas the LUMO level of C10-DNTT (2.33 eV) imposes a large barrier to electron injection. In addition, similar to CsF, charge transport through the MoO_x_ layer proceeds via trap-assisted conduction due to mixed-valence states and oxygen-deficiency defects^65,66^. As a result, gate-induced field-effect current remains suppressed, while photocarriers establish the intermediate state: holes are extracted through the MoO_x_/source contact, and electrons are transported along the n-type channel toward the drain. The band configurations for the off- and on-states are also provided in Supplementary Fig. 17, together with the intermediate-state band diagram. Also, to clearly illustrate the role of the MoO_x_/p-type/n-type stack at the source side of the T-HTR, band diagrams for devices with reversed source/drain configuration are also provided in Supplementary Fig. 17 for direct comparison.

Building upon the verified T-HTR framework using CsF, we evaluated the electrical behavior of the MoO_x_-based T-HTR as well, assessing the generality of the device design. Under 460 nm light illumination, the MoO_x_-based T-HTR similarly exhibits a ${V}_{{{{\rm{G}}}}}$-independent intermediate state accompanied by a well-defined ZTC region, reproducing the photocurrent-driven operation observed in the CsF-based counterpart (Fig. 3d and Fig. 3e). A linear relationship between light intensity and photocurrent was again confirmed in the intermediate state, with a dynamic range exceeding 10^5^ (Fig. 3f). The MoO_x_-based T-HTR also exhibits output characteristics consistent with the CsF-based design, featuring distinct charge transport regimes in both the intermediate and on-state regions (Supplementary Fig. 18). These results indicate the structural versatility of the T-HTR design: the light-tunable intermediate state is not specific to the CsF-based device, but is a general feature of the proposed architecture enabled by appropriate injection barrier engineering and energy level alignment. Furthermore, although the devices investigated here employ essentially intrinsic organic semiconductors, molecular doping in organic systems–when utilized–can shift the Fermi level via charge-transfer interactions, thereby modulating the built-in potential and the exciton-dissociation driving force at the heterojunction. Importantly, such doping-induced Fermi-level tuning does not substitute the role of the injection barrier but rather provides an additional degree of freedom for tailoring the interfacial energetics in conjunction with barrier engineering, further reinforcing the versatility of the T-HTR architecture.

### Optoelectronic ternary logic gates

**Fig. 4:**
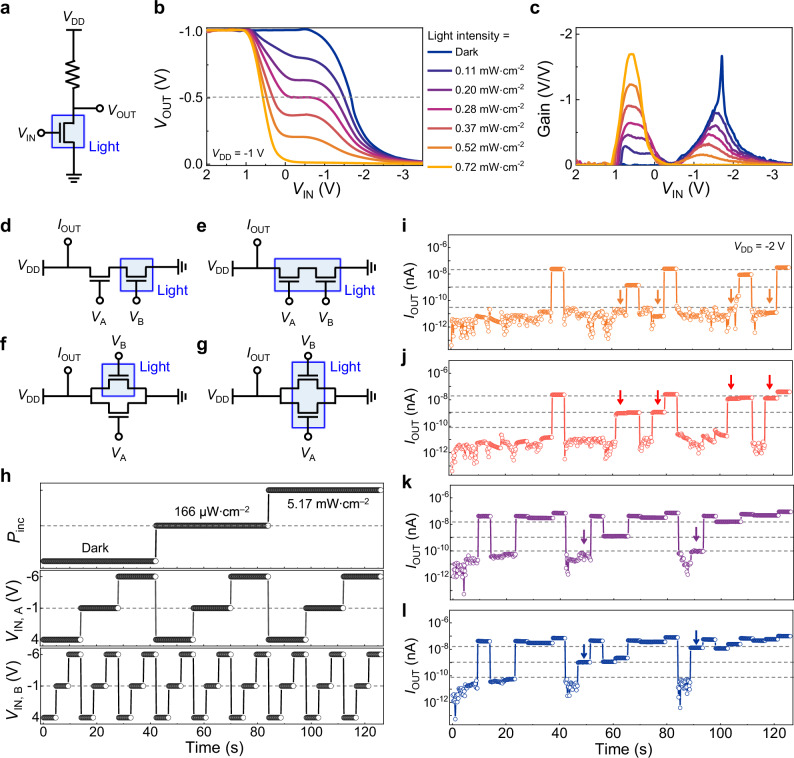
Optoelectronic ternary logic gates. **a** A schematic symbol of a ternary logic inverter (T-inverter). **b** Voltage transfer characteristics and **c** voltage gain profiles of the T-inverter under 460 nm illumination with respect to light intensity. **d**–**g** Schematic symbols of ternary logic gates constructed from two ternary heterojunction transistors, including series-connected AND gates and parallel-connected OR gates with light applied to either one or both transistors. **h** Time-dependent incident light intensity (${P}_{{{{\rm{inc}}}}}$, 460 nm) and input voltages (${V}_{{{{\rm{A}}}}}$, ${V}_{{{{\rm{B}}}}}$) applied to the ternary logic gates for pulsed measurements. **i**–**l** Output current (${I}_{{{{\rm{OUT}}}}}$) responses of the ternary logic gates corresponding to the configurations shown in (**d–g**), respectively. The arrows highlight the changes in output responses when light is applied to one or both of the two T-HTRs.

Optoelectronic logic gates, which process both optical and electrical inputs, are key building blocks for next-generation logic circuits, driving advancements in high-speed, energy-efficient computing architectures. The T-HTR, with its inherent ability to generate three distinct states in response to optical stimuli, offers a promising platform for implementing optoelectronic ternary logic gates. By simply integrating a load resistor, a ternary logic inverter (T-inverter) was realized using the CsF-based T-HTR with a 60 nm-thick CsF layer (Fig. 4a). The T-inverter can process both voltage and light as input signals, where voltage determines the base logic state, while light modulates the output value. The voltage transfer characteristics of the T-inverter illustrate the logic outputs as functions of both input voltage (${V}_{{{{\rm{IN}}}}}$) and light intensity (Fig. 4b). The off-state, intermediate state, and on-state of the T-HTR correspond to input 0, input 1, and input 2 of the T-inverter, respectively. Owing to the stable current levels in the intermediate state of the T-HTR, the T-inverter reliably maintains logic state 1. Since the dynamic range of the intermediate state corresponds to the on/off current ratio of the T-HTR, the output logic value (${V}_{{{{\rm{OUT}}}}}$) for input 1 can be modulated to represent logic levels ranging from 0 to 2, depending on the light intensity applied to the T-inverter. For instance, the T-inverter generates outputs of logic 2, logic 1, and logic 0 for input 1 under light intensities of dark, 0.28 mW cm^-2^, and 0.72 mW cm^-2^, respectively. In contrast, the outputs for inputs 0 and 2 in the ternary inverter remain fixed at logic 2 and logic 0, respectively, as light-driven modulation of the current levels in the off- and on-states of the T-HTR is far less distinct than that in the intermediate state (see Fig. 2c). Consequently, the T-inverter achieves both pull-up (logic 2) and pull-down (logic 0) operations while dynamically adjusting the intermediate logic output between these two states, enabling the functionalities of a standard ternary inverter (STI), positive ternary inverter (PTI), and negative ternary inverter (NTI) within a single inverter configuration (Supplementary Fig. 19). The DC voltage gain profile of the T-inverter shows two distinct peaks, corresponding to the transition between logic states (Fig. 4c). The first gain peak, positioned around ${V}_{{{{\rm{IN}}}}}$ ≈ 0.5 V, represents the transition from logic 2 to logic 1, and the second gain peak, appearing near ${V}_{{{{\rm{IN}}}}}$ ≈ -1.5 V, corresponds to the transition from logic 1 to logic 0. As the light intensity increases, the second gain peak gradually decreases from 1.7 to 0, while the first gain peak conversely increases from 0 to 1.7. This behavior stems from the light-dependent shift of logic 1. Specifically, at a higher light intensity, the output logic state of the intermediate state shifts toward logic 2 from logic 0, resulting in a larger ${V}_{{{{\rm{OUT}}}}}$ change during the transition from logic 2 to logic 1, while reducing the ${V}_{{{{\rm{OUT}}}}}$ change in the transition from logic 1 to logic 0. The electrical stability of the T-inverter was evaluated over 400 consecutive operations (Supplementary Fig. 20). Throughout all cycles, the T-inverter preserves its initial voltage transfer characteristics, maintaining ${V}_{{{{\rm{OUT}}}}}$ for each logic state without degradation. It is worth noting that the T-inverter can also be realized using the MoO_x_-based T-HTR by integrating a load resistor with an appropriate resistance (Supplementary Fig. 21), exhibiting operational characteristics analogous to its CsF-based counterpart.

The operational scheme of the T-inverter, which maintains the fundamental logic framework through inputs 0 and 2 while allowing flexible output transitions between logic 0, 1, and 2 for input 1, enables the development of ternary logic gates for reconfigurable Boolean logic operations. For example, various ternary logic gates were implemented using two CsF-based T-HTRs as fundamental building blocks (Fig. 4d–g). When the T-HTRs are connected in parallel, the output current (${I}_{{{{\rm{OUT}}}}}$) is represented as the sum of the currents through each parallel path, representing an OR logic gate. Conversely, when the T-HTRs are connected in series, the ${I}_{{{{\rm{OUT}}}}}$ is determined by the T-HTR with the highest resistance, allowing it to perform an AND operation. Here, the operational scheme can be further expanded based on where the light is applied. As a result, various operational configurations can be achieved: a series connection for an AND gate, with light applied to one of the two T-HTRs (Fig. 4d), and to both T-HTRs (Fig. 4e); a parallel connection for an OR gate, with light applied to one of the two T-HTRs (Fig. 4f), and to both T-HTRs (Fig. 4g). For input signals, voltage pulses (${V}_{{{{\rm{A}}}}}$ and ${V}_{{{{\rm{B}}}}}$) and a light pulse of varying intensity (${P}_{{{{\rm{inc}}}}}$) were applied to T-HTRs, with each input pulse having three distinct levels. For instance, the T-HTR can exhibit three states based on the applied voltage pulses: the off-state (${V}_{{{{\rm{G}}}}}$ = -6 V), the intermediate state (${V}_{{{{\rm{G}}}}}$ = -1 V), and the on-state (${V}_{{{{\rm{G}}}}}$ = 4 V) (Fig. 4h). Similarly, depending on the light intensity, the T-HTR can transition between off-state (${P}_{{{{\rm{inc}}}}}$ = 0 µW cm^-2^), intermediate state (${P}_{{{{\rm{inc}}}}}$ = 152 µW cm^-2^), and on-state (${P}_{{{{\rm{inc}}}}}$ = 2.01 mW cm^-2^). For both parallel and series T-HTR connections, the ternary logic gates exhibit consistent ${I}_{{{{\rm{OUT}}}}}$ in response to input signals ${V}_{{{{\rm{A}}}}}$, ${V}_{{{{\rm{B}}}}}$, and ${P}_{{{{\rm{inc}}}}}$ (Fig. 4i–l). By defining threshold current values of 5 × 10^-10 ^A as the lower bound and 1 × 10^-8 ^A as the upper bound for logic 1, the ${I}_{{{{\rm{OUT}}}}}$ is clearly distinguished into three discrete logic states. When classified as logic 0, logic 1, and logic 2 using these current thresholds, the output logic sets of the implemented ternary logic gates are fully consistent with their expected truth table (Supplementary Table 2). Notably, when light is applied to only one of the T-HTRs, the output pattern of the ternary logic gates changes (marked by an arrow), even under the same voltage pulses and light intensity. This dynamic response to selective (or patterned) light illumination highlights the versatility in the logic encoding of optoelectronic ternary circuits. Although the present demonstration adopts a current-based logic configuration, a voltage-based logic gate can also be implemented using T-HTRs by pairing p-type and n-type devices in a complementary manner, as conceptually demonstrated in Supplementary Fig. 22.

### Application to in-sensor computing

**Fig. 5:**
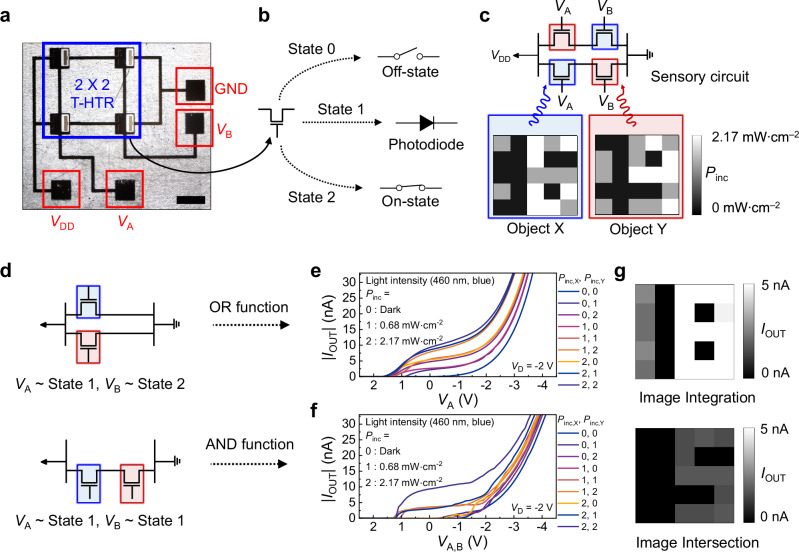
Pixel-level sensory circuit for in-sensor computing. **a** An optical microscopy image of the sensory circuit (scale bar: 1 µm). **b** A schematic symbol of the three distinct operational states of the T-HTR. **c** Conceptual representation of image processing using the sensory circuit and two 5×5 pixel matrices representing objects X and Y, each with predefined light intensity levels. Two T-HTRs receive optical signals from object X (blue shading), while the other two receive signals from object Y (red shading). **d** Dynamic reconfiguration of the sensory circuit for OR and AND logic operations depending on the applied voltage signals (${V}_{{{{\rm{A}}}}}$, ${V}_{{{{\rm{B}}}}}$). **e** Measured ${I}_{{{{\rm{OUT}}}}}$ of the sensory circuit for the OR logic operation and **f** the AND logic operation under 460 nm illumination with predefined light intensity levels for objects X and Y. **g** Reconstructed 5×5 pixel matrices demonstrating image integration (OR logic, top) and intersection (AND logic, bottom), highlighting the in-sensor computing capability of the proposed sensory circuit.

The T-HTR's ability to generate and modulate multiple logic states via electrical and optical inputs enables in-sensor computing, where data processing occurs directly within the sensor, minimizing energy consumption for data transmission and storage. As a proof-of-concept, a sensory circuit consisting of four CsF-based T-HTRs with a 60 nm-thick CsF layer was fabricated as a pixel-level sensory circuit, where two pairs of T-HTRs were first connected in series, and these two serially connected pairs were then integrated in parallel (Fig. 5a). Each T-HTR functions as a signal-switching element, dynamically controlling the connection between circuit pathways (Fig. 5b). Specifically, each T-HTR can operate in three distinct configurations: (1) in the off-state, the transistor remains non-conductive, leaving the circuit open (disconnected); (2) in the intermediate state, the transistor exhibits a photodiode-like behavior, where the light signal is converted into an electrical signal, partially connecting the circuit; and (3) in the on-state, the transistor forms a direct, low-resistance connection. This circuit configuration enables light-induced ${I}_{{{{\rm{OUT}}}}}$ to be processed through either the series or parallel path, depending on the input voltage applied to two pairs of T-HTRs (${V}_{{{{\rm{A}}}}}$ and ${V}_{{{{\rm{B}}}}}$), thereby enabling diverse image processing tasks within a single circuit architecture. For instance, when two distinct light signals from objects X and Y are input into two respective T-HTR, the sensory circuit can be dynamically reconfigured to process these light signals in OR logic mode (${V}_{{{{\rm{A}}}}}$ for off-state; ${V}_{{{{\rm{B}}}}}$ for intermediate state) or AND logic mode (both ${V}_{{{{\rm{A}}}}}$ and ${V}_{{{{\rm{B}}}}}$ for intermediate state), depending on the ${V}_{{{{\rm{A}}}}}$ and ${V}_{{{{\rm{B}}}}}$ (Fig. 5c, d).

To conceptually evaluate the image-processing capability of the sensory circuit, two 5×5 pixel matrices representing objects X and Y were arbitrarily constructed, where each pixel was assigned one of three predefined light intensity levels: dark (0 mW cm^-2^), 0.68 mW cm^-2^, and 2.17 mW cm^-2^. Then, to correlate these intensities with circuit responses, the ${I}_{{{{\rm{OUT}}}}}$ was measured under predefined light intensity sets, where two T-HTRs detected light from object X (${P}_{{{{\rm{inc}}}},{{{\rm{X}}}}}$), and the other two detected light from Y (${P}_{{{{\rm{inc}}}},{{{\rm{Y}}}}}$) (Fig. 5c). Specifically, the ${I}_{{{{\rm{OUT}}}}}$ was measured as a function of ${V}_{{{{\rm{A}}}}}$ while fixing ${V}_{{{{\rm{B}}}}}$ at -8 V to implement OR logic (Fig. 5e) and as a function of ${V}_{{{{\rm{A}}}}}$ while setting ${V}_{{{{\rm{B}}}}}$ equal to ${V}_{{{{\rm{A}}}}}$ to realize AND logic (Fig. 5f). The measured photocurrent responses corresponding to each light intensity were then mapped onto the pixel matrices to simulate image processing. This mapping shows that OR logic enables image integration by combining all illuminated regions of objects X and Y, whereas AND logic extracts the overlapping regions, representing image intersection (Fig. 5g). The reconstructed images demonstrate that the proposed sensory circuit successfully executes image processing tasks, highlighting its potential for in-sensor computing applications where logic operations are directly performed within the sensor layer.

## Discussion

In summary, we present a light-driven ternary heterojunction transistor (T-HTR) that enables both optical and electrical control, achieving three stable states within a single device. The T-HTR directly encodes variable optical signal characteristics into a discrete logic state, thereby reducing the need for complex multi-transistor configurations. This capability is realized by integrating a vertical p–n heterostructure with an engineered injection barrier at the source, which establishes a light-tunable intermediate state characterized by zero transconductance (${g}_{{{{\rm{m}}}}}$ = 0).

Consequently, a single T-HTR inverter can be reconfigured to operate as a standard, positive, or negative ternary inverter (STI, PTI, NTI) – marking the first demonstration of all three ternary inverter modes within a single device. Moreover, we demonstrate a pixel-level sensory circuit as a proof-of-concept for in-sensor computing, where multifunctional image-processing tasks, such as pixel integration and intersection, are performed through dynamic logic reconfiguration.

Overall, the T-HTR exemplifies an optoelectronic logic paradigm that exploits the tunable nature of light and bridges ternary logic with sensory data processing. Although the present platform remains at a proof-of-concept stage, particularly with respect to operating speed, its speed-power trade-off is well aligned with ultralow-power sensing and in-sensor computing applications, where reduced static power and operation steps can be more critical than high clock frequency. Further progress toward practical implementation will require lithographic device scaling to reduce parasitic capacitance, reproducible fabrication of uniform heterojunction/barrier stacks, and array-level integration with localized or integrated light sources. In parallel, optimizing the injection-barrier thickness and interfacial energy-level alignment, while reducing barrier-associated contact resistance, will be essential for minimizing the speed–power trade-off and preserving a sufficient intermediate-state margin for robust ternary logic operation. With these advances, this material-adaptable architecture will provide a pathway toward compact, energy-efficient optoelectronic systems for reconfigurable in-sensor computing.

## Methods

### Materials and substrate

1,3,5-trimethyl-1,3,5-trivinyl cyclotrisiloxane (V3D3 monomer; 95%) was purchased from Gelest, and tert-butyl peroxide (TBPO; 97%) was purchased from Sigma-Aldrich for fabricating the ultrathin polymer dielectric layer. The 2,9-Didecyldinaphtho[2,3-b:2’,3’-f]thieno[3,2-b]thiophene (C10-DNTT; 99%) p-type semiconductor was purchased from Luminescence Technology Corp. (Lumtec) and N,N′-ditridecyl-perylene-3,4,9,10-tetracarboxylic diimide (C13-PTCDI; 95%) n-type semiconductor was purchased from Sigma-Aldrich. Molybdenum trioxide (MoO_3_; 99.95 %) and Cesium fluoride (CsF; 99 %) were purchased from Sigma-Aldrich. All the chemicals were used as received without further purification. Glass substrate of 25 × 25 mm was cleaned with deionized (DI) water, acetone, and isopropyl alcohol sequentially for 15 min under ultrasonication, followed by blowing with dry N_2_ gas.

### Polymer dielectric deposition

Polymer dielectric layers were deposited by an iCVD system. For poly[1,3,5-trimethyl-1,3,5-trivinyl cyclotrisiloxane] (pV3D3) deposition, V3D3 and TBPO were vaporized and injected into the iCVD chamber at flow rates of 2.5 and 1 standard cubic centimeter per minute (sccm), respectively. The substrate temperature and chamber pressure were maintained at 40 °C and 300 mTorr, respectively. The filament was heated to 130 °C to decompose the initiator into radicals for both processes.

### Thin film characterization

The thickness of the organic semiconductors and dielectric layer was measured using a spectroscopic ellipsometer (M2000, J. A. Woollam, USA). Information about the band structure of the used materials was obtained from the literature. The absorbance of the 20 nm-thick C10-DNTT and 20 nm-thick C13-PTCDI layers at normal incidence was measured in ambient air using a UV-Vis spectrometer (UV-2550, SHIMADZU).

### Device fabrication

For gate electrodes, 50 nm-thick Al electrodes were thermally deposited at a deposition rate of ~1 Å s^-1^. The 50 nm-thick pV3D3 gate dielectric layer was deposited by the iCVD process, and the thickness was measured using an ellipsometer after the deposition. For the n-type semiconductor, 20 nm-thick C13-PTCDI was deposited by thermal evaporation at a deposition rate of 0.2–0.3 Å s^-1^. For the p-type semiconductor, 20 nm-thick C10-DNTT was deposited at a deposition rate of 0.2–0.3 Å s^-1^. During the deposition, the substrate temperature was maintained at 60 °C and the thickness was monitored in-situ by quartz crystal microbalance (QCM). Finally, 70 nm-thick Au source/drain electrodes were thermally deposited at the deposition rate of ~0.7 Å s^-1^ through a shadow mask with channel dimensions of 500 (L) × 1000 (W) μm. For n-type T-HTR, MoO_x_ injection barrier was deposited between C10-DNTT and Au source electrodes. For p-type T-HTR, CsF injection barrier was deposited between C13-PTCDI and Au source electrodes. All the thermal evaporation was carried out at a chamber pressure lower than 1 × 10^-6^ Torr.

### Device characterization

The electrical properties of the unit devices and logic gates were characterized using the semiconductor parametric analyzers (HP 4155 A and B1500A, Agilent Technologies, Inc.) under controlled dark ambient conditions. The optoelectrical properties were measured by exposing a collimated beam from blue and green LEDs to the top of the devices. The peak wavelengths of the blue and green LEDs are 460 nm and 535 nm, respectively, and the beam size was large enough to cover the entire device area. All the device fabrication, including thermal evaporation and iCVD processes, as well as all measurements, were performed in an inert N_2_-filled glove box.

## Supplementary information

Supplementary Information

Transparent Peer Review file

## Source data

Source data

## Data Availability

Source data are provided with this paper.
