# Supplementary material for: A light-driven multi-state heterojunction transistor for optoelectronic ternary logic circuits
Source: Nat Commun. 2026 Jun 19;17:7736. doi: 10.1038/s41467-026-74238-y (PMC13434661; doi:10.1038/s41467-026-74238-y)
Supplement: Supplementary file 1 [file 41467_2026_74238_MOESM1_ESM.pdf]

Supplementary Information

A light-driven multi-state heterojunction transistor for optoelectronic ternary logic circuits

Chungryeol Lee,[1,†] Dongho Choi,[2,†] Sanghoon Park,[2,†] Jeong-ik Park,[1] Taehyun Nam,[1] Minjae Jang,[1] Seunghyup Yoo,[2,3,*] and Sung Gap Im[1,3,*]

[1] Department of Chemical and Biomolecular Engineering
Korea Advanced Institute of Science and Technology (KAIST)
Daejeon 34141, Republic of Korea

[2] School of Electrical Engineering
Korea Advanced Institute of Science and Technology (KAIST)
Daejeon 34141, Republic of Korea

[3] Graduate School of Semiconductor Technology
Korea Advanced Institute of Science and Technology (KAIST)
Daejeon, 34141 Republic of Korea

Correspondence and requests for materials should be addressed to S.G.I. (sgim@kaist.ac.kr) or S.Y. (syoo_ee@kaist.ac.kr).

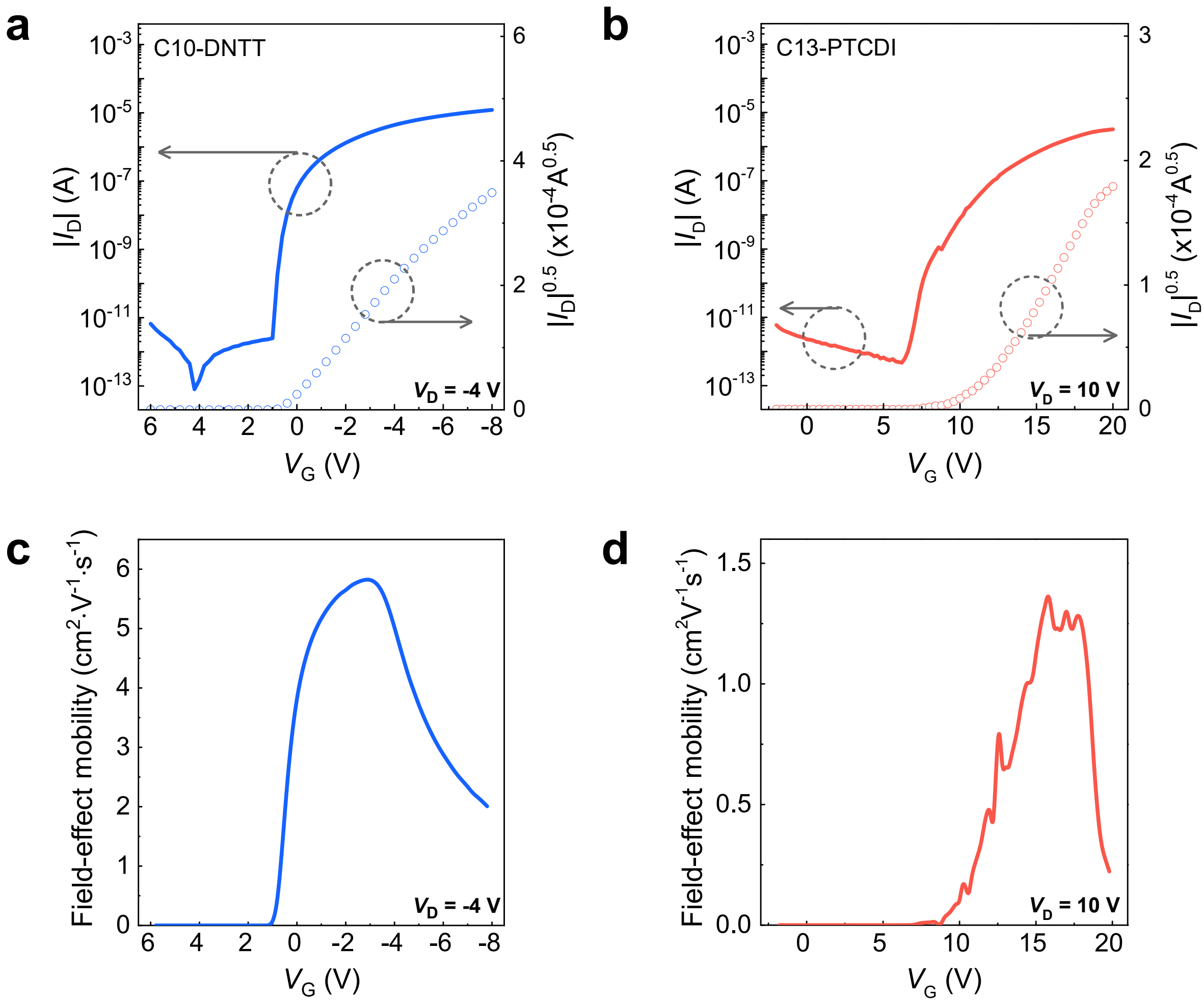

Supplementary Figure 1 | Electrical characteristics of thin-film transistors. **a**. Transfer characteristics of 2,9-Didecyldinaphtho-[2,3-b:2',3'-f]thieno[3,2-b]thiophene (C10-DNTT) and **b**. N,N′-Ditridecyl-3,4,9,10-perylenetetracarboxylic diimide (C13-PTCDI) thin-film transistors. **c**. Field-effect mobility (μ) profiles of C10-DNTT and **d**. C13-PTCDI thin-film transistors, extracted from their transfer characteristics.

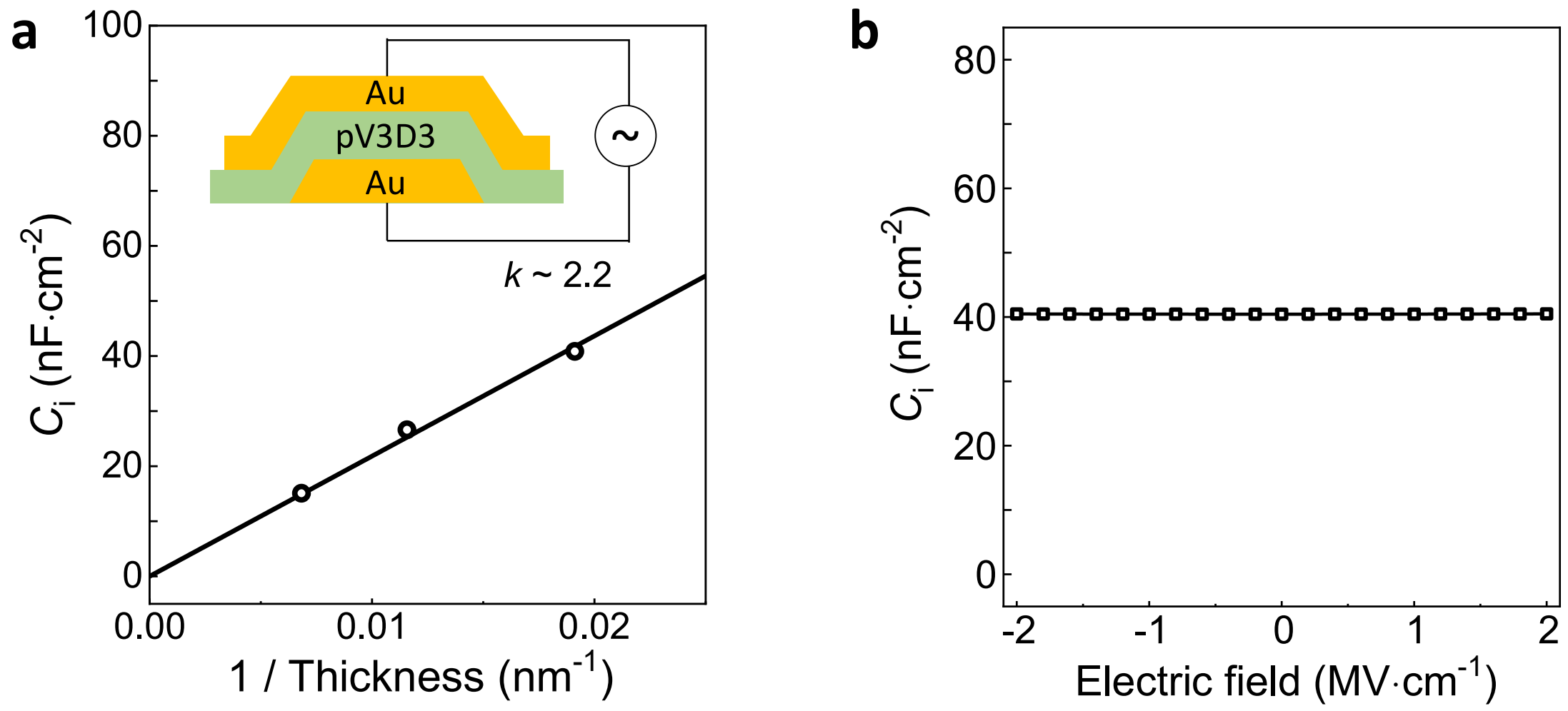

Supplementary Figure 2 | Dielectric properties of poly(1,3,5-trivinyl-1,3,5-trimethyl cyclotrisiloxane) (pV3D3) films. **a**. Capacitance per unit area (C_i) versus inverse dielectric thickness ($1/d$) of Au/pV3D3/Au devices for dielectric constant (k) extraction. **b**. C_i versus electric field (E_i) of an Au/pV3D3 (50 nm)/Au device.

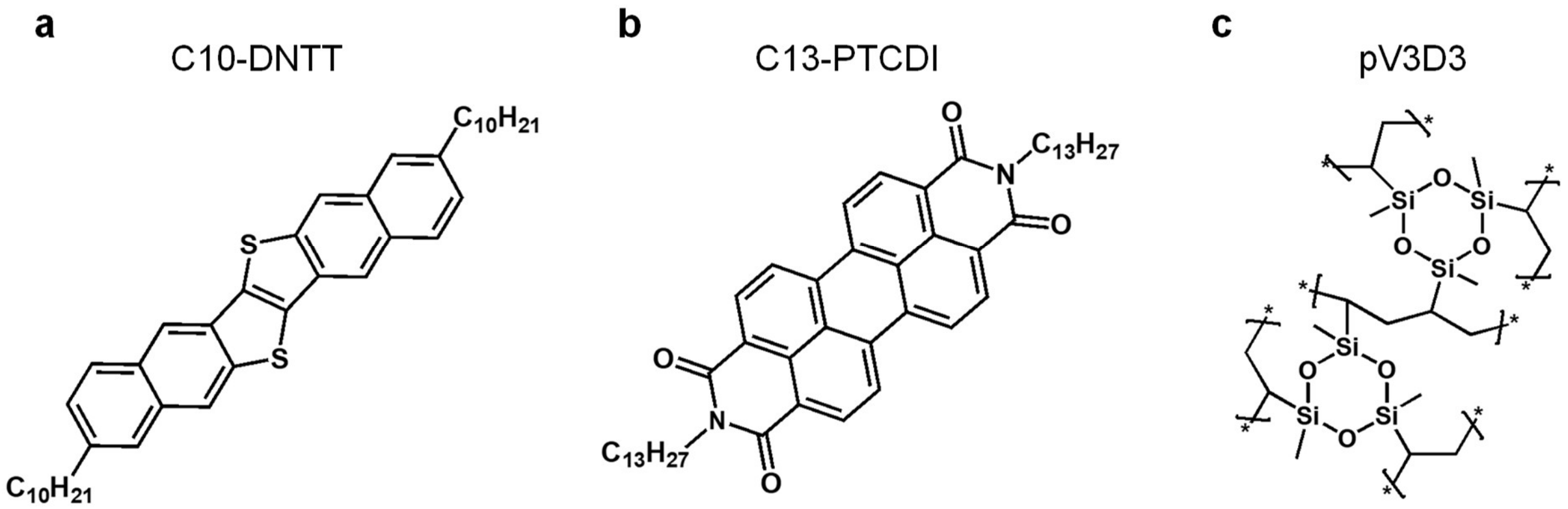

Supplementary Figure 3 | Chemical structures of the materials used in this study. **a**. Chemical structures of C10-DNTT, **b**. C13-PTCDI and **c**. pV3D3.

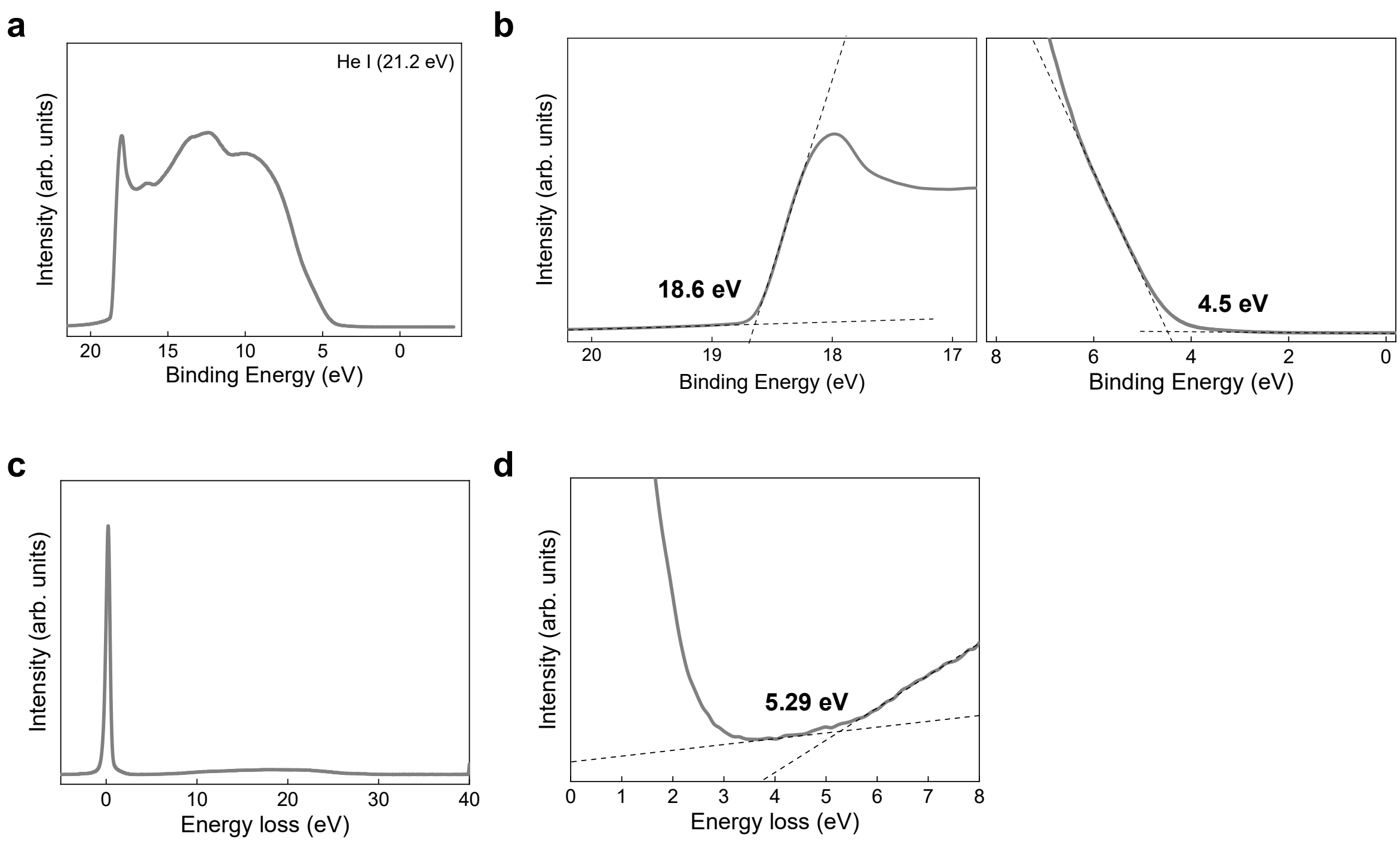

Supplementary Figure 4 | Electronic structure characterization of the Cesium fluoride (CsF) layer. **a**. Ultraviolet photoelectron spectroscopy (UPS) spectra of the CsF. **b**. The enlarged UPS spectra at the highest binding energy cut-off (left) and the lowest binding energy cut-off (right). **c**. Reflected electron energy loss spectroscopy (REELS) spectra of CsF. **d**. The enlarged REELS spectra.

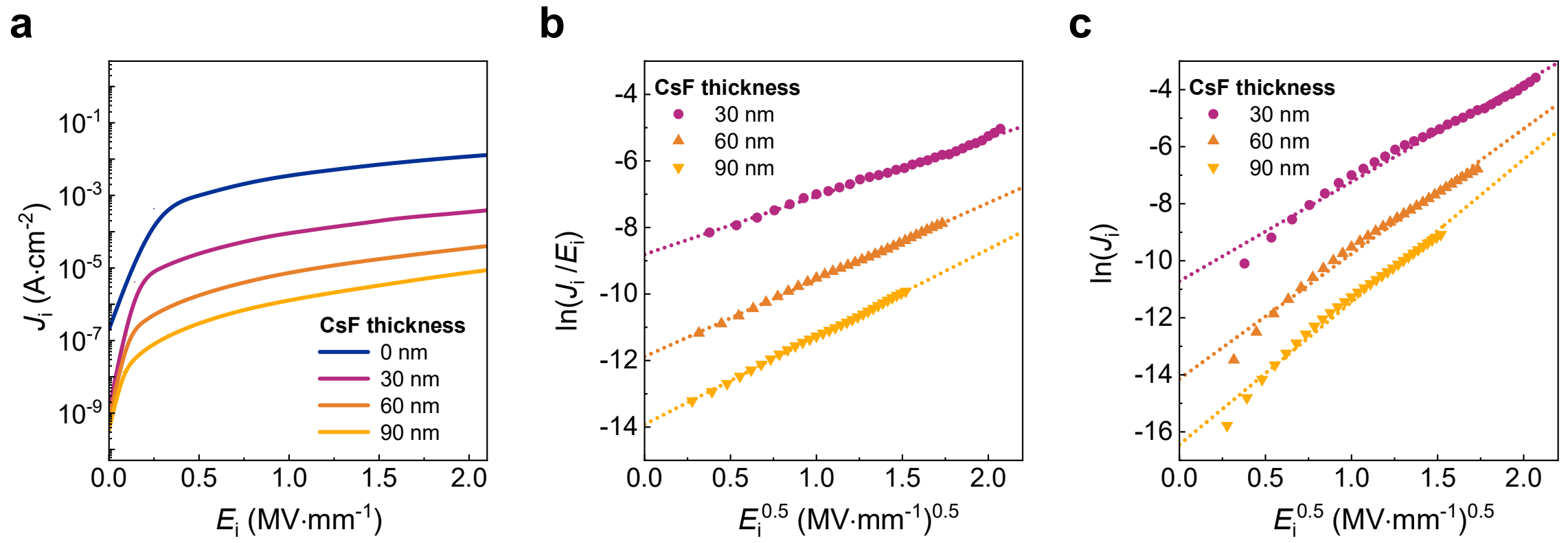

Supplementary Figure 5 | Charge transport through the CsF injection barrier. **a**. Current density–electric field (J_i–E_i) characteristics of the Au/C10-DNTT (20 nm)/C13-PTCDI (20 nm)/CsF (0 to 90 nm)/Au devices with different CsF thicknesses, showing a systematic reduction of current with increasing barrier thickness. **b**. Poole-Frenkel plots and **c**. Schottky plots derived from the J_i–E_i data.

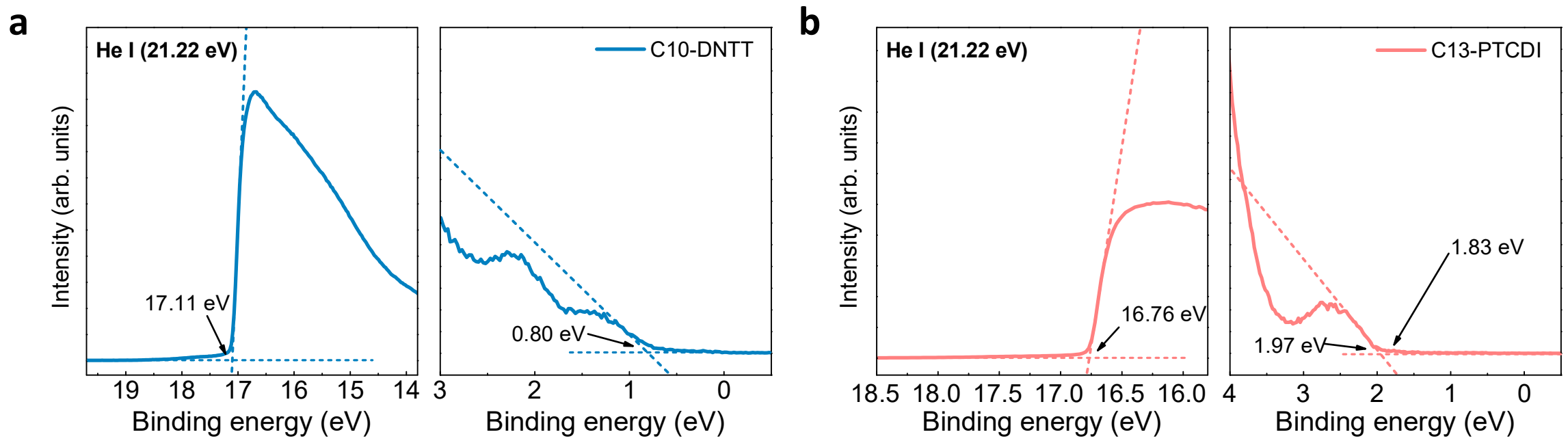

Supplementary Figure 6 | Electronic energy levels of the semiconductors.

a. UPS spectra of C10-DNTT and **b**. C13-PTCDI thin films.

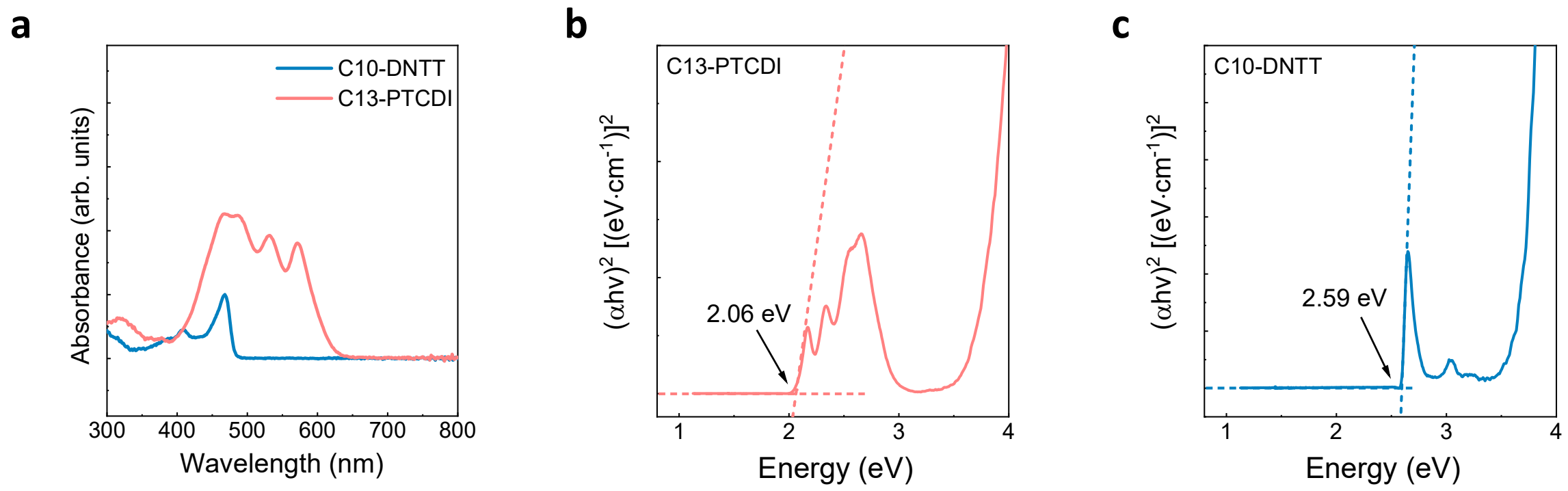

Supplementary Figure 7 | Optical bandgap characterization of the semiconductors. a. Ultraviolet-visible (UV-vis) absorption spectra of the thin films. Corresponding Tauc plots of **b**. C13-PTCDI and **c**. C10-DNTT thin films, used to extract the optical bandgap values.

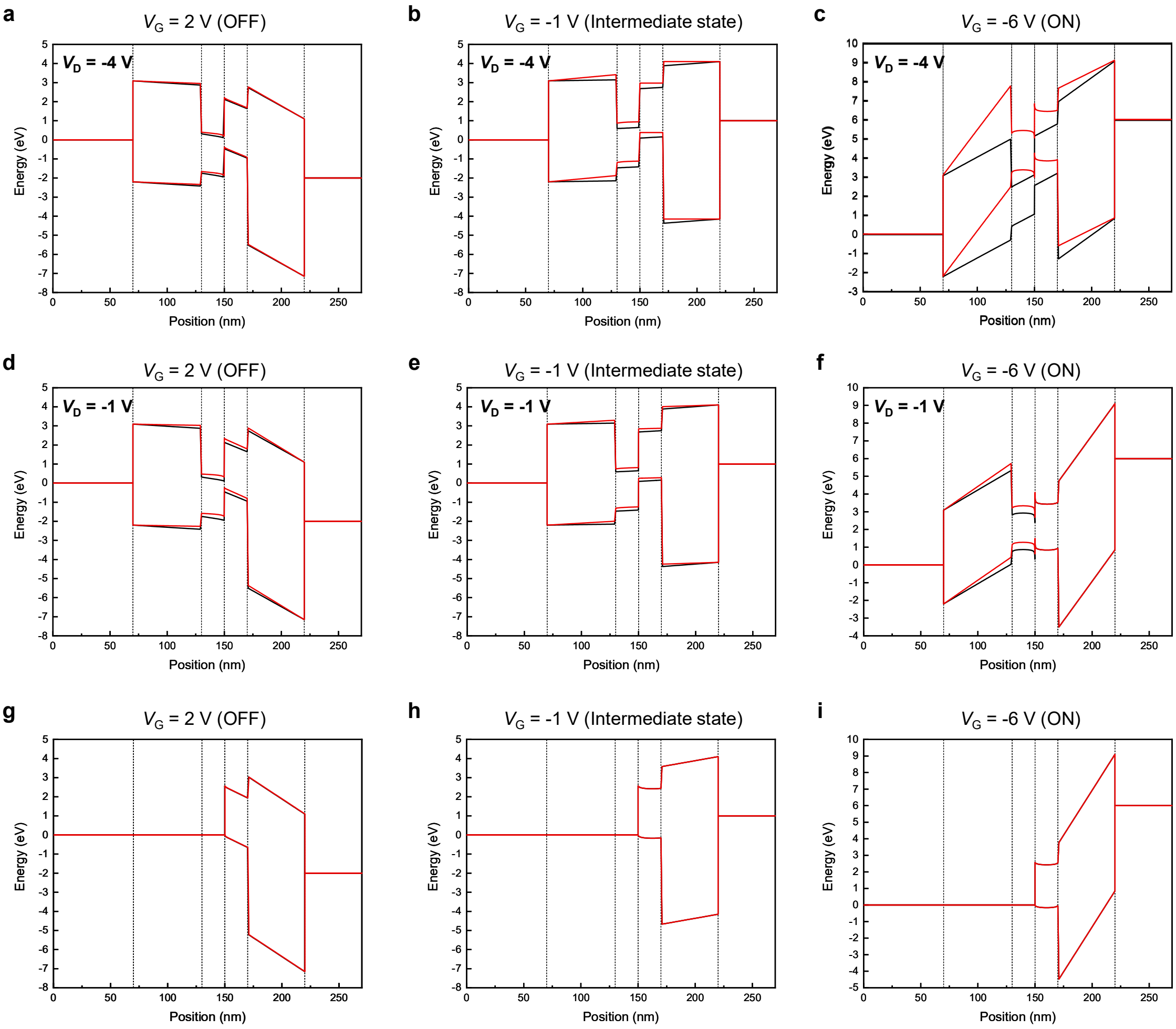

Supplementary Figure 8 | Simulated band diagrams of p-type ternary heterojunction transistor (T-HTRs). Simulated energy band diagrams of the scaled p-type T-HTRs at the source-side, in the dark (black lines) and under illumination (red lines) at three representative gate voltage (V_G) states: **a**. V_G = 2 V (OFF), **b**. V_G = -1 V (intermediate), and **c**. V_G = -6 V (ON). For **a**-**c**, drain voltage (V_D) = -4 V. Corresponding diagrams at V_D = -1 V are shown in **d**-**f**. Corresponding diagrams for the reversed source/drain configuration are shown in **g**-**i**. For **g**-**i**, V_D = -4 V. The input parameters used for the simulations are summarized in Supplementary Table 2.

In the off-state, the channel remains fully depleted, preventing the formation of a conductive pathway; as a result, charge transport is effectively suppressed and photogenerated carriers cannot percolate through the channel, yielding negligible current even under illumination. In the intermediate state, the engineered injection

barrier at the source suppresses gate-induced hole injection while allowing photogenerated carriers to flow, thereby producing a gate-voltage-independent photocurrent plateau with zero transconductance ($g_{\mathrm{m}} \approx 0$). In the on-state, a sufficiently strong V_{G} reduces the source-side hole-injection barrier and enables efficient hole transport through the p-type channel, with the photocurrent providing an additional contribution to the total conduction.

The drain bias (V_{D}) also influences the source-side band. At a small V_{D}, the potential drop along the channel is minor, so the field across the CsF is mainly governed by the V_{G}. In this regime, trap-assisted conduction dominates with a relatively small field enhancement. As V_{D} increases, part of the drain potential also drops across the source-side dielectric stack, effectively increasing the internal field in CsF. This enhanced field strengthens Poole-Frenkel conduction by lowering the effective barrier:

$$\emptyset_{\mathrm{B}} - \sqrt{eE_{\mathrm{eff}}\pi^{-1}\varepsilon_i{}^{-1}} \quad (1)$$

where $\emptyset_{\mathrm{B}}$ is the barrier height, e is the elementary charge, E_{eff} is the effective electric field, and ε_i is the dielectric permittivity. As a result, the current density increases according to:

$$J \propto E_{\mathrm{eff}} \times \exp\left(E_{eff}{}^{0.5}\right) \quad (2)$$

Here, E_{eff} represents the combined field composed of the gate-induced component and the additional field induced by the drain bias and photogenerated interfacial charge accumulation. When the source and drain are reversed, the source directly contacts the p-type channel without the injection barrier, enabling hole injection and resulting in behavior analogous to that of a conventional transistor without an intermediate state.

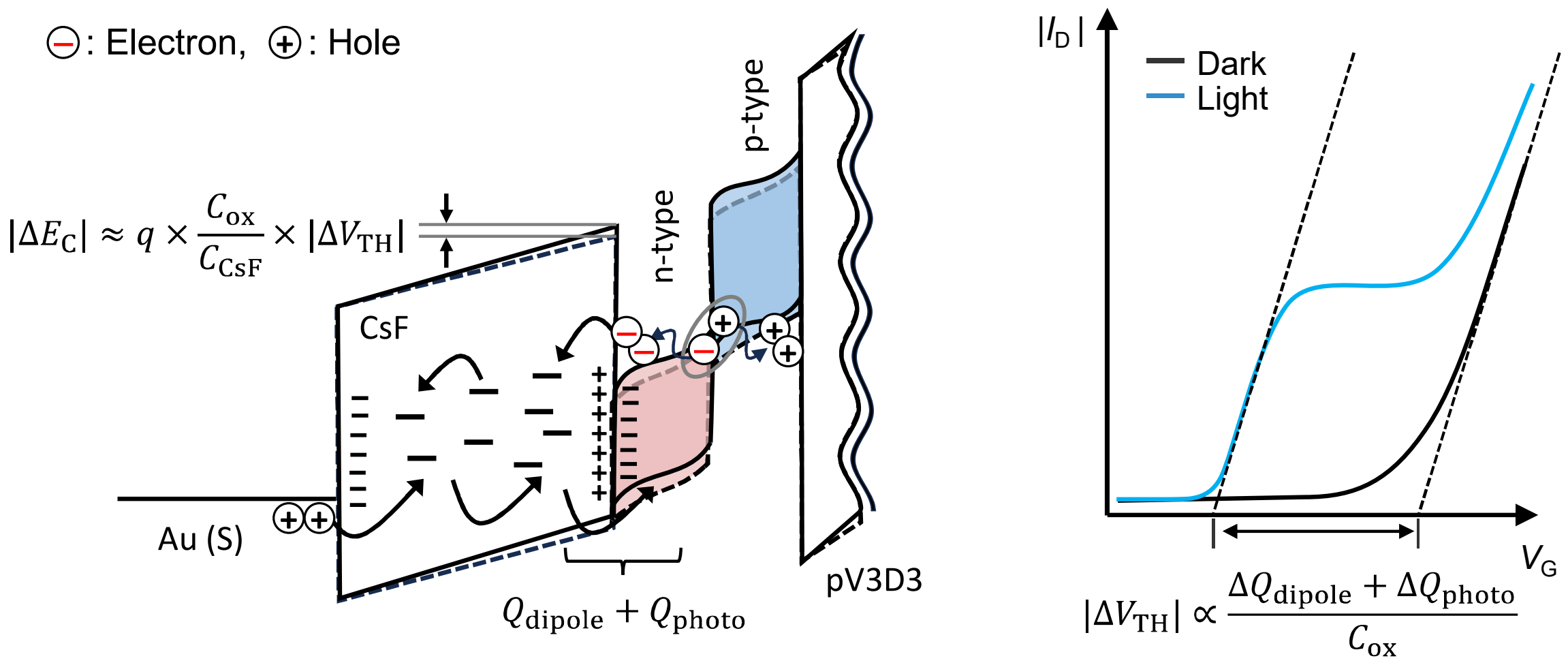

Supplementary Figure 9 | A schematic illustration of the illumination-induced threshold voltage (V_{TH}) shift.

Under illumination, excitons at the heterojunction interface dissociate: photogenerated minority carriers are driven toward the source and accumulate/trap beneath the blocking layer, while photogenerated majority carriers are transported through the channel toward the drain. In the p-type T-HTR, the accumulated/trapped photogenerated electrons at the semiconductor/CsF interface induce a positive charge on the CsF side, forming an interfacial dipole moment. The accumulated interfacial charge and dipole create an internal photovoltage and band bending that modulate the contact, which redistributes the series potential drop at a fixed V_G, increasing the fraction that falls across the CsF layer:

$$\Delta V_{CsF} \approx (C_{ox}/C_{CsF}) \times |\Delta V_{TH}| \quad (3)$$

where C_{ox} and C_{CsF} denote the capacitances per unit area of the gate dielectric and CsF layer, respectively, and ΔV_{TH} represents the light-induced shift in threshold voltage arising from interfacial charge accumulation and dipole formation. Therefore, conduction band on the organic/CsF side shifts by

$$\Delta E_c \approx q\Delta V_{CsF} \approx q\left(\frac{C_{ox}}{C_{CsF}}\right)|\Delta V_{TH}| \quad (4)$$

while the pV3D3 energy band correspondingly flattens. Consequently, the gate bias required for channel formation decreases and ΔV_{TH} shifts accordingly to negative direction in p-type T-HTR (and positively for n-type T-HTRs):

$$|\Delta V_{\mathrm{TH}}| \propto \frac{\Delta Q_{\mathrm{dipole}} + \Delta Q_{\mathrm{photo}}}{C_{\mathrm{ox}}} \quad (5)$$

where $\Delta Q_{\mathrm{dipole}}$ represents the charge separation-induced interfacial dipole moment and $\Delta Q_{\mathrm{photo}}$ denotes the accumulated/trapped photogenerated charge.

As the light intensity increases, the numerator ($\Delta Q_{\mathrm{dipole}} + \Delta Q_{\mathrm{photo}}$) increases, leading to a gradual decrease in V_{TH}. When trap sites beneath the barrier become saturated, the internal photovoltage no longer increases and V_{TH} saturates, converging to the value of the control device without the blocking layer. This indicates that the barrier layer raises the initial V_{TH} in the dark by suppressing minority carrier injection from the source, while strong illumination compensates for this barrier via light-induced contact modulation.

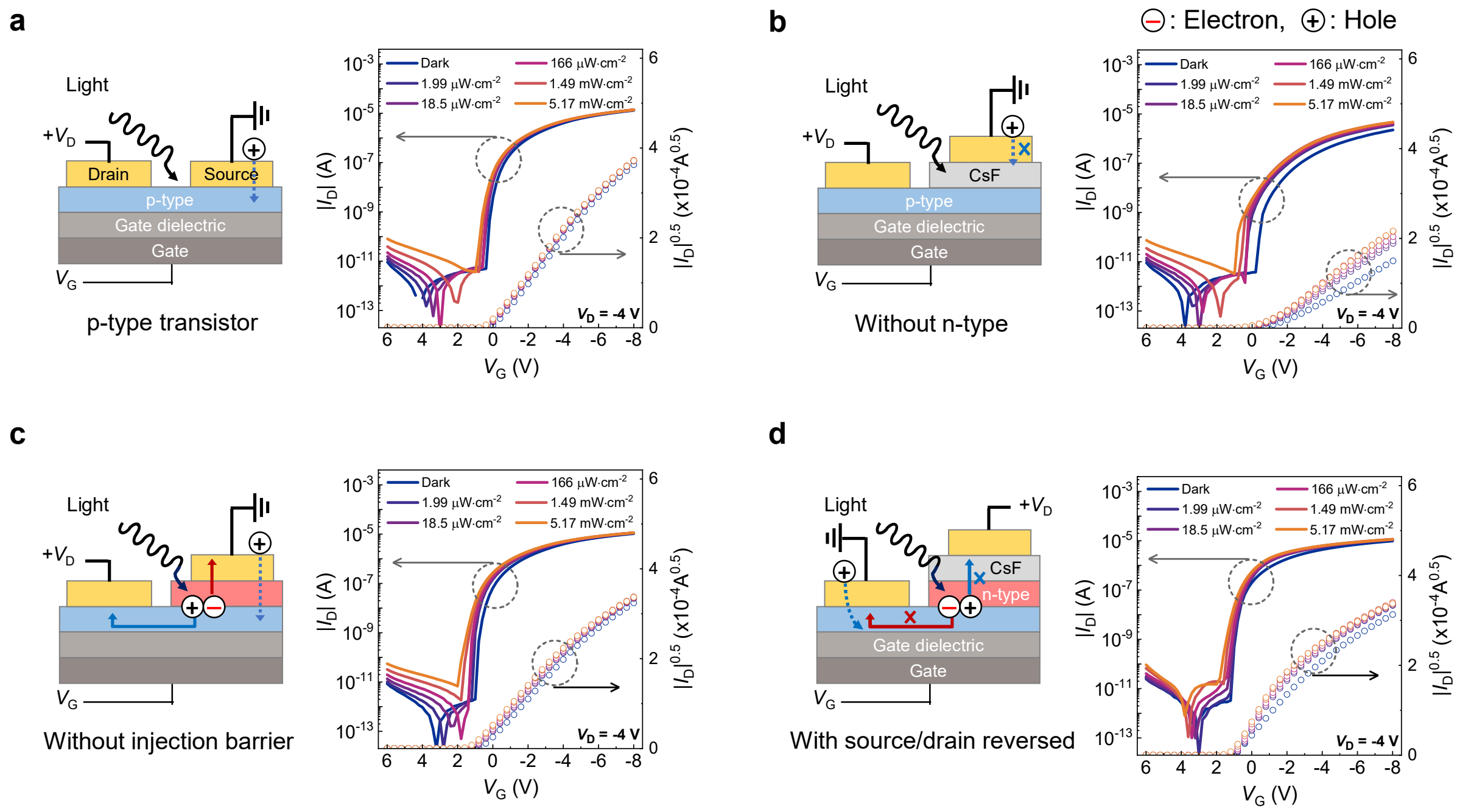

Supplementary Figure 10 | Transfer characteristics of control devices. a. Transfer characteristics of a p-type transistor, **b**. the T-HTR without a sub-semiconductor, **c**. the T-HTR without an injection barrier, and **d**. the T-HTR with the source and drain electrodes reversed.

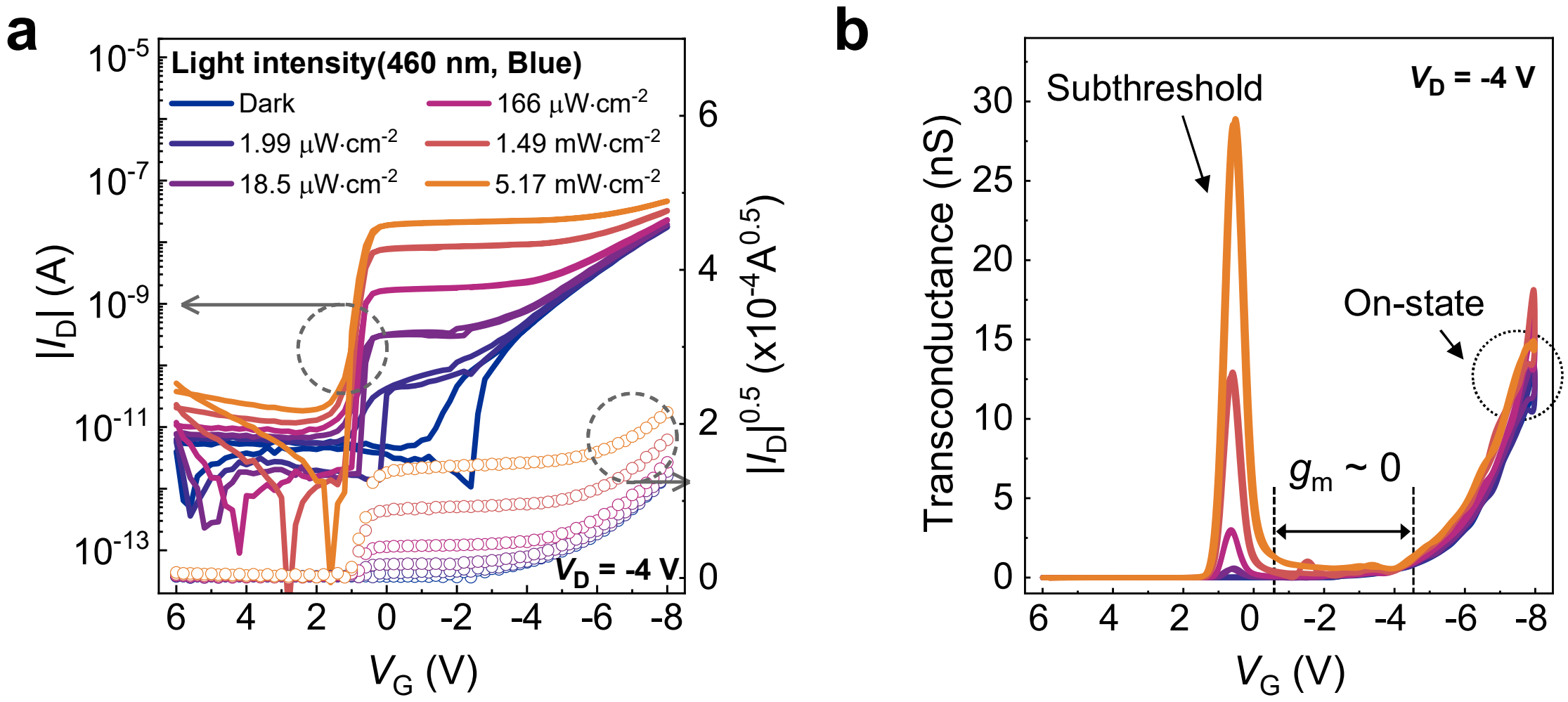

Supplementary Figure 11 | Electrical characterization of the T-HTR. **a**. The transfer characteristics and **b**. g_{m} of the T-HTR under 460 nm illumination as a function of light intensity.

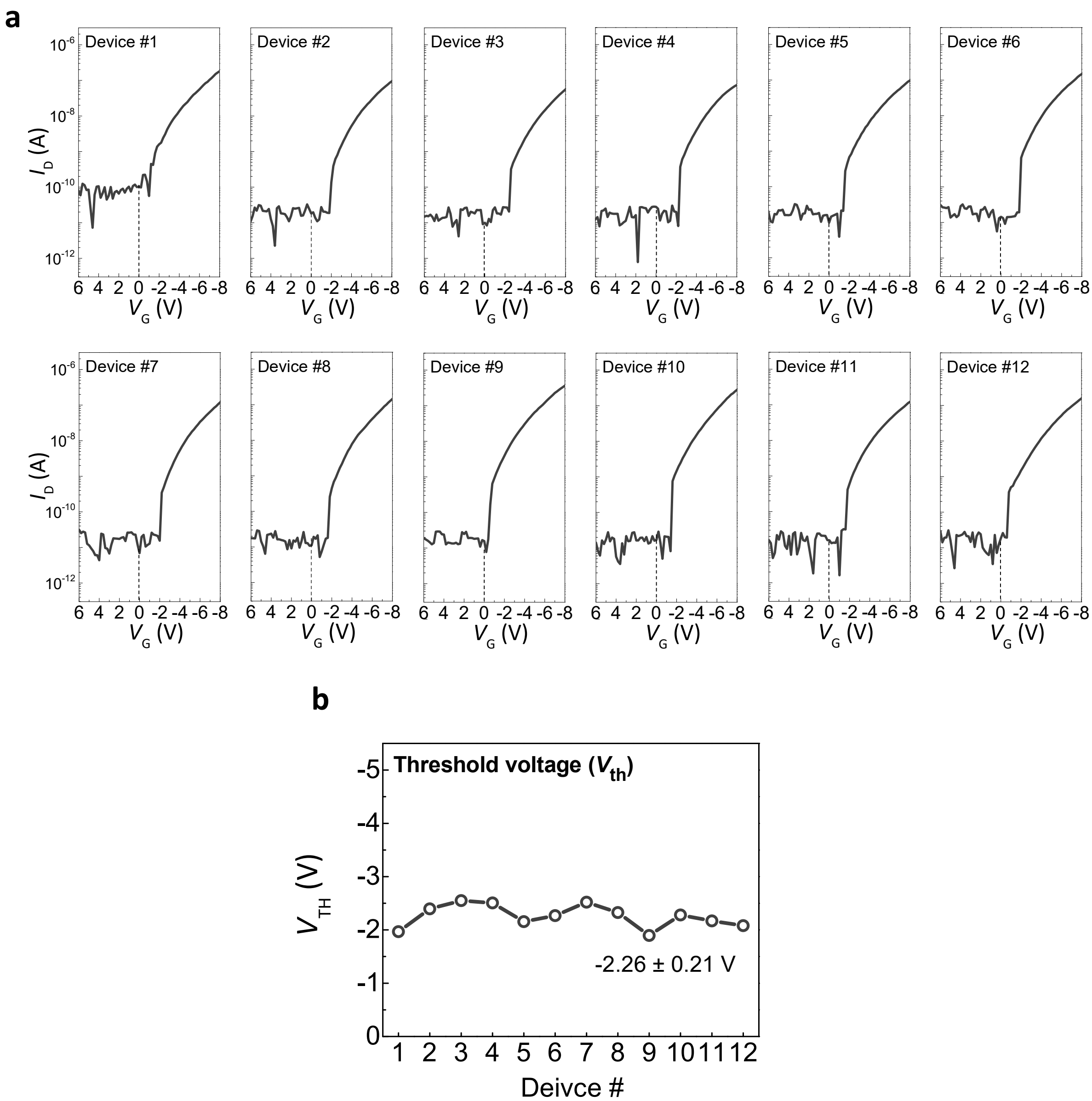

Supplementary Figure 12 | Device-to-device uniformity of the T-HTR. **a**. Transfer characteristics of 12 T-HTRs under dark conditions. **b**. Extracted V_{TH} of the T-HTRs.

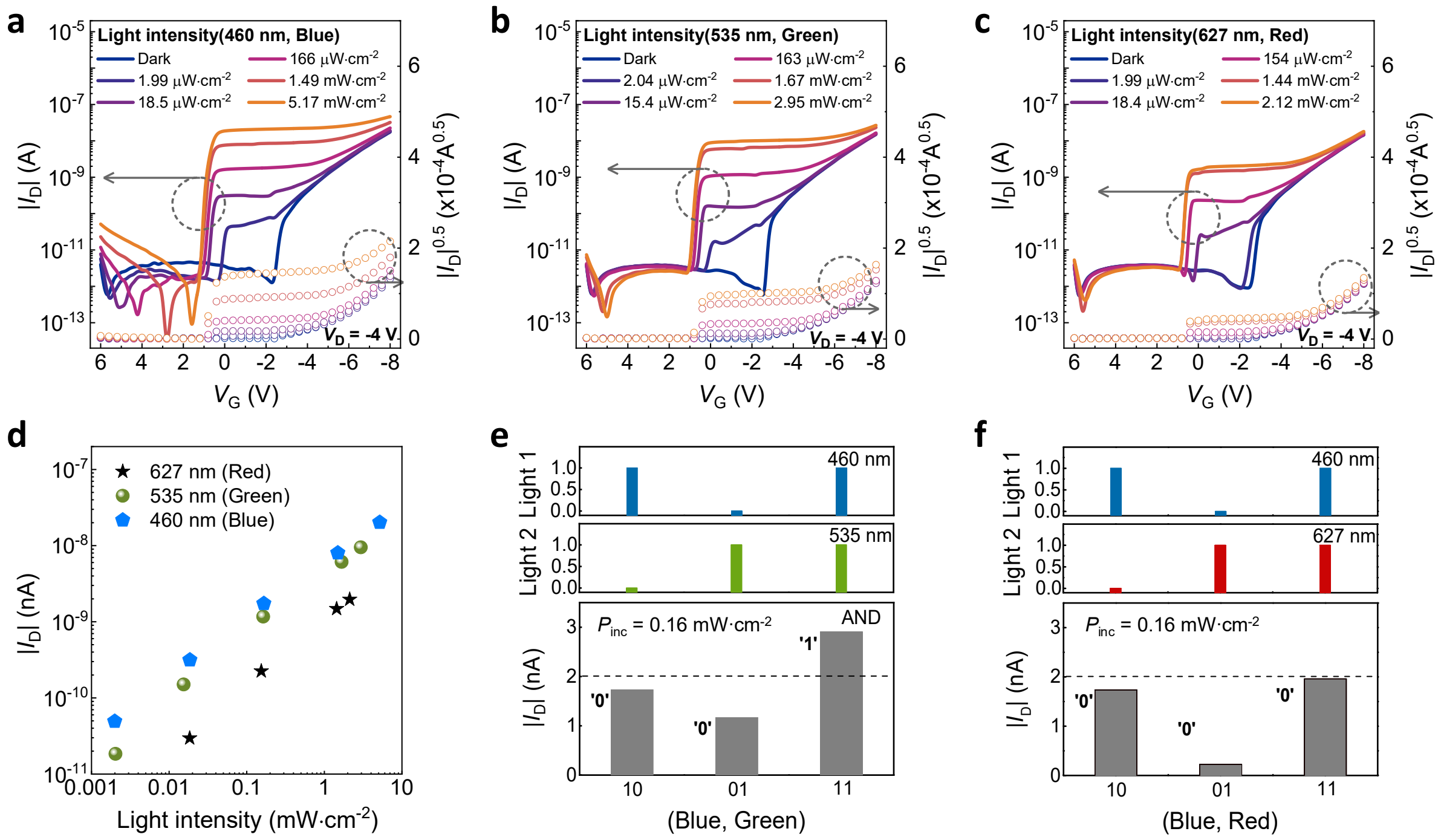

Supplementary Figure 13 | Wavelength-selective operation of the T-HTR. **a**. Transfer characteristics of the T-HTR under illumination at 460 nm (blue), **b**. 535 nm (green), and **c**. 627 nm (red). **d**. Extracted drain current (I_D) in the intermediate state under illumination at 460 nm, 535 nm, and 627 nm. **e**. Schematic illustration of the wavelength-selective logic operations of the T-HTR under 460 nm and 535 nm illumination, and **f**. 460 nm and 627 nm illumination at an incident light power density (P_{inc}) of 0.16 mW·cm^{-2}.

Under monochromatic illumination, double-sweep transfer curves showed that the photocurrent magnitude decreased in the order of 460 nm (blue), 535 nm (green), and 627 nm (red) illumination (**Supplementary Fig. 13a-d**).

Furthermore, these observations indicate that Boolean logic can be realized only under specific wavelength combinations at identical light intensity (**Supplementary Fig. 13e, f**). For instance, when both blue and green lights are simultaneously illuminated, the sum of their individual photocurrents exceeds the predefined threshold current (I_{th}), thereby yielding an output corresponding to the AND logic condition. In contrast, when blue and red illumination are applied simultaneously, the photocurrent generated by red illumination is too small; hence,

the combined photocurrent remains below I_{th}, and the AND logic condition cannot be satisfied. In other words, the AND operation is achievable only under certain wavelength combinations where the cumulative photocurrent surpasses the threshold. These representative results suggest that, in general, specific wavelength and moderate intensity tuning can allow reliable Boolean logic operations.

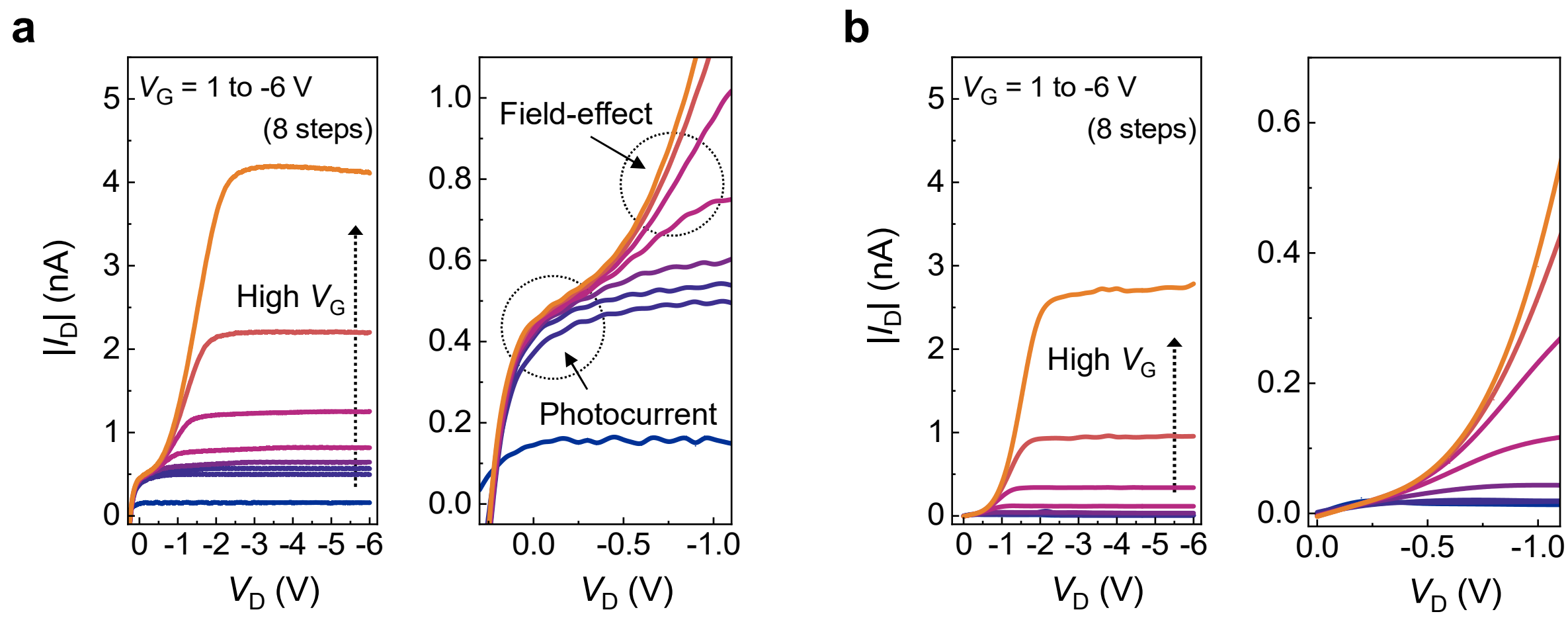

Supplementary Figure 14 | Output characteristics of the p-type T-HTR. **a**. Output characteristics of the T-HTR under 166 μW·cm^{-2} of 460 nm light illumination and **b**. under dark conditions.

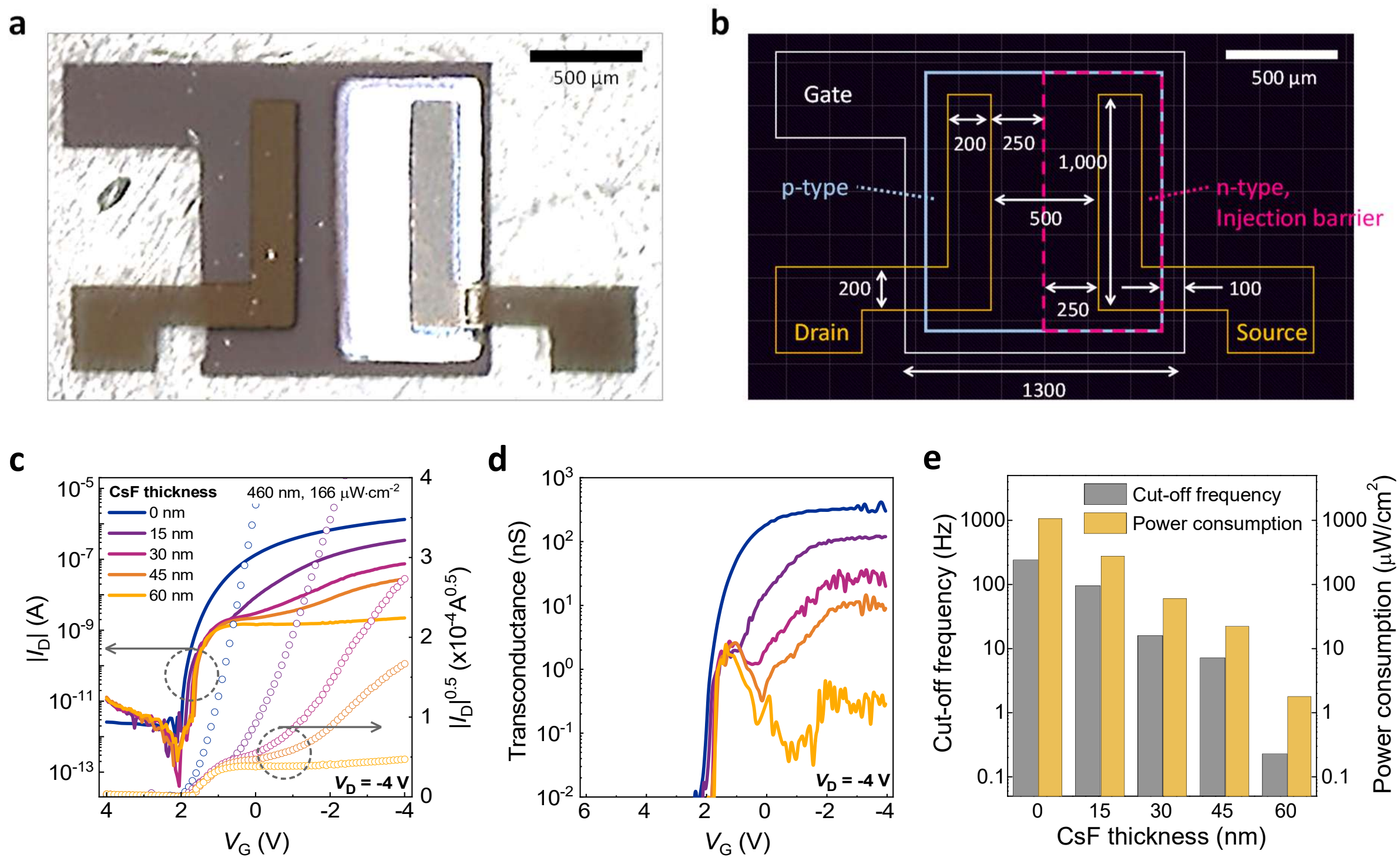

Supplementary Figure 15 | Speed–power trade-off controlled by CsF thickness. **a**. An optical microscopy image (scale bar: 500 μm) and **b**. shadow mask layout of the T-HTR (scale bar: 500 μm). **c**. The transfer characteristics, **d**. g_{m}, and **e**. cut-off frequency (f_{T}) versus static power consumption of the T-HTR as a function of CsF barrier thickness. Increasing the CsF thickness reduces both the cut-off frequency and power consumption, illustrating the speed-power trade-off governed by injection barrier engineering.

As the CsF thickness increased from 15 nm to 60 nm, the estimated f_{T} decreased from 10^2-10^3 Hz to below 1 Hz, whereas the on-state (dynamic) power density dropped from the mW cm^{-2} scale to the sub-μW cm^{-2} scale. Such a speed–power compromise is highly relevant for application domains where ultralow static power is prioritized over high operating frequency, including always-on optical sensors, wearable biomedical monitors, and distributed environmental sensor nodes typically operating in the Hz–kHz regime[1~8]. Furthermore, the ternary-logic framework intrinsically reduces the number of computational steps by ≈63 % (log2 / log3), offering additional energy savings[9].

In the present proof-of-concept devices, the channel structures were defined using a shadow-mask process, which inevitably results in relatively large device geometries and substantial gate-electrode overlap areas. Under such conditions, the estimated cut-off frequency is strongly influenced by the geometrical capacitance associated with these overlap regions. Therefore, minimizing gate-electrode overlap through lithographic patterning could provide a practical pathway for reducing parasitic capacitance and improving the operating frequency in future device implementations.

These analyses demonstrate that injection-barrier engineering provides a practical and tunable design handle to balance operation speed and power efficiency in light-driven multi-state logic systems.

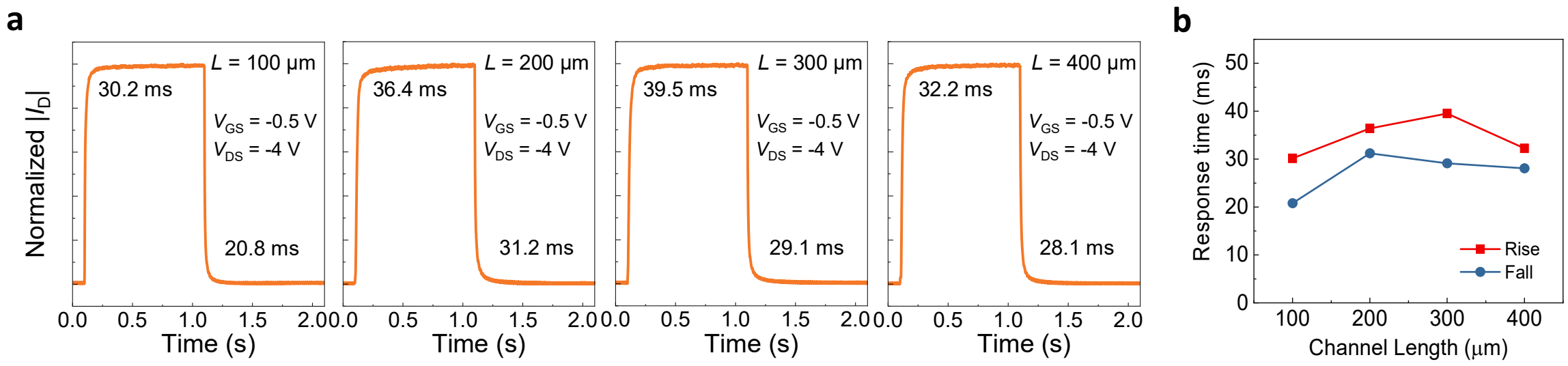

Supplementary Figure 16 | Channel length dependence of the transient response. **a**. Transient responses of the T-HTR for channel lengths (L) of 100 µm, 200 µm, 300 µm, and 400 µm. The drain current is normalized to its maximum value. **b**. The extracted rise and fall times as a function of L.

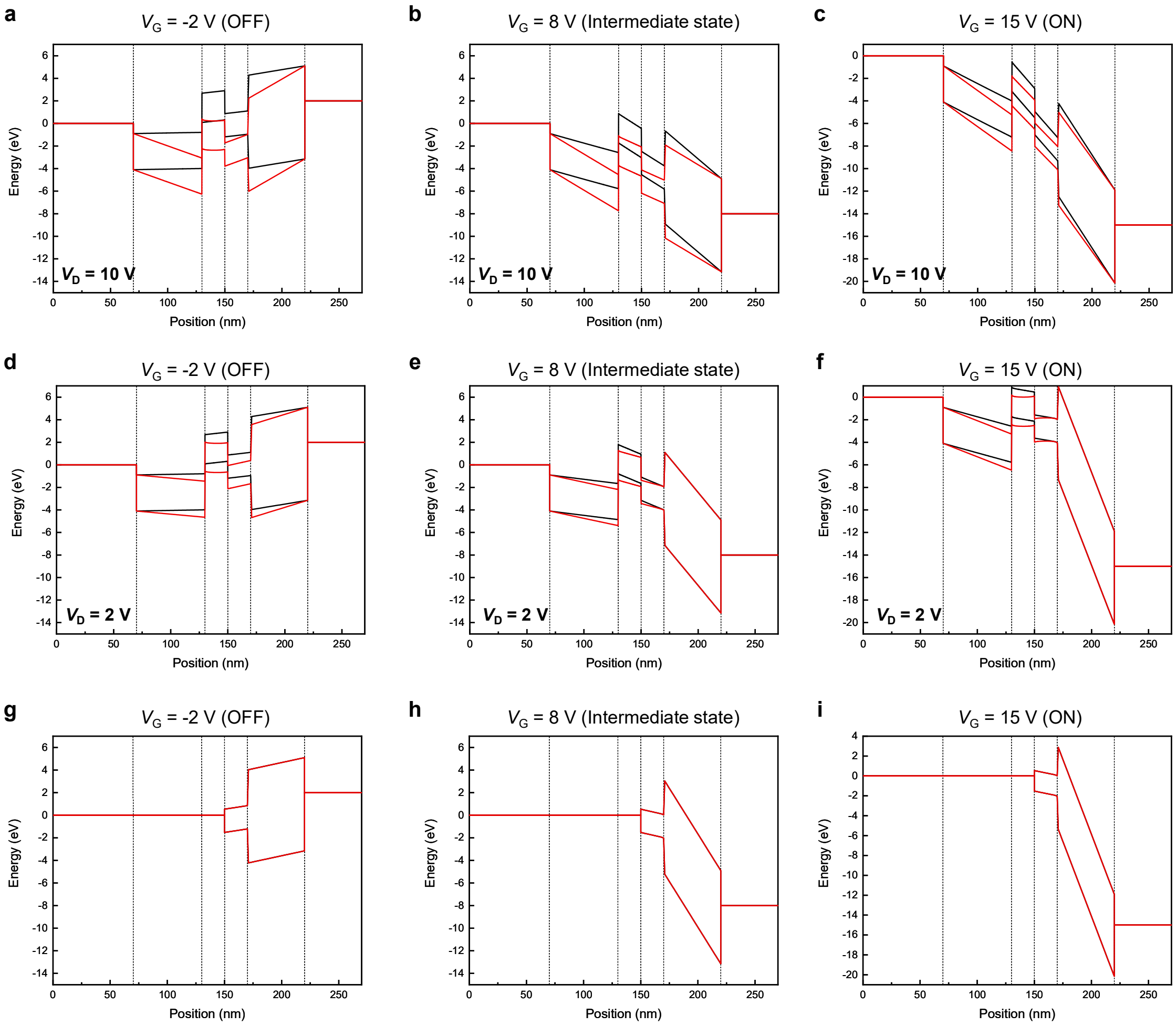

Supplementary Figure 17 | Simulated band diagrams of n-type T-HTRs. Simulated energy band diagrams of the scaled n-type T-HTRs at the source-side, in the dark (black lines) and under illumination (red lines) at three representative V_G states: **a**. V_G = -2 V (OFF), **b**. 8 V (intermediate), and **c**. 15 V (ON). For **a**-**c**, V_D = 10 V. Corresponding diagrams at V_D = 2 V are shown in **d**-**f**. Corresponding diagrams for the reversed source/drain configuration are shown in **g**-**i**. For **g**-**i**, V_D = 10 V. The input parameters used for the simulations are summarized in Supplementary Table 2.

In the off-state, the channel is fully depleted and both electrically and optically generated carriers are unable to form a percolating path, yielding negligible current. In the intermediate state, the molybdenum oxide (MoO_X) injection barrier suppresses gate-induced electron injection from the source while allowing photogenerated holes to be extracted, producing a light-driven, V_G-independent

current plateau ($g_{\mathrm{m}} \approx 0$). In the on-state, a sufficiently positive gate bias lowers the hole-injection barrier and enables efficient hole transport through the p-type channel, with photocurrent adding to the total conduction.

V_{D} additionally modulates the MoO_X barrier. At a low V_{D}, the field at the source contact is mainly V_{G}-controlled. At a larger V_{D}, part of the drain potential drops across the MoO_X/semiconductor stack, increasing the effective field and enhancing trap-assisted Poole-Frenkel conduction, thereby increasing current.

Reversing the source/drain terminals removes the MoO_x barrier at the injecting contact, permitting electron injection and resulting in conventional transistor behavior without an intermediate state.

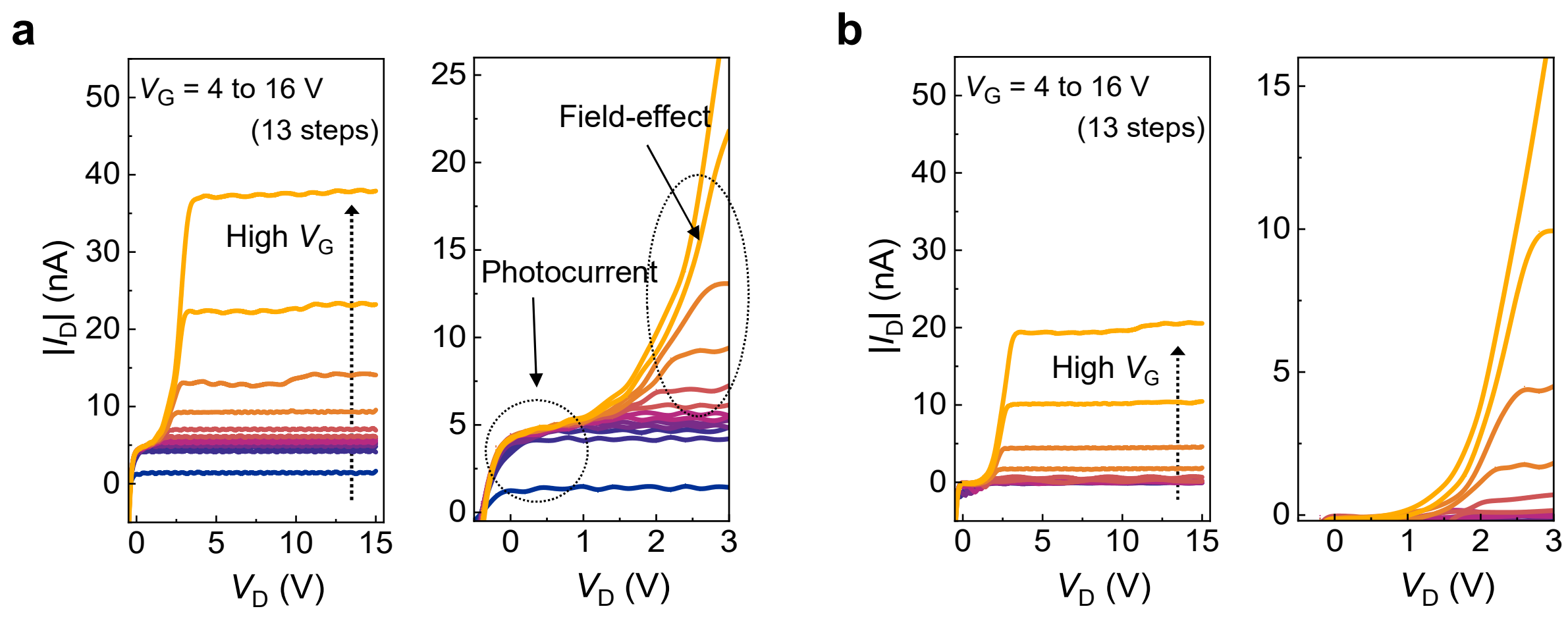

Supplementary Figure 18 | Output characteristics of the n-type T-HTR. a. Output characteristics of the n-type T-HTR under 166 μW·cm^{-2} of 460 nm light illumination and **b**. under dark conditions.

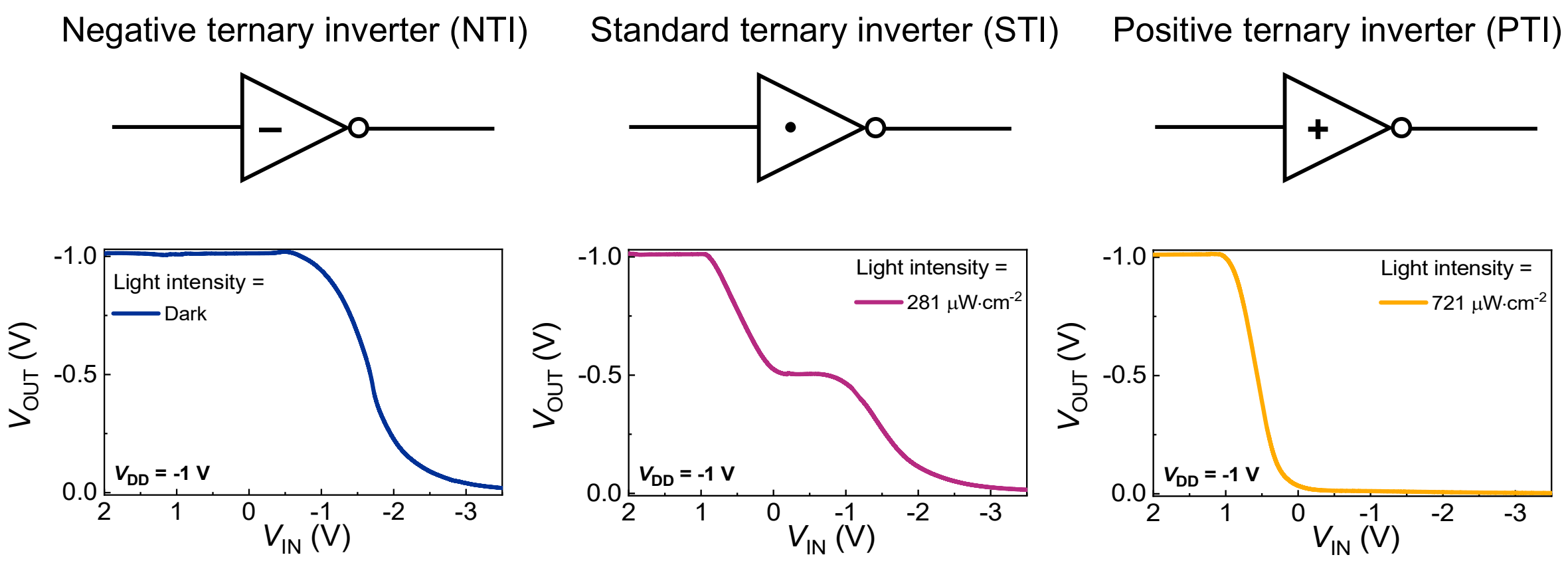

Supplementary Figure 19 | Multifunctionality of the proposed ternary logic inverter (T-inverter).

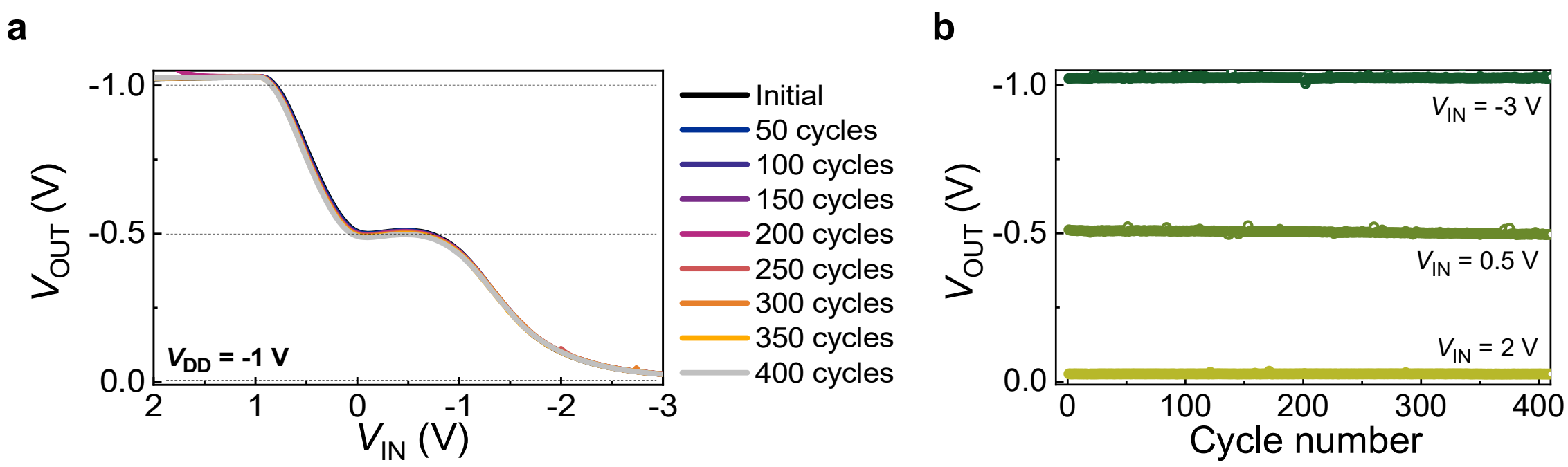

Supplementary Figure 20 | Cycle endurance of the T-inverter. **a**. Voltage transfer characteristics and **b**. extracted output voltage (V_{OUT}) at logic 0, logic 1, and logic 2 during 400 consecutive operations under 0.28 mW·cm^{-2} of 460 nm illumination.

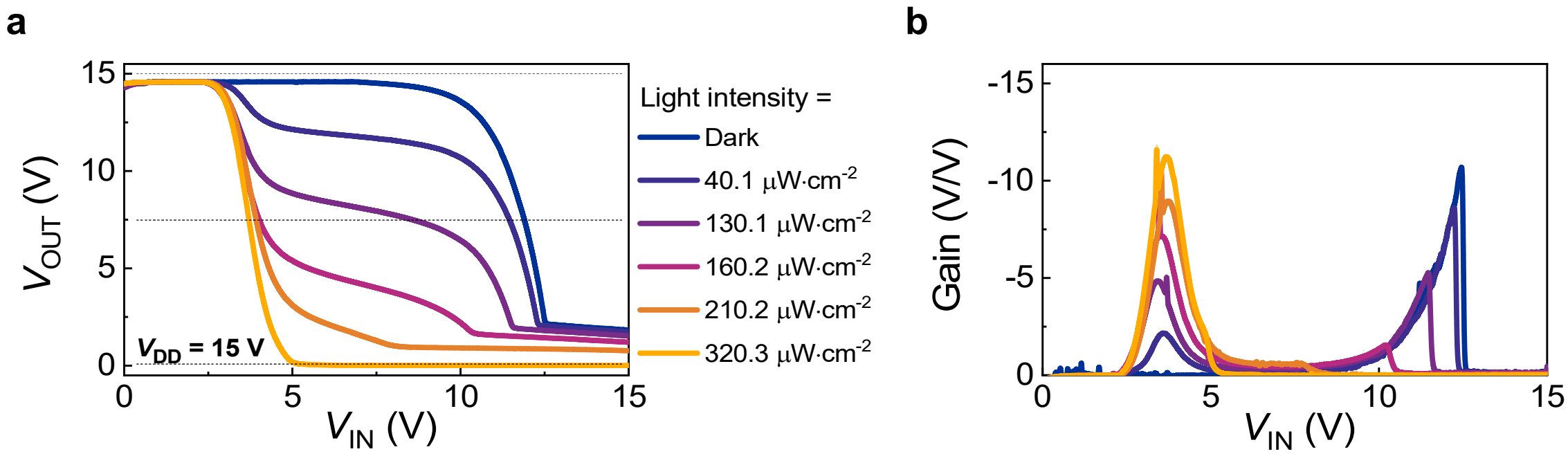

Supplementary Figure 21 | Electrical characteristics of the T-inverter based on an n-type T-HTR with a MoO_X injection barrier. **a**. Voltage transfer characteristics and **b**. voltage gain profiles of the T-inverter under 460 nm illumination with respect to light intensity.

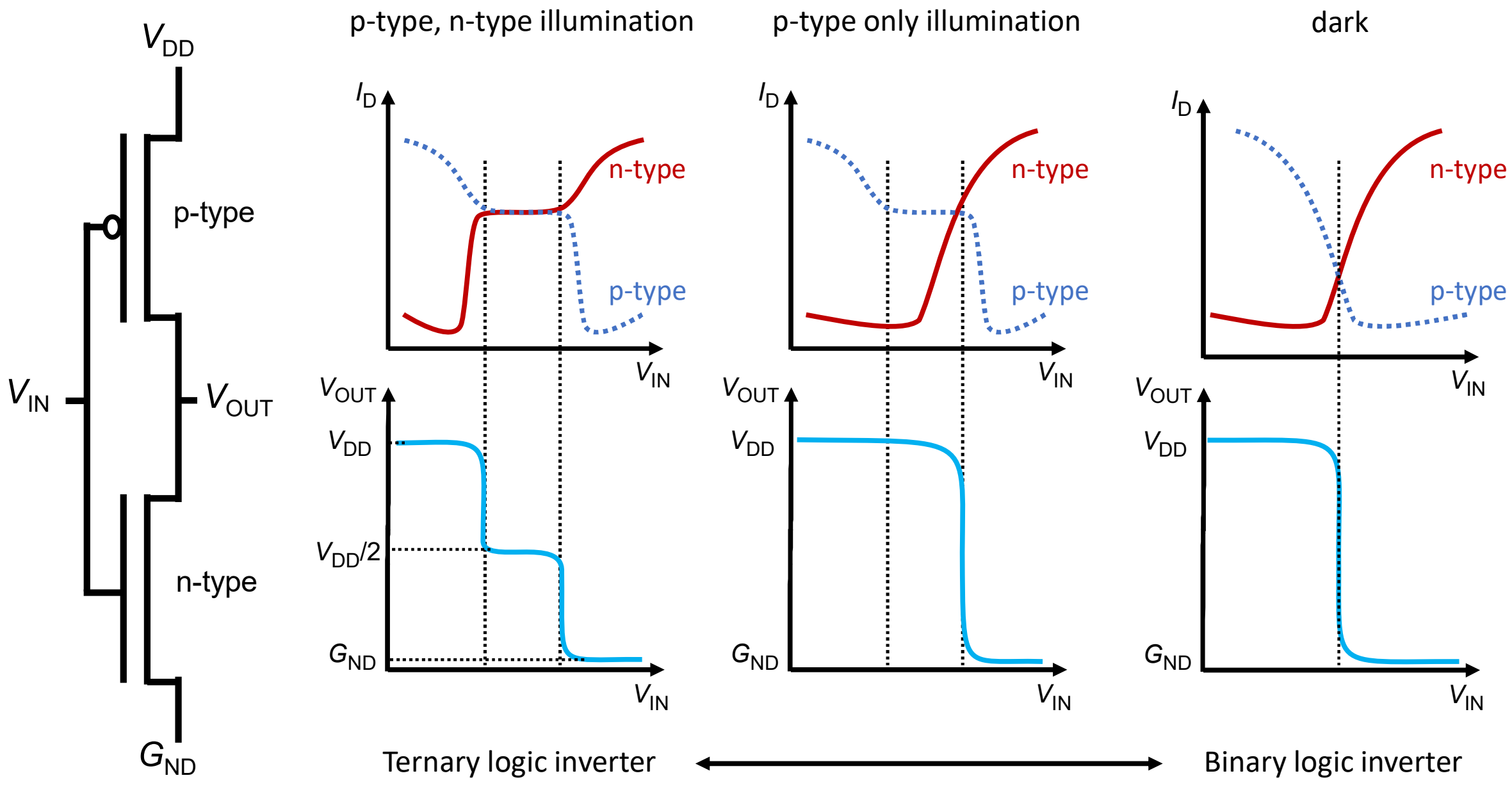

Supplementary Figure 22 | Conceptual schematic of voltage-output ternary logic gate configurations using complementary p-type and n-type T-HTRs under optical control.

It is noteworthy that our manuscript demonstrates not only the realization of p-type T-HTRs but also the successful implementation of n-type counterparts operating on the same working principle. This highlights the potential for constructing fully transistorized, voltage-output ternary logic gates based on a standard CMOS inverter topology.

For instance, both the p-type and n-type T-HTRs exhibit photo-induced intermediate states, which can be tuned such that their output currents are balanced to yield an intermediate logic level of approximately half the driving voltage (0.5 V_{DD}). In this complementary configuration, the p-type T-HTR acts as the pull-up element in its on-state, whereas the n-type T-HTR functions as the pull-down element, together realizing a standard ternary inverter topology. Moreover, the ability to optically toggle the intermediate states of each transistor provides an additional degree of freedom for logic design. When only one of the transistors is illuminated, the circuit transitions from the standard ternary inverter operation to either a positive or negative ternary inverter mode. In contrast, when both devices remain unilluminated, the circuit behaves as a conventional binary logic inverter.

This conceptual demonstration indicates that, similar to the current-based configuration, a variety of logic operations can also be achieved in the voltage-based scheme through optical control.

Supplementary Table 1 | Comparison of static power consumption with the previously reported ternary logic transistor.

Operating principle	Driving voltage at logic 1	Static current at logic 1	Channel (length × width)	Static power consumption at logic 1	Ref
Negative transconductance	20 V	> 10 nA	400 μm × 1,000 μm	> 50 μW cm^{-2}	[10]
Negative transconductance	3 V	> 1 nA	500 μm × 1,000 μm	> 0.6 μW cm^{-2}	[11]
Negative transconductance	> 30 V	> 100 nA	1,000 μm × 2,200 μm	> 136 μW cm^{-2}	[12]
Negative transconductance	1 V	> 0.1 nA	1 μm × 3 μm	> 3.3 mW cm^{-2}	[13]
Negative transconductance	2 V	> 1,000 nA	3 μm × 20 μm	> 3.3 W cm^{-2}	[14]
Zero transconductance	1 V	6 nA	100 μm × 150 μm	40 μW cm^{-2}	[15]
Zero transconductance	1 V	4 nA	60 μm × 80 μm	83 μW cm^{-2}	[16]
Zero transconductance	1 V	2 nA	1 μm × 55 μm	3.6 mW cm^{-2}	[17]
Zero transconductance	1 V	0.1 nA	1 μm × 1 μm	10 mW cm^{-2}	[18]
Zero transconductance	10 V	> 3 nA	100 μm × 1,000 μm	30 μW cm^{-2}	[19]
**Zero transconductance**	**4 V**	**0.004 nA**	500 μm × 1,000 μm	**3.2 pW** cm^{-2}	**This work**

Supplementary Table 2 | Truth table of the ternary logic gates.

Light	Input A	Input B	Config. a	Config. b	Config. c	Config. D
0	0	0	0	0	0	0
		1	0	0	0	0
		2	0	0	2	2
	1	0	0	0	0	0
		1	0	0	0	0
		2	0	0	2	2
	2	0	0	0	2	2
		1	0	0	2	2
		2	2	2	2	2
1	0	0	0	0	0	0
		1	0	0	1	1
		2	0	0	2	2
	1	0	0	0	**0**	**1**
		1	**0**	**1**	1	1
		2	**0**	**1**	2	2
	2	0	0	0	2	2
		1	1	1	2	2
		2	2	2	2	2
2	0	0	0	0	0	0
		1	0	0	2	2
		2	0	0	2	2
	1	0	0	0	**0**	**2**
		1	**0**	**2**	2	2
		2	**0**	**2**	2	2
	2	0	0	0	2	2
		1	2	2	2	2
		2	2	2	2	2

*Series connection for an AND gate with light applied either to one of the two T-HTRs (Config. a) or to both T-HTRs (Config. b), and parallel connection for an OR gate with light applied either to one of the two T-HTRs (Config. c) or to both T-HTRs (Config. d). The highlighted regions indicate the differences in output logic states depending on the number of illuminated devices.

Supplementary Table 3 | Parameters used for the TCAD simulation.

Material	Parameter	Value
Al	Work function	4.3 eV
	Refractive index (n) / Extinction coefficient (k) @460 nm	0.541 / 5.119
Au	Work function	4.9 eV
	Refractive index (n) / Extinction coefficient (k) @460 nm	1.394 / 1.828
C_{10}-DNTT	LUMO	2.33 eV
	HOMO	4.92 eV
	Band gap	2.59 eV
	Dielectric constant	3
	Electron mobility	10^{-3} $cm^2V^{-1}s^{-1}$
	Hole mobility	6 $cm^2V^{-1}s^{-1}$
	Doping concentration	10^{15} cm^{-3}
	Conduction band density of states (N_C)	10^{20} cm^{-3}
	Valence band density of states (N_V)	10^{20} cm^{-3}
	Refractive index (n) / Extinction coefficient (k) @460 nm	1.198 / 0.016
C_{13}-PTCDI	LUMO	4.36 eV
	HOMO	6.42 eV
	Band gap	2.06 eV
	Dielectric constant	3
	Electron mobility	1.3 $cm^2V^{-1}s^{-1}$
	Hole mobility	10^{-3} $cm^2V^{-1}s^{-1}$
	Conduction band density of states (N_C)	10^{20} cm^{-3}
	Valence band density of states (N_V)	10^{20} cm^{-3}
	Refractive index (n) / Extinction coefficient (k) @460 nm	1.370 / 0.066
CsF	Conduction band energy	1.81 eV
	Valence band energy	7.1 eV
	Band gap	5.29 eV
	Dielectric constant	7.9 [20]
	Refractive index (n) / Extinction coefficient (k) @460 nm	1.607 / ~ 0
MoOx	Conduction band energy	5.8 eV [21]
	Valence band energy	9.0 eV [21]
	Band gap	3.2 eV
	Dielectric constant	20 [22]
	Refractive index (n) / Extinction coefficient (k) @460 nm	2.065 / ~ 0
pV3D3	LUMO	1.2 eV [23]
	HOMO	9.45 eV [23]
	Band gap	8.25 eV
	Dielectric constant	2.2 [23]
	Refractive index (n) / Extinction coefficient (k) @460 nm	1.498 / ~ 0

TCAD simulations were conducted to generate the energy band diagrams, utilizing the input parameters listed in Supplementary Table 2. To model illumination effects, we

used the optical generation (optr) model with beam propagation (BPM) and transfer matrix (TR.MATRIX) methods, utilizing refractive indices (n, k) that were either experimentally measured or adopted from literature under 460 nm normal-incidence illumination.

Supplementary References

1 Dieffenderfer, J. *et al.*, Low-power wearable systems for continuous monitoring of environment and health for chronic respiratory disease. *IEEE journal of biomedical and health informatics*, 20(5), 1251-1264 (2016).

2 Kanoun, O. *et al.*, Energy-aware system design for autonomous wireless sensor nodes: A comprehensive review. *Sensors*, 21(2), 548 (2021).

3 Kim, H. *et al.*, Advances in Wireless, Batteryless, Implantable Electronics for Real-Time, Continuous Physiological Monitoring. *Nano-Micro Letters*, 16(1), 52 (2024).

4 Ebrahimi, Z. *et al.*, Ultralow-power photoplethysmography (PPG) sensors: A methodological review. *IEEE Sensors Journal*, 23(15), 16467-16480 (2023).

5 Tricoli, A. *et al.*, Wearable and miniaturized sensor technologies for personalized and preventive medicine. *Advanced Functional Materials*, 27(15), 1605271 (2017).

6 Zhang, T. *et al.*, Flexible electronics for cardiovascular healthcare monitoring. *The innovation*, 4(5), 100485 (2023).

7 Chong, Y. W. *et al.*, Energy harvesting for wearable devices: A review. *IEEE Sensors Journal*, 19(20), 9047-9062 (2019).

8 Gao, M. *et al.*, Power generation for wearable systems. *Energy & Environmental Science*, 14(4), 2114-2157 (2021).

9 Hurst., Multiple-valued logic—Its status and its future. *IEEE Transactions on computers*, 100, 1160-1179 (1984).

10 Shin, J. C. *et al.*, Oxide semiconductor heterojunction transistor with negative differential transconductance for multivalued logic circuits. *ACS nano*, 18(2), 1543-1554 (2024).

11 Lee, C. *et al.*, A reconfigurable binary/ternary logic conversion-in-memory based on drain-aligned floating-gate heterojunction transistors. *Nature Communications*, 14(1), 3757 (2023)

12 Yoo, H. *et al.*, Negative transconductance heterojunction organic transistors and their application to full‐swing ternary circuits. *Advanced Materials*, 31(29), 1808265 (2019)

13 Nourbakhsh, A. *et al.*, Transport properties of a MoS2/WSe2 heterojunction transistor and its potential for application. *Nano letters*, 16(2), 1359-1366 (2016)

14 Huang, M. *et al.*, Multifunctional high-performance van der Waals heterostructures. *Nature nanotechnology*, 12(12), 1148-1154 (2017)

15 Kim, J. *et al.*, Area-selective chemical doping on solution-processed MoS2 thin-film for multi-valued logic gates. *Nano letters*, 22(2), 570-577 (2021).

16 Kim, J. H. *et al.*, Carrier multiplication in van der Waals layered transition metal dichalcogenides. *Nature communications*, 10(1), 5488 (2019).

17 Han, J. *et al.*, Nanowire-based ternary transistor by threshold-voltage manipulation. *Applied Physics Letters*, 104(14) (2014)

18 Jeong, J. W. *et al.*, Tunnelling-based ternary metal–oxide–semiconductor technology. *Nature Electronics*, 2(7), 307-312 (2019)

19 Lim, D. U. *et al.*, Multi-state heterojunction transistors based on field-effect tunneling–transport transitions. *Advanced Materials*, 33(29), 2101243 (2021).

20 Hardy, J. *et al.*, Lattice Dynamics and Second-Order Raman Spectrum of CsF. *Physical Review*, 168(3), 1054 (1968).

21 Subbiah, J. *et al.*, MoO3/poly (9, 9-dioctylfluorene-co-N-[4-(3-methylpropyl)]-diphenylamine) double-interlayer effect on polymer solar cells. *Applied Physics Letters*, 96(6) (2010)

22 Holler, B. *et al.*, Holler, Brian2D Semiconductor Transistors with Van der Waals Oxide MoO3 as Integrated High-κ Gate Dielectric A., et al. 2D semiconductor transistors with van der Waals oxide MoO3 as integrated high-κ gate dielectric. *Advanced Electronic Materials*, 6(10), 2000635 (2020).

23 Moon, H. *et al.*, Synthesis of ultrathin polymer insulating layers by initiated chemical vapour deposition for low-power soft electronics. *Nature materials*, 14(6), 628-635 (2015).
