# Supplementary material for: A light-driven multi-state heterojunction transistor for optoelectronic ternary logic circuits
Source: Nat Commun. 2026 Jun 19;17:7736. doi: 10.1038/s41467-026-74238-y (PMC13434661; doi:10.1038/s41467-026-74238-y)
Supplement: Supplementary file 2 [file 41467_2026_74238_MOESM2_ESM.pdf]

nature portfolio

Peer Review File

A light-driven multi-state heterojunction transistor for optoelectronic ternary logic circuits

Corresponding Author: Professor Sung Gap Im

This file contains all reviewer reports in order by version, followed by all author rebuttals in order by version.

Attachments originally included by the reviewers as part of their assessment can be found at the end of this file.

Version 0:

Reviewer comments:

Reviewer #1

(Remarks to the Author)
The author proposes a light-driven ternary heterojunction transistor (T-HTR) exhibiting three distinct states within a single structure. This multi-state switching is achieved through the synergy of a vertical p-type/n-type heterojunction and a precisely controllable charge injection barrier at the source electrode. While this topic is of significant current interest, there are still some critical points need to be addressed:
1. The authors measured the UV-Vis absorption ranges of the individual semiconductors. However, the absorbance profile of the integrated p-n heterojunction may differ. Please discuss potential changes in the heterojunction's absorption characteristics and their implications. Furthermore, given the likelihood that light of different wavelengths differentially impacts device performance, could logic operations be performed under specific wavelength illumination?
2. Figure 2b shows clear negative differential transconductance (NDT), a behavior highly sensitive to interface traps. To better assess stability and trap influence, please proved the hysteresis information on the corresponding transfer curves and transconductance curves.
3. Please explain the observed shifts in threshold voltage (VTH) under varying light intensities in Figure 2a. What is the underlying mechanism driving this dependence?
4. The background section lacks an overview of recent progress in light-driven multi-valued logic circuits. Please discuss more recent related work.
5. The barrier layer lowers the on-current for all devices. As reduced current can limit operating frequency, which is a critical parameter for logic circuits, could the author comment on this potential trade-off and its implications?
6. The output signals in Figure 4d are current-based, and the state current levels in Figure 4f exhibit orders-of-magnitude differences. This complicates integration into real circuits. Could the author propose an alternative design for a fully transistor-based ternary logic gate utilizing voltage outputs to enhance practical applicability?

Reviewer #2

(Remarks to the Author)
Please kindly find the comments in the attachment.

Reviewer #3

(Remarks to the Author)
In this paper, the authors reported a light-driven ternary heterojunction transistor (T-HTR) enabled by incorporating a vertical p-type/n-type heterojunction structure together with a charge injection barrier at the source electrode of a conventional field-effect transistor. The T-HTR demonstrates three distinct states by tuning Vgs and the input light intensity, and relatively good stability. In addition, the authors also demonstrated ternary logic inverter (T-inverter), ternary OR and AND gates, and a prototype pixel-level sensory circuit. This work has some novelty in terms of transistor design, and might be interesting to readers in the research field of ternary computing. However, major revisions are needed before acceptance by Nature Communications.

1. The authors claim that the T-HTR has static power consumption as low as ~ 15 pW by multiplexing the lowest current of 3.8 pA by 4 V at V_G = 0 V. However, what's the distribution of the Vth? If Vth shifts, the static power consumption could be much larger. In addition, in a daytime environment without any encapsulation, the light intensity can be 100 mW/cm^2, corresponding to a much larger static power consumption. On the other hand, if encapsulation is adopted to block environment light, how to use light to achieve a ternary transistor operation considering practical applications?
2. The authors analyze the mechanism of T-HTR in Fig. 1g and Fig. 3c. However, I am confused why the CsF or MoO_x coating could change the work function of Au. For their UPS study, they measured the work-functions of film stacks. However, it cannot be directly related to the work-function of Au. In addition, the authors should analyze the working mechanism more with various V_{gs} and V_{ds} values using band diagrams, and with reversed source/drain orientation to better illustrate the design principles.
3. The T-HTR has very low operation current. For logic operations, clock speed is very important. The authors should give an estimation of how fast their logic circuits can work.
4. The design of T-HTR has a big problem for integration. How to make sure that the desired T-HTRs are illuminated in a circuit?
5. Minor: It is recommended that VD and gnd are added to Fig 1a and Fig. 3a to illustrate the test setup of T-HTR.

Version 1:

Reviewer comments:

Reviewer #1

(Remarks to the Author)
The authors report a light-driven ternary logic device, demonstrating in-sensor computing functionality with a particular emphasis on low static power consumption. Overall, the topic is timely and the general device concept is interesting. However, several important issues need to be clarified.
1. The authors state that there is a trade-off between power consumption and operating frequency, but some relevant data are inconsistent or not clearly presented. In the main text, when referring to Supplementary Table 1, the authors quote a static power of 3.2 pW/cm2, whereas Supplementary Table 1 uses 3.2 nW/cm2. Figures 2g and 2h compare devices without CsF and with 60 nm CsF, but the rise and fall times shown there appear inconsistent with the cutoff frequencies for 0 nm and 60 nm CsF in Supplementary Figure 14e. In addition, on page 10 the authors mention CsF thicknesses from 0 to 90 nm, but only provide experimental results up to 60 nm. Please also specify which CsF thickness is used for the devices in Figures 4 and 5, and which CsF thicknesses and cutoff frequencies correspond to the reported static power densities of 3.2 pW/cm2 and 3.2 nW/cm2.
2. Although Supplementary Figure 15 compares the effect of channel length, this alone is not sufficient to prove that the cutoff frequency is entirely determined by parasitic capacitance. It may also be limited by the intrinsic properties of the materials. Therefore, the estimate given after Supplementary Figure 14 that the device still has at least a 104-fold potential improvement in operating frequency appears somewhat optimistic. Moreover, more importantly, if the operating frequency is limited by 1/(2πRC), simple geometric scaling may not necessarily improve the RC-limited frequency: as the device area is reduced, the capacitance C decreases, but the resistance R increases proportionally.
3. Why do the authors attribute the conduction in the CsF layer to Poole–Frenkel conduction rather than Schottky emission?
4. On page 10 the text states "2.33 eV for C10-DNTT", whereas on page 19 it states "C13-PTCDI (2.33 eV)". One of these values appears to be incorrect.
5. The sentence "This improvement suggests that the CsF layer effectively suppresses the dominant trap-mediated relaxation pathways at the metal/semiconductor interface." is repeated twice (on pages 16 and 17).

Reviewer #2

(Remarks to the Author)
Though the authors have demonstrated significant improvements in this revised manuscript, the proposed operating mechanism is still not quite convincing.
(1) TCAD simulations are strongly recommended to show the evolution of band diagrams. The electron affinity and bandgap of each material can be used as inputs to generate the band alignment profiles for each bias condition.
(2) If the authors determined the interface trap density (Qit) from Cox (gate capacitance per unit area), why did they divide the result by the gate area again to obtain the surface charge density (σ)?

Reviewer #3

(Remarks to the Author)
In the revised manuscript, the authors have addressed my previous concerns. However, some issues need further clarification before acceptance for publication.
1. The authors treat the n- (C13-PTCDI) and p- type (C10-DNTT) semiconductors as dielectrics in their band diagram analysis. As a result, there is no band bending at interfaces (including both heterostructure and semiconductor-dielectric interfaces). Such analysis could be misleading considering depletion/accumulation regions that might have dimensions comparable to the thickness of the semiconductors. A more thorough analysis should be included. Additionally, how the

authors determine the bend diagram under light illumination is also lacking.
In the revised manuscript, some typos should also be corrected:
(1) Fig. 1g and Fig. 3c, “dark (dashed lines)” and “illumination (solid lines)” do not match the figures.
(2) Page 11: “the apparent work function of the Au/CsF contact (~3.8 eV) places its Fermi level above the lowest unoccupied molecular orbital (LUMO) level of C13-PTCDI (2.06 eV), whereas the much deeper highest occupied molecular orbital (HOMO) level of the C10-DNTT (4.92 eV) imposes a large barrier.” The LUMO of C13-PTCDI is not correct.
2. The authors claim that “Although the present proof-of-concept devices employ large shadow-mask-defined geometries, scaling the channel length and minimizing gate–electrode overlap through photolithography would reduce C_{GG} by ~10^4-fold, potentially extending the cut-off frequency into the hundreds-of-kHz range. Also, combined with high-mobility semiconductors such as oxides or 2D materials, the attainable speed could further approach the MHz regime.” However, the authors neither demonstrated the scaling of their devices or ternary transistors based on high-mobility oxides or 2D materials, making this claim vague. Without real demonstration, the concern of integration of their ternary devices with merits over binary computation is not fully addressed.

Version 2:

Reviewer comments:

Reviewer #1

(Remarks to the Author)
The authors have addressed most of my previous concerns, and the manuscript has been substantially improved, particularly through the addition of TCAD simulations and the clarification of the operating mechanism. The overall presentation is now significantly clearer and more self-consistent.

I only have two minor comments that, in my opinion, would further strengthen the manuscript.

1. The authors attribute the transport in the CsF layer mainly to Poole–Frenkel conduction based on the linear relationship between ln(J/E) and sqrt(E). While this interpretation is reasonable, similar behavior may also arise from Schottky emission or hopping-assisted transport over a limited electric-field range.

To further support the Poole–Frenkel assignment, the authors may consider providing additional evidence, such as temperature-dependent J–E measurements, comparison with Schottky-emission fitting, extraction of the apparent dielectric constant from the fitted slope, or thickness-/electrode-dependence analysis. In the absence of such evidence, I suggest that the authors slightly soften the wording regarding the dominant transport mechanism.

2.The authors have appropriately revised the previous discussion regarding frequency scaling and now present the work more carefully as a proof-of-concept demonstration. Nevertheless, the experimentally demonstrated cutoff frequency remains relatively low compared to conventional logic devices.

While the proposed architecture is interesting for ultralow-power sensing and in-sensor computing applications, I recommend that the authors further emphasize the current proof-of-concept nature of the platform and briefly discuss the remaining challenges toward practical implementation, such as operating speed, scalability, and large-scale integration compatibility.

Reviewer #2

(Remarks to the Author)
All my concerns have been properly addressed. Therefore, the manuscript is suitable for publication.

Reviewer #3

(Remarks to the Author)
The authors fully addressed my concern. No more revision needed.

Open Access This Peer Review File is licensed under a Creative Commons Attribution 4.0 International License, which permits use, sharing, adaptation, distribution and reproduction in any medium or format, as long as you give appropriate credit to the original author(s) and the source, provide a link to the Creative Commons license, and indicate if changes were made.
In cases where reviewers are anonymous, credit should be given to 'Anonymous Referee' and the source.
The images or other third party material in this Peer Review File are included in the article's Creative Commons license, unless indicated otherwise in a credit line to the material. If material is not included in the article's Creative Commons license and your intended use is not permitted by statutory regulation or exceeds the permitted use, you will need to obtain permission directly from the copyright holder.
To view a copy of this license, visit https://creativecommons.org/licenses/by/4.0/

Response to the Reviewers' Comments

The authors thank the Reviewers for their thorough review of our manuscript and the valuable comments. We have revised the manuscript and supporting information based on the Reviewers' comments. The Reviewers' comments appear in **black**, and the authors' responses are in **blue**. In the revised manuscript, the changes with respect to the previous version are highlighted.

Reviewer (#1)'s COMMENTS:

The author proposes a light-driven ternary heterojunction transistor (T-HTR) exhibiting three distinct states within a single structure. This multi-state switching is achieved through the synergy of a vertical p-type/n-type heterojunction and a precisely controllable charge injection barrier at the source electrode. While this topic is of significant current interest, there are still some critical points need to be addressed:

Q1) The authors measured the UV-Vis absorption ranges of the individual semiconductors. However, the absorbance profile of the integrated p-n heterojunction may differ. Please discuss potential changes in the heterojunction's absorption characteristics and their implications. Furthermore, given the likelihood that light of different wavelengths differentially impacts device performance, could logic operations be performed under specific wavelength illumination?

Response:

We sincerely appreciate the Reviewer's valuable comment regarding the possible difference between the absorption of the individual semiconductors and that of the integrated p-n heterojunction. In response to the comment, we compared the UV-vis spectra of the p-type and n-type single-layer films and the bilayer p-n heterojunction films (each semiconductor layer was 20 nm thick) (**Fig. R1**). In single layers, C13-PTCDI (n-type) shows an absorption edge near ~600 nm (optical bandgap of 2.06 eV), while C10-DNTT (p-type) exhibits an edge near ~480 nm (optical bandgap of 2.59 eV). As expected, the heterojunction presents a complementary, broadened absorption spectral coverage. Notably, the absorbance of the bilayer is lower than the simple sum of the two single layers due to the Fresnel reflections and interference at the air/semiconductor and semiconductor/semiconductor interfaces that redistribute the internal field and increase the reflectance[R1].

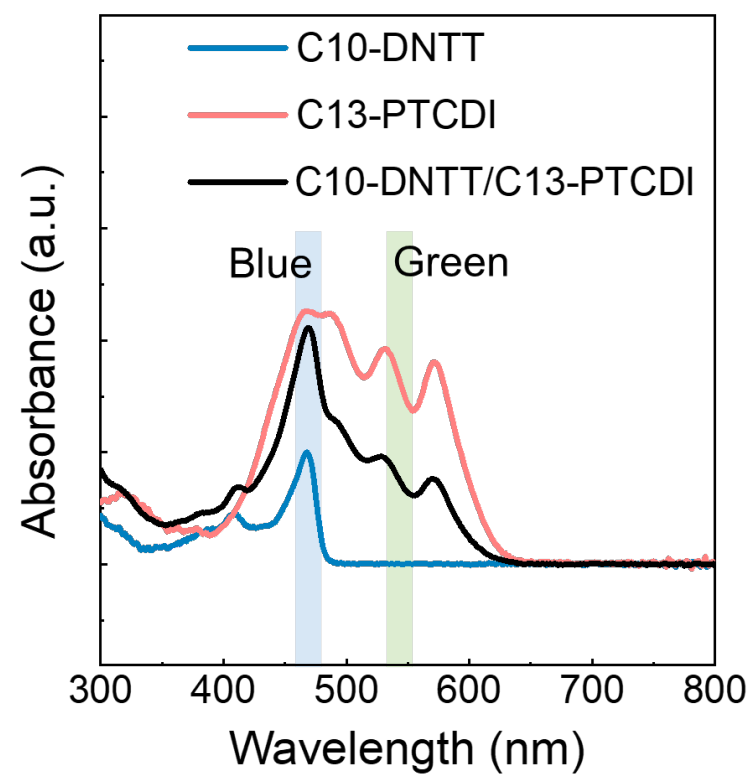

Figure R1. UV/Vis absorption spectra of the C13-PTCDI, C10-DNTT, and C13-PTCDI/C10-DNTT bilayer thin films.

These optical features translate into device characteristics (**Fig. R2a-d**). Under monochromatic illumination, double-sweep transfer curves showed that the photocurrent magnitude decreased in the order of 460 nm (blue), 535 nm (green), and 627 nm (red) illumination.

Furthermore, these observations indicate that Boolean logic can be realized only under specific wavelength combinations at identical light intensity (**Fig. R2e-f**). For instance, when both blue and green lights are simultaneously illuminated, the sum of their individual photocurrents exceeds the predefined threshold current (I_{th}), thereby yielding an output corresponding to the AND logic condition. In contrast, when blue and red illumination are applied simultaneously, the photocurrent generated by red illumination is too small; hence, the combined photocurrent remains below I_{th}, and the AND logic condition cannot be satisfied. In other words, the AND operation is achievable only under certain wavelength combinations where the cumulative photocurrent surpasses the threshold. These representative results suggest that, in general, specific wavelength and moderate intensity tuning can allow reliable Boolean logic operations.

We have revised the manuscript by adding the optical absorption characteristics of heterojunction and their direct connection to logic functionality in the proposed T-HTR.

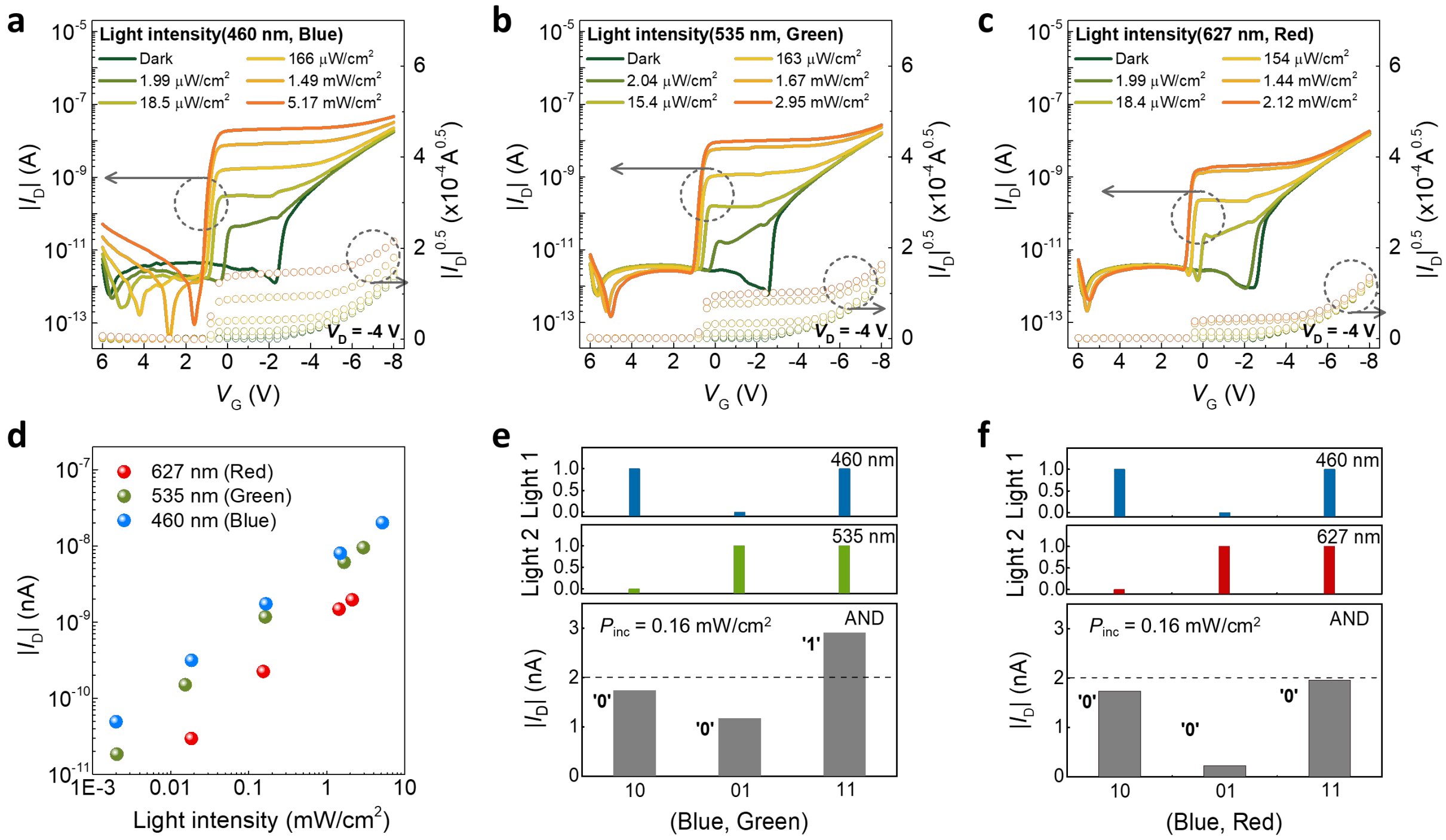

Figure R2. a. Transfer characteristics of the T-HTR under illumination at 460 nm (blue), **b.** 535 nm (green), and **c.** 627 nm (red). **d.** Extracted drain current (I_D) in the intermediate state under illumination at 460 nm, 535 nm, and 627 nm. **e**. Schematic illustration of the wavelength-selective logic operations of the T-HTR under 460 nm–535 nm and **f**. 460 nm–627 nm illumination at an incident light power density (P_{inc}) of 0.16 mW/cm^2.

[R1] *Journal of Applied Physics*, **86**(1), 487-496 (1999).

The revised parts are as follows;

(1) Revised the main text in the revised manuscript (p. 8).

Ultraviolet-visible (UV-vis) spectroscopy revealed that C10-DNTT exhibited an absorption peak around 460 nm (blue light), whereas C13-PTCDI showed absorption peaks not only near 460 nm but also around 535 nm (green light) (**Fig. 1e**). When the two materials were stacked to form a C13-PTCDI/C10-DNTT bilayer heterojunction, the absorption spectrum became broader and complementary, encompassing both regions while exhibiting a slightly lower overall absorbance than the simple sum of the individual layers due to Fresnel reflections and interference at the interfaces[40].

(2) Revised the main text in the revised manuscript (p. 14).

When the photocurrent was plotted as a function of light intensity, it exhibited a linear relationship in the intermediate state (**Fig. 2c**). Such a good linearity is attributed to the injection barrier layer, which suppresses efficiently the light-induced additional carrier injection from the source, thereby ensuring a linear proportion of photocurrent to the amount of the photogenerated carriers[53,54,55,56]. To examine the wavelength-dependent operation of the device, the T-HTR was illuminated with monochromatic light at 460, 535, and 627 nm. Distinct photocurrent responses were observed, consistent with the absorption characteristics of the C13-PTCDI/C10-DNTT heterojunction (**Supplementary Fig. 12**). This spectral dependence demonstrates the optical tunability of the T-HTR and suggests the feasibility of wavelength-selective logic operations.

(3) Replaced Fig. 1e with **Figure R1** in the revised manuscript (p. 31).

Figure 1 | Design of the ternary heterojunction transistor (T-HTR). a. The schematic illustration and **b**. optical microscopy image of the T-HTR (scale bar: 500 μm). **c**. The schematic illustration of transfer characteristics and **d**. operational mechanism of the T-HTR consisting of three switching states. **e**. Ultraviolet-visible (UV/Vis) absorption spectra of the n-type (C13-PTCDI), p-type (C10-DNTT) and n-type/p-type bilayer organic semiconductors. **f**. Ultraviolet photoelectron spectroscopy (UPS) spectra of Au contact with thermally deposited cesium fluoride (CsF) as a function of CsF thickness. **g**. Schematic energy band diagrams of the source-side stack in the dark (dashed lines) and under illumination (solid lines). The trapped charges at the CsF/p-type interface enhances the internal electric field in CsF.

(4) Added the **Figure R2** to Supplementary Information, together with the corresponding descriptions (p. 15).

Under monochromatic illumination, double-sweep transfer curves showed that the photocurrent magnitude decreased in the order of 460 nm (blue), 535 nm (green), and

627 nm (red) illumination (**Supplementary Fig. 12a-d**).

Furthermore, these observations indicate that Boolean logic can be realized only under specific wavelength combinations at identical light intensity (**Supplementary Fig. 12e, f**). For instance, when both blue and green lights are illuminated simultaneously, the sum of their individual photocurrents exceeds the predefined threshold current (I_{th}), thereby yielding an output corresponding to the AND logic condition. In contrast, when blue and red illumination are applied together, the photocurrent generated by red illumination is too small; hence, the combined photocurrent remains below I_{th}, and the AND logic condition cannot be satisfied. In other words, the AND operation is achievable only under certain wavelength combinations where the cumulative photocurrent surpasses the threshold. These representative results suggest that, in general, specific wavelength and moderate intensity tuning can allow reliable Boolean logic operations.

(5) Added the reference [40] in the revised manuscript.

Q2) Figure 2b shows clear negative differential transconductance (NDT), a behavior highly sensitive to interface traps. To better assess stability and trap influence, please proved the hysteresis information on the corresponding transfer curves and transconductance curves.

Response:

We appreciate the Reviewer's careful observation. First of all, we would like to clarify that the behavior shown in Fig. 2b does not correspond to negative differential transconductance (NDT). NDT is defined as the regime where the I_{D} decreases with increasing gate voltage (V_{G}), resulting in negative transconductance values (g_{m}<0). In our T-HTR, on the other hand, the g_{m} remains positive throughout the whole measurement range; what is observed in Fig. 2b is not a decrease of I_{D} with V_{G}, but rather the flattening of g_{m} to near zero, which signifies the suppression of field-effect current and the emergence of the V_{G}-independent intermediate state.

Nevertheless, we fully agree with the Reviewer that hysteresis analysis is an important indicator of interface trap states and operational stability. To address this point, we have

conducted additional double-sweep measurements of the transfer characteristics and transconductance (**Fig. R3**). The results show negligible hysteresis between the forward and reverse sweeps, confirming that the intermediate state and the suppressed g_{m} are not the artifacts of trap-induced instability but arise from the engineered injection barrier design.

We have revised the manuscript by including the hysteresis measurements and clarifying that the observed g_{m} flattening corresponds to a V_{G}-independent intermediate state rather than NDT.

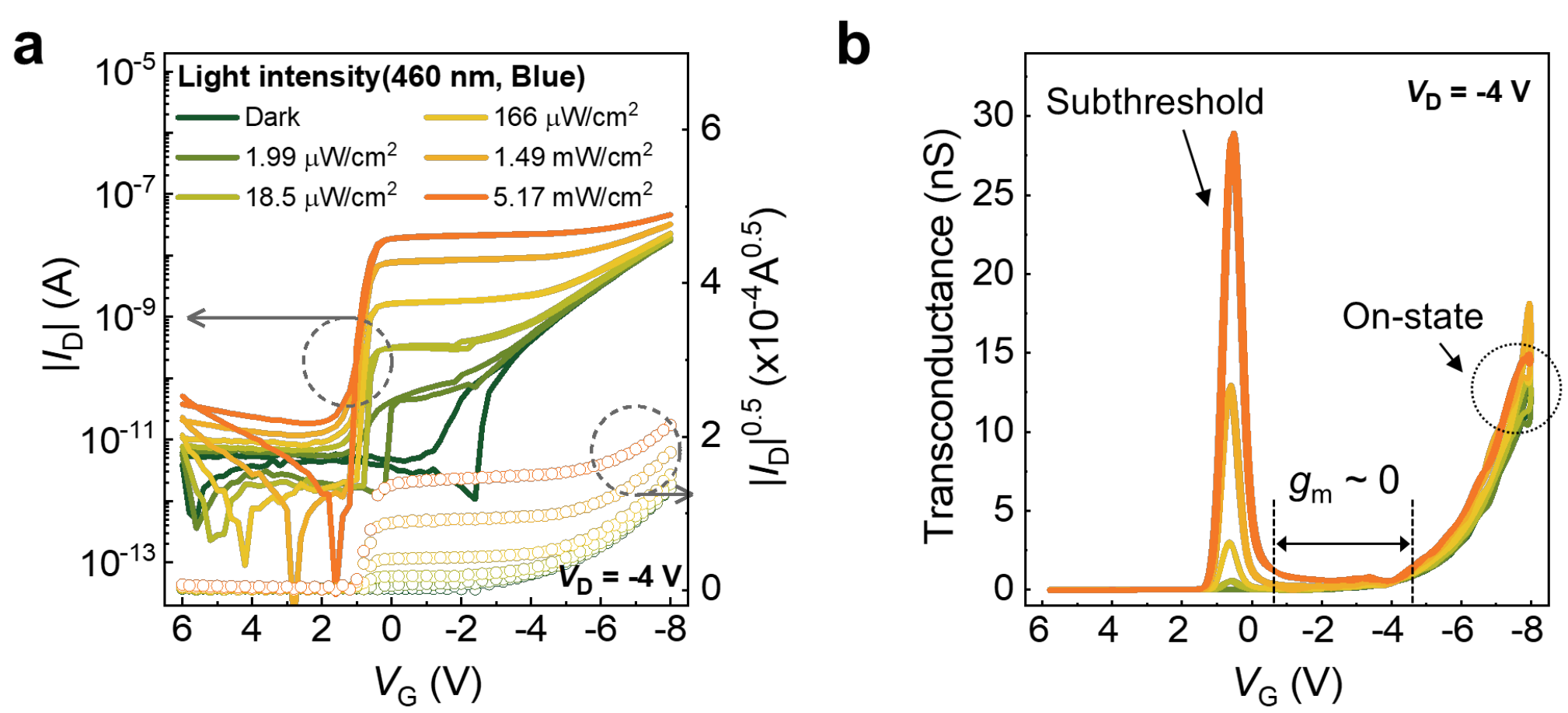

Figure R3. Electrical characterization of the T-HTR. **a**. The transfer characteristics and **b**. g_{m} of the T-HTR under 460 nm illumination as a function of intensity.

The revised parts are as follows;

(1) Revised the main text in the revised manuscript (p. 13).

The sharp transconductance peak in the subthreshold region corresponds to the transition from channel depletion to channel accumulation, while the subsequent near-zero flattening of the transconductance in the ZTC region indicates the suppression of field-effect current. It should be noted that this behavior does not correspond to a negative transconductance regime. While negative transconductance involves a decrease in I_{D} with increasing V_{G} ($g_{\mathrm{m}} < 0$), the T-HTR maintains positive g_{m} ($g_{\mathrm{m}} > 0$) that simply approaches zero, reflecting the suppression of field-effect current and the formation of a V_{G}-independent intermediate state. Also, double-sweep measurements of transfer and transconductance curves exhibited negligible hysteresis,

confirming that the near-zero-g_{m} region arises from the engineered injection barrier rather than trap-induced instability (**Supplementary Fig. 10**).

(2) Added the **Figure R3** to Supplementary Information (p. 14).

Q3) Please explain the observed shifts in threshold voltage (V_{TH}) under varying light intensities in Figure 2a. What is the underlying mechanism driving this dependence?

Response:

The T-HTR characterized in Fig. 2a in the manuscript employs a p-type semiconductor as the channel, with the source side configured as a vertical p-n heterojunction capped by a CsF injection barrier layer. Under illumination, excitons at the heterojunction interface dissociate: photogenerated minority carriers are driven toward the source and accumulate/trap beneath the blocking layer, while photogenerated majority carriers are transported through the channel toward the drain. The accumulated/trapped photogenerated electrons at the semiconductor/CsF interface induce a positive charge on the CsF side, forming an interfacial dipole moment. The accumulated interfacial charge and dipole create an internal photovoltage and band bending that modulate the contact (Refs. 39–42 in manuscript), which redistributes the series potential drop at a fixed V_{G}, increasing the fraction that falls across CsF layer:

$$\Delta V_{\mathrm{CsF}} \approx (C_{\mathrm{ox}}/C_{\mathrm{CsF}}) \times |\Delta V_{\mathrm{TH}}|$$

where C_{ox} and C_{CsF} denote the capacitances per unit area of the gate dielectric and CsF layer, respectively, and ΔV_{TH} represents the light-induced shift in threshold voltage arising from interfacial charge accumulation and dipole formation. Therefore, while the pV3D3 energy band correspondingly flattens, the conduction band on the organic/CsF side shifts by

$$\Delta E_{\mathrm{c}} \approx q\Delta V_{\mathrm{CsF}} \approx q\left(\frac{C_{\mathrm{ox}}}{C_{\mathrm{CsF}}}\right)|\Delta V_{\mathrm{TH}}|.$$

Consequently, the gate bias required for channel formation decreases, and accordingly V_{TH} shifts to negative direction in the T-HTR:

$$|\Delta V_{\mathrm{TH}}| \propto \frac{\Delta Q_{\mathrm{dipole}} + \Delta Q_{\mathrm{photo}}}{C_{\mathrm{ox}}}$$

where $\Delta Q_{\mathrm{dipole}}$ represents the charge separation-induced interfacial dipole moment and $\Delta Q_{\mathrm{photo}}$ denotes the accumulated/trapped photogenerated charge.

As the light intensity increases, the numerator part ($\Delta Q_{\mathrm{dipole}} + \Delta Q_{\mathrm{photo}}$) increases, leading to a gradual decrease in V_{TH}. When trap sites beneath the barrier become saturated, the internal photovoltage no longer increases and V_{TH} saturates, converging to the value of the control device without the blocking layer. This indicates that the barrier layer raises the initial V_{TH} in the dark by suppressing minority carrier injection from the source, while strong illumination compensates this barrier via light-induced contact modulation (**Fig. R4**).

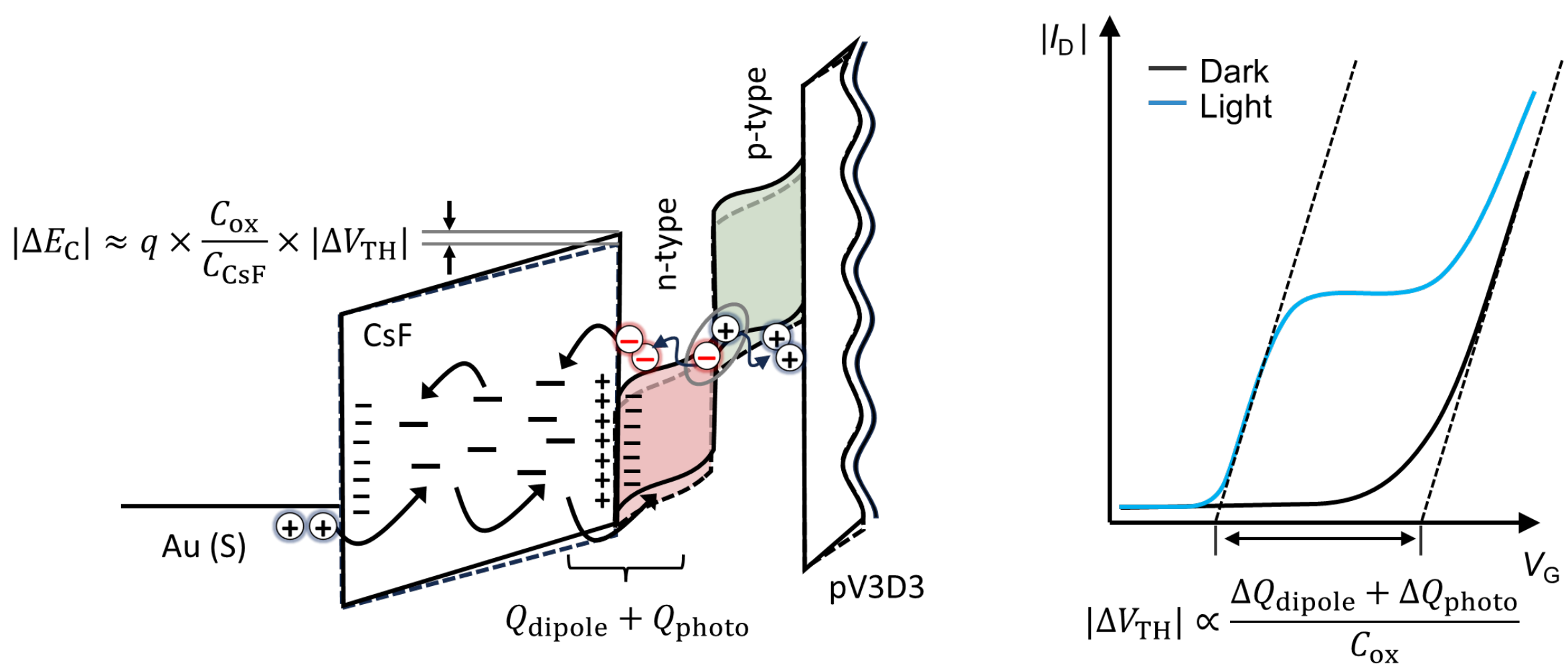

Figure R4. Schematic illustration of the illumination-induced V_{TH} shift in the T-HTR.

We have revised the manuscript by incorporating this detailed explanation of the illumination-induced ΔV_{TH} to highlight the role of interfacial photovoltage and dipole effects.

The revised parts are as follows;

(1) Revised the main text in the revised manuscript (p. 12).

The transfer characteristics of the T-HTR were investigated (**Fig. 2a**). Under dark

condition, the T-HTR exhibited typical on and off switching behavior. On the other hand, upon 460 nm light illumination, a constant photocurrent was observed between the on and off-states, defining the intermediate state. As the light intensity increased, the V_{TH} decreased gradually, which is attributed to the internal photovoltage generated by interfacial dipoles and the accumulation of photogenerated minority carriers beneath the injection-barrier layer[49,50,51,52]. These trapped charges locally bend the energy bands, effectively lowering the gate bias required for channel formation (**Supplementary Fig. 8**).

(2) Added the **Figure R4** to Supplementary Information together with the corresponding descriptions (p. 11).

Under illumination, excitons at the heterojunction interface dissociate: photogenerated minority carriers are driven toward the source and accumulate/trap beneath the blocking layer, while photogenerated majority carriers are transported through the channel toward the drain. In the p-type T-HTR, the accumulated/trapped photogenerated electrons at the semiconductor/CsF interface induce a positive charge on the CsF side, forming an interfacial dipole moment. The accumulated interfacial charge and dipole create an internal photovoltage and band bending that modulate the contact, which redistributes the series potential drop at a fixed V_{G}, increasing the fraction that drops across CsF:

$$\Delta V_{\mathrm{CsF}} \approx (C_{\mathrm{ox}}/C_{\mathrm{CsF}}) \times |\Delta V_{\mathrm{TH}}|$$

where C_{ox} and C_{CsF} denote the capacitances per unit area of the gate dielectric and CsF layer, respectively, and ΔV_{TH} represents the light-induced shift in threshold voltage arising from interfacial charge accumulation and dipole formation. Therefore, conduction band on the organic/CsF side shifts by

$$\Delta E_{\mathrm{c}} \approx q\Delta V_{\mathrm{CsF}} \approx q\left(\frac{C_{\mathrm{ox}}}{C_{\mathrm{CsF}}}\right)|\Delta V_{\mathrm{TH}}|,$$

while the pV3D3 energy band correspondingly flattens. Consequently, the gate bias required for channel formation decreases and ΔV_{TH} accordingly to negative direction in p-type T-HTR (and positively for n-type T-HTRs):

$$|\Delta V_{\mathrm{TH}}| \propto \frac{\Delta Q_{\mathrm{dipole}} + \Delta Q_{\mathrm{photo}}}{C_{\mathrm{ox}}}$$

where $\Delta Q_{\mathrm{dipole}}$ represents the charge separation-induced interfacial dipole moment and $\Delta Q_{\mathrm{photo}}$ denotes the accumulated/trapped photogenerated charge.

As the light intensity increases, the numerator ($\Delta Q_{\mathrm{dipole}} + \Delta Q_{\mathrm{photo}}$) increases, leading to a gradual decrease in V_{TH}. When trap sites beneath the barrier become saturated, the internal photovoltage no longer increases and V_{TH} saturates, converging to the value of the control device without the blocking layer. This indicates that the barrier layer raises the initial V_{TH} in the dark by suppressing minority carrier injection from the source, while strong illumination compensates this barrier via light-induced contact modulation.

Q4) The background section lacks an overview of recent progress in light-driven multi-valued logic circuits. Please discuss more recent related work.

Response:

We appreciate the Reviewer's comment and agree that a more comprehensive overview of recent progress in light-driven multi-valued logic (MVL) devices is important to contextualize our work.

Recent advances have demonstrated MVL operations by exploiting anti-ambipolar transistors (AATs) with optically tunable NDT characteristics. For example, Cui *et al*. reported configurable anti-ambipolar photoresponses in InSe transistors, where trap-assisted processes enabled optically switchable ternary logic operations[R2]. Similarly, Panigrahi *et al*. achieved high-performance organic MVL circuits using optically tunable AATs based on C8-BTBT/PhC2-BQQDI

heterojunctions, attaining improved voltage transfer characteristics and enhanced static noise margins under UV illumination[R3]. Additional strategies, such as combining geometry optimization with UV irradiation, have been used to stabilize intermediate logic levels in organic ternary inverters[R4]. Furthermore, Shin *et al.* demonstrated photonic modulation of NDT in heterojunction transistors, enabling binary–ternary logic conversion under optical inputs[R5].

These studies clearly indicate that optical signals can be exploited as an important additional control knob for MVL operation, enhancing functionality and integration density. However, they also pose intrinsic limitations. In particular, these devices rely on NDT as the core mechanism, which inherently introduces instability in the logic operation due to their inherent sensitivity to traps and bias conditions. Moreover, the optical modulation often leads to only a limited dynamic range of output variation, rendering it challenging to achieve clear and reliable separation between multiple logic states. Consequently, although these works successfully demonstrate light responsiveness and multi-state behavior, the practical effectiveness of the logic operation still remains limited.

In contrast, our approach is based on establishing a zero-transconductance intermediate state stabilized by an engineered injection barrier, which avoids the intrinsic instability associated with the NDT-driven operation. This design not only eliminates the susceptibility to trap-induced fluctuations but also provides an unprecedentedly wide and well-defined dynamic range for the intermediate state, ensuring clear separation between logic levels under optical input.

In the revised manuscript, we have included a discussion of several representative works to better contextualize these distinctions.

[R2] *Applied Physics Letters*, **125**(5) 051105 (2024).
[R3] *Journal of Materials Chemistry C*, **10**(14), 5559-5566 (2022).
[R4] *Advanced Materials Technologies*, **8**(22), 2301049 (2023).
[R5] *Small*, **21**(30), 2501543 (2025).

The revised parts are as follows;

(1) Revised the main text in the revised manuscript (p. 5).

The realization of optoelectronic multi-valued logic circuits requires the development of transistors capable of exhibiting three distinct switching states. Such transistors have been demonstrated using heterojunction transistors with various channel materials, including organic semiconductors[16,17,18], graphene[19,20,21], metal oxides[22,23,24], and two-dimensional (2D) transition metal dichalcogenides (TMDs)[25,26,27]. A key characteristic of heterojunction transistors is the presence of negative transconductance, where the drain current (I_{D}) peaks and subsequently decreases within a specific gate voltage (V_{G}) range, thereby constructing an intermediate state[28]. Several light-driven multi-valued logic devices have extended this concept by exploiting optically tunable negative-transconductance behavior in heterojunction transistors. For example, InSe-based transistors have exhibited configurable photo-responses enabling ternary logic operation[29], where trap-assisted processes enabled negative transconductance and optically switchable multi-level states. Similarly, optically reconfigurable ternary logic operation has been demonstrated in C8-BTBT/PhC2-BQQDI heterojunction transistors, exhibiting improved voltage-transfer characteristics and enhanced static-noise margins under UV illumination[30,31]. Additional efforts have focused on photonic modulation of negative transconductance in In_2O_3/PDPP3T heterojunction transistors, enabling binary-to-ternary logic conversion under monochromatic optical inputs[32]. While these studies successfully demonstrated that light can serve as an additional control knob for multi-state logic, the negative-transconductance mechanism is inherently V_{G}-dependent, and therefore the heterojunction transistor cannot establish a stable logic state by itself. This necessitates the use of an additional complementary transistor to construct a complementary inverter, imposing a critical design constraint. Moreover, under illumination, the coexistence of photocurrent and gate-induced field-effect current further aggravates the instability of the intermediate state. This overlap also limits the dynamic range of the photocurrent, reducing its precision in optical-state encoding and thus resulting in significant leakage current in the intermediate regime that may ultimately result in high static power consumption[30,31,32,33]. Overcoming these limitations requires a transistor that intrinsically exhibits a V_{G}-independent intermediate state, whose level is finely tunable by light. To date, however, no such device has been

experimentally demonstrated.

(2) Added the reference [29-32] in the revised manuscript.

Q5) The barrier layer lowers the on-current for all devices. As reduced current can limit operating frequency, which is a critical parameter for logic circuits, could the author comment on this potential trade-off and its implications?

Response:

We thank the Reviewer for raising this important point. We fully agree that the insertion of the barrier layer inevitably lowers the on-current of the devices, and the reduced current may, in principle, limit the attainable operating frequency. To analyze this trade-off, we examined transfer characteristics systematically as a function of CsF thickness and evaluated the relationship between switching speed and power consumption. In the on-state, the unity-gain cut-off frequency[R6] is

$$f_{\mathrm{T}} = \frac{g_m}{2\pi C_{\mathrm{gg}}}$$

where g_{m} is the transconductance and C_{GG} is the total gate capacitance. g_{m} was obtained from transfer characteristics of the T-HTR (**Fig. R7a, b**). C_{GG} was estimated from the measured gate dielectric unit-area capacitance (C_{i}) (**Fig. R5**) multiplied by the summation of channel area ($W \times L$) and the gate–source/drain overlap area ($W \times [L_{\mathrm{ov,S}} + L_{\mathrm{ov,D}}]$) of the T-HTR (**Fig. R6**), which usually gives a capacitance far larger than the effective value in operation, and thus provides a conservative (lower-bound) estimate of the cut-off frequency. On the other hand, power consumption was calculated from the product of the driving voltage and the current density. Through this analysis, we confirmed a clear trade-off between switching speed and power consumption in our devices. As the CsF barrier thickness increases, the on-current decreases, leading to a reduction in the cut-off frequency from the 10^2–10^3 Hz range to below 1 Hz. At the same time, the static power consumption drops from the mW cm^{-2} scale to the sub-μW cm^{-2} scale (**Fig. R7c**). This clearly demonstrates that barrier engineering enables ultralow power operation, albeit at the expense of switching speed.

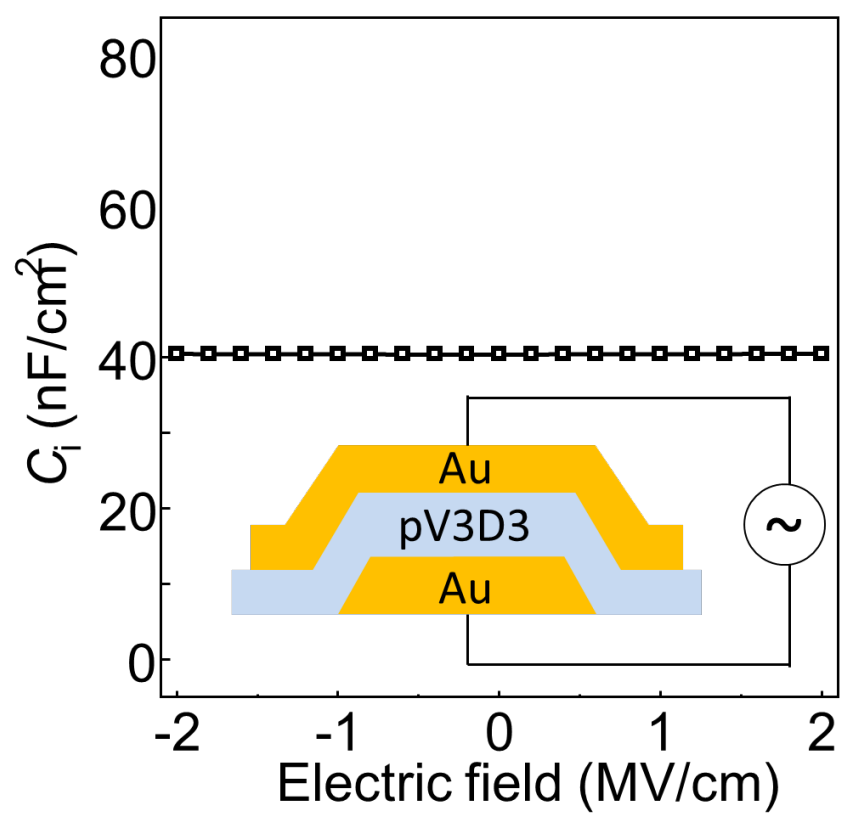

Figure R5. Capacitance per unit area (C_i)–electric field (E_i) of Au/poly(1,3,5-trivinyl-1,3,5-trimethyl cyclotrisiloxane) (pV3D3, 50 nm)/Au device.

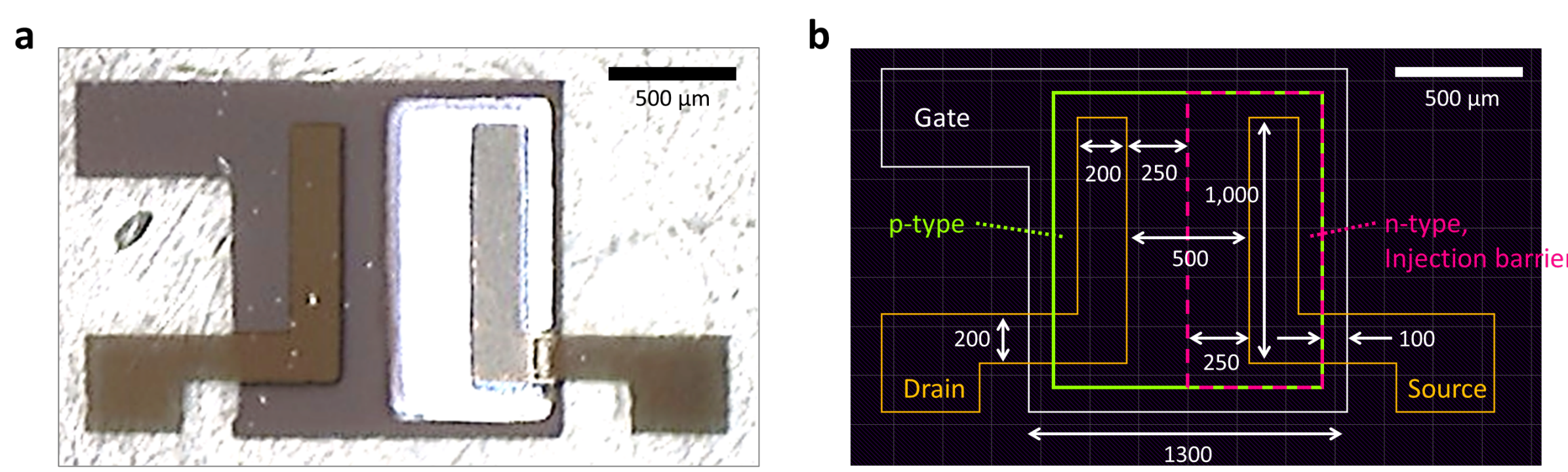

Figure R6. Dimensions of the T-HTR. **a**. An optical microscopy image (scale bar: 500 μm) and **b**. Shadow mask layout of the T-HTR (scale bar: 500 μm).

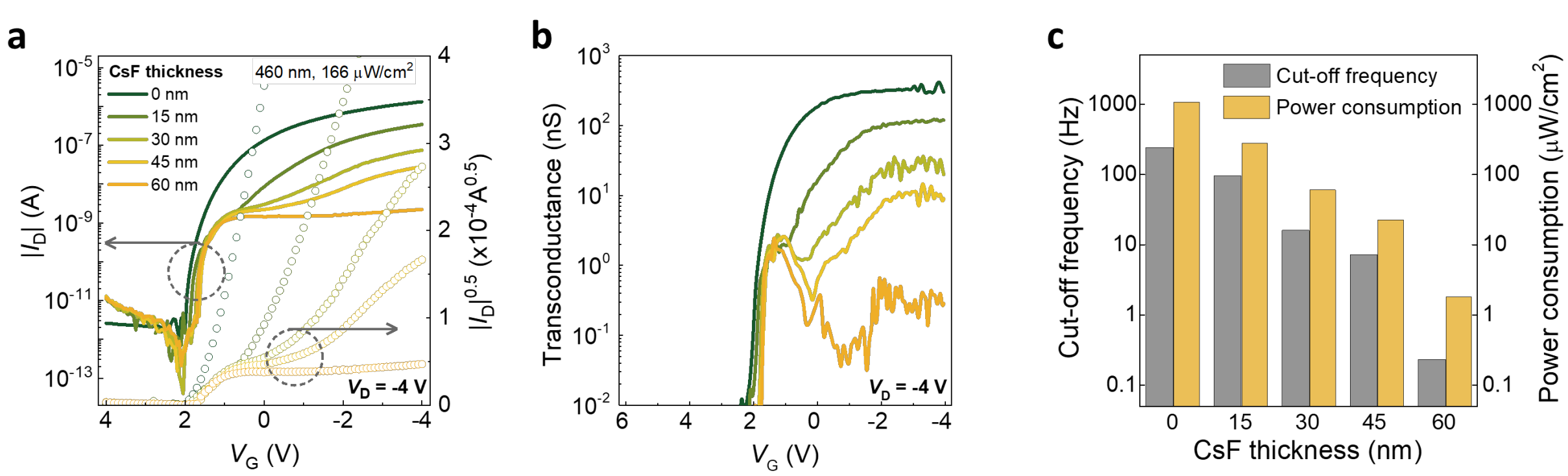

Figure R7. a. The transfer characteristics, **b**. transconductance (g_m), and **c**. cut-off frequency versus static power consumption of the T-HTR as a function of CsF barrier thickness. Increasing the CsF thickness reduces both the cut-off frequency and power consumption, illustrating the

speed-power trade-off governed by injection barrier engineering.

Rather than a drawback, this trade-off is highly relevant for application domains where ultralow static power is prioritized over high clock speed. In many always-on sensors, wearable biomedical monitors, and distributed environmental sensor nodes, the required operating frequencies typically lie only in the Hz–kHz regime, while the overriding design constraint is to minimize static power into the μW or even nW–pW range for long-term autonomous operation [R7], [R8], [R9], [R10]. This consensus has been consistently highlighted in the literature across wearable systems, energy-harvesting wireless sensor networks, and many other always-on sensing platforms[R11], [R12], [R13], [R14]. Moreover, our approach is particularly well suited for low-power applications, where minimizing both energy consumption and operation steps is critical, as the use of ternary logic reduces the number of computation steps to approximately log2/log3 ≈ 63.1 % compared to binary logic[R15]. This highlights that barrier engineering in T-HTRs provides a practical pathway toward energy-efficient, multi-valued logic systems.

Also, we emphasize that the present device was fabricated from thermally evaporated organic semiconductors patterned with shadow masks. This simple process is highly suitable for proof-of-concept demonstrations of heterojunction transistors, but it inevitably yields devices with relatively large dimensions, with critical lengths on the order of several hundred micrometers. By employing high-resolution patterning techniques such as standard photolithography, which allows scaling down to a few micrometers, the gate-to-source/drain overlap can be reduced drastically, thereby minimizing parasitic capacitances. In such a scenario, C_{GG} would decrease by approximately four orders of magnitude, leading to a corresponding ~10^4–fold improvement in the cut-off frequency, potentially reaching the hundreds of kHz range. Notably, this improvement arises purely from the reduction of parasitic capacitances, which does not directly affect energy consumption–although a lower parasitic capacitance can further reduce the overall switching energy. Moreover, the approach demonstrated in this work is mainly based on the band engineering through injection barrier design, and is therefore not limited to a particular semiconductor class. This implies that, with high-mobility semiconductors such as oxides or two-dimensional materials, the attainable operation speed could further be enhanced into the MHz regime.

In the revised manuscript, we have included this discussion to clarify the implications

of the current–frequency trade-off and to contextualize the relevance of our device concept for low-power applications.

[R6] *Superlattices and Microstructures*, **90**, 191-197 (2016).
[R7] *IEEE journal of biomedical and health informatics*, **20**(5), 1251-1264 (2016).
[R8] *Sensors*, **21**(2), 548 (2021).
[R9] Nano-Micro Letters, **16**(1), 52 (2024).
[R10] *IEEE Sensors Journal*, **23**(15), 16467-16480 (2023).
[R11] *Advanced Functional Materials*, **27**(15), 1605271 (2017).
[R12] The innovation, **4**(5), 100485 (2023).
[R13] *IEEE Sensors Journal*, **19**(20), 9047-9062 (2019).
[R14] Energy & Environmental Science, **14**(4), 2114-2157 (2021).
[R15] *IEEE Transactions on computers*, **100**, 1160-1179 (1984).

The revised parts are as follows;

(1) Revised the main text in the revised manuscript (p. 9).

A poly(1,3,5-trimethyl-1,3,5-trivinyl cyclotrisiloxane) (pV3D3, 50 nm) layer deposited in vapor-phase via initiated chemical vapor deposition (iCVD) was utilized as the gate dielectric layer[41]. The dielectric constant of the pV3D3 film was determined to be approximately 2.2, from which the dielectric thickness in the T-HTR was determined to be 50 nm (**Supplementary Fig. 2**).

(2) Revised the main text in the revised manuscript (p. 15).

The influence of the injection barrier thickness on the intermediate state was investigated (**Fig. 2e**). As the CsF thickness increased from 15 nm to 60 nm, the transition into the intermediate state became more distinct. The range of the intermediate state – defined as the V_G window between the initial V_G, where transconductance started to decrease from its peak, and the end V_G, where transconductance increased back to a level comparable to the initial peak value – broadened from less than 1 V for 15 nm-thick CsF to more than 4 V for 60 nm-thick CsF (**Fig. 2f**). Such expansion resulted from the progressively restricted effective charge

injection from the source with increasing barrier thickness, which shifted the onset voltage of field-effect current to higher V_G.

To further elucidate how the injection barrier thickness affects the device figure of merit, we quantitatively analyzed its influence on the operating speed and power consumption (**Supplementary Fig. 14**). As the CsF thickness increased, the on-current gradually decreases due to the restricted charge injection from the source. The reduction in on-current lowers the transconductance and consequently the unity-gain cut-off frequency ($f_T = g_m/2\pi C_{gg}$, where g_m is the transconductance and C_{gg} is the total gate capacitance)[57], while the static power consumption ($P = I \times V$, where I and V denote the operating current and driving voltage, respectively) decreases proportionally, resulting in a clear trade-off between operating speed and power efficiency. As the CsF thickness increased from 15 nm to 60 nm, the estimated cut-off frequency decreased from 10^2-10^3 Hz to below 1 Hz, whereas the static power density dropped from the mW cm^{-2} scale to the sub-μW cm^{-2} scale.

(3) Added the **Figure R5** to Supplementary Information (p. 3).

(4) Added **Figure R6** and **Figure R7** to the Supplementary Information, together with the corresponding descriptions (p. 19).

As the CsF thickness increased from 15 nm to 60 nm, the estimated f_T decreased from 10^2-10^3 Hz to below 1 Hz, whereas the static power density dropped from the mW cm^{-2} scale to the sub-μW cm^{-2} scale. Such a speed–power compromise is highly relevant for application domains where ultralow static power is prioritized over high operating frequency, including always-on optical sensors, wearable biomedical monitors, and distributed environmental sensor nodes typically operating in the Hz–kHz regime[1~8]. Furthermore, the ternary-logic framework intrinsically reduces the number of computational steps by ≈ 63 % ($\log_2$ / $\log_3$)[9], offering additional energy savings.

Although the present proof-of-concept devices employ large shadow-mask-defined geometries, scaling the channel length and minimizing gate–electrode overlap through photolithography would reduce total gate capacitance (C_{gg}) by ~10^4-fold, potentially extending the cut-off frequency into the hundreds-of-kHz range. Also, combined with high-mobility semiconductors such as oxides or 2D materials, the attainable speed could further approach the MHz regime.

(5) Added the reference [1-9] in the Supplementary Information.

Q6) The output signals in Figure 4d are current-based, and the state current levels in Figure 4f exhibit orders-of-magnitude differences. This complicates integration into real circuits. Could the author propose an alternative design for a fully transistor-based ternary logic gate utilizing voltage outputs to enhance practical applicability?

Response:

Thank you for the reviewer's insightful comment. We agree that the current levels in Fig. 4f differ by orders of magnitude, which may not be optimal for direct implementation in current-based logic circuits. It is noteworthy that our manuscript demonstrates not only the realization of p-type T-HTRs but also the successful implementation of n-type counterparts operating on the same working principle. This highlights the potential for constructing fully transistorized, voltage-output ternary logic gates based on a standard CMOS inverter topology (**Fig. R8**).

As illustrated in **Fig. R8**, both the p-type and n-type T-HTRs exhibit photo-induced intermediate states, which can be tuned such that their output currents are balanced to yield an intermediate logic level of approximately half the driving voltage (1/2 V_{DD}). In this complementary configuration, the p-type T-HTR acts as the pull-up element in its on-state, whereas the n-type T-HTR functions as the pull-down element, together realizing a standard ternary inverter topology. Moreover, the ability to optically toggle the intermediate states of each transistor provides an additional degree of freedom for logic design. When only one of the transistors is illuminated, the circuit transitions from the standard ternary inverter operation to either a positive or negative ternary inverter mode. In contrast, when both devices remain

unilluminated, the circuit behaves as a conventional binary logic inverter. This conceptual demonstration indicates that, similar to the current-based configuration, a variety of logic operations can also be achieved in the voltage-based scheme through optical control.

We have revised the Supplementary Information to include schematics and implementation details of the proposed transistor-based ternary logic gate configurations with voltage outputs.

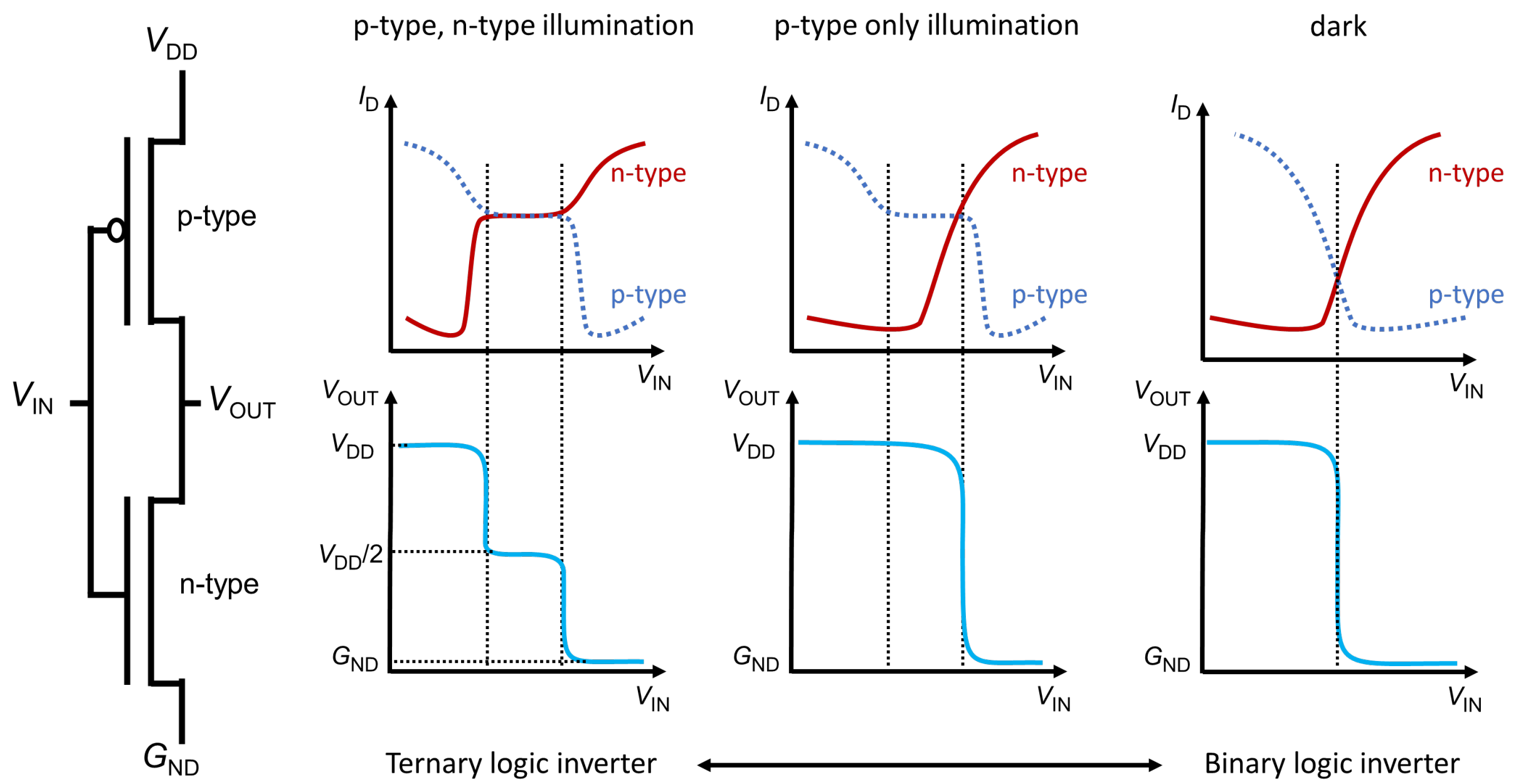

Figure R8. Conceptual schematic of voltage-output ternary logic gate configurations using complementary p-type and n-type T-HTRs under optical control.

(1) Revised the main text in the revised manuscript (p. 23).

Notably, when light was applied to only one of the T-HTRs, the output pattern of the ternary logic gates changed (marked by an arrow), even under the same voltage pulses and light intensity. This dynamic response to selective (or patterned) light illumination highlights the versatility in the logic encoding of optoelectronic ternary circuits. Although the present demonstration adopts a current-based logic configuration, a voltage-based logic gate can also be implemented using T-HTRs by pairing p-type and n-type devices in a complementary manner, which is conceptually demonstrated in **Supplementary Fig. 22**.

(2) Added **Figure R8** to the Supplementary Information, together with the corresponding descriptions (p. 28).

It is noteworthy that our manuscript demonstrates not only the realization of p-type T-HTRs but also the successful implementation of n-type counterparts operating on the same working principle. This highlights the potential for constructing fully transistorized, voltage-output ternary logic gates based on a standard CMOS inverter topology.

For instance, both the p-type and n-type T-HTRs exhibit photo-induced intermediate states, which can be tuned such that their output currents are balanced to yield an intermediate logic level of approximately half the driving voltage (1/2 V_{DD}). In this complementary configuration, the p-type T-HTR acts as the pull-up element in its on-state, whereas the n-type T-HTR functions as the pull-down element, together realizing a standard ternary inverter topology. Moreover, the ability to optically toggle the intermediate states of each transistor provides an additional degree of freedom for logic design. When only one of the transistors is illuminated, the circuit transitions from the standard ternary inverter operation to either a positive or negative ternary inverter mode. In contrast, when both devices remain unilluminated, the circuit behaves as a conventional binary logic inverter. This conceptual demonstration indicates that, similar to the current-based configuration, various logic operations can also be achieved in the voltage-based scheme through optical control.

Reviewer (#2)'s COMMENTS:

The manuscript proposed a novel light-driven ternary heterojunction transistor (T-HTR) design, demonstrating three distinct states and serving as key enablers for ternary logic gates. The presence of the PN junction at the source induces a built-in electric field which facilitates the separation of phonon-generated carriers. In addition, its potential in implementing in-sensor computing applications has also been explored. While some results appear interesting, this work still suffer from several critical shortcomings: inadequate physical mechanism analysis and device characterization etc. Before this manuscript can be considered for publication in *Nature Communications*, substantial revisions are required to meet the journal's rigorous standards.

Q1) Several essential device geometry and material property related parameters are missing in this manuscript: the relative dielectric constant of gate oxide layer, the bandgap and electron affinity of the p-type/n-type semiconductor, the mobility of the conducting channel, the length of the gate/source/drain electrodes etc. Moreover, more rigorous band alignments for the heterojunction and the source side that determines carrier injections should be given to present a more convincing physical picture about the operating mechanism. TCAD simulations will be favored.

Response:

We thank the Reviewer for pointing out the important issues. We agree that key device geometry and material parameters should be provided explicitly for completeness. In the revised manuscript, we have added the following details and corresponding values:

1) Relative dielectric constant of the gate dielectric layer, poly(1,3,5-trivinyl-1,3,5-trimethyl cyclotrisiloxane, pV3D3): This was extracted using the parallel plate capacitor model,

$$C_i = \varepsilon_0 \frac{kA}{d}$$

where, C_{i} is the measured capacitance per unit area, ε_0 the vacuum permittivity, k the dielectric constant, and d the thickness of the dielectric. The obtained k value was ~ 2.2, which was consistent with the previous reports[R16] (**Fig. R9**).

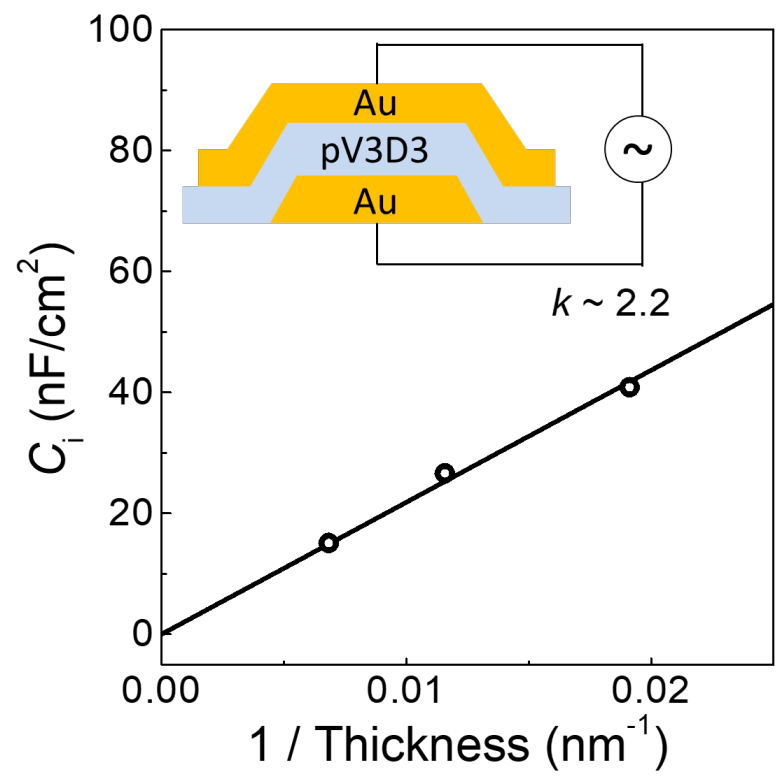

Figure R9. Capacitance per unit area (C_i) – inverse dielectric thickness ($1/d$) of Au/ pV3D3/Au device.

2) Bandgap and electron affinity of the semiconductors: The optical bandgap was determined from UV–vis absorption spectra using Tauc plots (**Fig. R10**), yielding values of 2.59 eV for C10-DNTT and 2.06 eV for C13-PTCDI (**Table R1**). The electron affinity was extracted from the ionization energy and bandgap. From ultraviolet photoelectron spectroscopy (UPS) analysis, the ionization energies of C10-DNTT and C13-PTCDI were 4.92 eV and 6.42 eV, respectively (**Fig. R11**). By combining these values with the optical bandgap, electron affinities of 2.33 eV for C10-DNTT and 4.36 eV for C13-PTCDI were obtained (**Table R1**).

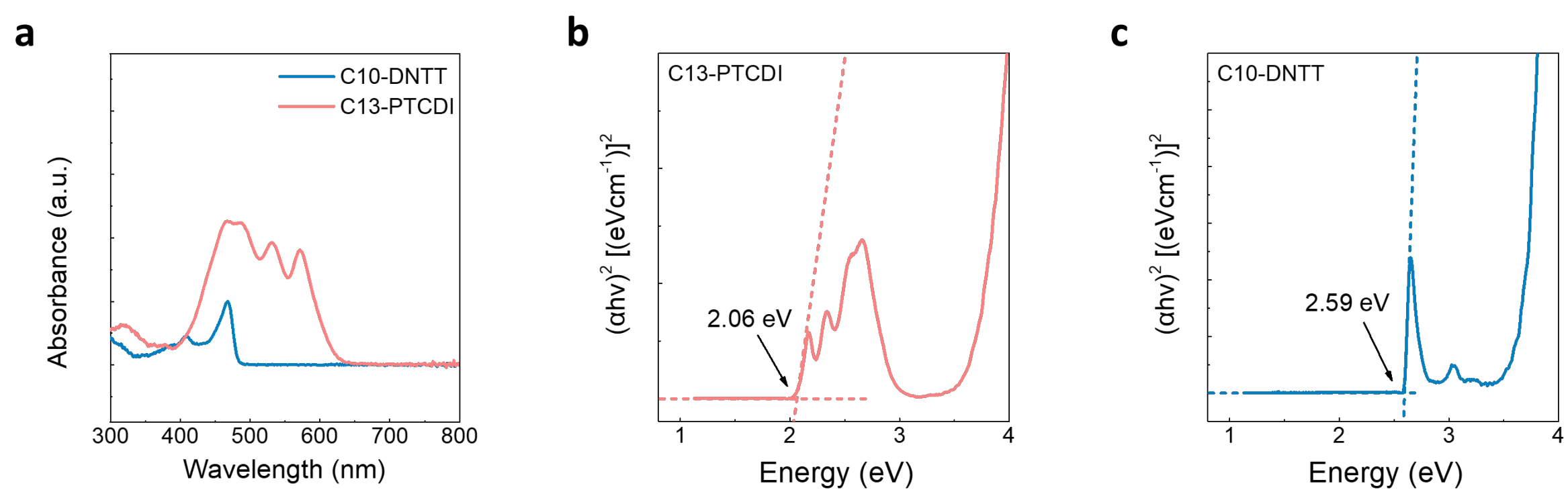

Figure R10. a. UV–vis absorption spectra and **b**. corresponding Tauc plots of C10-DNTT and C13-PTCDI thin films, used to extract the optical bandgap values.

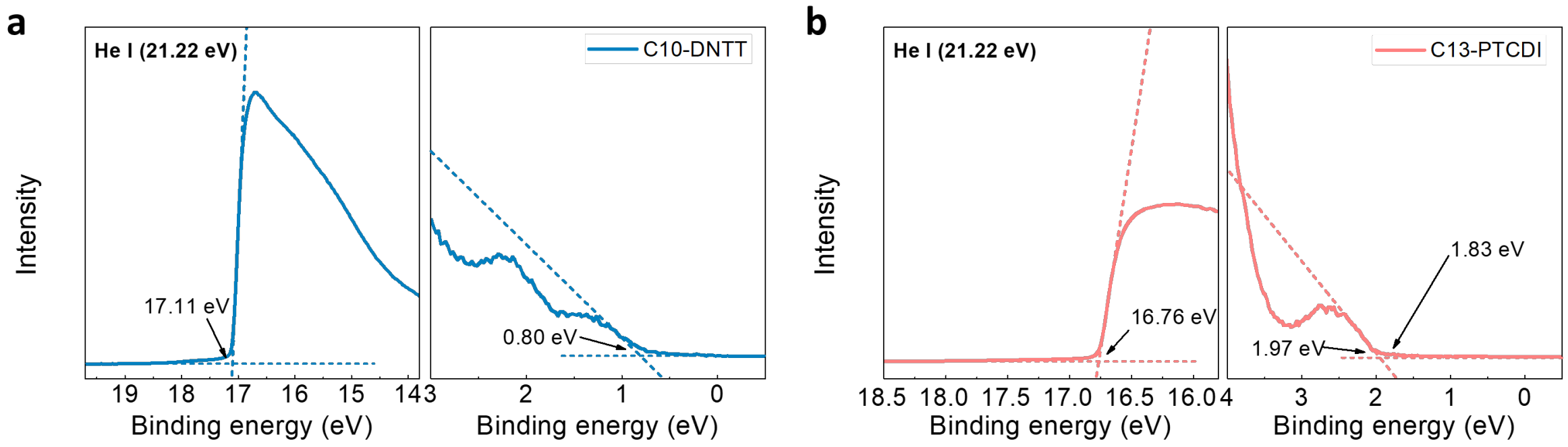

Figure R11. a. UPS spectra of C10-DNTT and **b**. C13-PTCDI thin films.

Table R1. Summary of energy levels of C10-DNTT and C13-PTCDI.

Materials	Optical bandgap (eV)	Work function (eV)	HOMO (eV)	LUMO (eV)
C10-DNTT	2.59	4.12	4.92	2.33
C13-PTCDI	2.06	4.45	6.42	4.36

3) Mobility of the conducting channels: To determine the hole and electron mobilities, p-type (C10-DNTT) and n-type (C13-PTCDI) thin-film transistors were fabricated, respectively. The field-effect mobility (μ) was extracted from the transfer characteristics using the following equation,

$$I_d = \frac{1}{2}\mu C_i \frac{W}{L}(V_{\mathrm{G}} - V_{\mathrm{TH}})^2$$

where, C_{i} is the measured capacitance per unit area, W the channel width, L the channel length, V_{G} the gate voltage, V_{TH} the threshold voltage of the dielectric transistor. The hole mobility of C10-DNTT was $\mu_{\mathrm{h}} \approx 6$ $cm^2V^{-1}s^{-1}$, and the electron mobility of C13-PTCDI was $\mu_{\mathrm{e}} \approx 1.3$ $cm^2V^{-1}s^{-1}$, which were consistent with the previous reports[R17], [R18] (**Fig. R12**).

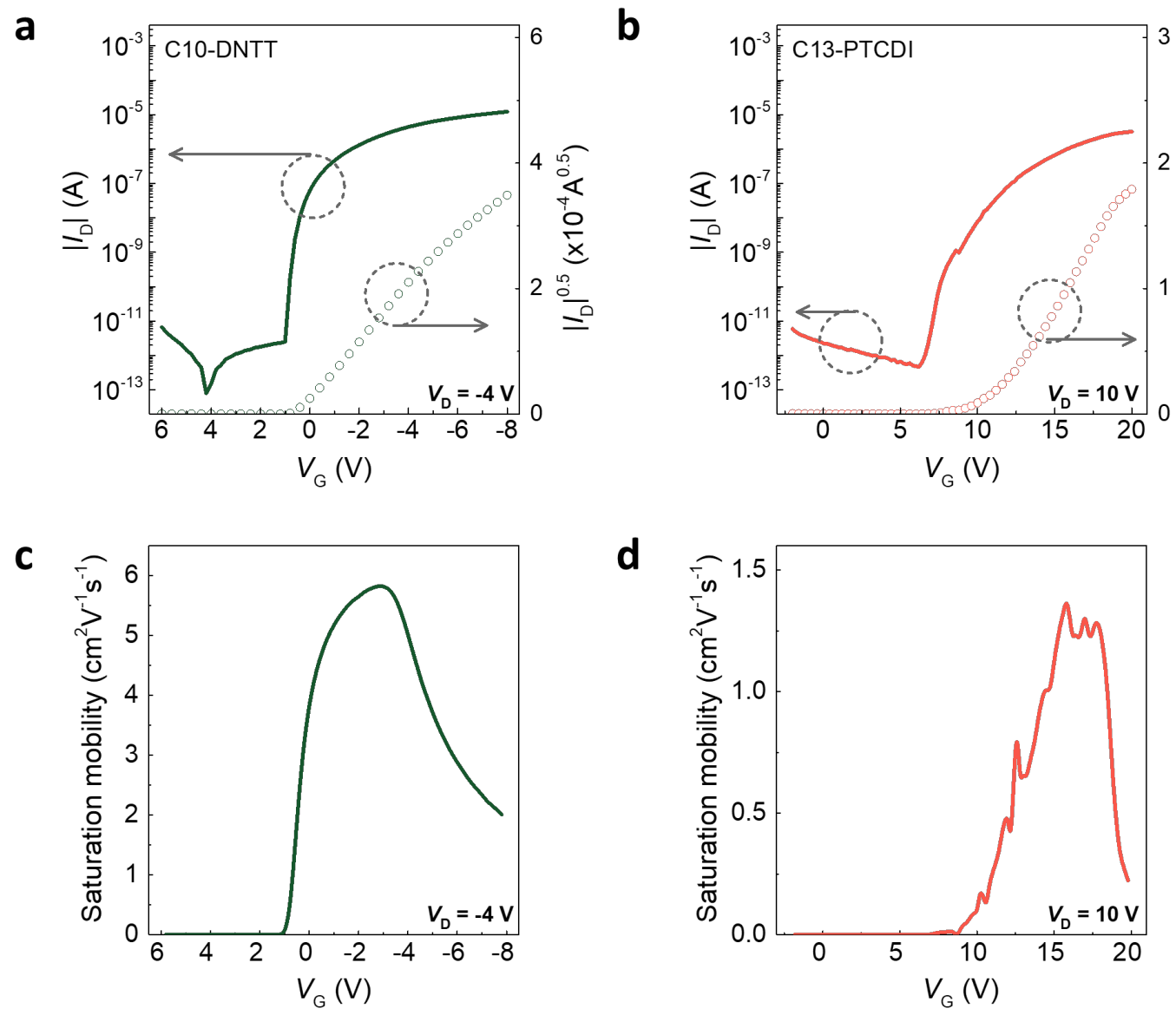

Figure R12. a. Transfer characteristics of C10-DNTT and **b**. C13-PTCDI thin-film transistors. **c**. μ profiles of C10-DNTT and **d**. C13-PTCDI thin-film transistors, extracted from their transfer characteristics.

In addition, to obtain a more rigorous band alignment for the p-type T-HTR, we constructed the source-side band profiles using calculation-derived schematic models constrained by experimentally determined boundary conditions. Note that the band alignment at the Au/CsF interface was obtained from the UPS results shown in Fig. 1f of the manuscript. The main operating mechanism, which will be discussed in more detail in the following **Q2**, is trap-assisted (Poole-Frenkel) conduction across the CsF layer. Thus, thickness of the CsF later mainly affects the field inside CsF and the length of the trap-mediated path. The voltage distribution across each dielectric layer was determined using the serial-capacitor relation derived from the unit-area capacitance.

$$C_i = \frac{\varepsilon_0 \varepsilon_{r,i}}{t_i}, \qquad \Delta V_i = \frac{t_i/\varepsilon_{r,i}}{\sum (t_l/\varepsilon_{r,j})} \times V_G, \qquad E_i = \frac{\Delta V_i}{t_i} = \frac{1/\varepsilon_{r,i}}{\sum (t_j/\varepsilon_{r,j})} \times V_G$$

where t_i is the thickness of the i-th dielectric layer, $\varepsilon_{r,i}$ is its relative permittivity, ε_0 is the vacuum permittivity, ΔV_i is the voltage drop across the i-th layer, and E_i is the corresponding electric field. Using the parameters of our structure (pV3D3: t = 50 nm, ε_r= 2.2; organic: t = 20 nm, ε_r= 3 for each[R19, R20]; CsF: t = 60 nm, ε_r= 7.9[R21]), the baseline voltage drop is

estimated to be approximately 52 % across pV3D3, 31 % across the organic heterojunction, and 17 % across CsF.

While we acknowledge that TCAD simulations would provide quantitative insights, it is challenging to incorporate all relevant physics – such as photo-exciton generation, carrier trapping, and Poole-Frenkel transport – as well as the necessary material-specific parameters with sufficient accuracy. Instead, we analyzed calculation-based schematic band diagrams that qualitatively reproduce the bias-dependent behaviors (**Fig. R13**). Here, the two organic layers (C13-PTCDI and C10-DNTT) were treated as dielectric segments due to the limitation of the band diagram program (Boise State University, GitHub: BoiseState/Multi-Dielectric).

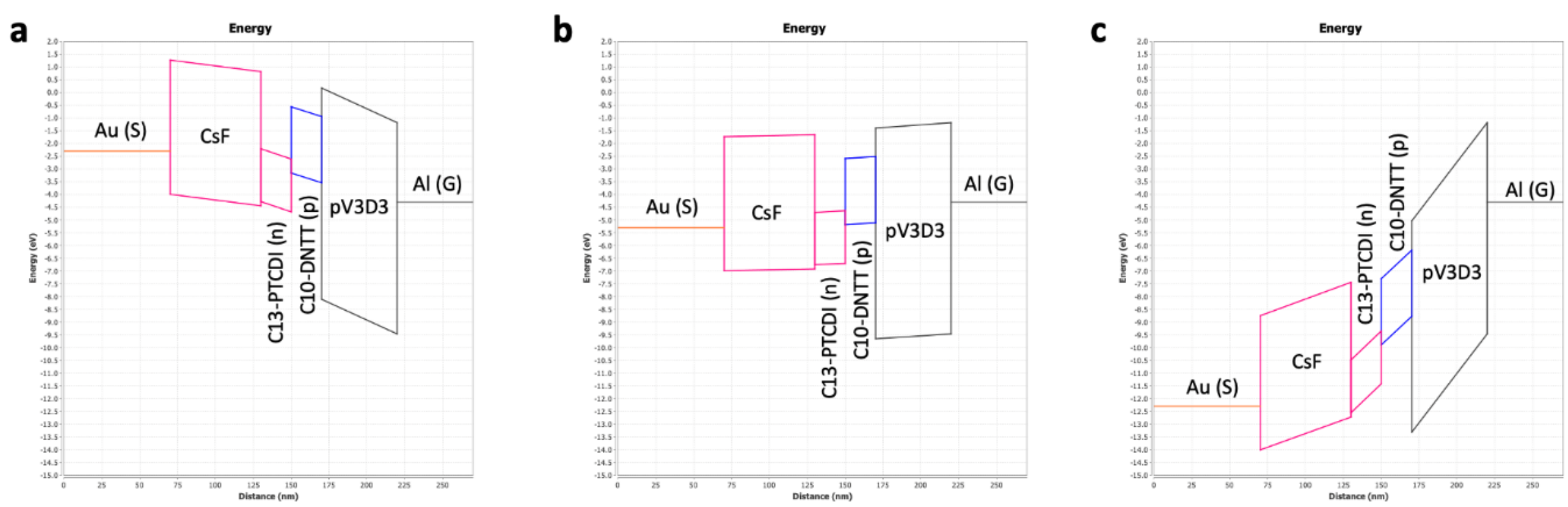

Figure R13. Schematic energy band diagrams of the source-side stack, constructed based on experimental boundary conditions. **a.** V_G = 2 V (OFF state), **b.** V_G = -1 V (intermediate state), and **c.** V_G = -8 V (ON state).

Based on the simulation-derived framework established in **Fig. R13**, the schematic structure of **Fig. R14** was first constructed to reproduce the fundamental band configuration, upon which the illumination-induced charge-trapping behavior was subsequently analyzed. Under illumination, photogenerated electrons are trapped at the C13-PTCDI and CsF interface, leading to an increase in the interfacial charge density with irradiance. The trapped charge (Q_{it}) can be described by the relation:

$$Q_{it} \simeq C_{ox} \times \Delta V_{TH}$$

where C_{ox} is the gate dielectric capacitance per unit area and ΔV_{TH} is the illumination-induced

V_{TH} shift. This trapped charge corresponds to a negative surface charge density at the interface, $\sigma = Q_{it}/area$, which enhances the internal electric field in CsF as follows.

$$\Delta E = \frac{\sigma}{\varepsilon_0 \varepsilon_r}$$

Because the negative surface charge is located at the C13-PTCDI/CsF interface, Gauss's law redistributes the potential drop across that series stack at fixed V_G, so that the $|E|$ in CsF increases (steeper band tilt), while the upstream pV3D3 is partially screened (flatter band tilt).

As the density of trapped electrons increases, the strengthened field modifies carrier transport across the CsF layer by Poole-Frenkel transport, expressed as:

$$J \sim E \times \exp\left[-\frac{e\left(\emptyset_B - \sqrt{\frac{eE}{\pi\varepsilon_i}}\right)}{kT}\right] \propto E \times \exp\left[\sqrt{E}\right]$$

where, J is the current density, E is the electric field, $\emptyset_B$ is the potential barrier height, e is the elementary charge, ε_i is the dielectric permittivity, k is Boltzmann's constant and T is the temperature. The Poole-Frenkel conduction originates from bulk and interfacial traps within CsF, as the enhanced internal field increases the pre-exponential factor of the current density and reduces the effective barrier height, collectively resulting in an enhancement of the current.

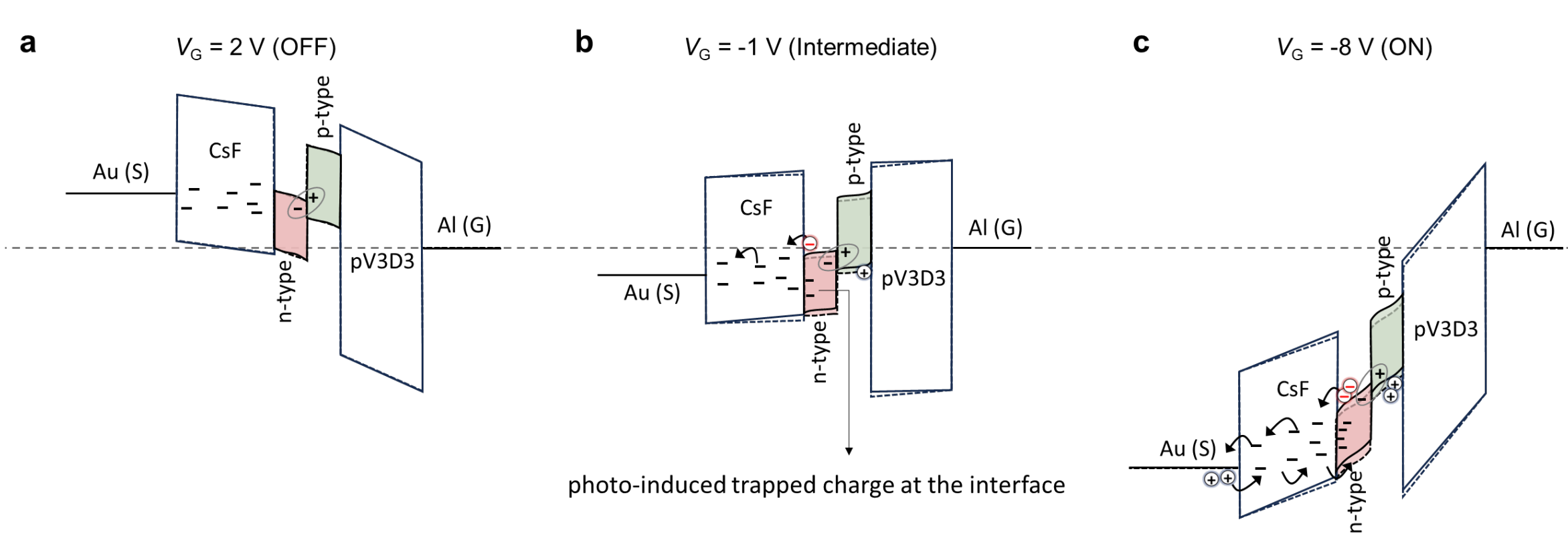

Figure R14. Schematic energy band diagrams of the p-type T-HTRs at the source-side, in the dark (dashed lines) and under illumination (solid lines) at three representative V_G states: **a.** V_G

= 2 V (OFF), **b**. -1 V (intermediate), and **c**. -8 V (ON).

The accumulation of interfacial charge therefore leads to a stronger internal field in CsF, which further promotes the multistep trap-assisted transport across the CsF layer. Simultaneously, the negative charge at the interface photogates the p-type C10-DNTT channel and redistributes the applied potential, such that a larger portion drops across CsF. This mechanism accounts for the increased slope of the vacuum level and band edges that is observed in the illuminated state.

We have revised the manuscript by explicitly including the missing device parameters and by providing more rigorous band alignments.

[R16] *Nature materials*, **14**(6), 628-635 (2015).
[R17] Science advances, **6**(21), eaaz5156 (2020).
[R18] Applied Physics Letters, **97**(24) (2010).
[R19] *Journal of Materials Chemistry C*, **10**(2), 726-733 (2022).
[R20] *MRS Online Proceedings Library*, **1091**, 1091-AA05 (2008).
[R21] *Physical Review*, **168**(3), 1054 (1968).

The revised parts are as follows;

(1) Revised the main text in the revised manuscript (p. 8).

In this study, 2,9-Didecyldinaphtho[2,3-b:2',3'-f]thieno[3,2-b]thiophene (C10-DNTT, 20 nm) and N,N'-Ditridecyl-3,4,9,10-perylenetetracarboxylic diimide (C13-PTCDI, 20 nm) were utilized as the p-type and n-type channel materials, respectively, both of which are well-established high-mobility organic semiconductors, exhibiting hole and electron mobilities exceeding 1 $cm^2 V^{-1} s^{-1}$ (**Supplementary Fig. 1**). They are also compatible with sequential vacuum evaporation, thereby facilitating the construction of p-type/n-type heterojunction structure[34].

(2) Revised the main text in the revised manuscript (p. 9).

A poly(1,3,5-trimethyl-1,3,5-trivinyl cyclotrisiloxane) (pV3D3, 50 nm) layer deposited in

vapor-phase via initiated chemical vapor deposition (iCVD) was utilized as the gate dielectric layer[41]. The dielectric constant of the pV3D3 film was determined to be approximately 2.2, from which the dielectric thickness in the T-HTR was determined to be 50 nm (**Supplementary Fig. 2**).

(3) Revised the main text in the revised manuscript (p. 10).

In this study, a 60 nm-thick CsF layer was employed to enhance the injection barrier functionality beyond dipole formation. To elucidate the charge-transport mechanism through this thick CsF layer, we fabricated vertical Au/C10-DNTT (20 nm)/C13-PTCDI (20 nm)/CsF (0 to 90 nm)/Au stacks that replicate the source-side configuration of the T-HTR. The current density (J_i)–electric field (E_i) characteristics of this stacked device showed a systematic decrease in J_i with increasing CsF thickness, confirming that a thicker barrier effectively suppresses carrier transmission (**Supplementary Fig. 4**). Notably, when the experimental data were analyzed in Poole-Frenkel coordinates [$\ln(J_\mathrm{i}/E_\mathrm{i})$ versus $\sqrt{E_\mathrm{i}}$][47,48], excellent linearity was obtained, indicating that carrier transport is dominated by trap-assisted Poole-Frenkel conduction. In this mechanism, a thicker CsF layer reduces the average electric field and extends the trap-mediated path, thereby lowering the overall transmission probability. To further quantify the semiconductor energy levels used for band alignment, UPS analysis was also performed on the organic semiconductors. The ionization energies extracted from the UPS spectra were 4.92 eV for C10-DNTT and 6.42 eV for C13-PTCDI (**Supplementary Fig. 5**). Subsequently, the optical bandgaps were obtained from UV-vis absorption measurements using Tauc analysis (**Supplementary Fig. 6**), yielding values of 2.59 eV and 2.06 eV for C10-DNTT and C13-PTCDI, respectively. By combining the UPS-derived ionization energies with the optical bandgaps, the electron affinities were determined to be 2.33 eV for C10-DNTT and 4.36 eV for C13-PTCDI.

The energy band diagram of the T-HTR from gate to source illustrates how the thick CsF layer governs the source-contact behavior in the light-induced intermediate state (**Fig. 1g**). Under illumination, exciton dissociation at the p-n heterojunction generates

minority electrons that drift toward the CsF side. In this configuration, both hole injection from the source and light-induced minority-electron transport from the n-type layer are available through the trap-assisted Poole-Frenkel process, but the band alignment makes electron transport energetically favorable while rendering hole injection highly unfavorable: the apparent work function of the Au/CsF contact (~3.8 eV) places its Fermi level above the lowest unoccupied molecular orbital (LUMO) level of C13-PTCDI (2.06 eV), whereas the much deeper highest occupied molecular orbital (HOMO) level of the C10-DNTT (4.92 eV) imposes a large barrier. Notably, photogenerated holes can still be transported through the p-type channel toward the drain, contributing to photocurrent, even though gate-induced hole injection from the source remains energetically suppressed. Consequently, field-effect current remains suppressed, whereas photocurrent can readily flow through the trap-assisted pathway, giving rise to the stable intermediate conduction state. Meanwhile, a fraction of minority electrons that drift toward the CsF side is likely captured by interfacial states at the C13-PTCDI/CsF junction. This establishes a photo-induced dipole and alters the potential distribution across the series dielectric stack at a fixed V_G via Gauss's law, increasing the electric field in the CsF layer (steeper band slope) while partially screening the upstream pV3D3 layer (flatter band slope). This enhanced electric field in CsF lowers the effective barrier for trap-assisted Poole-Frenkel conduction, leading to large threshold voltage (V_{TH}) shift in devices incorporating a CsF injection barrier under illumination, as will be discussed later.

The band configurations for the off- and on-states are also provided in **Supplementary Fig. 7**, together with the intermediate-state band diagram. In the off state, the channel is fully depleted; even under illumination, photogenerated carriers cannot contribute meaningfully to conduction because they cannot percolate through the depleted channel. In the on state, under strong gate bias, the electric field effectively reduces the hole-injection barrier through CsF and enables efficient hole transport into the p-type channel, with photocurrent further augmenting the total conduction. To clearly illustrate the role of the CsF/n-type/p-type stack at the source side of the T-HTR, band

diagrams for devices with reversed source/drain configuration are also provided in **Supplementary Fig. 7** for direct comparison.

(4) Replaced Fig. 1g with **Figure R14b** in the revised manuscript (p. 31).

Figure 1 | Design of the ternary heterojunction transistor (T-HTR). a. The schematic illustration and **b**. optical microscopy image of the T-HTR (scale bar: 500 μm). **c**. The schematic illustration of transfer characteristics and **d**. operational mechanism of the T-HTR consisting of three switching states. **e**. Ultraviolet-visible (UV/Vis) absorption spectra of the n-type (C13-PTCDI), p-type (C10-DNTT) and n-type/p-type bilayer organic semiconductors. **f**. Ultraviolet photoelectron spectroscopy (UPS) spectra of Au contact with thermally deposited cesium fluoride (CsF) as a function of CsF thickness. **g**. Schematic energy band diagrams of the source-side stack in the dark (dashed lines) and under illumination (solid lines). The trapped charges at the CsF/p-type interface enhances the internal electric field in CsF.

(4) Added the **Figure R9** to Supplementary Information (p. 3)

(5) Added the **Figure R10** to Supplementary Information (p. 8)

(6) Added the **Figure R11** to Supplementary Information (p. 7)

(7) Added the **Figure R12** to Supplementary Information (p. 1)

(8) Added the **Figure R14** to Supplementary Information together with the corresponding descriptions (p. 9).

In the off-state, the channel remains fully depleted, preventing the formation of a conductive pathway; as a result, charge transport is effectively suppressed and photogenerated carriers cannot percolate through the channel, yielding negligible current even under illumination. In the intermediate state, the engineered injection barrier at the source suppresses gate-induced hole injection while allowing photogenerated carriers to flow, thereby producing a gate-voltage-independent

photocurrent plateau with zero transconductance (g_m ≈ 0). In the on-state, a sufficiently strong V_G reduces the source-side hole-injection barrier and enables efficient hole transport through the p-type channel, with the photocurrent providing an additional contribution to the total conduction.

The effect of drain bias (V_D) also influences the source-side band. At a small V_D, the potential drop along the channel is minor, so the field across CsF is mainly governed by the V_G. In this regime, trap-assisted conduction dominates with a relatively small field enhancement. As V_D increases, part of the drain potential also drops across the source-side dielectric stack, effectively increasing the internal field in CsF. This enhanced field strengthens Poole-Frenkel conduction by lowering the effective barrier ($\emptyset_B - \sqrt{eE_{eff}/\pi\varepsilon_i}$), resulting in an increase of current ($J \propto E_{eff} \times \exp[\sqrt{E_{eff}}]$). Here, E_{eff} represents the combined field composed of the gate-induced component and the additional field induced from the drain bias and photogenerated interfacial charge accumulation.

When the source and drain are reversed, the source directly contacts the p-type channel without the injection barrier, enabling hole injection and resulting in behavior analogous to that of a conventional transistor without an intermediate state.

(9) Added the reference [47-48] in the revised manuscript.

Q2) The insertion of CsF layer serves as blocking barrier for source carrier injection. In this case, do carriers from the source electrode tunnel through the CsF layer? If so, the thickness of the CsF layer will play a pivotal role in determining the tunneling probability. Please clarify on that.

Response:

We thank the Reviewer for raising this important point regarding the injection mechanism through the CsF layer. Since the CsF layer is 60 nm thick, direct tunneling or Fowler-Nordheim (F-N) tunneling is physically negligible. To directly examine the effect of CsF

thickness, we fabricated vertical Au/C10-DNTT (20 nm)/C13-PTCDI (20 nm)/CsF (0 to 90 nm)/Au devices mimicking the source-side of the T-HTR and measured their current density-electric field (J_i–E_i) characteristics. (**Fig. R15a, b**). The results showed that the current density systematically decreased as the CsF thickness increased, confirming that a thicker CsF barrier more effectively suppressed carrier transmission. Importantly, when we analyzed the conduction mechanism, the experimental data exhibited excellent linearity in Poole-Frenkel plots [ln(J_i/E_i) vs $\sqrt{E_i}$] (**Fig R15c**)[R22], [R23], indicating that carriers do not traverse the CsF layer via direct tunneling, but through trap-assisted hopping conduction described by the Poole-Frenkel mechanism.

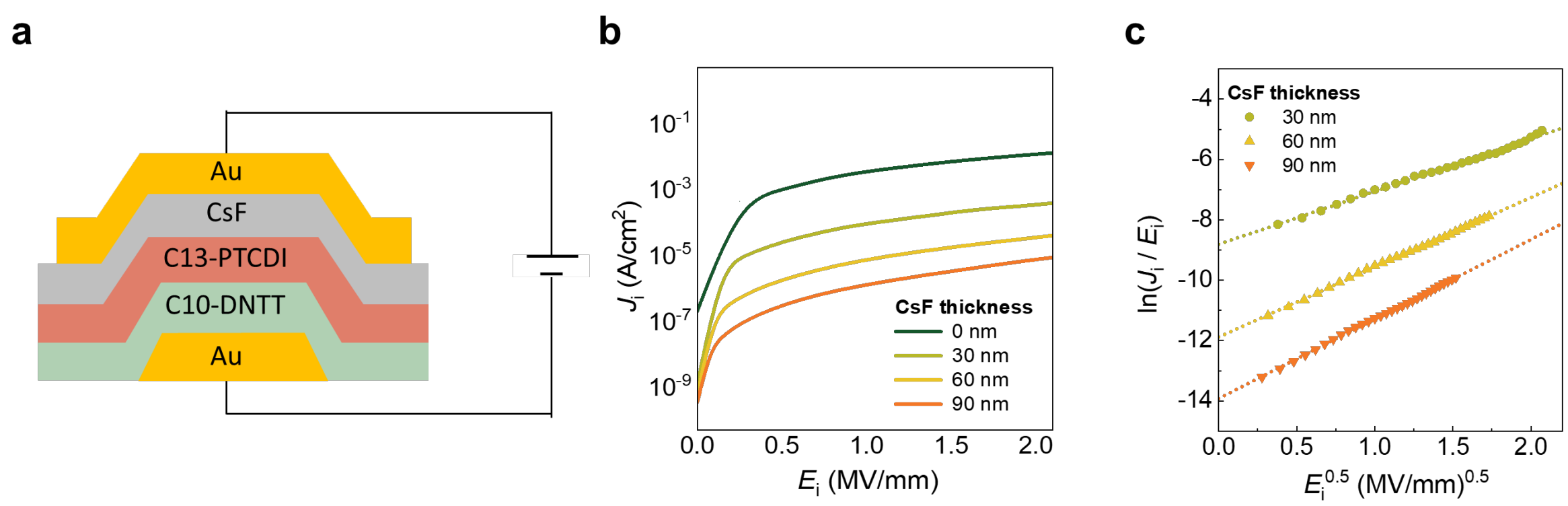

Figure R15. a. Schematic illustration of the Au/C10-DNTT (20 nm)/C13-PTCDI (20 nm)/CsF (0 to 90 nm)/Au vertical stack structure used to emulate the source-side configuration of the T-HTR. **b**. Current density–electric field($J_i - E_i$) characteristics of the vertical devices with different CsF thicknesses, showing a systematic reduction of current with increasing barrier thickness. **c**. Poole-Frenkel plots derived from the $J_i - E_i$ data, exhibiting linear behavior consistent with trap-assisted Poole-Frenkel conduction.

In this framework, the role of thickness can be rationalized as follows: with increasing the CsF thickness, (i) the average electric field is reduced, alleviating the barrier-lowering effect in the Poole-Frenkel conduction process, and (ii) the trap-mediated pathway becomes longer, lowering the effective transmission probability. As a result, the injection probability decreases progressively with the thickness increase. This interpretation is fully consistent with our vertical-stack measurements and with the CsF-thickness dependent T-HTR characteristics, in which the V_G-independent intermediate state becomes wider and more stable as the CsF thickness increases.

We have revised the manuscript by clarifying the injection mechanism as Poole-Frenkel conduction and by adding the corresponding experimental validation with vertical-stack devices.

[R22] *Physical Review*, **155**(3), 657 (1967).
[R23] *ACS Applied electronic materials*, **3**(9), 3674-3692 (2021).

(1) Revised the main text in the revised manuscript (p. 10).

In this study, a 60 nm-thick CsF layer was employed to enhance the injection barrier functionality beyond dipole formation. To elucidate the charge-transport mechanism through this thick CsF layer, we fabricated vertical Au/C10-DNTT (20 nm)/C13-PTCDI (20 nm)/CsF (0 to 90 nm)/Au devices that replicate the source-side configuration of the T-HTR. The current density (J_i)–electric field (E_i) characteristics of this stacked device showed a systematic decrease in J_i with increasing CsF thickness, confirming that a thicker barrier effectively suppresses carrier transmission (**Supplementary Fig. 4**). Notably, when the experimental data were analyzed in Poole-Frenkel coordinates [$\ln(J_i/E_i)$ versus $\sqrt{E_i}$][47,48], excellent linearity was obtained, indicating that carrier transport is dominated by trap-assisted Poole-Frenkel conduction. In this mechanism, a thicker CsF layer reduces the average electric field and extends the trap-mediated path, thereby lowering the overall transmission probability.

(2) Added the **Figure R15** to Supplementary Information, together with the corresponding descriptions (p. 5).

To directly examine the effect of CsF thickness, we fabricated vertical Au/C10-DNTT (20 nm)/C13-PTCDI (20 nm)/CsF (0 to 90 nm)/Au stacks mimicking the source-side of the T-HTR and measured their J_i–E_i characteristics. (**Supplementary Fig. 4a, b**). The results showed that the current density systematically decreased as the CsF thickness increased, confirming that a thicker CsF barrier more effectively suppressed carrier transmission. Importantly, when we analyzed the conduction mechanism, the experimental data exhibited excellent linearity in Poole-Frenkel plots [$\ln(J_i/E_i)$ versus $\sqrt{E_i}$] (**Supplementary Fig. 4c**), indicating that carriers do not

traverse the CsF layer via direct tunneling, but instead through trap-assisted hopping conduction described by the Poole-Frenkel mechanism:

$$J \sim E \times \exp\left[-\frac{e\left(\emptyset_{\mathrm{B}} - \sqrt{\frac{eE}{\pi \varepsilon_i}}\right)}{kT}\right] \propto E \times \exp\left[\sqrt{E}\right]$$

where, J is the current density, E is the electric field, $\emptyset_{\mathrm{B}}$ is the potential barrier height, e is the elementary charge, ε_i is the dielectric permittivity, k is Boltzmann's constant and T is the temperature.

(3) Added the reference [47-48] in the revised manuscript.

Q3) In view of dominating mechanism for the transistor design, the thickness and doping level of both p-type and n-type semiconductors will also have a significant impact on the performance, which determines the magnitude of built-in electric field and the depletion width of the heterojunction. Please clarify on that.

Response:

We fully agree with the reviewer that the thickness and doping level of both the p- and n-type semiconductors are important parameters that influence the heterojunction properties. In conventional inorganic p-n junctions, these parameters directly determine the built-in electric field and the depletion width, thereby governing exciton dissociation through field-driven mechanisms. However, in organic small-molecule semiconductors such as C10-DNTT and C13-PTCDI used in this study, the junction generally forms an accumulation-type heterojunction rather than a depletion-type junction[R24]. This arises because both C10-DNTT and C13-PTCDI are intrinsically p- and n-type semiconductors irrelevant to doping, with the Fermi level of C13-PTCDI lying deeper than that of C10-DNTT. Upon contact, therefore, holes tend to accumulate on the p-type side while electrons accumulate on the n-type side at the interface. In such accumulation-type heterojunctions, the built-in potential dictates the degree of interfacial charge accumulation rather than an extended depletion field.

Regarding the thickness, the optical absorption follows the Beer–Lambert law; thicker layers absorb more photons and generate more excitons, while thinner layers transmit more light and reduce exciton generation. Meanwhile, exciton harvesting is limited by the finite exciton diffusion length, which in organic semiconductors is typically in the range of 5–20 nm[R25]. If the layer thickness exceeds this range, most of excitons recombine before reaching the heterojunction, lowering the photocurrent efficiency. Likewise, if the films are too thin, insufficient light absorption reduces exciton generation. In addition, the charge transport properties of organic semiconductors are also thickness-dependent due to their unique polycrystalline growth morphology formed during vacuum deposition. A thickness more than 10 nm is generally required to yield a laterally continuous crystalline layer that establishes effective pathways for charge carrier transport[R26, R27, R28]. On the other hand, if the films are made substantially thicker, the contact resistance at the source interface tends to increase, which degrades the injection efficiency[R29]. For these reasons, organic thin-film transistors generally employ semiconductor layers with the thicknesses around 20 nm. Therefore, in our T-HTR, we employed 20 nm-thick C10-DNTT and C13-PTCDI layers as the optimal thickness, which not only closely match their reported exciton diffusion lengths but also ensure favorable morphology for efficient charge transport while minimizing the contact resistance, thereby achieving balanced absorption, exciton harvesting, and carrier injection at the heterojunction interface.

Regarding doping, while our organic semiconductors are essentially intrinsic, intentional doping in organics can be achieved, if necessary, typically by introducing molecular dopants that undergo charge transfer with the host semiconductor, thereby shifting the Fermi level rather than substituting atoms as in inorganics[R30]. A larger Fermi-level difference between the two semiconductors increases the interfacial accumulation of carriers, strengthening the energetic driving force for exciton dissociation. Conversely, stronger p-doping in C10-DNTT shifts its Fermi level closer to the HOMO, while stronger n-doping in C13-PTCDI shifts its Fermi level closer to the LUMO. These shifts reduce the equilibrium Fermi-level offset and thus decrease the built-in potential, while opposite (compensating) doping would have the reverse effect.

While thickness and doping indeed modulate exciton generation and interfacial dissociation efficiency, it is important to note that the central focus of our study is not on optimizing the exciton dissociation itself but on establishing V_G-independent intermediate state.

This intermediate state is uniquely enabled by the engineered injection barrier at the organic semiconductor/source interface, which efficiently suppresses gate-induced carrier injection while allowing photogenerated carriers to be transported. Consequently, the stable intermediate photocurrent arises predominantly from barrier engineering rather than from the built-in potential of the heterojunction. Thus, thickness and doping levels serve only as auxiliary tuning factors, whereas the injection barrier governs the stability and functionality of the intermediate state, which represents the key innovation of our T-HTR design.

We have revised the manuscript to provide the rationale for selecting a 20 nm semiconductor thickness based on exciton diffusion and transport considerations, and to clarify the role of molecular doping as a potential Fermi-level tuning strategy in organic heterojunctions.

[R24] *Ma, D., & Chen, Y. Organic Semiconductor Heterojunctions and Its Application in Organic Light-Emitting Diodes*, Springer Series in Materials Science, Vol. **250**, Springer, Berlin/Heidelberg, Germany (2017).
[R25] *Energy & Environmental Science*, **8**(7), 1867-1888 (2015).
[R26] *Organic Electronics*, **14**(12), 3213-3221 (2013).
[R27] *Advanced Materials*, **17**(14), 1795-1798 (2005).
[R28] *Materials*, **13**(7), 1583 (2020).
[R29] *Advanced Materials*, **34**(2), 2104075 (2022).
[R30] *Advanced Materials*, **29**(42), 1703063 (2017).

(1) Revised the main text in the revised manuscript (p. 8).

They are also compatible with sequential vacuum evaporation, thereby facilitating the construction of p-type/n-type heterojunction structure[34]. A thickness of 20 nm for both semiconductors was selected, as it closely matches the reported exciton diffusion length in these small-molecule systems[35] and ensures a continuous crystalline morphology for efficient charge transport[35,37,38], while avoiding contact-resistance penalties associated with thicker films[39].

(2) Revised the main text in the revised manuscript (p. 20).

These results indicate the structural versatility of the T-HTR design: the light-tunable

intermediate state is not specific to the CsF-based device, but is a general feature of the proposed architecture enabled by appropriate injection barrier engineering and energy level alignment. Furthermore, although the devices investigated here employ essentially intrinsic organic semiconductors, molecular doping in organic systems, if utilized, can shift the Fermi level via charge-transfer interactions, thereby modulating the built-in potential and the exciton-dissociation driving force at the heterojunction. Importantly, such doping-induced Fermi-level tuning does not substitute the role of the injection barrier but rather provides an additional degree of freedom for tailoring the interfacial energetics in conjunction with barrier engineering, further reinforcing the versatility of the T-HTR architecture.

(3) Added the reference [35-39] in the revised manuscript.

Q4) The time-resolved response of the transistor relies on two processes: the separation of phonon-generated carriers and the transit of carriers within the channel. Therefore, the channel mobility and length will have significant impact on the transient response. Whether the scaling of the channel length will improve the time-resolved performance? Please provide additional experimental or simulation results.

Response:

We sincerely appreciate the Reviewer's insightful question. To clarify, we systematically fabricated p-type T-HTR with channel length (L) split from 100 μm to 400 μm and measured both transfer curves and time-resolved response under identical bias conditions (V_{G} = -0.5 V, V_{D} = -4 V) (**Fig. R16a**). The results clearly showed that rise/fall times (τ) exhibited negligible dependence on L (**Fig. R16b**). This behavior is well explained by a contact-dominated RC delay introduced by the injection barrier at the source. Using a simple compact model[R31],

$$\tau \approx (R_{\mathrm{C}} + R_{\mathrm{ch}}) \times C_{\mathrm{eff}}$$

where R_{C} is the contact resistance, R_{ch} is the channel resistance (here, R_{ch} proportional to L), and C_{eff} is the effective capacitance. When the barrier suppresses carrier

injection effectively, R_{C} is much larger than R_{ch}. Consequently, the response time is determined primarily by R_{C} and C_{eff}, and thus becomes effectively independent of L. Therefore, the response speeds in the proposed devices are governed primarily by the contact/barrier RC at the source interface.

We have revised the manuscript by including the transient measurements across different channel lengths and the corresponding RC delay analysis to demonstrate the contact-dominated response.

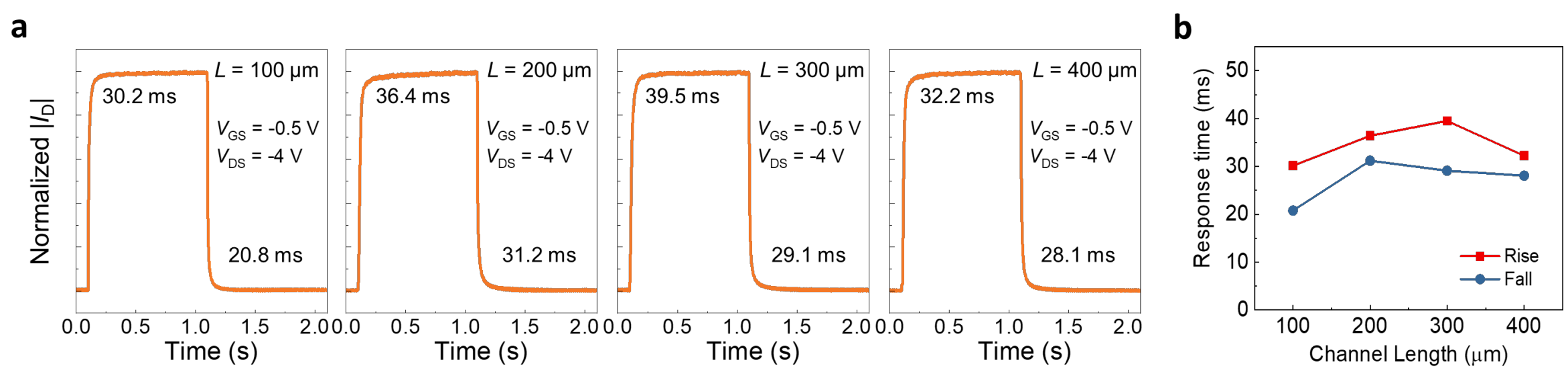

Figure R16. a. Transient responses of devices with a CsF injection barrier for channel lengths (L) of 100 µm, 200 µm, 300 µm, and 400 µm. The drain current is normalized with maximum value. **b**. The extracted rise and fall times as a function of L.

[R31] *Small*, **8**(1), 73-79 (2012)

(1) Revised the main text in the revised manuscript (p. 17).

In contrast, with the 60 nm-thick CsF injection barrier, the rising time was significantly reduced to 34.3 ms, and the falling time shortened to 31.2 ms, resulting in a much sharper transient response with well-defined on/off switching behavior. This improvement suggests that the CsF layer effectively suppresses the dominant trap-mediated relaxation pathways at the metal/semiconductor interface. We note, however, that the reversible V_{TH} modulation observed under illumination indicates the possible presence of a small number of rapidly responding interfacial states or dipole-forming sites at the CsF/semiconductor boundary. These states may transiently accommodate photocarriers without inducing long-lived storage, consistent with the negligible hysteresis and rapid signal recovery observed.

Given that the incorporation of CsF renders the device contact-resistance dominated, the possible influence of channel transport on the transient response was further examined by varying the channel length of the T-HTR from 100 to 400 μm (**Supplementary Fig. 15**). The rising and falling times exhibited negligible dependence on channel length, indicating that the temporal response is not channel-limited but governed by a contact-dominated RC delay introduced by the source-side injection barrier. This behavior can be described by a simple RC model [$\tau \sim (R_C + R_{ch}) \times C_{eff}$][61], where the barrier yields much higher contact resistance (R_C) than channel resistance (R_{ch}), making the transient time effectively independent of channel length.

(2) Added the **Figure R16** to Supplementary Information (p. 21).

(3) Added the reference [61] in the revised manuscript.

Q5) In Line 204 of this manuscript, the authors mentioned that the increment of light intensity leads to the negative shift of threshold voltage. Generally, the threshold voltage is mainly determined by the height of source barrier for a transistor. A clearer description should be given to shed light upon this point.

Response:

We appreciate the Reviewer's constructive and thoughtful comment. The negative shift of V_{TH} with increasing light intensity does not imply a permanent change of the intrinsic source barrier height. Rather, light creates an internal photovoltage at the source-side p-n heterojunction via (i) accumulation/trapping of the photogenerated minority carriers and (ii) formation of an interfacial dipole induced by separation between the photogenerated minority carriers and their opposite charges. These electrostatic effects lower the effective hole-injection barrier seen by the channel, so the gate needs less bias to turn the device on, and thus the extracted V_{TH} (an apparent threshold) shifts to more negative values. In terms of band diagram, the interfacial charge redistributes the series potential drop at a fixed V_G, increasing the fraction that drops across CsF:

$$\Delta V_{CsF} \approx (C_{ox}/C_{CsF}) \times |\Delta V_{TH}|$$

where C_{ox} and C_{CsF} denote the capacitances per unit area of the gate dielectric and CsF layer, respectively, and ΔV_{TH} represents the light-induced shift in threshold voltage arising from interfacial charge accumulation and dipole formation. Therefore, conduction band on the organic/CsF side shifts by

$$\Delta E_{\mathrm{c}} \approx q\Delta V_{\mathrm{CsF}} \approx q\left(\frac{C_{\mathrm{ox}}}{C_{\mathrm{CsF}}}\right)|\Delta V_{\mathrm{TH}}|,$$

while the pV3D3 energy band correspondingly flattens. Consequently, the gate bias required for channel formation decreases and ΔV_{TH} accordingly to negative direction in the T-HTR:

$$|\Delta V_{\mathrm{TH}}| \propto \frac{\Delta Q_{\mathrm{dipole}} + \Delta Q_{\mathrm{photo}}}{C_{\mathrm{ox}}}$$

where $\Delta Q_{\mathrm{dipole}}$ represents the charge separation-induced interfacial dipole moment and $\Delta Q_{\mathrm{photo}}$ denotes the accumulated/trapped photogenerated charge.

When trap states near the barrier become saturated, the photovoltage no longer increases and the V_{TH} shift saturates, converging to the value of the control device without the barrier. This shows that the barrier primarily raises the dark V_{TH} by suppressing the majority carrier injection from the source, and illumination compensates it through contact modulation (**Fig. R17**). For completeness, as discussed in **Q2**, our 60-nm CsF layer is too thick for direct or Fowler–Nordheim tunneling. The source-side conduction follows trap-assisted Poole-Frenkel transport. Light modifies the local space-charge/electric field distribution, therefore reducing the effective injection barrier rather than the material barrier height.

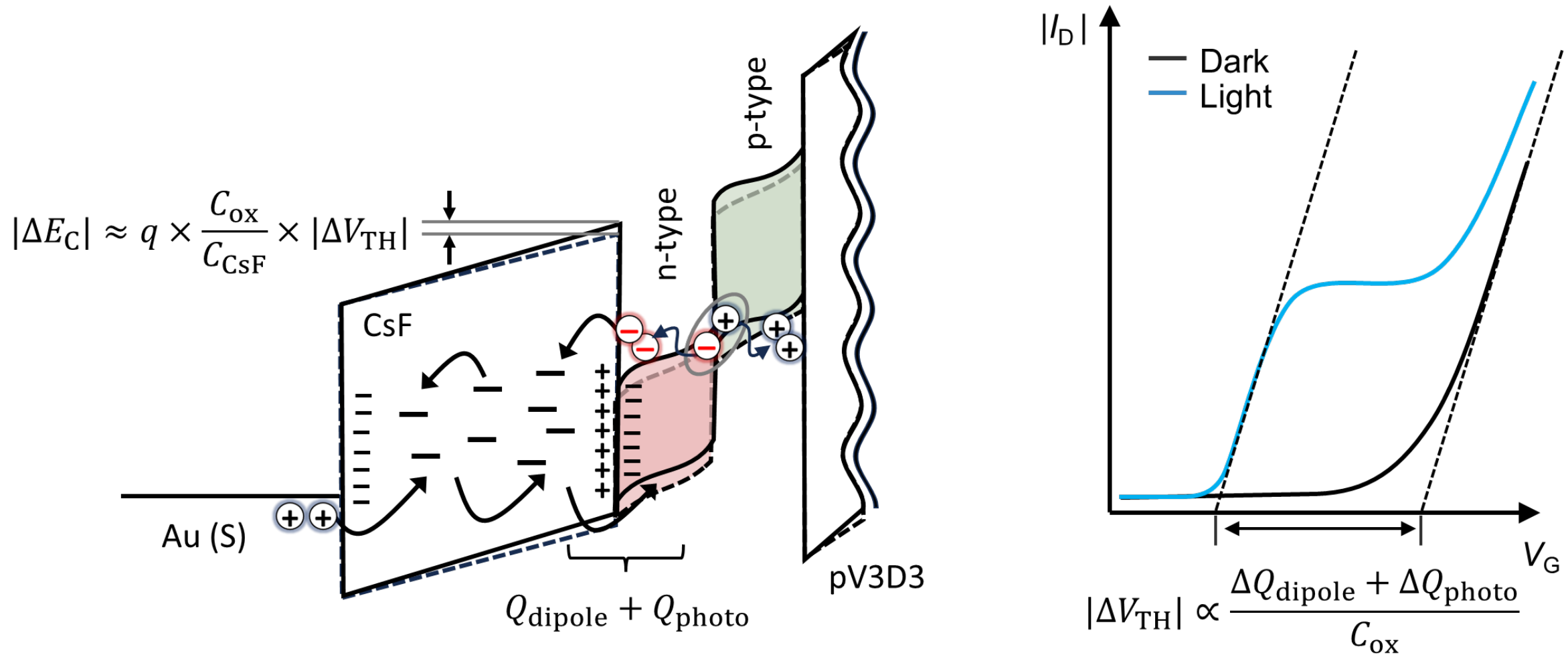

Figure R17. Schematic illustration of the illumination-induced V_{TH} shift in the T-HTR.

We have revised the manuscript by providing a clearer description of how illumination induces effective barrier lowering via electrostatic effects rather than permanent changes of the source barrier.

The revised parts are as follows;

(1) Revised the main text in the revised manuscript (p. 12).

The transfer characteristics of the T-HTR were investigated (**Fig. 2a**). Under dark condition, the T-HTR exhibited typical on and off switching behavior. On the other hand, upon 460 nm light illumination, a constant photocurrent was observed between the on and off-states, defining the intermediate state. As the light intensity increased, the V_{TH} decreased gradually, which is attributed to the internal photovoltage generated by interfacial dipoles and the accumulation of photogenerated minority carriers beneath the injection-barrier layer[49,50,51,52]. These trapped charges locally bend the energy bands, effectively lowering the gate bias required for channel formation (**Supplementary Fig. 8**).

(2) Added the **Figure R17** to Supplementary Information together with the corresponding descriptions (p. 11).

Under illumination, excitons at the heterojunction interface dissociate: photogenerated minority carriers are driven toward the source and accumulate/trap beneath the blocking layer, while photogenerated majority carriers are transported through the channel toward the drain. In the p-type T-HTR, the accumulated/trapped photogenerated electrons at the semiconductor/CsF interface induce a positive charge on the CsF side, forming an interfacial dipole moment. The accumulated interfacial charge and dipole create an internal photovoltage and band bending that modulate the contact, which redistributes the series potential drop at a fixed V_{G}, increasing the fraction that drops across CsF:

$$\Delta V_{\mathrm{CsF}} \approx (C_{\mathrm{ox}}/C_{\mathrm{CsF}}) \times |\Delta V_{\mathrm{TH}}|$$

where C_{ox} and C_{CsF}) denote the capacitances per unit area of the gate dielectric and CsF layer, respectively, and ΔV_{TH} represents the light-induced shift in threshold voltage arising from interfacial charge accumulation and dipole formation. Therefore, conduction band on the organic/CsF side shifts by

$$\Delta E_{\mathrm{c}} \approx q\Delta V_{\mathrm{CsF}} \approx q\left(\frac{C_{\mathrm{ox}}}{C_{\mathrm{CsF}}}\right)|\Delta V_{\mathrm{TH}}|,$$

while the pV3D3 energy band correspondingly flattens. Consequently, the gate bias required for channel formation decreases and ΔV_{TH} accordingly to negative direction in p-type T-HTR (and positively for n-type T-HTRs):

$$|\Delta V_{\mathrm{TH}}| \propto \frac{\Delta Q_{\mathrm{dipole}} + \Delta Q_{\mathrm{photo}}}{C_{\mathrm{ox}}}$$

where $\Delta Q_{\mathrm{dipole}}$ represents the charge separation-induced interfacial dipole moment and $\Delta Q_{\mathrm{photo}}$ denotes the accumulated/trapped photogenerated charge.

As the light intensity increases, the numerator ($\Delta Q_{\mathrm{dipole}} + \Delta Q_{\mathrm{photo}}$) increases, leading to a gradual decrease in V_{TH}. When trap sites beneath the barrier become saturated, the internal photovoltage no longer increases and V_{TH} saturates, converging to the value of the control device without the blocking layer. This indicates that the barrier layer raises the initial V_{TH} in the dark by suppressing minority carrier injection from the source, while strong illumination compensates this barrier via light-induced contact modulation.

Q6) In **Supplementary Table I**, the units for the static power consumption of the last two entries should be corrected from 'A' to 'W' for consistency. Additionally, since the table includes devices with varying geometries, a fair comparison of static power consumption should be normalized by device area. Furthermore, power consumption and performance are inherently trade-offs in transistor design. An excessively low current density will lead to poor device performance, such as slow switching speeds. Thus, more detailed discussions and comprehensive figures illustrating this trade-off would strengthen the analysis.

Response:

We thank the Reviewer for the constructive suggestions. We have corrected the units accordingly and have normalized the static power consumption by the active channel area (**Table R2**).

Table R2 | Comparison of static power consumption with the previously reported ternary logic transistor.

Operating principle	Driving voltage at logic '1'	Static current at logic '1'	Channel (length × width)	Static power consumption at logic '1'	Ref
Negative transconductance	20 V	> 10 nA	400 μm × 1,000 μm	> 50 μW/cm^2	[10]
Negative transconductance	3 V	> 1 nA	500 μm × 1,000 μm	> 0.6 μW/cm^2	[11]
Negative transconductance	> 30 V	> 100 nA	1,000 μm × 2,200 μm	> 136 μW/cm^2	[12]
Negative transconductance	1 V	> 0.1 nA	1 μm × 3 μm	> 3.3 mW/cm^2	[13]
Negative transconductance	2 V	> 1,000 nA	3 μm × 20 μm	> 3.3 W/cm^2	[14]

Zero transconductance	1 V	6 nA	100 μm × 150 μm	40 μW/cm^2	[15]
Zero transconductance	1 V	4 nA	60 μm × 80 μm	83 μW/cm^2	[16]
Zero transconductance	1 V	2 nA	1 μm × 55 μm	3.6 mW/cm^2	[17]
Zero transconductance	1 V	0.1 nA	1 μm × 1 μm	10 mW/cm^2	[18]
Zero transconductance	10 V	> 3 nA	100 μm × 1,000 μm	30 μW/cm^2	[19]
**Zero transconductance**	**4 V**	**0.004 nA**	500 μm × 1,000 μm	**0.0032 μW/cm^2**	**This work**

Also, we agree with the Reviewer that power consumption and performance are inherently coupled in transistor design. In the T-HTR, the insertion of a CsF injection barrier inherently lowers the on-current, thereby reducing the attainable switching speed. To analyze this trade-off, we examined transfer characteristics systematically as a function of CsF thickness and evaluated the relationship between switching speed and power consumption. In the on-state, the unity-gain cut-off frequency[R6] is

$$f_{\mathrm{T}} = \frac{g_m}{2\pi C_{\mathrm{gg}}}$$

where g_{m} is the transconductance and C_{GG} is the total gate capacitance. g_{m} was obtained from transfer characteristics of the T-HTR (**Fig. R20a, b**). C_{GG} was estimated from the measured gate dielectric unit-area capacitance (C_{i}) (**Fig. R18**) multiplied by the summation of channel area ($W \times L$) and the gate–source/drain overlap area ($W \times [L_{\mathrm{ov,S}} + L_{\mathrm{ov,D}}]$) of the T-HTR (**Fig. R19**), which usually gives a capacitance far larger than the effective value in operation, and thus provides a conservative (lower-bound) estimate of the cut-off frequency. On the other hand, power consumption was calculated from the product of the driving voltage and the current density. Through this analysis, we confirmed a clear trade-off between switching speed and power consumption in our devices. As the CsF barrier thickness increases, the on-current decreases, leading to a reduction in the cut-off frequency from the 10^2–10^3 Hz range to below 1 Hz. At the same time, the static power consumption drops from the mW cm^{-2} scale to the sub-μW cm^{-2} scale (**Fig. R20c**). This clearly demonstrates that barrier engineering enables ultralow power operation, albeit at the expense of switching speed.

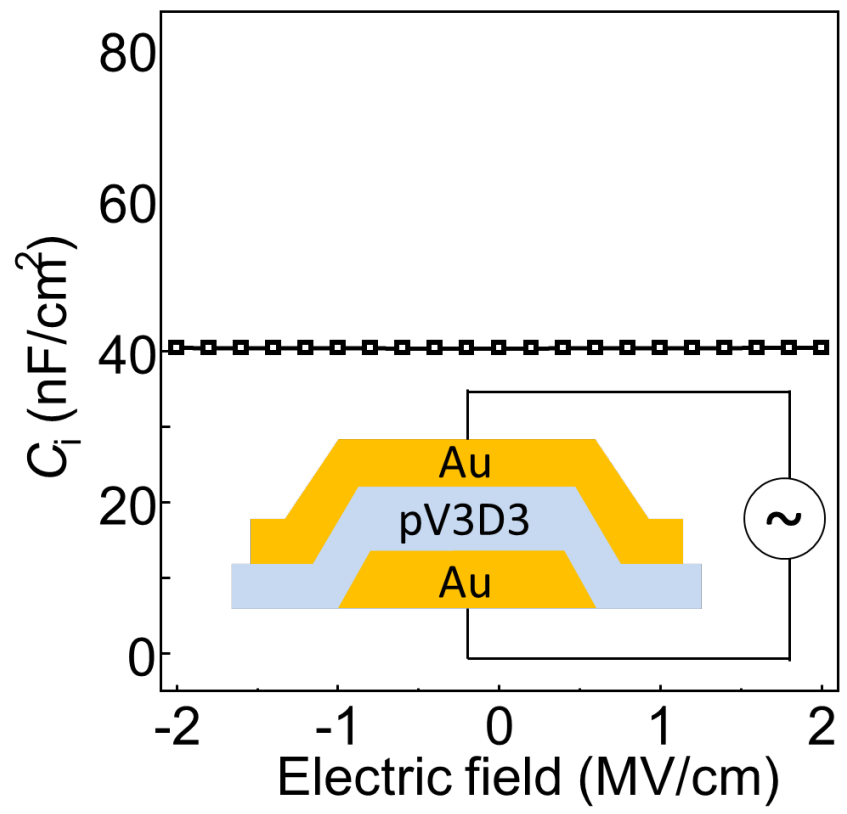

Figure R18. Capacitance per unit area (C_i)–electric field (E_i) of Au/poly(1,3,5-trivinyl-1,3,5-trimethyl cyclotrisiloxane) (pV3D3)/Au device.

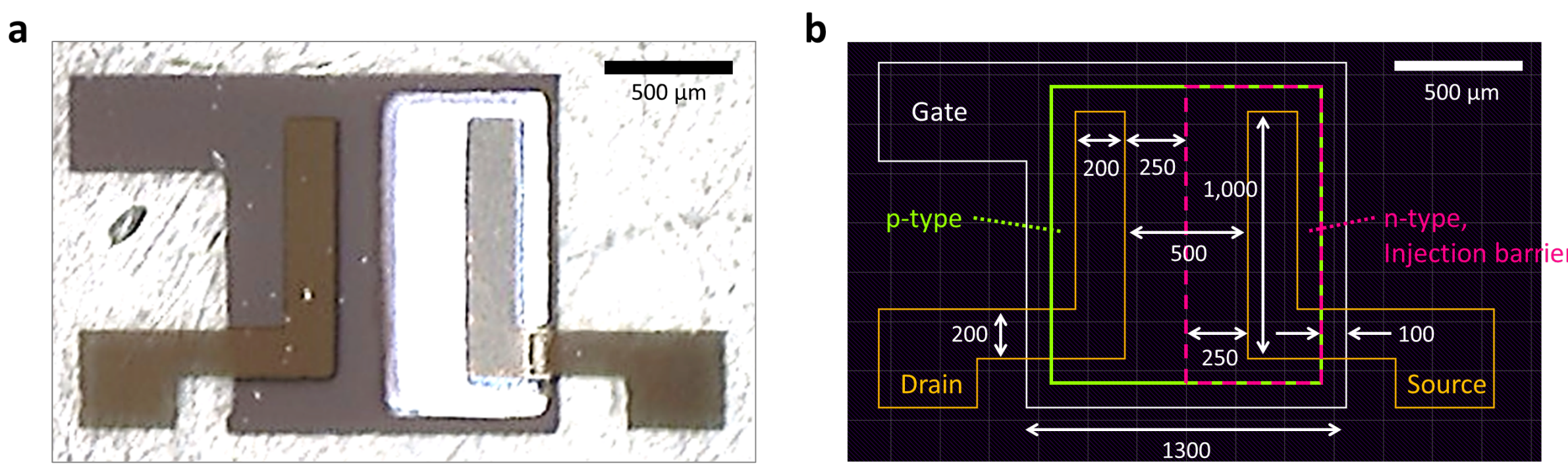

Figure R19. Dimensions of the T-HTR. **a.** An optical microscopy image (scale bar: 500 μm) and **b.** Shadow mask layout of the T-HTR (scale bar: 500 μm).

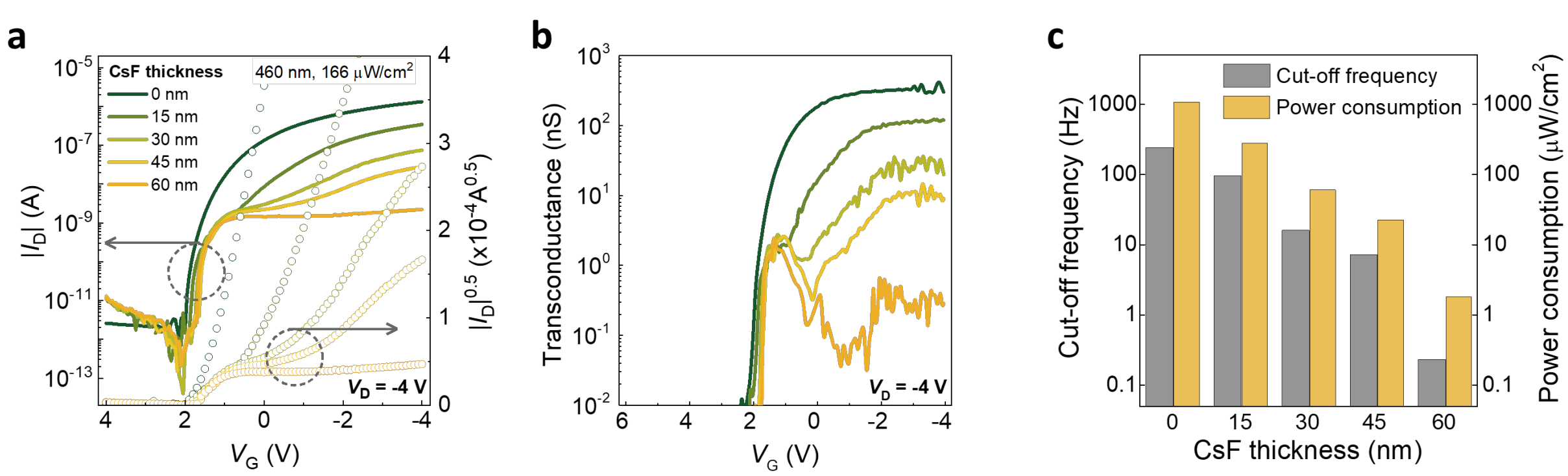

Figure R20. a. The transfer characteristics, **b.** transconductance (g_m), and **c.** cut-off frequency versus static power consumption of the T-HTR as a function of CsF barrier thickness. Increasing the CsF thickness reduces both the cut-off frequency and power consumption, illustrating the

speed-power trade-off governed by injection barrier engineering.

Rather than a drawback, this trade-off is highly relevant for application domains where ultralow static power is prioritized over high clock speed. In many always-on sensors, wearable biomedical monitors, and distributed environmental sensor nodes, the required operating frequencies typically lie only in the Hz–kHz regime, while the overriding design constraint is to minimize static power into the μW or even nW–pW range for long-term autonomous operation [R7], [R8], [R9], [R10]. This consensus has been consistently highlighted in the literature across wearable systems, energy-harvesting wireless sensor networks, and many other always-on sensing platforms[R11], [R12], [R13], [R14]. Moreover, our approach is particularly well suited for low-power applications where minimizing both energy consumption and operation steps is critical, as the use of ternary logic reduces the number of computation steps to approximately log2/log3 ≈ 63.1 % compared to binary logic[R15]. This highlights that barrier engineering in T-HTRs provides a practical pathway toward energy-efficient, multi-valued logic systems.

Also, we emphasize that the present device was fabricated from thermally evaporated organic semiconductors patterned with shadow masks. This simple process is highly suitable for proof-of-concept demonstrations of heterojunction transistors, but it inevitably yields devices with relatively large dimensions, with critical lengths on the order of several hundred micrometers. By employing high-resolution patterning techniques such as standard photolithography, which allows scaling down to a few micrometers, the gate-to-source/drain overlap could be drastically reduced, thereby minimizing parasitic capacitances. In such a scenario, C_{GG} would decrease by approximately four orders of magnitude, leading to a corresponding ~10^4–fold improvement in the cut-off frequency, potentially reaching the hundreds of kHz range. Notably, this improvement arises purely from the reduction of parasitic capacitances, which does not directly affect energy consumption–although a lower parasitic capacitance can further reduce the overall switching energy. Moreover, the approach demonstrated in this work is mainly based on the band engineering through injection barrier design, and is therefore not limited to a particular semiconductor class. This implies that, with high-mobility semiconductors such as oxides or two-dimensional materials, the attainable operation speed could further be enhanced into the MHz regime.

In the revised manuscript, we have included this discussion to clarify the implications

of the current–frequency trade-off and to contextualize the relevance of our device concept for low-power applications.

[R6] Superlattices and Microstructures, **90**, 191-197 (2016).
[R7] *IEEE journal of biomedical and health informatics*, **20**(5), 1251-1264 (2016).
[R8] *Sensors*, **21**(2), 548 (2021).
[R9] Nano-Micro Letters, **16**(1), 52 (2024).
[R10] *IEEE Sensors Journal*, **23**(15), 16467-16480 (2023).
[R11] *Advanced Functional Materials*, **27**(15), 1605271 (2017).
[R12] The innovation, **4**(5), 100485 (2023).
[R13] *IEEE Sensors Journal*, **19**(20), 9047-9062 (2019).
[R14] Energy & Environmental Science, **14**(4), 2114-2157 (2021).
[R15] *IEEE Transactions on computers*, **100**, 1160-1179 (1984).

The revised parts are as follows;

(1) Revised the main text in the revised manuscript (p. 2)

This enables the device to be maintained in the off-state in the absence of intermediate logic operations, thereby minimizing static power consumption to as low as ~3.2 pW/cm^2.

(2) Revised the main text in the revised manuscript (p. 9)

A poly(1,3,5-trimethyl-1,3,5-trivinyl cyclotrisiloxane) (pV3D3, 50 nm) layer deposited in vapor-phase via initiated chemical vapor deposition (iCVD) was utilized as the gate dielectric layer[41]. The dielectric constant of the pV3D3 film was determined to be approximately 2.2, from which the dielectric thickness in the T-HTR was determined to be 50 nm (**Supplementary Fig. 2**).

(2) Revised the main text in the revised manuscript (p. 15).

The influence of the injection barrier thickness on the intermediate state was investigated (**Fig. 2e**). As the CsF thickness increased from 15 nm to 60 nm, the transition into the intermediate state became more distinct. The range of the

intermediate state – defined as the V_G window between the initial V_G, where transconductance started to decrease from its peak, and the end V_G, where transconductance increased back to a level comparable to the initial peak value – broadened from less than 1 V for 15 nm-thick CsF to more than 4 V for 60 nm-thick CsF (**Fig. 2f**). Such expansion resulted from the progressively restricted effective charge injection from the source with increasing barrier thickness, which shifted the onset voltage of field-effect current to higher V_G.

To further elucidate how the injection barrier thickness affects the device figure of merit, we quantitatively analyzed its influence on the operating speed and power consumption (**Supplementary Fig. 14**). As the CsF thickness increased, the on-current gradually decreases due to the restricted charge injection from the source. The reduction in on-current lowers the transconductance and consequently the unity-gain cut-off frequency ($f_T = g_m/2\pi C_{gg}$ where g_m is the transconductance and C_{GG} is the total gate capacitance)[57], while the static power consumption ($P = I \times V$, where I and V denote the operating current and driving voltage, respectively) decreases proportionally, resulting in a clear trade-off between operating speed and power efficiency. As the CsF thickness increased from 15 nm to 60 nm, the estimated cut-off frequency decreased from 10^2-10^3 Hz to below 1 Hz, whereas the static power density dropped from the mW cm^{-2} scale to the sub-μW cm^{-2} scale.

(3) Added the **Figure R18** to Supplementary Information (p. 3)

(4) Added **Figure R19** and **Figure R20** to the Supplementary Information, together with the corresponding descriptions (p. 19).

As the CsF thickness increased from 15 nm to 60 nm, the estimated f_T decreased from 10^2-10^3 Hz to below 1 Hz, whereas the static power density dropped from the mW cm^{-2} scale to the sub-μW cm^{-2} scale. Such a speed–power compromise is highly relevant for application domains where ultralow static power is prioritized over high operating frequency, including always-on optical sensors, wearable biomedical monitors, and distributed environmental sensor nodes typically operating in the Hz–

kHz regime[1~8]. Furthermore, the ternary-logic framework intrinsically reduces the number of computational steps by ≈ 63 % ($\log_2 / \log_3$)[9], offering additional energy savings.

Although the present proof-of-concept devices employ large shadow-mask-defined geometries, scaling the channel length and minimizing gate–electrode overlap through photolithography would reduce C_{GG} by ~10^4-fold, potentially extending the cut-off frequency into the hundreds-of-kHz range. Also, combined with high-mobility semiconductors such as oxides or 2D materials, the attainable speed could further approach the MHz regime.

(5) Replaced **Supplementary Table 1** with **Table R2** in the Supplementary Information (p. 31).

(6) Added the reference [1-9] in the Supplementary Information.

Reviewer (#3)'s COMMENTS:

In this paper, the authors reported a light-driven ternary heterojunction transistor (T-HTR) enabled by incorporating a vertical p-type/n-type heterojunction structure together with a charge injection barrier at the source electrode of a conventional field-effect transistor. The T-HTR demonstrates three distinct states by tuning V_{GS} and the input light intensity, and relatively good stability. In addition, the authors also demonstrated ternary logic inverter (T-inverter), ternary OR and AND gates, and a prototype pixel-level sensory circuit. This work has some novelty in terms of transistor design, and might be interesting to readers in the research field of ternary computing. However, major revisions are needed before acceptance by *Nature Communications*.

Q1) The authors claim that the T-HTR has static power consumption as low as ~ 15 pW by multiplexing the lowest current of 3.8 pA by 4 V at V_{G} = 0 V. However, what's the distribution of the V_{TH}? If V_{TH} shifts, the static power consumption could be much larger. In addition, in a daytime environment without any encapsulation, the light intensity can be 100 mW/cm^2, corresponding to a much larger static power consumption. On the other hand, if encapsulation is adopted to block environment light, how to use light to achieve a ternary transistor operation considering practical applications?

Q4) The design of T-HTR has a big problem for integration. How to make sure that the desired T-HTRs are illuminated in a circuit?

Response:

We thank the Reviewer for raising this important point. Let us respond to the comment 1 and 4 simultaneously.

To address this concern, we measured the transfer characteristics of 12 individual T-HTRs under dark conditions (**Fig. R21a**). The transfer curves of these devices exhibited nearly identical shapes and consistently showed threshold voltages (V_{TH}) above 0 V, indicating high device-to-device uniformity. The extracted V_{TH} values were tightly distributed around -2.26 ± 0.21 V, confirming that the static power consumption is not significantly affected by the threshold variation (**Fig. R21b**).

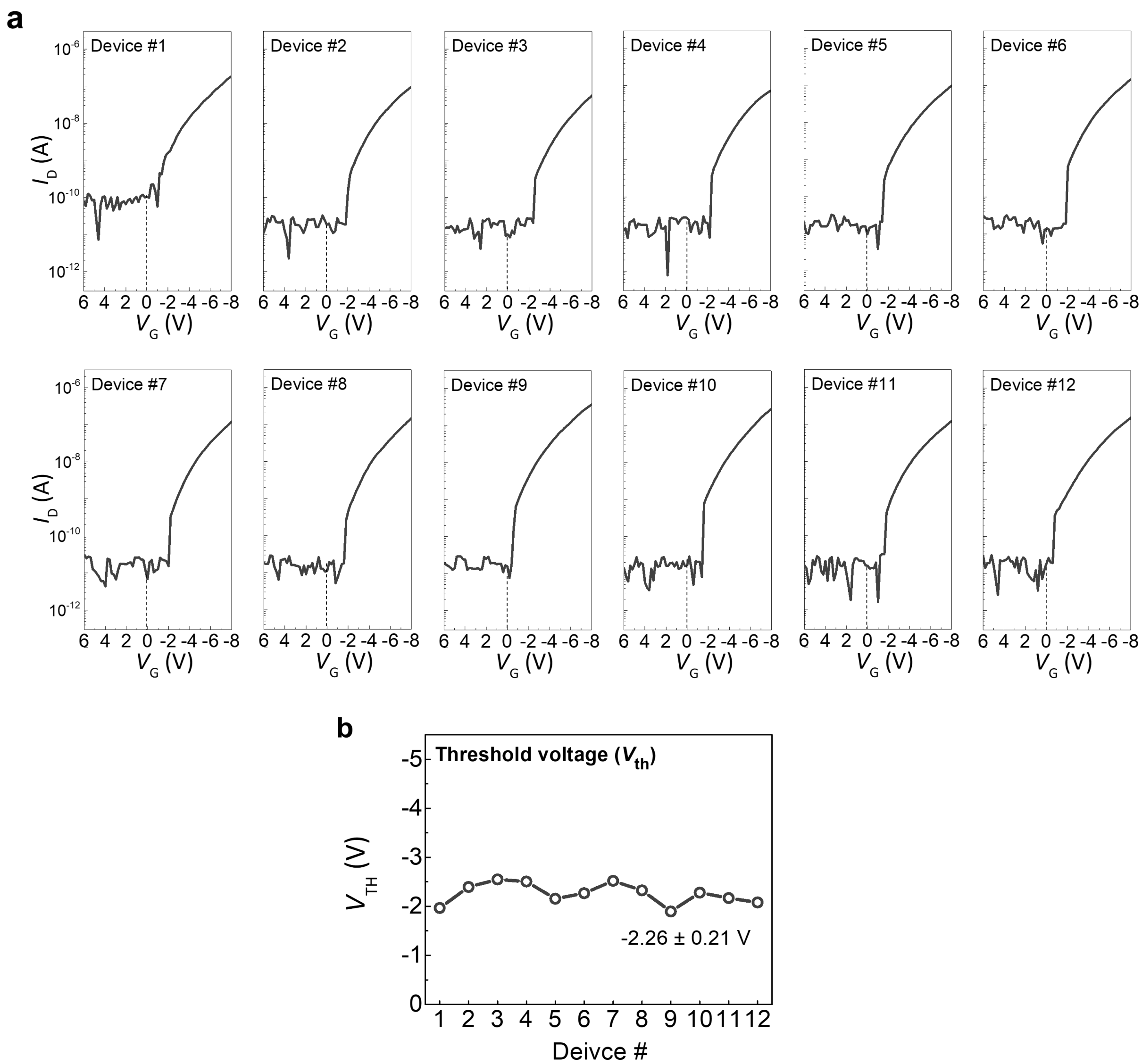

Figure R21. Device-to-device uniformity of the T-HTR. **a**. Transfer characteristics of 12 T-HTRs under dark conditions. **b**. Extracted V_{TH} of the T-HTRs.

Regarding the operation under ambient light and circuit integration, our target use is controlled illumination rather than uncontrolled daylight. In practice we suggest two complementary measures, as shown below.

1) Packaging/optical isolation: employ an opaque encapsulation (black matrix and metal light shields) with defined optical windows above selected cells. We can also use band-pass or neutral-density (ND) filters matched to the T-HTR absorption. This setup can keep non-addressed devices in the dark, and retain pW-level standby power.

2) On-panel light sources: drive only the intended T-HTRs using integrated micro-LEDs (or other narrowband emitters) placed per cell or per logic block. The emitters are pulsed or duty-cycled, so the average optical and electrical power remains low. Background light can also be suppressed with tamper detection if needed.

With these approaches, only the addressed T-HTRs are illuminated while the rest of the circuit remains in the dark. This directly answers the integration question: illumination selectivity can be achieved by pixelated emitters as well as local apertures/shields, which are well-established techniques from display backplanes and optical sensor modules[R32], [R33], [R34]. Therefore, low dark static power can be reproduced reliably across the devices, and practical operation mainly relies on localized, controllable illumination or filtered spectral bands to activate the desired ternary state without sacrificing circuit-level power or robustness.

We have revised the manuscript to include device-to-device V_{TH} distribution data and to discuss practical circuit-level strategies such as optical isolation and on-panel light sources for selective illumination.

(1) Revised the main text in the revised manuscript (p. 13)

This also enabled the device to be maintained in the off-state in the absence of intermediate logic operations, thereby minimizing static power consumption to as low as ~3.2 pW/cm^2, which is significantly lower than that of conventional ternary logic transistors (**Supplementary Table 1**). Such low static power consumption at the intermediate state was reproducibly obtained across 12 devices, with the dark-state V_{TH} tightly distributed around -2.26 ± 0.21 V (**Supplementary Fig. 11**).

(2) Revised the main text in the revised manuscript (p. 3)

A fundamental strategy in optoelectronic logic circuits involves modulating the field-effect characteristics of semiconductors using light, thereby diversifying the switching behavior of individual transistor units. Such optical modulation is typically achieved under controlled illumination rather than ambient light, using localized or integrated light sources to ensure stable and selective device operation. This approach facilitates light-based information processing and flexible logic execution within a minimal circuit

footprint and thus alleviating the necessity for complex circuit architectures or large-scale hardware configurations.

(3) Added the **Figure R21** to Supplementary Information (p. 15)

[R32] *Microelectronics journal*, **37**(5), 433-451 (2006).
[R33] *Applied Physics Letters*, 125(15), 150504 (2024).
[R34] *Advanced Materials*, 34(12), 2108979 (2022).

Q2) The authors analyze the mechanism of T-HTR in Fig. 1g and Fig. 3c. However, I am confused why the CsF or MoO_X coating could change the work function of Au. For their UPS study, they measured the work-functions of film stacks. However, it cannot be directly related to the work-function of Au. In addition, the authors should analyze the working mechanism more with various V_{GS} and V_{DS} values using band diagrams, and with reversed source/drain orientation to better illustrate the design principles.

Response:

We thank the Reviewer for this insightful comment. We agree that ultraviolet photoelectron spectroscopy (UPS) measurements on multilayer film stacks cannot be interpreted as the bare Au work function itself. Rather than that, the measured shift reflects the effective surface potential at the Au/interlayer interface. Therefore, in Fig. 1g and Fig. 3c, our intention was not to claim that the intrinsic work function of Au was modified, but rather to show that the introduction of CsF or MoO_x layers induces strong interfacial dipoles, leading to an apparent shift in the effective work function of the electrode stack. This interpretation is consistent with many prior studies on alkali halide and transition metal oxide interlayers (Refs. 34–39, 50–52 in manuscript).

To address the Reviewer's concern regarding the apparent change of Au work function and, in line with the Reviewer's suggestion to analyze the device operation under different gate voltage (V_G), drain voltage (V_D) conditions as well as with reversed source/drain orientation, we expanded our band structure analysis for the p-type T-HTR in a more rigorous and comprehensive manner. The optical bandgaps of C10-DNTT and C13-PTCDI were obtained from UV–vis absorption spectra and Tauc plots (**Fig. R22a, b**). The ionization energies were

extracted from the UPS spectra of C10-DNTT and C13-PTCDI thin films (**Fig. R23a, b**). By combining the optical bandgaps with the UPS-derived ionization energies, we calculated the electron affinity values for both p- and n-type semiconductors. A summary of the resulting energy levels is provided in **Table R3**.

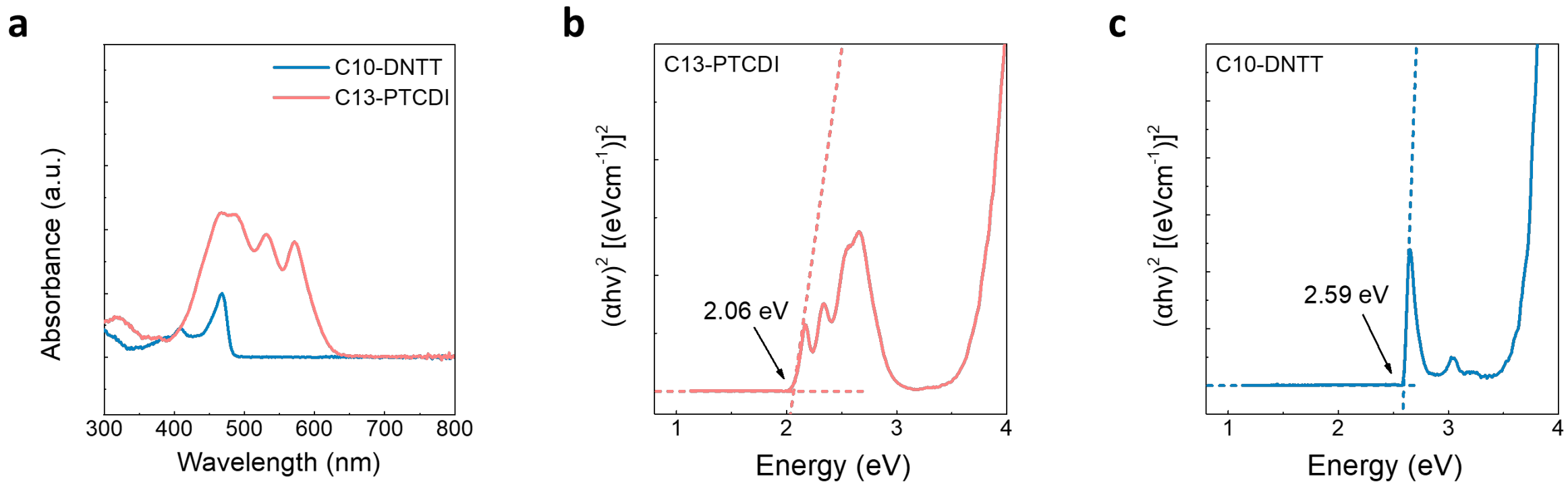

Figure R22. a. UV–vis absorption spectra and **b**. corresponding Tauc plots of C10-DNTT and C13-PTCDI thin films, used to extract the optical bandgap values.

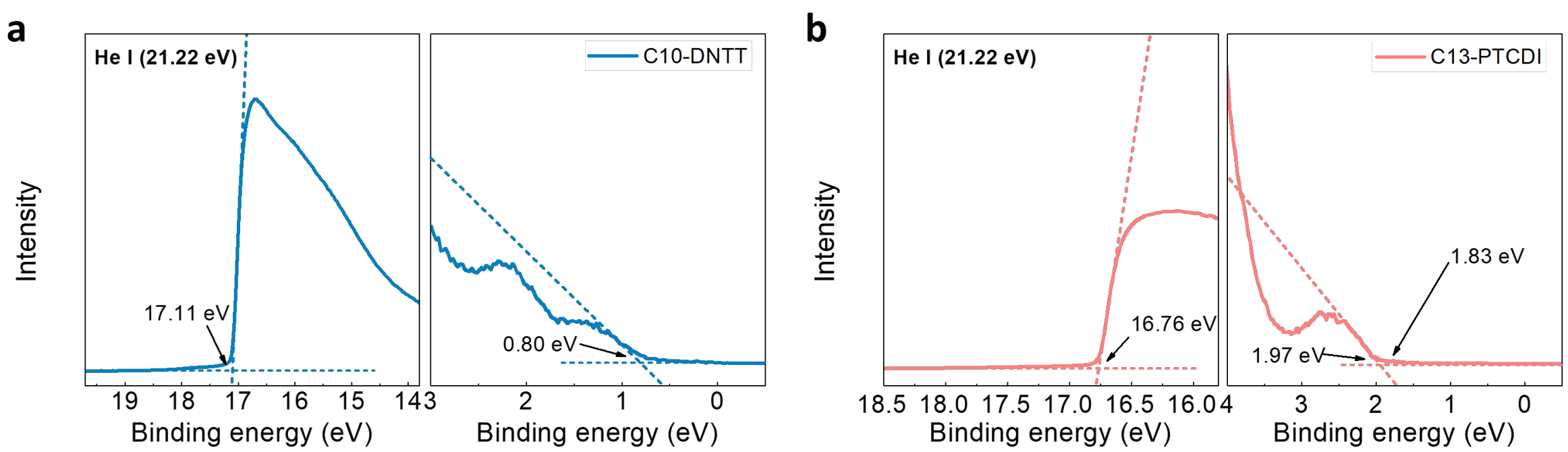

Figure R23. a. UPS spectra of C10-DNTT and **b**. C13-PTCDI thin films.

Table R3. Parameters with MoO_X (on C10-DNTT) and CsF (on C13-PTCDI) interlayers.

Materials	Optical bandgap (eV)	Work function (eV)	HOMO (eV)	LUMO (eV)
C10-DNTT	2.59	4.12	4.92	2.33
C13-PTCDI	2.06	4.45	6.42	4.36

Using these values as input boundary conditions, we carried out electrostatic calculations to quantitatively evaluate the source-side band alignment under dark conditions.

Figs. R24 and R25 present the resulting energy band diagrams at three representative gate bias states: V_G = 2 V (OFF), -1 V (intermediate), and -8 V (ON), and the reversed source/drain orientation for p-type and n-type T-HTR, respectively. In both figures, the two organic layers (C13-PTCDI and C10-DNTT) were treated as dielectric segments due to the limitation of the band diagram program (Boise State University, GitHub: BoiseState/Multi-Dielectric).

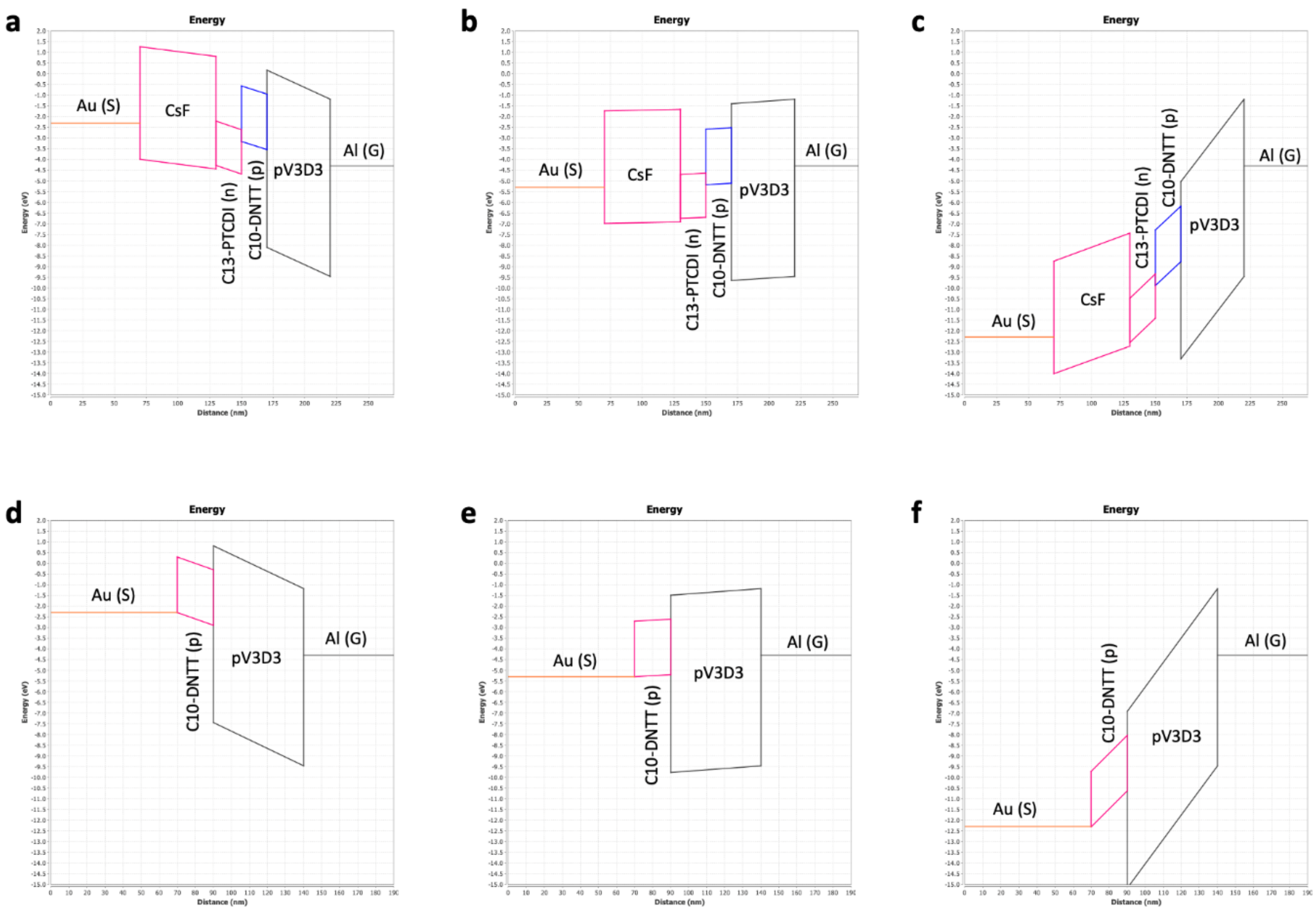

Figure R24. Schematic energy band diagrams of the p-type T-HTRs at the source-side, constructed based on experimental boundary conditions at three representative V_G states: **a.** V_G = 2 V (OFF), **b.** -1 V (intermediate), and **c.** -8 V (ON). Corresponding diagrams for the reversed source/drain configuration are shown in **d-f**.

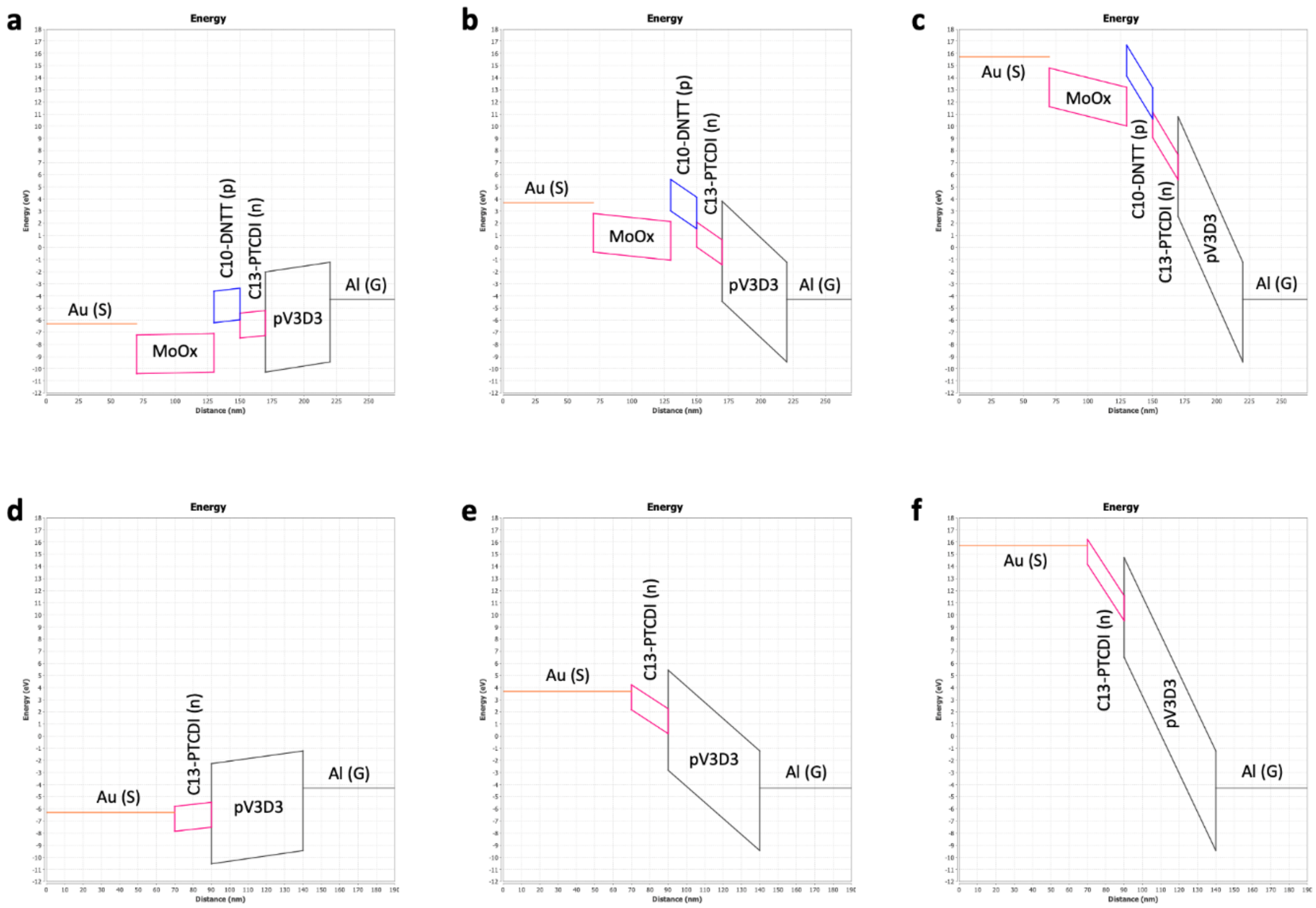

Figure R25. Schematic energy band diagrams of the n-type T-HTRs at the source-side, constructed based on experimental boundary conditions at three representative V_G states: **a.** V_G = -2 V (OFF), **b**. 8 V (intermediate), and **c**. 20 V (ON). Corresponding diagrams for the reversed source/drain configuration are shown in **d-f**.

Here, along the vertical cross-section of the source-side stack, the applied voltage distributes across each layer according to the serial-capacitor relation:

$$C_\mathrm{i} = \frac{\varepsilon_0 \varepsilon_\mathrm{r,i}}{t_\mathrm{i}}, \qquad \Delta V_\mathrm{i} = \frac{t_\mathrm{i}/\varepsilon_\mathrm{r,i}}{\sum (t_\mathrm{l}/\varepsilon_\mathrm{r,j})} \times V_\mathrm{G}, \qquad E_\mathrm{i} = \frac{\Delta V_\mathrm{i}}{t_\mathrm{i}} = \frac{1/\varepsilon_\mathrm{r,i}}{\sum (t_\mathrm{j}/\varepsilon_\mathrm{r,j})} \times V_\mathrm{G}$$

where t_i is the thickness of the i-th dielectric layer, $\varepsilon_\mathrm{r,i}$ is its relative permittivity, ε_0 is the vacuum permittivity, ΔV_i is the voltage drop across i-th layer, and E_i is the corresponding electric field. Using the parameters of our structure [poly(1,3,5-trivinyl-1,3,5-trimethyl cyclotrisiloxane) (pV3D3): t = 50 nm, ε_r= 2.2 (**Fig. R26**); C10-DNTT and C13-PTCDI: t = 20 nm, ε_r= 3 for each[R19, R20]; CsF: t = 60 nm, ε_r= 7.9[R21]; MoOx: t = 60 nm, ε_r= 20[R22]], the baseline voltage drop is estimated to be approximately 52 % across pV3D3, 31 % across the

organic heterojunction, and 17 % across CsF for the p-type T-HTR. For the n-type T-HTR, the corresponding voltage drops were calculated to be approximately 58% across pV3D3, 34% across the organic heterojunction, and 8% across MoO_X.

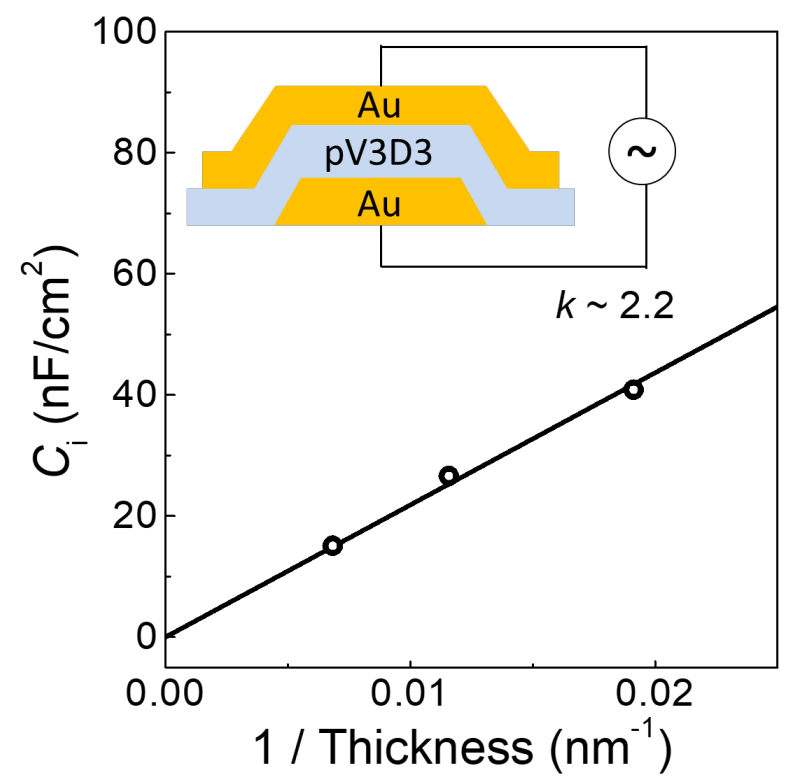

Figure R26. Capacitance per unit area (C_i) – inverse dielectric thickness ($1/d$) of Au/pV3D3/Au device.

Under illumination, photogenerated charge carriers are trapped at the channel/injection barrier layers, leading to an increase in the interfacial charge density with irradiance. For the p-type T-HTR, photogenerated electrons are trapped at the C13-PTCDI/CsF interface (or the C10-DNTT/Au interface). For the n-type T-HTR, photogenerated holes are trapped at the C10-DNTT/MoO_X interface (or the C13-PTCDI/Au interface). The trapped charge (Q_{it}) can be described by the relation:

$$Q_{it} \simeq C_{ox} \times \Delta V_{TH}$$

where C_{ox} is the gate dielectric capacitance per unit area and ΔV_{TH} is the illumination-induced threshold voltage shift. This trapped charge corresponds to an interfacial surface charge density, $\sigma = Q_{it}/area$, which enhances the internal electric field of CsF layer in the p-type device (or the MoO_X layer in the n-type device), depending on the polarity of the trapped carriers.

$$\Delta E = \frac{\sigma}{\varepsilon_0 \varepsilon_r}$$

For the p-type T-HTR, because the negative surface charge is located at the C13-PTCDI/CsF

interface, Gauss's law redistributes the potential drop across that series stack at a fixed V_G, resulting in an increased field ($|E|$) in CsF (steeper band tilt), while the upstream pV3D3 is partially screened (flatter band tilt).

As the density of trapped electrons increases, the strengthened internal field in CsF under illumination directly impacts the injection mechanism at the source side, which is governed by trap-assisted hopping conduction described by the Poole-Frenkel (PF) mechanism, expressed as:

$$J \sim E \times \exp\left[-\frac{e\left(\emptyset_B - \sqrt{\frac{eE}{\pi\varepsilon_i}}\right)}{kT}\right] \propto E \times \exp\left[\sqrt{E}\right]$$

where, J is the current density, E is the electric field, $\emptyset_B$ is the potential barrier height, e is the elementary charge, ε_i is the dielectric permittivity, k is Boltzmann's constant and T is the temperature. This interpretation is supported by our vertical-stack control devices [Au/C10-DNTT (20 nm)/C13-PTCDI (20 nm)/CsF (0 to 90 nm)/Au], in which the current density systematically decreases with increasing CsF thickness, while the experimental data exhibited excellent linearity in Poole-Frenkel plots [ln(J_i/E_i)–sqrt(E_i)] (**Fig. R27**). For the n-type T-HTR, the same principle applies, where positive surface charges trapped at the C10-DNTT/MoO_X interface enhance the internal field in MoO_X, thereby facilitating hole injection through a Poole-Frenkel trap-assisted transport mechanism, as established in prior studies[R35], [R36].

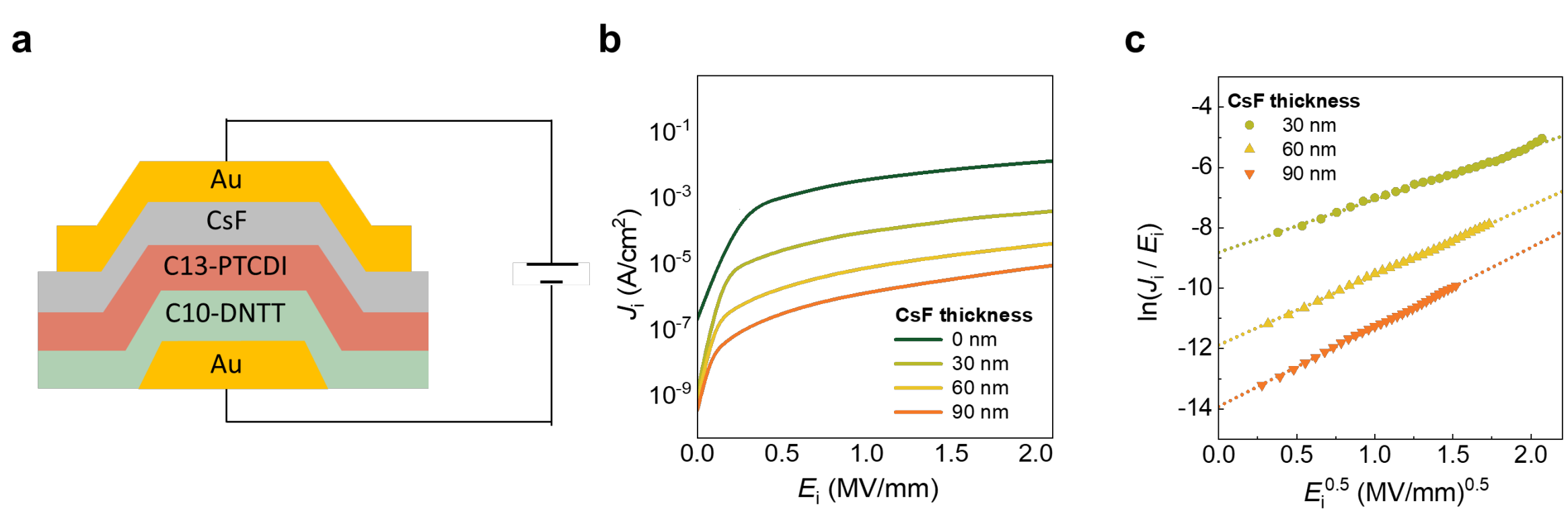

Figure R27. a. Schematic illustration of the Au/C10-DNTT (20 nm)/C13-PTCDI (20 nm)/CsF (0 to 90 nm)/Au vertical stack structure used to emulate the source-side configuration of the

T-HTR. **b**. Current density–electric field($J_i - E_i$) characteristics of the vertical devices with different CsF thicknesses, showing a systematic reduction of current with increasing barrier thickness. **c**. Poole-Frenkel plots derived from the $J_i - E_i$ data, exhibiting linear behavior consistent with trap-assisted Poole-Frenkel conduction.

The accumulation of interfacial charge therefore leads to a stronger internal field in CsF, which promotes multistep trap-assisted transport across CsF layer, resulting in an enhancement of the current. Simultaneously, the negative charge at the interface photogates the p-type C10-DNTT channel and redistributes the applied potential, such that a larger portion drops across CsF. This mechanism is qualitatively reflected in the schematic band diagrams for the intermediate state and ON states, as shown by solid lines in **Fig. R28**.

For the n-type T-HTR, a similar process occurs with positive interfacial charge accumulated at the C10-DNTT/MoO_X interface, which strengthens the internal field in MoO_X and facilitates hole injection into the C13-PTCDI layer under illumination. The corresponding band evolution is illustrated in **Fig. R29**.

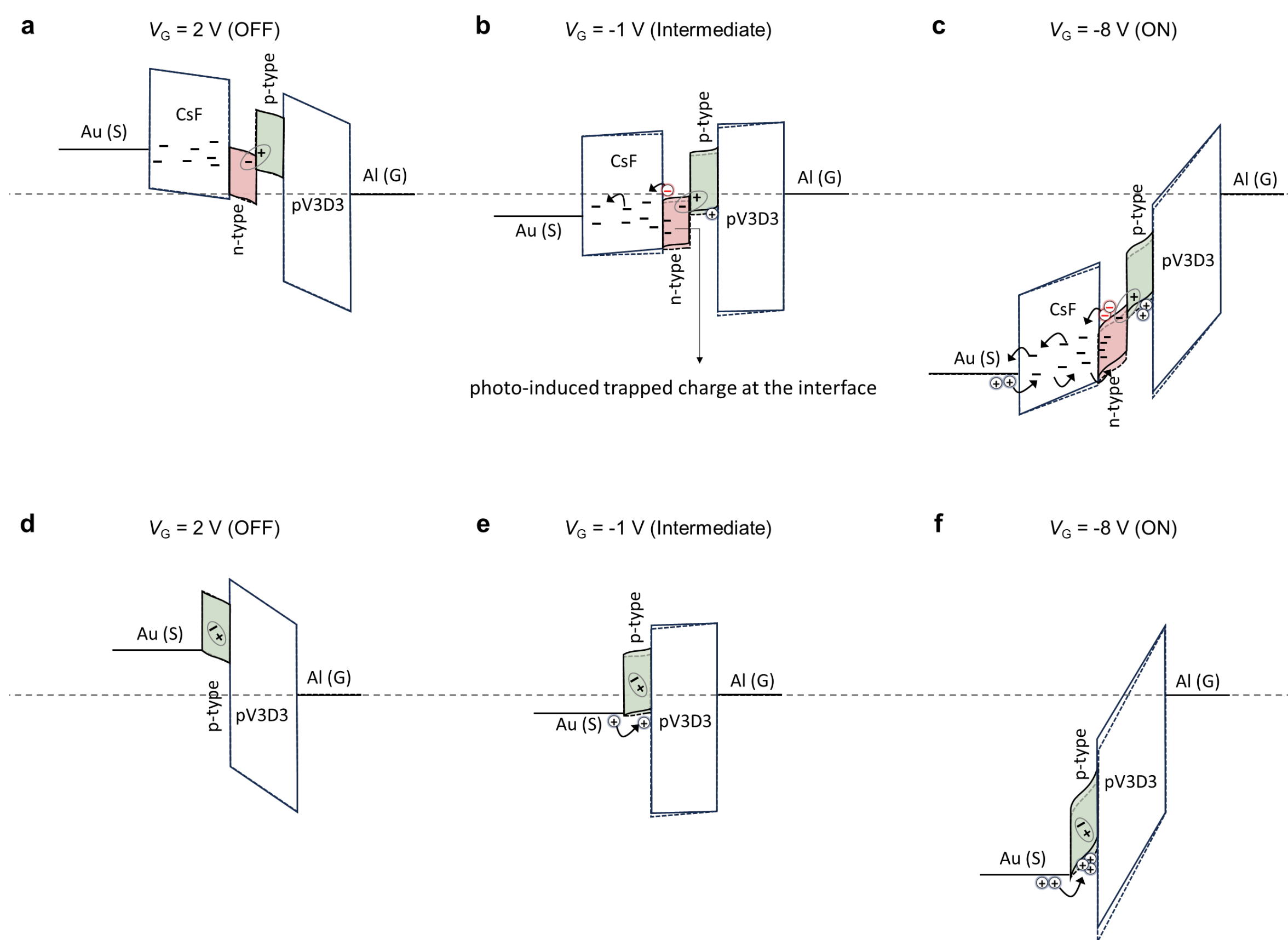

Figure R28. Schematic energy band diagrams of the p-type T-HTRs at the source-side, in the dark (dashed lines) and under illumination (solid lines) at three representative V_G states: **a**. V_G = 2 V (OFF), **b**. -1 V (intermediate), and **c**. -8 V (ON). Corresponding diagrams for the reversed source/drain configuration are shown in **d-f**.

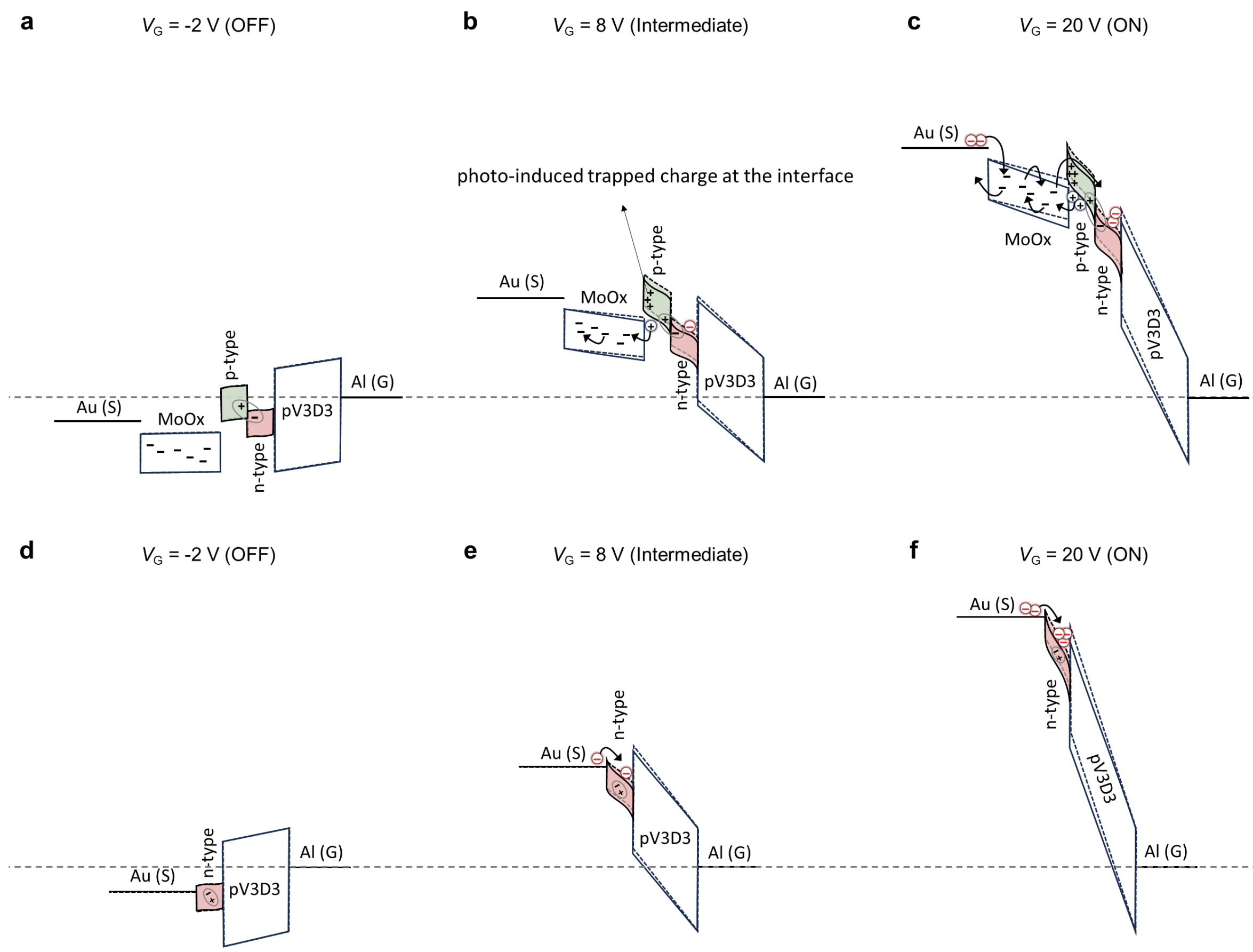

Figure R29. Schematic energy band diagrams of the n-type T-HTR at the source-side stack, in the dark (dashed lines) and under illumination (solid lines) at three representative V_G states: **a**. V_G = -2 V (OFF), **b**. 8 V (intermediate), and **c**. 20 V (ON). Corresponding diagrams for the reversed source/drain configuration are shown in **d-f**.

The effect of drain bias (V_D) also influences the source-side band. At a small $|V_D|$ (e.g., -1 V for the p-type and +2 V for the n-type), the potential drop along the channel is minor, so the field across CsF (p-type) or MoO_X (n-type) is mainly governed by the gate bias. In this regime, trap-assisted conduction dominates with a relatively small field enhancement.

As $|V_D|$ increases (e.g., -4 V for the p-type and +10 V for the n-type), part of the drain

potential also drops across the source-side dielectric stack, effectively increasing the internal field in CsF or MoO_X, respectively. This enhanced field strengthens Poole-Frenkel conduction by lowering the effective barrier ($\emptyset_B - \sqrt{eE_{eff}/\pi\varepsilon_i}$), resulting in an increase of current ($J \propto E_{eff} \times \exp[\sqrt{E_{eff}}]$). Here, E_{eff} represents the combined field composed of the gate-induced component and the additional field induced from the drain bias and photogenerated interfacial charge accumulation.

We revised the manuscript to better emphasize these design principles and provide the corresponding band diagrams and discussion in the Results section.

[R19] *Journal of Materials Chemistry C*, **10**(2), 726-733 (2022).
[R20] *MRS Online Proceedings Library*, **1091**, 1091-AA05 (2008).
[R21] *Physical Review*, **168**(3), 1054 (1968).
[R22] *Advanced Electronic Materials*, **6**(10), 2000635 (2020).
[R35] *Thermochimica Acta*, **54**(1-2), 35-45 (1982).
[R36] *Journal of Applied Physics*, **124**(8) (2018).

(1) Revised the main text in the revised manuscript (p. 9)

To investigate the energy level alignment at the source electrode of the T-HTR, ultraviolet photoelectron spectroscopy (UPS) was conducted on Au with thermally deposited CsF as a function of CsF thickness (**Fig. 1f**). The apparent work function of the Au surface decreased from 4.9 eV to 3.8 eV as the CsF thickness increased from 0 nm to 10 nm. Notably, the most pronounced shift occurred within the first nanometer, corresponding to a drop from 4.9 eV to approximately 4.1 eV, followed by a gradual saturation at larger thicknesses. This reduction reflects the formation of strong interfacial dipoles at the buried Au/CsF interface that modify the electrostatic potential across the metal/dipole stack and effectively lower the vacuum level at the outer CsF surface. Such dipole-induced shifts have been widely reported for alkali halide interlayers and arise from partial charge transfer and polarization at the metal/insulator interface. The subsequent saturation behavior is consistent with the cancellation of densely packed dipoles and the attainment of an electrostatic equilibrium at the surface[45,46].

(2) Revised the main text in the revised manuscript (p. 10)

In this study, a 60 nm-thick CsF layer was employed to enhance the injection barrier functionality beyond dipole formation. To elucidate the charge-transport mechanism through this thick CsF layer, we fabricated vertical Au/C10-DNTT (20 nm)/C13-PTCDI (20 nm)/CsF (0 to 90 nm)/Au stacks that replicate the source-side configuration of the T-HTR. The current density (J_i)–electric field (E_i) characteristics of this stacked device showed a systematic decrease in J_i with increasing CsF thickness, confirming that a thicker barrier effectively suppresses carrier transmission (**Supplementary Fig. 4**). Notably, when the experimental data were analyzed in Poole-Frenkel coordinates [$\ln(J_\mathrm{i}/E_\mathrm{i})$ versus $\sqrt{E_\mathrm{i}}$][47,48], excellent linearity was obtained, indicating that carrier transport is dominated by trap-assisted Poole-Frenkel conduction. In this mechanism, a thicker CsF layer reduces the average electric field and extends the trap-mediated path, thereby lowering the overall transmission probability. To further quantify the semiconductor energy levels used for band alignment, UPS analysis was also performed on the organic semiconductors. The ionization energies extracted from the UPS spectra were 4.92 eV for C10-DNTT and 6.42 eV for C13-PTCDI (**Supplementary Fig. 5**). Subsequently, the optical bandgaps were obtained from UV-vis absorption measurements using Tauc analysis (**Supplementary Fig. 6**), yielding values of 2.59 eV and 2.06 eV for C10-DNTT and C13-PTCDI, respectively. By combining the UPS-derived ionization energies with the optical bandgaps, the electron affinities were determined to be 2.33 eV for C10-DNTT and 4.36 eV for C13-PTCDI.

The energy band diagram of the T-HTR from gate to source illustrates how the thick CsF layer governs the source-contact behavior in the light-induced intermediate state (**Fig. 1g**). Under illumination, exciton dissociation at the p-n heterojunction generates minority electrons that drift toward the CsF side. In this configuration, both hole injection from the source and light-induced minority-electron transport from the n-type layer are available through the trap-assisted Poole-Frenkel process, but the band alignment makes electron transport energetically favorable while rendering hole injection highly unfavorable: the apparent work function of the Au/CsF contact (~3.8

eV) places its Fermi level above the lowest unoccupied molecular orbital (LUMO) level of C13-PTCDI (2.06 eV), whereas the much deeper highest occupied molecular orbital (HOMO) level of the C10-DNTT (4.92 eV) imposes a large barrier. Notably, photogenerated holes can still be transported through the p-type channel toward the drain, contributing to photocurrent, even though gate-induced hole injection from the source remains energetically suppressed. Consequently, field-effect current remains suppressed, whereas photocurrent can readily flow through the trap-assisted pathway, giving rise to the stable intermediate conduction state. Meanwhile, a fraction of minority electrons that drift toward the CsF side is likely captured by interfacial states at the C13-PTCDI/CsF junction. This establishes a photo-induced dipole and alters the potential distribution across the series dielectric stack at a fixed V_{G} via Gauss's law, increasing the electric field in the CsF layer (steeper band slope) while partially screening the upstream pV3D3 layer (flatter band slope). This enhanced electric field in CsF lowers the effective barrier for trap-assisted Poole-Frenkel conduction, leading to large threshold voltage (V_{TH}) shift in devices incorporating a CsF injection barrier under illumination, as will be discussed later.

The band configurations for the off- and on-states are also provided in **Supplementary Fig. 7**, together with the intermediate-state band diagram. In the off state, the channel is fully depleted; even under illumination, photogenerated carriers cannot contribute meaningfully to conduction because they cannot percolate through the depleted channel. In the on state, under strong gate bias, the electric field effectively reduces the hole-injection barrier through CsF and enables efficient hole transport into the p-type channel, with photocurrent further augmenting the total conduction. To clearly illustrate the role of the CsF/n-type/p-type stack at the source side of the T-HTR, band diagrams for devices with reversed source/drain configuration are also provided in **Supplementary Fig. 7** for direct comparison.

(3) Revised the main text in the revised manuscript (p. 19)

To examine the energy level alignment at the source electrode of the T-HTR, UPS was conducted on Au with thermally deposited MoO_X as a function of MoO_X thickness (**Fig.**

3b). As the MoO_X thickness increased from 0 to 10 nm, the apparent work function of the Au progressively increased from 4.9 eV to 5.75 eV, with the steepest rise occurring within the first nanometer, reflecting the formation of strong interfacial dipoles at the Au/MoO_X interface. The band diagram of the T-HTR from gate to source reveals how the MoO_X layer governs the source-contact behavior in the light-induced intermediate state, which is analogous to the CsF-based T-HTR (**Fig. 3c**). Exciton dissociation at the p–n heterojunction generates minority holes that drift toward the MoO_x interface under illumination. Although both photogenerated holes from the p-type semiconductor and electrons from the source are in principle available, the energy alignment strongly favors hole transport while suppressing electron injection: the Au/MoO_X work function (~5.75 eV) aligns closely with the HOMO level of C10-DNTT (4.92 eV), whereas the LUMO level of C13-PTCDI (2.33 eV) imposes a large barrier to electron injection. In addition, similar to the CsF, charge transport through the MoO_X layer proceeds via trap-assisted Poole-Frenkel conduction due to the mixed valence states and oxygen-deficiency defects[65,66]. As a result, gate-induced field-effect current remains suppressed, while photocarriers establish the intermediate state: holes are extracted through the MoO_X/source contact, and electrons are transported along the n-type channel toward the drain. The band configurations for the off- and on-states are also provided in **Supplementary Fig. 16**, together with the intermediate-state band diagram. Also, to clearly illustrate the role of the MoO_X/p-type/n-type stack at the source side of the T-HTR, band diagrams for devices with reversed source/drain configuration are also provided in **Supplementary Fig. 16** for direct comparison.

(4) Replaced Fig. 1g with **Figure R28b** in the revised manuscript (p. 31).

Figure 1 | Design of the ternary heterojunction transistor (T-HTR). a. The schematic illustration and **b**. optical microscopy image of the T-HTR (scale bar: 500 μm). **c**. The schematic illustration of transfer characteristics and **d**. operational mechanism of the T-HTR consisting of three switching states. **e**. Ultraviolet-visible (UV/Vis) absorption spectra of the n-type (C13-PTCDI), p-type (C10-DNTT) and n-type/p-type bilayer organic semiconductors. **f**. Ultraviolet photoelectron spectroscopy (UPS) spectra

of Au contact with thermally deposited cesium fluoride (CsF) as a function of CsF thickness. **g**. Schematic energy band diagrams of the source-side stack in the dark (dashed lines) and under illumination (solid lines). The trapped charges at the CsF/p-type interface enhances the internal electric field in CsF.

(5) Replaced Fig. 3c with **Figure R29b** in the revised manuscript (p. 33).

Figure 3 | Versatility of the T-HTR architecture. a. A schematic illustration of the T-HTR with molybdenum oxide (MoO_X) injection barrier. **b**. UPS spectra of Au contact with thermally deposited MoO_X as a function of MoO_X thickness. **c**. Schematic energy band diagrams of the source-side stack in the dark (dashed lines) and under illumination (solid lines). The trapped charges at the MoO_X/n-type interface enhances the internal electric field in MoO_X. **d**. The transfer characteristics, **e**. g_m, and **f**. I_D at the off-, intermediate, and on-state of the T-HTR under 460 nm illumination with respect to intensity.

(6) Added the **Figure R22** to Supplementary Information (p. 8)

(7) Added the **Figure R23** to Supplementary Information (p. 7)

(8) Added the **Figure R26** to Supplementary Information (p. 3)

(9) Added the **Figure R27** to Supplementary Information (p. 5)

(10) Added the **Figure R28** to Supplementary Information to the Supplementary Information, together with the corresponding descriptions (p. 9).

In the off-state, the channel remains fully depleted, preventing the formation of a conductive pathway; as a result, charge transport is effectively suppressed and photogenerated carriers cannot percolate through the channel, yielding negligible current even under illumination. In the intermediate state, the engineered injection barrier at the source suppresses gate-induced hole injection while allowing photogenerated carriers to flow, thereby producing a gate-voltage-independent

photocurrent plateau with zero transconductance ($g_{\mathrm{m}} \approx 0$). In the on-state, a sufficiently strong V_{G} reduces the source-side hole-injection barrier and enables efficient hole transport through the p-type channel, with the photocurrent providing an additional contribution to the total conduction.

The effect of drain bias (V_{D}) also influences the source-side band. At a small V_{D}, the potential drop along the channel is minor, so the field across CsF is mainly governed by the V_{G}. In this regime, trap-assisted conduction dominates with a relatively small field enhancement. As V_{D} increases, part of the drain potential also drops across the source-side dielectric stack, effectively increasing the internal field in CsF. This enhanced field strengthens Poole–Frenkel conduction by lowering the effective barrier ($\emptyset_{\mathrm{B}} - \sqrt{eE_{\mathrm{eff}}/\pi\varepsilon_i}$), resulting in an increase of current ($J \propto E_{\mathrm{eff}} \times \exp[\sqrt{E_{\mathrm{eff}}}]$). Here, E_{eff} represents the combined field composed of the gate-induced component and the additional field induced from the drain bias and photogenerated interfacial charge accumulation.

When the source and drain are reversed, the source directly contacts the p-type channel without the injection barrier, enabling hole injection and resulting in behavior analogous to that of a conventional transistor without an intermediate state.

(11) Added the **Figure R29** to Supplementary Information to the Supplementary Information, together with the corresponding descriptions (p. 22).

In the off-state, the channel is fully depleted and both electrically and optically generated carriers are unable to form a percolating path, yielding negligible current. In the intermediate state, the molybdenum oxide (MoO_X) injection barrier suppresses gate-induced electron injection from the source while allowing photogenerated holes to be extracted, producing a light-driven, V_{G}-independent current plateau ($g_{\mathrm{m}} \approx 0$). In the on-state, a sufficiently positive gate bias lowers the hole-injection barrier and enables efficient hole transport through the p-type channel, with photocurrent adding to the total conduction.

V_D additionally modulates the MoO_X barrier. At low V_D, the field at the source contact is mainly V_G-controlled. At larger V_D, part of the drain potential drops across the MoO_X/semiconductor stack, increasing the effective field and enhancing trap-assisted Poole–Frenkel conduction, thereby increasing current.

Reversing the source/drain terminals removes the MoO_x barrier at the injecting contact, permitting electron injection and resulting in conventional transistor behavior without an intermediate state.

(12) Added the reference [47-48] and [65-66] in the revised manuscript.

Q3) The T-HTR has very low operation current. For logic operations, clock speed is very important. The authors should give an estimation of how fast their logic circuits can work.

Response:

We thank the Reviewer for raising this important point. We agree that the insertion of the barrier layer inevitably lowers the on-current of the devices, and reduced current can, in principle, limit the attainable operating frequency. To analyze this trade-off, we examined transfer characteristics systematically as a function of CsF thickness and evaluated the relationship between switching speed and power consumption. In the on-state, the unity-gain cut-off frequency[R6] is

$$f_T = \frac{g_m}{2\pi C_{gg}}$$

where g_m is the transconductance and C_{GG} is the total gate capacitance. g_m was obtained from transfer characteristics of the T-HTR (**Fig. R32a, b**). C_{GG} was estimated from the measured gate dielectric unit-area capacitance (C_i) (**Fig. R30**) multiplied by the summation of channel area ($W \times L$) and the gate–source/drain overlap area ($W \times [L_{ov,S} + L_{ov,D}]$) of the T-HTR (**Fig. R31**), which usually gives a capacitance far larger than the effective value in operation, and thus provides a conservative (lower-bound) estimate of the cut-off frequency. On the other hand, power consumption was calculated from the product of the driving voltage

and the current density. Through this analysis, we confirmed a clear trade-off between switching speed and power consumption in our devices. As the CsF barrier thickness increases, the on-current decreases, leading to a reduction in the cut-off frequency from the 10^2–10^3 Hz range to below 1 Hz. At the same time, the static power consumption drops from the mW cm^{-2} scale to the sub-μW cm^{-2} scale (**Fig. R32c**). This clearly demonstrates that barrier engineering enables ultralow power operation, albeit at the expense of switching speed.

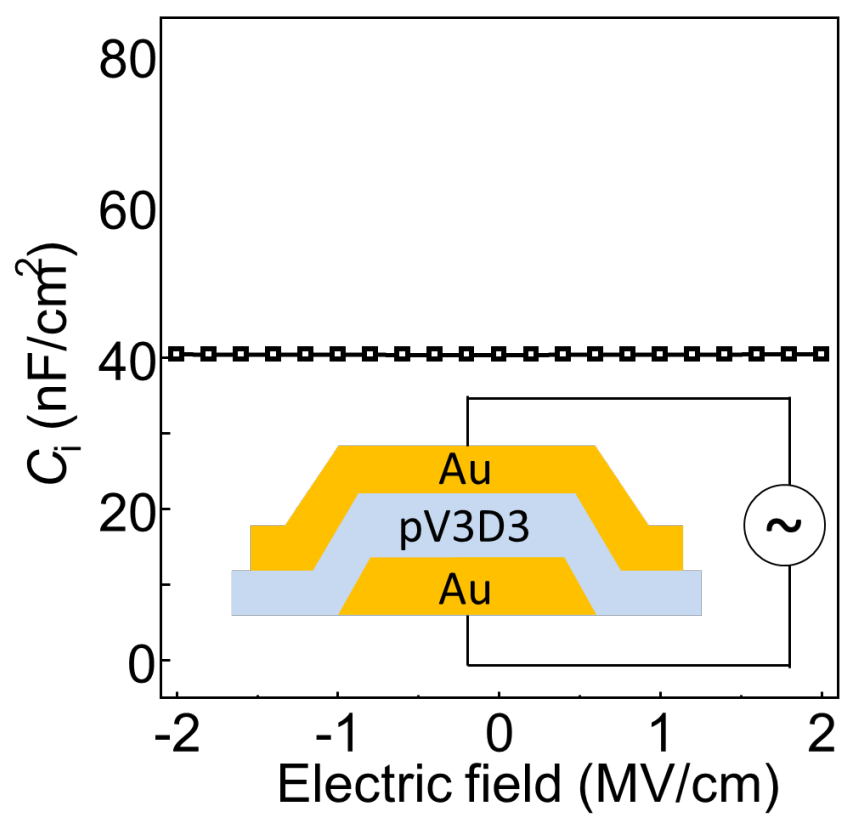

Figure R30. Capacitance per unit area (C_i)–electric field (E_i) of Au/poly(1,3,5-trivinyl-1,3,5-trimethyl cyclotrisiloxane) (pV3D3)/Au device.

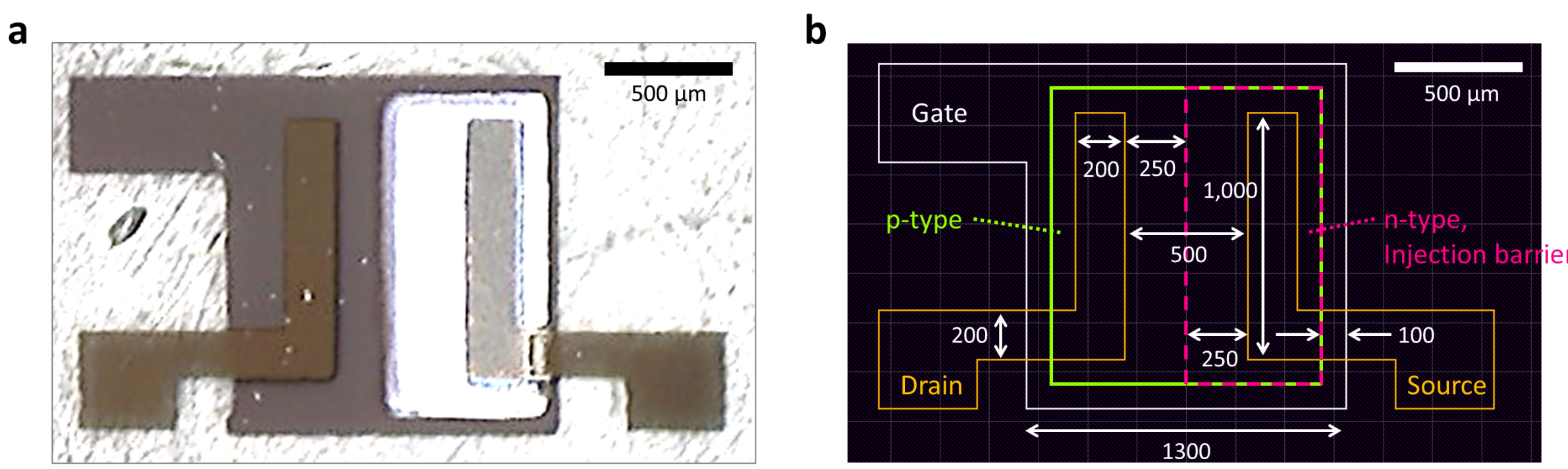

Figure R31. Dimensions of the T-HTR. **a.** An optical microscopy image (scale bar: 500 μm) and **b.** Shadow mask layout of the T-HTR (scale bar: 500 μm).

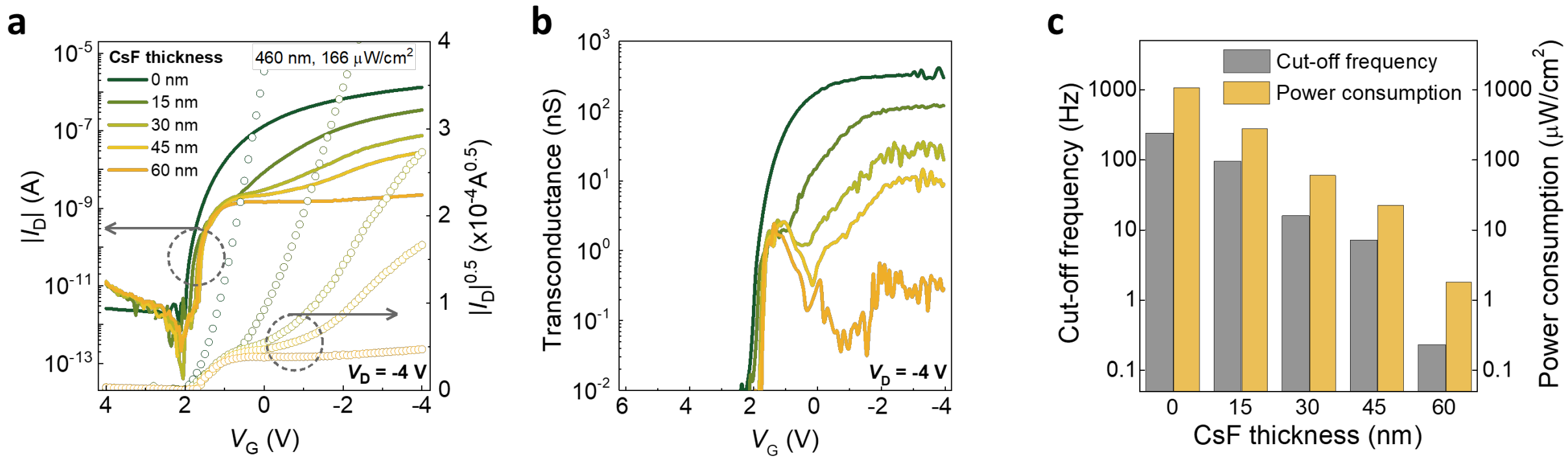

Figure R32. a. The transfer characteristics, **b**. transconductance (g_m), and **c**. cut-off frequency versus static power consumption of the T-HTR as a function of CsF barrier thickness. Increasing the CsF thickness reduces both the cut-off frequency and power consumption, illustrating the speed-power trade-off governed by injection barrier engineering.

Rather than a drawback, this trade-off is highly relevant for application domains where ultralow static power is prioritized over high clock speed. In many always-on sensors, wearable biomedical monitors, and distributed environmental sensor nodes, the required operating frequencies typically lie only in the Hz–kHz regime, while the overriding design constraint is to minimize static power into the μW or even nW–pW range for long-term autonomous operation [R7], [R8], [R9], [R10]. This consensus has been consistently highlighted in the literature across wearable systems, energy-harvesting wireless sensor networks, and many other always-on sensing platforms[R11], [R12], [R13], [R14]. Moreover, our approach is particularly well suited for low-power applications where minimizing both energy consumption and operation steps is critical, as the use of ternary logic reduces the number of computation steps to approximately log2/log3 ≈ 63.1 % compared to binary logic[R15]. This highlights that barrier engineering in T-HTRs provides a practical pathway toward energy-efficient, multi-valued logic systems.

Also, we emphasize that the present device was fabricated from thermally evaporated organic semiconductors patterned with shadow masks. This simple process is highly suitable for proof-of-concept demonstrations of heterojunction transistors, but it inevitably yields devices with relatively large dimensions, with critical lengths on the order of several hundred micrometers. By employing high-resolution patterning techniques such as standard photolithography, which allows scaling down to a few micrometers, the gate-to-source/drain overlap could be drastically reduced, thereby minimizing parasitic capacitances. In such a

scenario, C_{GG} would decrease by approximately four orders of magnitude, leading to a corresponding ~10^4–fold improvement in the cut-off frequency, potentially reaching the hundreds of kHz range. Notably, this improvement arises purely from the reduction of parasitic capacitances, which does not directly affect energy consumption–although a lower parasitic capacitance can further reduce the overall switching energy. Moreover, the approach demonstrated in this work is mainly based on the band engineering through injection barrier design, and is therefore not limited to a particular semiconductor class. This implies that, with high-mobility semiconductors such as oxides or two-dimensional materials, the attainable operation speed could further be enhanced into the MHz regime.

In the revised manuscript, we have included this discussion to clarify the implications of the current–frequency trade-off and to contextualize the relevance of our device concept for low-power applications.

[R6] *Superlattices and Microstructures*, **90**, 191-197 (2016).
[R7] *IEEE journal of biomedical and health informatics*, **20**(5), 1251-1264 (2016).
[R8] *Sensors*, **21**(2), 548 (2021).
[R9] Nano-Micro Letters, **16**(1), 52 (2024).
[R10] *IEEE Sensors Journal*, **23**(15), 16467-16480 (2023).
[R11] *Advanced Functional Materials*, **27**(15), 1605271 (2017).
[R12] The innovation, **4**(5), 100485 (2023).
[R13] *IEEE Sensors Journal*, **19**(20), 9047-9062 (2019).
[R14] Energy & Environmental Science, **14**(4), 2114-2157 (2021).
[R15] *IEEE Transactions on computers*, **100**, 1160-1179 (1984).

The revised parts are as follows;

(1) Revised the main text in the revised manuscript (p. 9).

A poly(1,3,5-trimethyl-1,3,5-trivinyl cyclotrisiloxane) (pV3D3, 50 nm) layer deposited in vapor-phase via initiated chemical vapor deposition (iCVD) was utilized as the gate dielectric layer[41]. The dielectric constant of the pV3D3 film was determined to be approximately 2.2, from which the dielectric thickness in the T-HTR was determined to be 50 nm (**Supplementary Fig. 2**).

(2) Revised the main text in the revised manuscript (p. 15).

The influence of the injection barrier thickness on the intermediate state was investigated (**Fig. 2e**). As the CsF thickness increased from 15 nm to 60 nm, the transition into the intermediate state became more distinct. The range of the intermediate state – defined as the V_G window between the initial V_G, where transconductance started to decrease from its peak, and the end V_G, where transconductance increased back to a level comparable to the initial peak value – broadened from less than 1 V for 15 nm-thick CsF to more than 4 V for 60 nm-thick CsF (**Fig. 2f**). Such expansion resulted from the progressively restricted effective charge injection from the source with increasing barrier thickness, which shifted the onset voltage of field-effect current to higher V_G.

To further elucidate how the injection barrier thickness affects the device figure of merit, we quantitatively analyzed its influence on the operating speed and power consumption (**Supplementary Fig. 14**). As the CsF thickness increased, the on-current gradually decreases due to the restricted charge injection from the source. The reduction in on-current lowers the transconductance and consequently the unity-gain cut-off frequency ($f_T = g_m/2\pi C_{gg}$ where g_m is the transconductance and C_{GG} is the total gate capacitance)[57], while the static power consumption ($P = I \times V$, where I and V denote the operating current and driving voltage, respectively) decreases proportionally, resulting in a clear trade-off between operating speed and power efficiency. As the CsF thickness increased from 15 nm to 60 nm, the estimated cut-off frequency decreased from 10^2-10^3 Hz to below 1 Hz, whereas the static power density dropped from the mW cm^{-2} scale to the sub-μW cm^{-2} scale.

(3) Added the **Figure R30** to Supplementary Information (p. 3)

(4) Added **Figure R31** and **Figure R32** to the Supplementary Information, together with the corresponding descriptions (p. 19).

As the CsF thickness increased from 15 nm to 60 nm, the estimated f_{T} decreased from 10^2-10^3 Hz to below 1 Hz, whereas the static power density dropped from the mW cm^{-2} scale to the sub-μW cm^{-2} scale. Such a speed–power compromise is highly relevant for application domains where ultralow static power is prioritized over high operating frequency, including always-on optical sensors, wearable biomedical monitors, and distributed environmental sensor nodes typically operating in the Hz–kHz regime[1~8]. Furthermore, the ternary-logic framework intrinsically reduces the number of computational steps by ≈ 63 % ($\log_2$ / $\log_3$)[9], offering additional energy savings.

Although the present proof-of-concept devices employ large shadow-mask-defined geometries, scaling the channel length and minimizing gate–electrode overlap through photolithography would reduce C_{GG} by ~10^4-fold, potentially extending the cut-off frequency into the hundreds-of-kHz range. Also, combined with high-mobility semiconductors such as oxides or 2D materials, the attainable speed could further approach the MHz regime.

(5) Added the reference [1-9] in the Supplementary Information.

Q5) It is recommended that V_{D} and G_{ND} are added to Fig 1a and Fig. 3a to illustrate the test setup of T-HTR.

Response:

We thank the Reviewer for the helpful suggestion. We agree that explicitly indicating the V_{D} and G_{ND} in Fig. 1a and Fig. 3a will make the measurement setup of the T-HTR clearer. In the revised manuscript, we have modified both schematic diagrams to include V_{D} and G_{ND} labels, thereby improving the clarity of the device configuration.

(1) Revised the Fig. 1a (p. 31) and Fig. 3a (p. 33).

Response to the Reviewers' Comments

The authors thank the Reviewers for their thorough review of our manuscript and the valuable comments. We have revised the manuscript and supporting information based on the Reviewers' comments. The Reviewers' comments appear in **black**, and the authors' responses are in **blue**. In the revised manuscript, the changes with respect to the previous version are highlighted.

Reviewer (#1)'s COMMENTS:

The authors report a light-driven ternary logic device, demonstrating in-sensor computing functionality with a particular emphasis on low static power consumption. Overall, the topic is timely and the general device concept is interesting. However, several important issues need to be clarified.

Q1) The authors state that there is a trade-off between power consumption and operating frequency, but some relevant data are inconsistent or not clearly presented. In the main text, when referring to Supplementary Table 1, the authors quote a static power of 3.2 pW/cm^2, whereas Supplementary Table 1 uses 3.2 nW/cm^2. Figures 2g and 2h compare devices without CsF and with 60 nm CsF, but the rise and fall times shown there appear inconsistent with the cutoff frequencies for 0 nm and 60 nm CsF in Supplementary Figure 14e. In addition, on page 10 the authors mention CsF thicknesses from 0 to 90 nm, but only provide experimental results up to 60 nm. Please also specify which CsF thickness is used for the devices in Figures 4 and 5, and which CsF thicknesses and cutoff frequencies correspond to the reported static power densities of 3.2 pW/cm^2 and 3.2 nW/cm^2. **Q3)** Why do the authors attribute the conduction in the CsF layer to Poole–Frenkel conduction rather than Schottky emission?"

Response:

We sincerely appreciate the reviewer's careful examination and constructive comments. We have thoroughly reviewed the manuscript and supplementary information on these points and have revised the text accordingly to enhance clarity and accuracy.

1) Clarification of the static power consumption value (3.2 pW/cm^2 vs 3.2 nW/cm^2)

The correct value is 3.2 pW/cm^2, and the value listed as 3.2 nW/cm^2 in Supplementary Table 1 was the result of a typographical oversight. We have revised Supplementary Table 1 to

ensure consistency and avoid confusion. We sincerely apologize for all the confusion that we may have caused in the previous submission.

2) Comparison between transient photocurrent response (Fig. 2g-h) and cut-off frequency estimation (Supplementary Fig. 15e)

The two datasets address distinct physical processes. Fig. 2g and 2h evaluate the transient photocurrent response under a constant bias condition, where only the optical input is modulated. These measurements probe how rapidly photogenerated carriers are separated and transported to the electrodes, and were primarily used to infer the impact of trap density at the semiconductor/contact interface. In contrast, Supplementary Figure 15e provides an analytically derived cut-off frequency, based on the small-signal unity-gain frequency:

$$f_{\mathrm{T}} = \frac{g_m}{2\pi C_{\mathrm{gg}}}$$

where g_{m} is the transconductance and C_{GG} is the total gate capacitance. g_{m} was obtained from transfer characteristics of a light-driven ternary heterojunction transistor (T-HTR). This estimation reflects the electrical switching limit under gate modulation, rather than the transient optical response. We agree that without clarification these two metrics may appear inconsistent. We have thus added explanatory text in the Results section to clearly describe the physical meaning and measurement conditions of each quantity.

3) Clarification regarding CsF thickness range (0-90 nm) referenced in the main text

The complete CsF thickness-dependent measurements (0-90 nm) are provided in Supplementary Figure 5, where the current density-electric field ($J_{\mathrm{i}}-E_{\mathrm{i}}$) plot of Au/C10-DNTT/C13-PTCDI/CsF/Au vertical structures, designed to emulate the source-side configuration of the T-HTR, were measured to determine the conduction mechanism.

Importantly, this dataset also forms the experimental basis for our response to Q3 **[Q3) Why do the authors attribute the conduction in the CsF layer to Poole–Frenkel conduction rather than Schottky emission?"]**. In this study, a CsF thickness of 60 nm was adopted as the representative condition for the T-HTR, as it reliably enabled the formation of a stable intermediate state while preserving clear on/off switching behavior. Given that CsF exhibits a wide bandgap characteristic as an insulating dielectric (>5 eV), Schottky emission

through a 60 nm-thick layer is unlikely to be responsible for the observed current transport. Instead, when analyzing the $J_i - E_i$ characteristics of the vertical CsF structures, the data showed excellent linearity in Poole–Frenkel coordinates [ln(J_i/E_i) vs $\sqrt{E_i}$], demonstrating that carrier transport occurs primarily through trap-assisted hopping rather than through a Schottky emission process. This finding supports our interpretation that the conduction mechanism in the CsF layer is dominated by Poole-Frenkel transport.

4) Specification of CsF thickness used in Figure 4 and Figure 5, and clarification of thickness values corresponding to reported power densities

All device results shown in Figure 4 and Figure 5 including the ternary inverter, ternary logic gates, and sensory circuit were obtained using the 60 nm CsF configuration, which was selected as the representative device structure considering its well-defined intermediate state. Accordingly, the ultralow static power density of 3.2 pW/cm^2 reported in the main text corresponds to devices fabricated with 60 nm CsF.

It is important to clarify that this value represents the static power consumption in the intermediate (standby) state, where the device operates at a near-off current level in the absence of optical modulation. In contrast, the power consumption discussed in Supplementary Figure 15 corresponds to the on-state (dynamic) power consumption, evaluated under full electrical conduction and used to analyze the trade-off between operating frequency and power consumption as a function of CsF thickness. This distinction explains why the power densities in Supplementary Figure 15 are orders of magnitude higher (from the mW/cm^2 to sub-μW/cm^2 range) than the ultralow standby power reported for the intermediate state (3.2 pW/cm^2). To avoid all the potential ambiguity, we have explicitly specified the CsF thickness used in Figure 4 and Figure 5 and clarified the definitions of static and on-state power consumption in the relevant sections of the main text and Supplementary Information.

We also note that the power consumption values in Supplementary Figure 15 were previously referred to as static power, whereas they in fact correspond to on-state (dynamic) power consumption, which may have caused confusion when comparing these values with the intermediate-state static power reported in the main text. This terminology has now been corrected throughout the manuscript.

We revised the manuscript as follows:

(1) Revised the Supplementary Table 1 in the Supplementary Information (p. 32).

Supplementary Table 1 | Comparison of static power consumption with the previously reported ternary logic transistor.

Operating principle	Driving voltage at logic '1'	Static current at logic '1'	Channel (length × width)	Static power consumption at logic '1'	Ref
Negative transconductance	20 V	> 10 nA	400 μm × 1,000 μm	> 50 μW/cm²	[10]
Negative transconductance	3 V	> 1 nA	500 μm × 1,000 μm	> 0.6 μW/cm²	[11]
Negative transconductance	> 30 V	> 100 nA	1,000 μm × 2,200 μm	> 136 μW/cm²	[12]
Negative transconductance	1 V	> 0.1 nA	1 μm × 3 μm	> 3.3 mW/cm²	[13]
Negative transconductance	2 V	> 1,000 nA	3 μm × 20 μm	> 3.3 W/cm²	[14]
Zero transconductance	1 V	6 nA	100 μm × 150 μm	40 μW/cm²	[15]
Zero transconductance	1 V	4 nA	60 μm × 80 μm	83 μW/cm²	[16]
Zero transconductance	1 V	2 nA	1 μm × 55 μm	3.6 mW/cm²	[17]
Zero transconductance	1 V	0.1 nA	1 μm × 1 μm	10 mW/cm²	[18]
Zero transconductance	10 V	> 3 nA	100 μm × 1,000 μm	30 μW/cm²	[19]
**Zero transconductance**	**4 V**	**0.004 nA**	500 μm × 1,000 μm	**3.2 pW/cm²**	**This work**

(2) Revised the main text in the revised manuscript (p. 16).

The influence of the injection barrier on response speed was also investigated by applying a 460 nm light pulse (2.01 mW/cm^2) for 1 s and measuring the photocurrent in the intermediate state (**Fig. 2g**), which captures the transient response of photogenerated carriers under a constant electrical bias. Accordingly, this transient optical response is distinct from the cut-off frequency discussed above, which reflects the electrical switching limit under gate modulation.

(3) Revised the main text in the revised manuscript (p. 2).

This design suppresses effectively the field-effect charge injection while preserving photogenerated carrier transport, leading to the formation of stable photocurrent

proportional to the input light intensity, with a dynamic range exceeding 10^5. This enables the device to be maintained in the off-state in the absence of intermediate logic operations, thereby minimizing static power consumption to as low as ~3.2 pW/cm^2 in the intermediate state.

(4) Revised the main text in the revised manuscript (p. 13).

This also enabled the device to be maintained in the off-state in the absence of intermediate logic operations, thereby minimizing static power consumption to as low as ~3.2 pW/cm^2 in the intermediate state, which is significantly lower than that of conventional ternary logic transistors (**Supplementary Table 1**).

(5) Revised the main text in the revised manuscript (p. 16).

To further elucidate how the injection barrier thickness affects the device figure of merit, we quantitatively analyzed its influence on the operating speed and power consumption (**Supplementary Fig. 15**). As the CsF thickness increased, the on-current gradually decreases due to the restricted charge injection from the source. The reduction in on-current lowers the transconductance and consequently the unity-gain cut-off frequency ($f_T = g_m/2\pi C_{gg}$ where g_m is the transconductance and C_{gg} is the total gate capacitance)[57], while the static power consumption ($P = I \times V$, where I and V denote the operating current and driving voltage, respectively) decreases proportionally, resulting in a clear trade-off between operating speed and power efficiency. As the CsF thickness increased from 15 nm to 60 nm, the estimated cut-off frequency decreased from 10^2-10^3 Hz to below 1 Hz, whereas the on-state (dynamic) power density dropped from the mW cm^{-2} scale to the sub-μW cm^{-2} scale.

(6) Revised the Supplementary Information (p. 20).

As the CsF thickness increased from 15 nm to 60 nm, the estimated f_T decreased from 10^2-10^3 Hz to below 1 Hz, whereas the on-state (dynamic) power density dropped from the mW cm^{-2} scale to the sub-μW cm^{-2} scale. Such a speed–power compromise is highly relevant for application domains where ultralow static power is

prioritized over high operating frequency, including always-on optical sensors, wearable biomedical monitors, and distributed environmental sensor nodes typically operating in the Hz–kHz regime[1~8]. Furthermore, the ternary-logic framework intrinsically reduces the number of computational steps by ≈63 % (log2 / log3), offering additional energy savings[9].

(7) Revised the main text in the revised manuscript (p. 21).

The T-HTR, with its inherent capability to generate three distinct states under the optical stimulus, offers a promising platform for implementing optoelectronic ternary logic gates. By simply integrating a load resistor, a ternary logic inverter (T-inverter) was realized using the CsF-based T-HTR with a 60 nm-thick CsF layer (**Fig. 4a**).

(8) Revised the main text in the revised manuscript (p. 24).

The ability of the T-HTR to generate and modulate multiple logic states through electrical and optical inputs enables in-sensor computing, in which data processing occurs directly within the sensor, minimizing energy consumption for data transmission and storage. As a proof-of-concept, a sensory circuit consisting of four CsF-based T-HTRs with a 60 nm-thick CsF layer was fabricated as a pixel-level sensory circuit, where two pairs of T-HTRs were first connected in series, and these two serially connected pairs were then integrated in parallel (**Fig. 5a**).

Q2) Although Supplementary Figure 15 compares the effect of channel length, this alone is not sufficient to prove that the cutoff frequency is entirely determined by parasitic capacitance. It may also be limited by the intrinsic properties of the materials. Therefore, the estimate given after Supplementary Figure 14 that the device still has at least a 10^4-fold potential improvement in operating frequency appears somewhat optimistic. Moreover, more importantly, if the operating frequency is limited by $1/(2\pi RC)$, simple geometric scaling may not necessarily improve the RC-limited frequency: as the device area is reduced, the capacitance C decreases, but the resistance R increases proportionally.

Response:

We appreciate the Reviewer's insightful comments and agree that our previous discussion may have presented an overly simplified interpretation of the complex factors governing the cut-off frequency. As the Reviewer correctly pointed out, reducing device dimensions in an isotropic manner – i.e., uniformly scaling down both width and channel length – would not necessarily yield meaningful improvement, as the decrease in capacitance could be partially offset by a proportional increase in channel resistance. We fully acknowledge that the original text did not sufficiently clarify this nuance.

We would like to clarify that our intention was not to claim that parasitic capacitance is the sole determinant of the operating frequency, nor that a 10^4-fold improvement is guaranteed through uniform geometric scaling. Instead, our discussion aimed to highlight that targeted scaling, particularly in reducing the gate-to-source/drain overlap area while maintaining the aspect ratio of the active channel, can selectively suppress parasitic capacitance without proportionally affecting the channel resistance. Under such scaling conditions, the capacitance, C decreases while the resistance, R remains largely unchanged, which potentially enables a meaningful improvement in cut-off frequency.

Furthermore, we cannot agree more with the Reviewer that parasitic capacitance is not the sole parameter governing the cut-off frequency. Intrinsic factors including carrier mobility or charge trapping dynamics at the interface also influence the device switching speed. In our present proof-of-concept demonstration, however, the devices were fabricated using a shadow mask process, resulting in relatively large geometries. Under these fabrication constraints, the most analytically tractable and experimentally verifiable avenue for improving the cut-off frequency was the reduction of gate–overlap-induced parasitic capacitance. Based on this consideration, we initially used a capacitance scaling argument to discuss the potential improvement. However, upon reflection, we acknowledge that suggesting a quantitative projection of up to a 10^4-fold enhancement may have been an overstated degree of certainty.

In light of the Reviewer's comment, we have revised the manuscript to avoid implying deterministic numerical scaling and have removed the explicit 10^4 projection. The revised text now focuses on a qualitative description, emphasizing that overlap-area minimization represents one potential–but not exclusive–strategy to improve operating frequency, particularly for low-power ternary device architectures.

We revised the manuscript as follows:

(1) Revised the Supplementary Information (p. 20).

As the CsF thickness increased from 15 nm to 60 nm, the estimated f_T decreased from 10^2-10^3 Hz to below 1 Hz, whereas the on-state (dynamic) power density dropped from the mW cm^{-2} scale to the sub-μW cm^{-2} scale. Such a speed–power compromise is highly relevant for application domains where ultralow static power is prioritized over high operating frequency, including always-on optical sensors, wearable biomedical monitors, and distributed environmental sensor nodes typically operating in the Hz–kHz regime[1~8]. Furthermore, the ternary-logic framework intrinsically reduces the number of computational steps by ≈63 % (log2 / log3), offering additional energy savings[9].

In the present proof-of-concept devices, the channel structures were defined using a shadow-mask process, which inevitably results in relatively large device geometries and thereby substantial gate-electrode overlap areas. Under such conditions, the estimated cut-off frequency is strongly influenced by geometrical capacitance associated with these overlap regions. Therefore, minimizing gate-electrode overlap through lithographic patterning could provide a practical pathway for reducing parasitic capacitance and improving the operating frequency in future device implementations.

These analyses demonstrate that injection-barrier engineering provides a practical and tunable design handle to balance operation speed and power efficiency in light-driven multi-state logic systems.

Q4) On page 10 the text states "2.33 eV for C10-DNTT", whereas on page 19 it states "C13-PTCDI (2.33 eV)". One of these values appears to be incorrect.

Q5) The sentence "This improvement suggests that the CsF layer effectively suppresses the dominant trap-mediated relaxation pathways at the metal/semiconductor interface." is repeated twice (on pages 16 and 17).

Response:

We thank the Reviewer for carefully identifying these inconsistencies. The incorrect energy-level assignment has now been corrected: the value of 2.33 eV corresponds to the LUMO (electron affinity) of C10-DNTT, whereas the LUMO level of C13-PTCDI is 4.36 eV. Additionally, the duplicated sentence ("This improvement suggests that the CsF layer effectively suppresses the dominant trap-mediated relaxation pathways at the metal/semiconductor interface.") appeared in two consecutive paragraphs due to an editing oversight. The redundant sentence has now been removed to avoid repetition.

We appreciate the Reviewer's attention to these details, and the manuscript has been revised accordingly.

(1) Revised the main text in the revised manuscript (p. 19).

Although both photogenerated holes from the p-type semiconductor and electrons from the source are in principle available, the energy alignment strongly favors hole transport while suppressing electron injection: the Au/MoO_X work function (~5.75 eV) aligns closely with the HOMO level of C10-DNTT (4.92 eV), whereas the LUMO level of C13-PTCDI (4.36 eV) imposes a large barrier to electron injection.

(2) Revised the main text in the revised manuscript (p. 17).

In contrast, with the 60 nm-thick CsF injection barrier, the rising time was significantly reduced to 34.3 ms, and the falling time shortened to 31.2 ms, resulting in a much sharper transient response with well-defined on/off switching behavior. This improvement suggests that the CsF layer effectively suppresses the dominant trap-mediated relaxation pathways at the metal/semiconductor interface. We note, however, that the reversible V_{TH} modulation observed under illumination indicates the possible presence of a small number of rapidly responding interfacial states or dipole-forming sites at the CsF/semiconductor boundary.

Reviewer (#2)'s COMMENTS:

Though the authors have demonstrated significant improvements in this revised manuscript, the proposed operating mechanism is still not quite convincing.

Q1) TCAD simulations are strongly recommended to show the evolution of band diagrams. The electron affinity and bandgap of each material can be used as inputs to generate the band alignment profiles for each bias condition.

Response:

We thank the Reviewer for the constructive recommendation. To provide a more solid physical analysis, we have performed TCAD simulations.

To ensure computational convergence while preserving the physical validity of the charge transport, the simulated device features a scaled channel length of 5 μm, with the layer thickness kept identical to the actual device (**Fig. R1**).

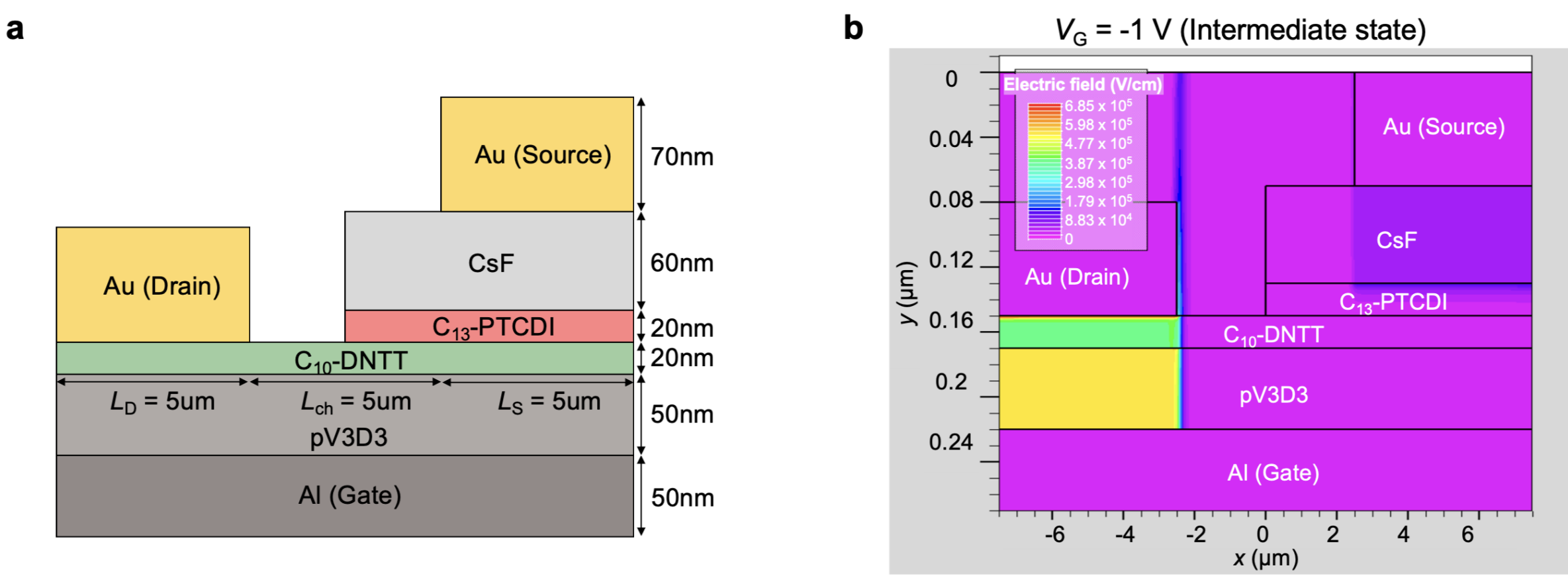

Figure R1. a. The schematic illustration of the scaled p-type T-HTR device structure used for the simulation (channel length = 5 μm). **b.** Electric field distribution in the intermediate state (V_G = -1 V, V_D = -4 V).

All the input parameters used for the simulations are summarized in **Table R1**, incorporating values that were either determined experimentally or adopted from the literature[R1], [R2], [R3], [R4]. In TCAD simulation, C10-DNTT and C13-PTCDI were defined as semiconductors, whereas the injection barrier layer (CsF) was defined as insulators, since CsF exhibited a large bandgap of approximately 5.29 eV along with a valence band maximum of around 7.1 eV (**Fig. R2**).

Table R1. Parameters used for the TCAD simulation.

Material	Parameter	Value
Al	Work function	4.3 eV
	Refractive index (n) / Extinction coefficient (k) @460 nm	0.541 / 5.119
Au	Work function	4.9 eV
	Refractive index (n) / Extinction coefficient (k) @460 nm	1.394 / 1.828
C_{10}-DNTT	LUMO	2.33 eV
	HOMO	4.92 eV
	Band gap	2.59 eV
	Dielectric constant	3
	Electron mobility	10^{-3} $cm^2V^{-1}s^{-1}$
	Hole mobility	6 $cm^2V^{-1}s^{-1}$
	Doping concentration	10^{15} cm^{-3}
	Conduction band density of states (N_C)	10^{20} cm^{-3}
	Valence band density of states (N_V)	10^{20} cm^{-3}
	Refractive index (n) / Extinction coefficient (k) @460 nm	1.198 / 0.016
C_{13}-PTCDI	LUMO	4.36 eV
	HOMO	6.42 eV
	Band gap	2.06 eV
	Dielectric constant	3
	Electron mobility	1.3 $cm^2V^{-1}s^{-1}$
	Hole mobility	10^{-3} $cm^2V^{-1}s^{-1}$
	Conduction band density of states (N_C)	10^{20} cm^{-3}
	Valence band density of states (N_V)	10^{20} cm^{-3}
	Refractive index (n) / Extinction coefficient (k) @460 nm	1.370 / 0.066
CsF	Conduction band energy	1.81 eV
	Valence band energy	7.1 eV
	Band gap	5.29 eV
	Dielectric constant	7.9 [R1]
	Refractive index (n) / Extinction coefficient (k) @460 nm	1.607 / ~ 0
MoOx	Conduction band energy	5.8 eV [R2]
	Valence band energy	9.0 eV [R2]
	Band gap	3.2 eV
	Dielectric constant	20 [R3]
	Refractive index (n) / Extinction coefficient (k) @460 nm	2.065 / ~ 0
pV3D3	LUMO	1.2 eV [R4]
	HOMO	9.45 eV [R4]
	Band gap	8.25 eV
	Dielectric constant	2.2 [R4]
	Refractive index (n) / Extinction coefficient (k) @460 nm	1.498 / ~ 0

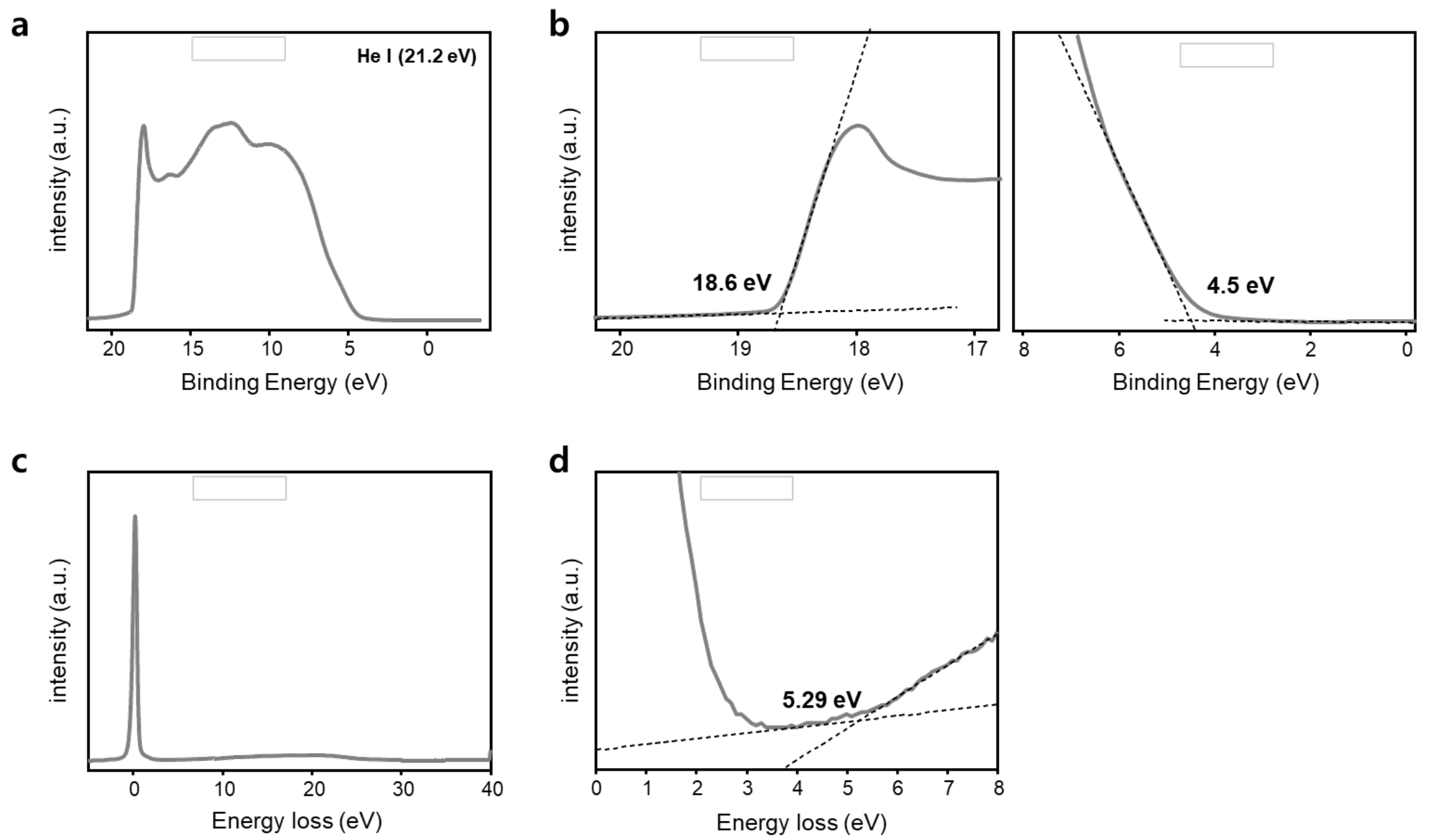

Figure R2. a. Ultraviolet photoelectron spectroscopy (UPS) spectra of the CsF. (b) The enlarged UPS spectra at highest binding energy cut-off (left) and lowest binding energy cut-off (right). (c) Reflected electron energy loss spectroscopy (REELS) spectra of CsF. (d) The enlarged REELS spectra.

To accurately capture the energy band modulation under illumination, we incorporated the optical generation model (optr) combined with the beam propagation method (BPM). Furthermore, the TR.MATRIX parameters were specified to enable transfer matrix method, ensuring precise calculation of the optical intensity distribution within the multi-layer thin-film stack. The *n* and *k* values of each material were experimentally determined via ellipsometry. A light source with a wavelength of 460 nm was applied at normal incidence from the top of the device.

Fig. R3 presents the simulated band diagrams for the p-type T-HTR evaluated at three representative V_G states. In the OFF state (V_G = 2 V), the fully depleted channel suppresses charge transport, yielding negligible current. Under illumination, photogenerated electrons move toward the p-type interface, partially screening the electric field and resulting in a flatter band profile across the n-type/CsF region. In the intermediate state (V_G = -1 V), 60 nm-thick CsF barrier effectively blocks the gate-induced hole injection. However, under illumination,

photogenerated electrons accumulate at the CsF interface. This interfacial charge steepens the CsF energy band, enhancing the internal electric field to enable trap-assisted photocurrent with near-zero transconductance. Finally, in the ON state (V_G = -6 V), a strong negative gate bias sufficiently lowers the source-side injection barrier, allowing for dominant field-effect hole transport through the channel.

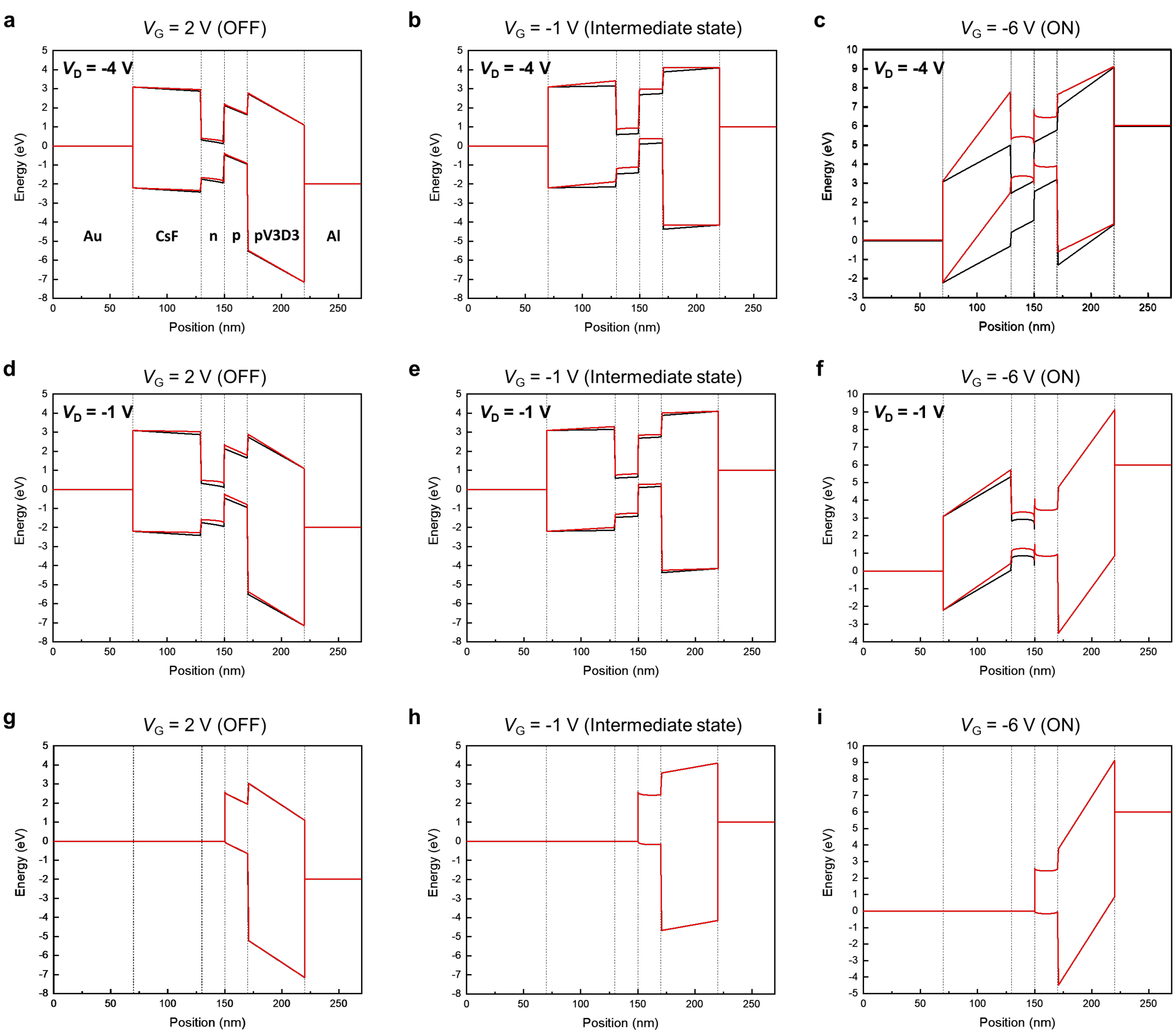

Figure R3. Simulated energy band diagrams of the scaled p-type T-HTRs at the source-side, in the dark (black lines) and under illumination (red lines; the wavelength of 460 nm) at three representative V_G states: **a**. V_G = 2 V (OFF), **b**. -1 V (intermediate), and **c**. -6 V (ON). For **a-c**, V_D = -4 V. Corresponding diagrams at V_D = -1 V are shown in **d-f**. Corresponding diagrams for the reversed source/drain configuration are shown in **g-i**. For g-i, V_D = -4 V.

Beyond the gate-driven modulation, the simulation also elucidates the influence of the drain bias (V_D) on the source-side bands (**Fig. R3d-f**). Increasing the V_D from -1 V to -4 V increases the lateral electric field. Therefore, a part of the drain potential also drops across the source-side dielectric stack, effectively increasing the internal field in CsF layer. This enhanced field strengthens Poole-Frenkel conduction by lowering the effective barrier ($\emptyset_B - \sqrt{eE_{eff}/\pi\varepsilon_i}$), leading to an increase of current ($J \propto E_{eff} \times \exp[\sqrt{E_{eff}}]$). Here, E_{eff} represents the combined field composed of the gate-induced component and the additional field induced from the drain bias and photogenerated interfacial charge accumulation.

When the source and drain are reversed (**Fig. R3g-i**), the source directly contacts the p-type channel. This eliminates the injection barrier from CsF layer, resulting in conventional transistor operation without the distinct intermediate state.

The TCAD simulation results for the n-type T-HTR (**Fig. R4** and **Fig. R5**) exhibits symmetrical operational principles. Evaluated at V_G = -2 (OFF), 8 V (intermediate), and 15 V (ON) with V_D = 2 V and 10 V, the device behavior is consistently governed by the MoO_X injection barrier and photogenerated hole accumulation. The reverse configuration similarly results in the loss of the intermediate state, verifying the universality of the proposed design principles. We have included these simulation results and corresponding discussions in the revised manuscript to clearly illustrate the operational mechanism.

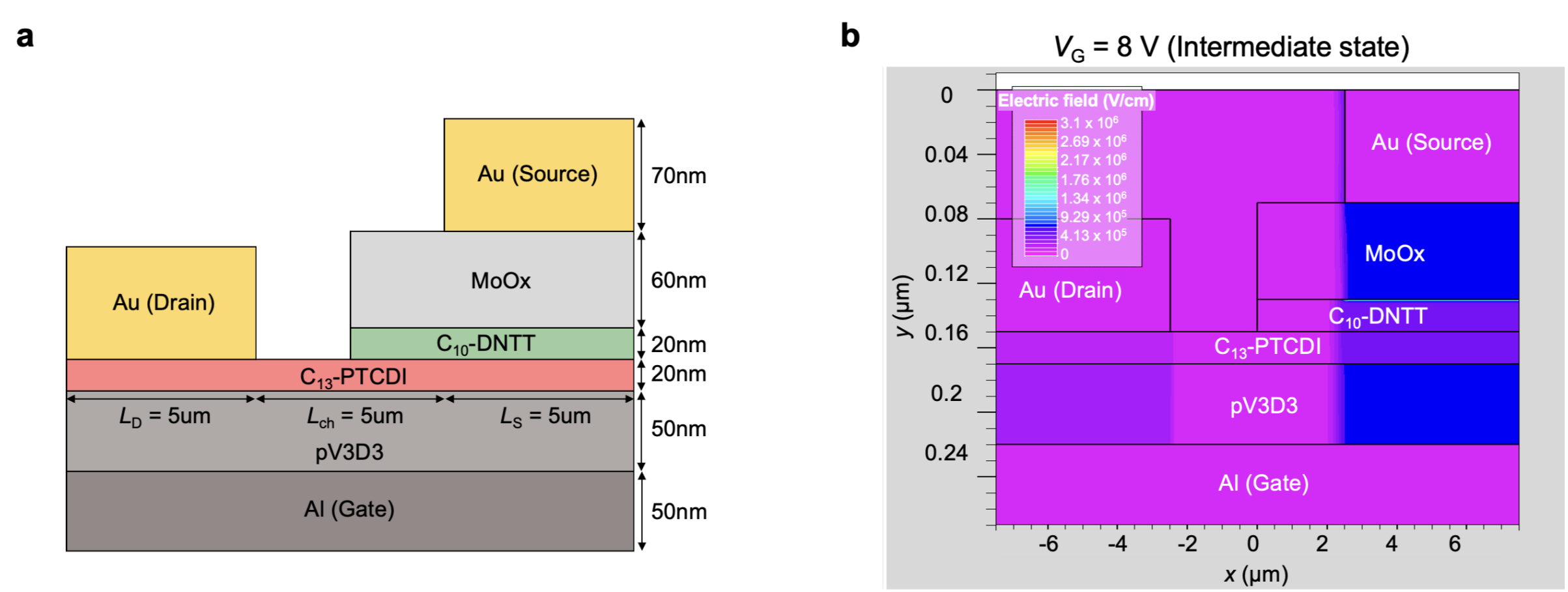

Figure R4. a. The schematic illustration of the scaled n-type T-HTR device structure (channel length = 5 μm). **b.** Electric field distribution in the intermediate state (V_G = 8V, V_D = 10 V).

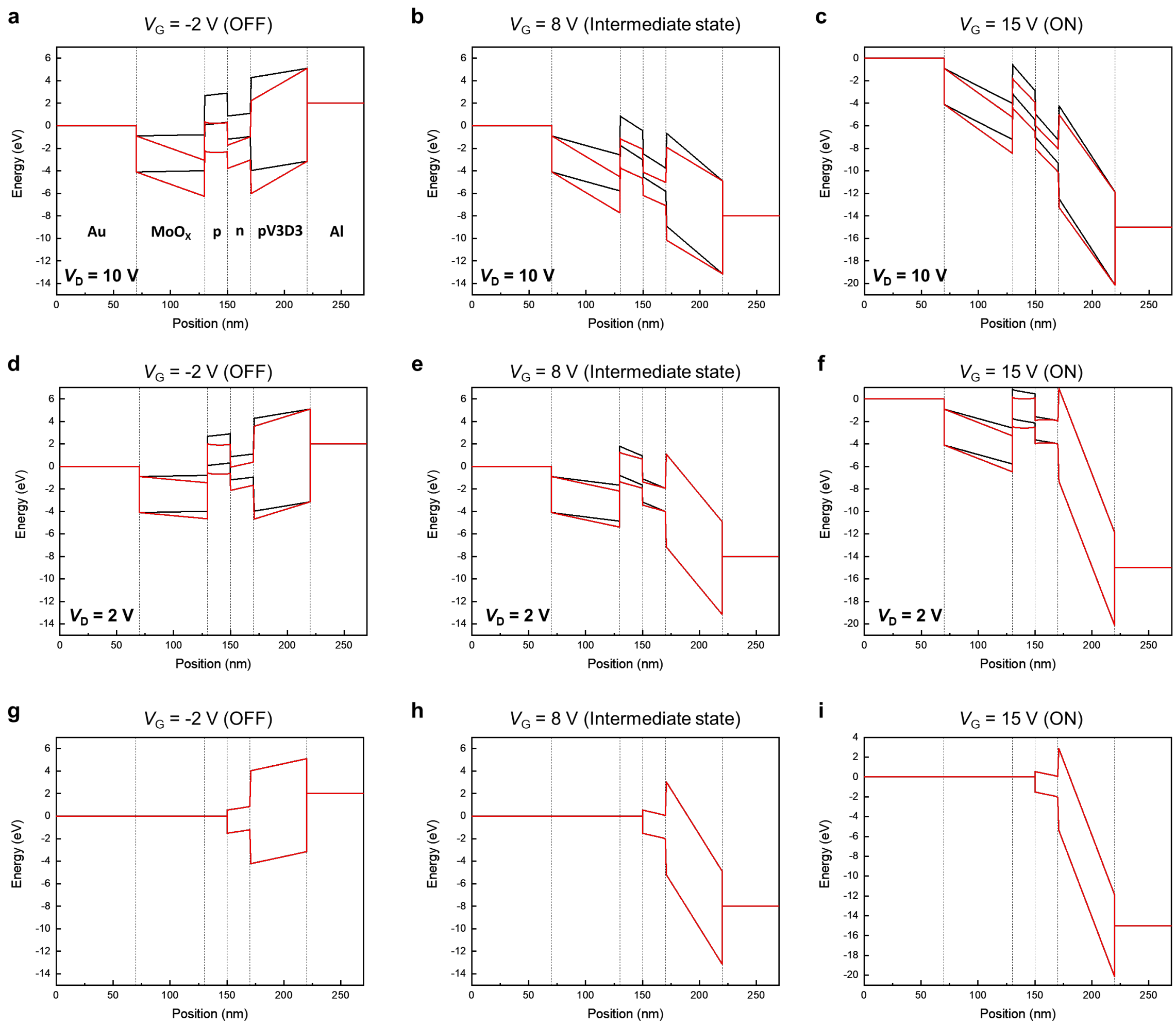

Figure R5. Simulated energy band diagrams of the scaled n-type T-HTRs at the source-side, in the dark (black lines) and under illumination (red lines; the wavelength of 460 nm) at three representative V_G states: **a**. V_G = -2 V (OFF), **b**. 8 V (intermediate), and **c**. 15 V (ON). For a-c, V_D = 10 V. Corresponding diagrams at V_D = 2 V are shown in **d-f**. Corresponding diagrams for the reversed source/drain configuration are shown in **g-i**. For g-i, V_D = 10 V.

[R1] *Physical Review*, **168**(3), 1054 (1968).

[R2] *Applied Physics Letters*, **96**(6), 063303 (2010).

[R3] *Advanced Electronic Materials*, **6**(10), 2000635 (2020).

[R4] *Nature Materials*, **14**(6), 628-635 (2015).

We appreciate the Reviewer's attention to band diagrams, and the manuscript has been revised accordingly.

(1) Revised Fig. 1g in the revised manuscript (p. 31).

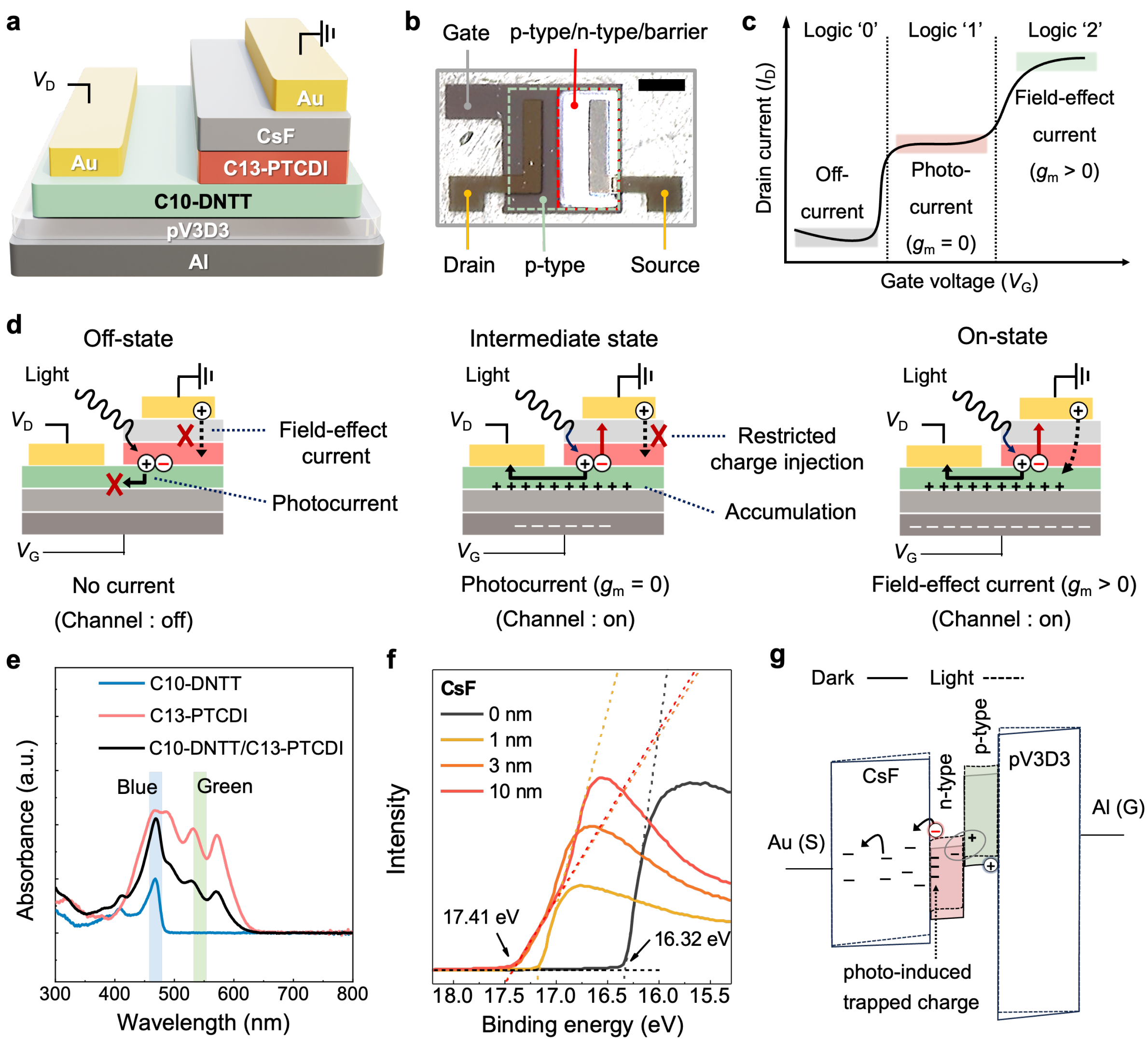

Figure 1 | Design of the ternary heterojunction transistor (T-HTR). a. The schematic illustration and **b**. optical microscopy image of the T-HTR (scale bar: 500 μm). **c**. The schematic illustration of transfer characteristics and **d**. operational mechanism of the T-HTR consisting of three switching states. **e**. Ultraviolet-visible (UV/Vis) absorption spectra of the n-type (C13-PTCDI), p-type (C10-DNTT) and n-type/p-type bilayer organic semiconductors. **f**. Ultraviolet photoelectron spectroscopy (UPS) spectra of Au contact with thermally deposited cesium fluoride (CsF) as a function of CsF

thickness. **g**. Energy band diagrams of the source-side stack in the dark (solid lines) and under illumination (dashed lines). The photo-induced trapped charge at the CsF/p-type interface enhances the internal electric field in CsF.

(2) Revised Fig. 3c in the revised manuscript (p. 33).

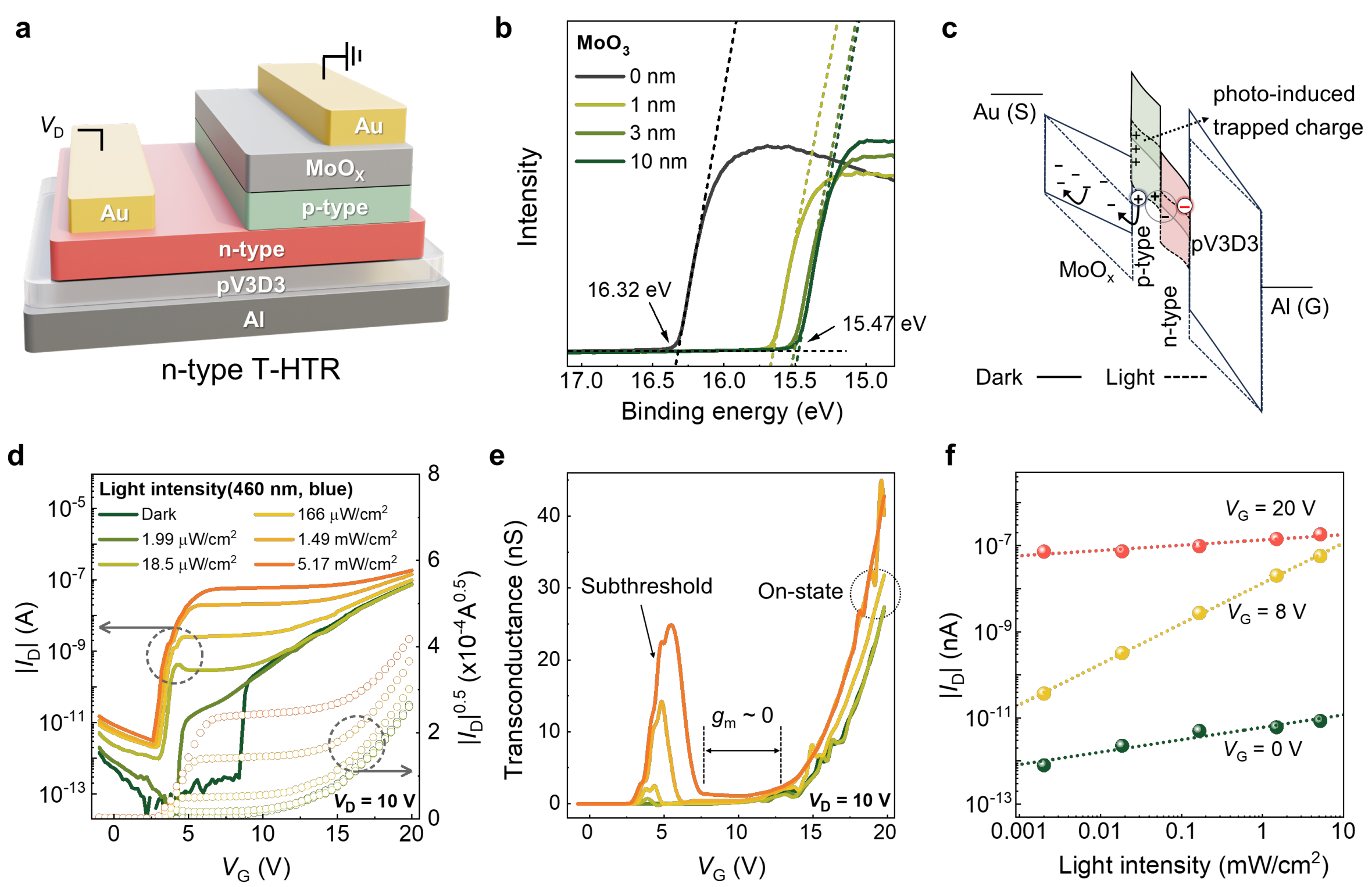

Figure 3 | Versatility of the T-HTR architecture. a. A schematic illustration of the T-HTR with molybdenum oxide (MoO_X) injection barrier. **b**. UPS spectra of Au contact with thermally deposited MoO_X as a function of MoO_X thickness. **c.** Energy band diagrams of the source-side stack in the dark (solid lines) and under illumination (dashed lines). The photo-induced trapped charge at the MoO_X/n-type interface enhances the internal electric field in MoO_X. **d.** The transfer characteristics, **e.** g_m, and **f.** I_D at the off-, intermediate, and on-state of the T-HTR under 460 nm illumination with respect to intensity.

(3) Added Supplementary Figure. 4 with **Fig. R2** in the revised Supplementary Information (p. 5).

(4) Revised the main text in the revised manuscript (p. 10).

In this study, a 60 nm-thick CsF layer was employed to enhance the injection barrier functionality beyond dipole formation. The CsF exhibited a large bandgap of approximately 5.29 eV along with a valence band maximum of around 7.1 eV (**Supplementary Fig. 4**).

(5) Replaced Supplementary Figure. 8 with **Fig. R3** in the revised Supplementary Information (p. 10).

(6) Replaced Supplementary Figure. 17 with **Fig. R5** in the revised Supplementary Information (p. 23).

(7) Added the **Table R1** to Supplementary Information (p. 33)

(8) Added the reference [1-4] in the Supplementary Information.

Q2) If the authors determined the interface trap density (Q_{it}) from gate capacitance per unit area (C_{ox}), why did they divide the result by the gate area again to obtain the surface charge density (σ)?

Response:

We thank the Reviewer for pointing out this critical notational error, and we apologize for the confusion we may have caused.

In our previous response letter, we intended for Q_{it} to denote the total trapped charge at the interface. Unfortunately, however, we mistakenly wrote the relation as $Q_{\mathrm{it}} \simeq C_{\mathrm{ox}} \times \Delta V_{\mathrm{TH}}$. As the Reviewer correctly pointed out, since C_{ox} is already defined as the gate dielectric capacitance per unit area (F/cm^2), the $C_{\mathrm{ox}} \times \Delta V_{\mathrm{TH}}$ directly represents the density of the trapped charge (C/cm^2). Therefore, dividing this result by the gate area again is dimensionally incorrect.

To correctly define Q_{it} as the total trapped charge, the surface charge density (σ) should be described by the relation:

$$\sigma = Q_{\mathrm{it}}/\mathrm{area} \simeq C_{\mathrm{ox}} \times \Delta V_{\mathrm{TH}}$$

Please be assured that this was a typographical error in the response letter and did not affect our calculation or data analysis.

We have revised the previous response letter to accurately reflect the physical dimensions as follows:

(1) Corrected the Reviewer (#2)'s comments - **Q1** (p. 25).

… The trapped charge (Q_{it}) can be described by the relation:

$$\sigma = Q_{\mathrm{it}}/\mathrm{area} \simeq C_{\mathrm{ox}} \times \Delta V_{\mathrm{TH}}$$

where C_{ox} is the gate dielectric capacitance per unit area and ΔV_{TH} is the illumination-induced V_{TH} shift. This trapped charge corresponds to a negative sheet charge density (σ) at the interface, which enhances the internal electric field in CsF as follows. …

Reviewer (#3)'s COMMENTS:

In the revised manuscript, the authors have addressed my previous concerns. However, some issues need further clarification before acceptance for publication.

Q1) The authors treat the n-type (C13-PTCDI) and p-type (C10-DNTT) semiconductors as dielectrics in their band diagram analysis. As a result, there is no band bending at interfaces (including both heterostructure and semiconductor-dielectric interfaces). Such analysis could be misleading considering depletion/accumulation regions that might have dimensions comparable to the thickness of the semiconductors. A more thorough analysis should be included. Additionally, how the authors determine the bend diagram under light illumination is also lacking.

In the revised manuscript, some typos should also be corrected:

(1) Fig. 1g and Fig. 3c, "dark (dashed lines)" and "illumination (solid lines)" do not match the figures.

(2) Page 11: "the apparent work function of the Au/CsF contact (~3.8 eV) places its Fermi level above the lowest unoccupied molecular orbital (LUMO) level of C13-PTCDI (2.06 eV), whereas the much deeper highest occupied molecular orbital (HOMO) level of the C10-DNTT (4.92 eV) imposes a large barrier." The LUMO of C13-PTCDI is not correct.

We sincerely appreciate the Reviewer's insightful comments. We completely agree that the previous multi-dielectric layer approximation was insufficient to capture the band bending at the interfaces and the effects of light and photogenerated carriers. To address this concern and provide a rigorous analysis, we have replaced the previous schematic models with TCAD simulations in the revised manuscript.

In the TCAD simulations, C10-DNTT and C13-PTCDI were defined as semiconductors, whereas the injection barrier layers (CsF or MoO_X) were defined as insulators. The input parameters used for the simulations are summarized in **Table R2**, incorporating values that were either experimentally measured or adopted from the literature[R5], [R6], [R7], [R8]. To ensure computational convergence while preserving the physical validity of the charge transport, the simulated device features a scaled channel length of 5 μm, with the layer thickness kept identical to the actual device (**Fig. R6-9**). For more details, please refer to our response to Reviewer (#2)'s comment #1.

Table R2. Parameters used for the TCAD simulation.

Material	Parameter	Value
Al	Work function	4.3 eV
	Refractive index (n) / Extinction coefficient (k) @460 nm	0.541 / 5.119
Au	Work function	4.9 eV
	Refractive index (n) / Extinction coefficient (k) @460 nm	1.394 / 1.828
C_{10}-DNTT	LUMO	2.33 eV
	HOMO	4.92 eV
	Band gap	2.59 eV
	Dielectric constant	3
	Electron mobility	10^{-3} $cm^2V^{-1}s^{-1}$
	Hole mobility	6 $cm^2V^{-1}s^{-1}$
	Doping concentration	10^{15} cm^{-3}
	Conduction band density of states (N_C)	10^{20} cm^{-3}
	Valence band density of states (N_V)	10^{20} cm^{-3}
	Refractive index (n) / Extinction coefficient (k) @460 nm	1.198 / 0.016
C_{13}-PTCDI	LUMO	4.36 eV
	HOMO	6.42 eV
	Band gap	2.06 eV
	Dielectric constant	3
	Electron mobility	1.3 $cm^2V^{-1}s^{-1}$
	Hole mobility	10^{-3} $cm^2V^{-1}s^{-1}$
	Conduction band density of states (N_C)	10^{20} cm^{-3}
	Valence band density of states (N_V)	10^{20} cm^{-3}
	Refractive index (n) / Extinction coefficient (k) @460 nm	1.370 / 0.066
CsF	Conduction band energy	1.81 eV
	Valence band energy	7.1 eV
	Band gap	5.29 eV
	Dielectric constant	7.9 [R1]
	Refractive index (n) / Extinction coefficient (k) @460 nm	1.607 / ~ 0
MoOx	Conduction band energy	5.8 eV [R2]
	Valence band energy	9.0 eV [R2]
	Band gap	3.2 eV
	Dielectric constant	20 [R3]
	Refractive index (n) / Extinction coefficient (k) @460 nm	2.065 / ~ 0
pV3D3	LUMO	1.2 eV [R4]
	HOMO	9.45 eV [R4]
	Band gap	8.25 eV
	Dielectric constant	2.2 [R4]
	Refractive index (n) / Extinction coefficient (k) @460 nm	1.498 / ~ 0

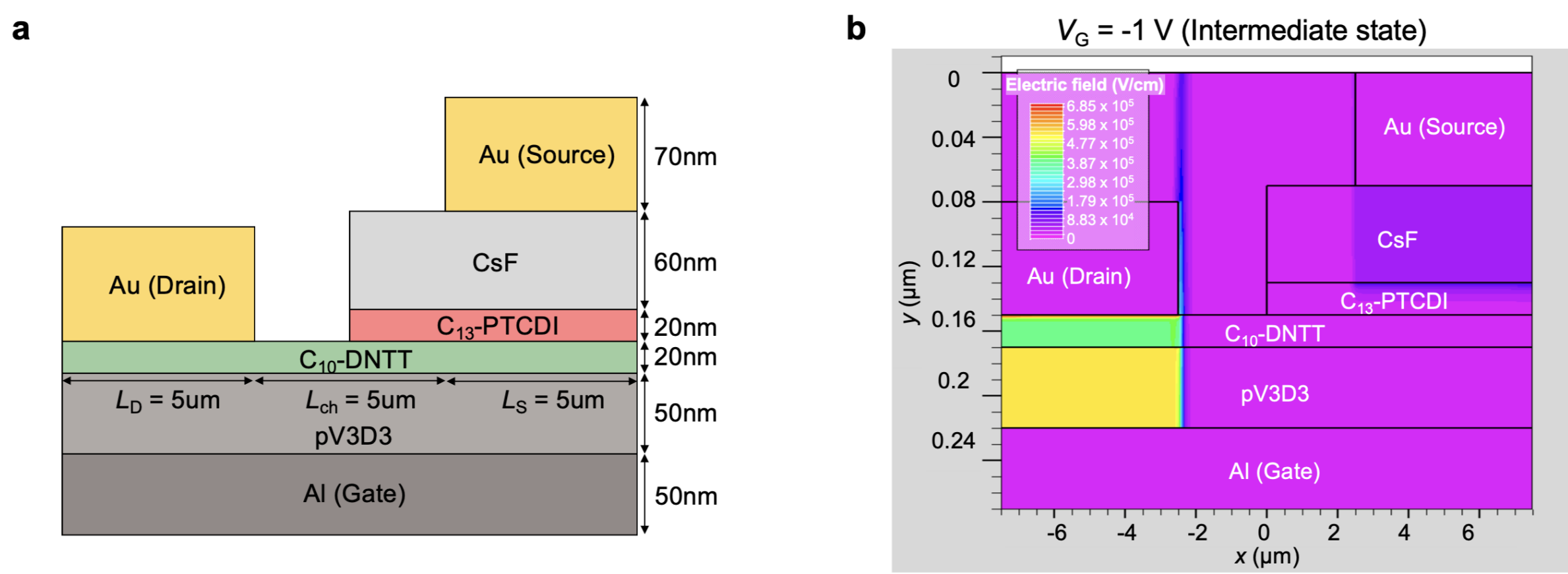

Figure R6. a. The schematic illustration of the scaled p-type T-HTR device structure used for the simulation (channel length = 5 μm). **b.** Electric field distribution in the intermediate state (V_G = -1 V, V_D = -4 V).

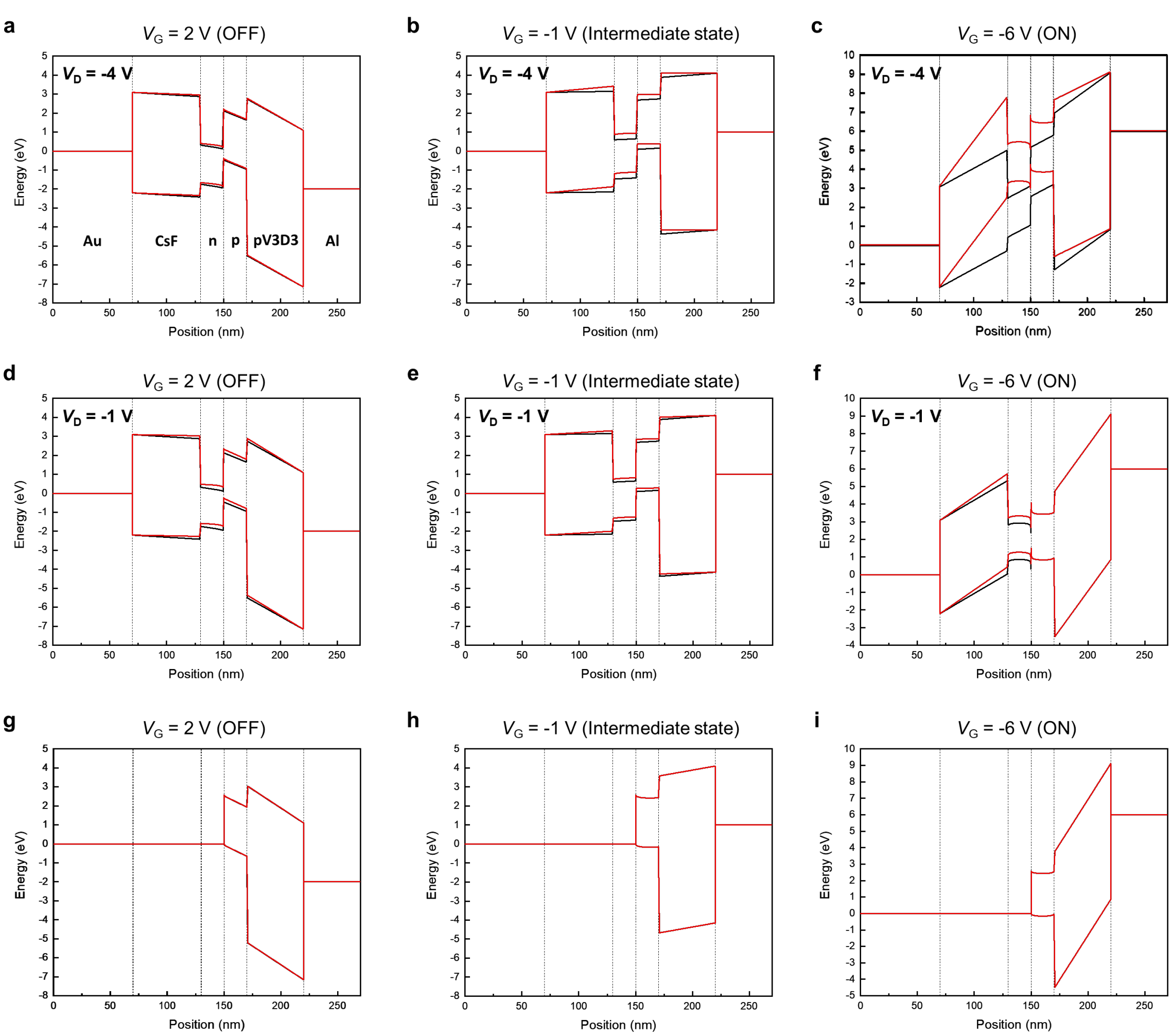

Figure R7. Simulated energy band diagrams of the scaled p-type T-HTRs at the source-side, in the dark (black lines) and under illumination (red lines) at three representative V_G states: **a**. V_G = 2 V (OFF), **b**. -1 V (intermediate), and **c**. -6 V (ON). For **a-c**, V_D = -4 V. Corresponding diagrams at V_D = -1 V are shown in **d-f**. Corresponding diagrams for the reversed source/drain configuration are shown in **g-i**. For **g-i**, V_D = -4 V. .

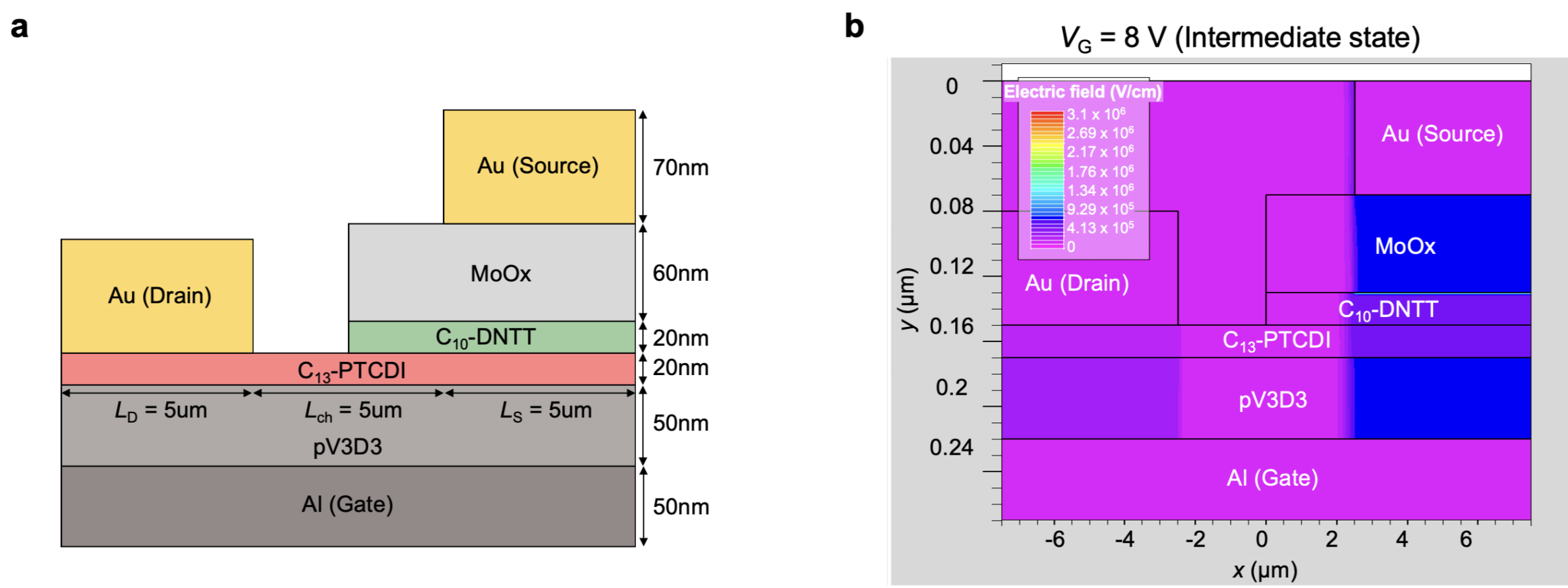

Figure R8. a. The schematic illustration of the scaled n-type T-HTR device structure (channel length = 5 μm). **b.** Electric field distribution in the intermediate state (V_G = 8V, V_D = 10 V).

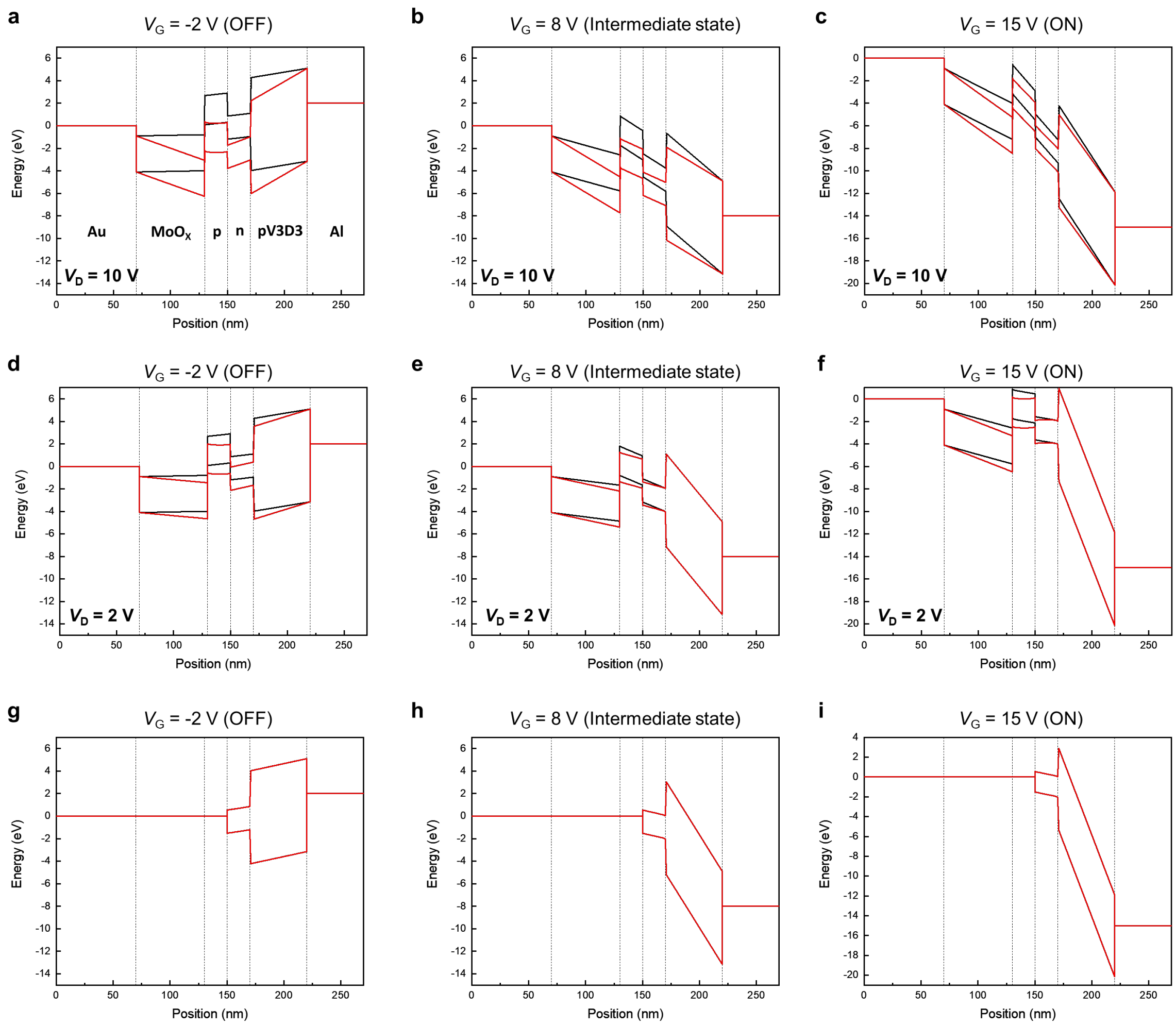

Figure R9. Simulated energy band diagrams of the scaled n-type T-HTRs at the source-side, in the dark (black lines) and under illumination (red lines) at three representative V_G states: **a.** V_G = -2 V (OFF), **b**. 8 V (intermediate), and **c**. 15 V (ON). For **a-c**, V_D = 10 V. Corresponding diagrams at V_D = 2 V are shown in **d-f**. Corresponding diagrams for the reversed source/drain configuration are shown in **g-i**. For **g-i**, V_D = 10 V.

Also, we thank the Reviewer for carefully pointing out the typographical inconsistencies and errors in the revised manuscript. These issues have been corrected by revising the figure captions so that the solid and dashed lines consistently correspond to the dark and illuminated conditions, respectively. We further corrected the LUMO level of C13-PTCDI from 2.06 eV to 4.36 eV based on the experimentally extracted energy levels. In addition, the HOMO level used to evaluate the hole injection barrier has been updated from that of C10-DNTT to that of C13-PTCDI, as the Au/CsF contact is directly formed on the C13-PTCDI layer. We sincerely apologize

for any confusion that may have been caused in the previous submission.

We appreciate the Reviewer's attention to band diagrams, and the manuscript has been revised accordingly.

[R5] *Physical Review*, **168**(3), 1054 (1968).
[R6] *Applied Physics Letters*, **96**(6), 063303 (2010).
[R7] *Advanced Electronic Materials*, **6**(10), 2000635 (2020).
[R8] *Nature materials*, **14**(6), 628-635 (2015).

(1) Revised Fig. 1g in the revised manuscript (p. 31).

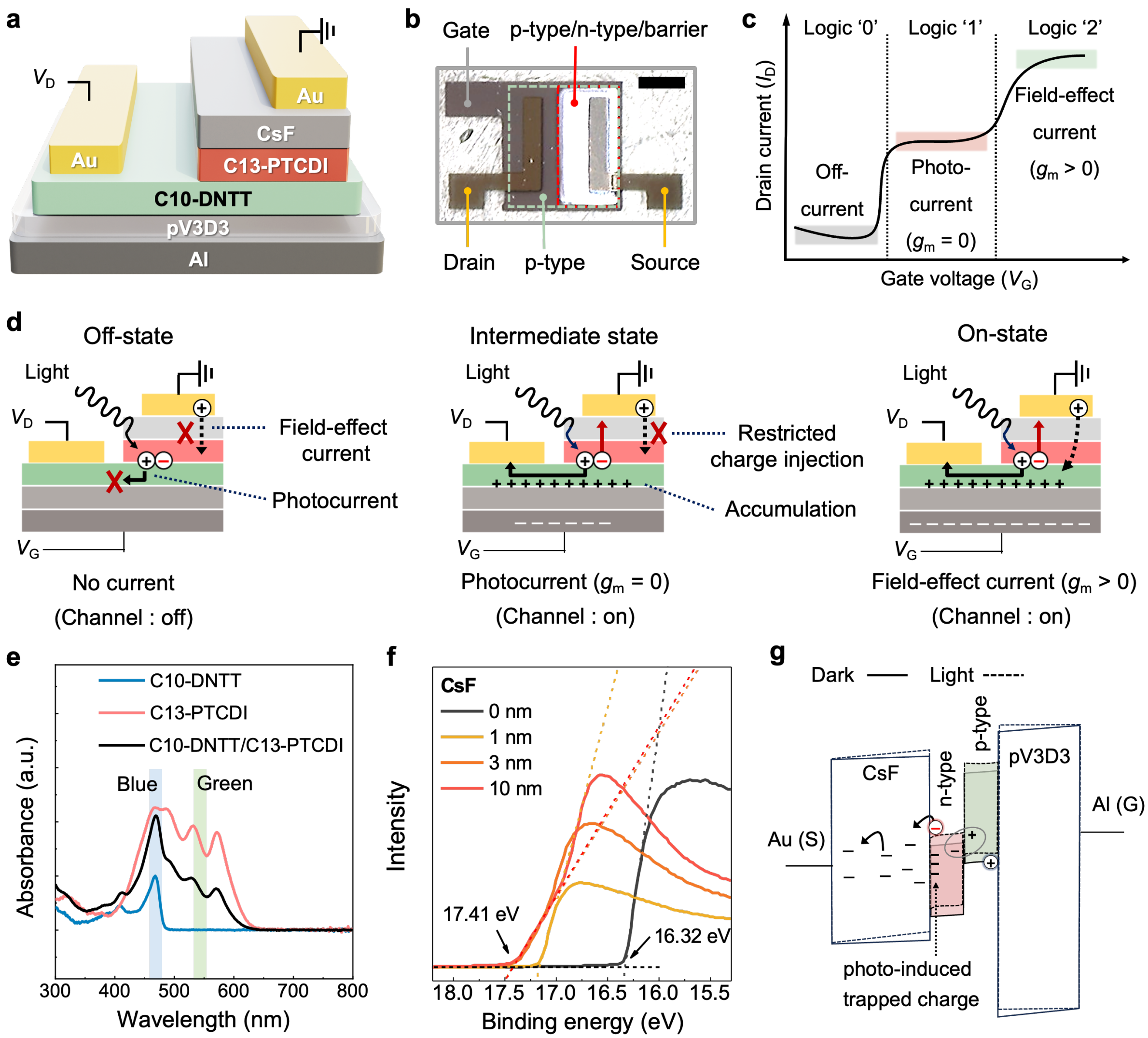

Figure 1 | Design of the ternary heterojunction transistor (T-HTR). a. The

schematic illustration and **b**. optical microscopy image of the T-HTR (scale bar: 500 μm). **c**. The schematic illustration of transfer characteristics and **d**. operational mechanism of the T-HTR consisting of three switching states. **e**. Ultraviolet-visible (UV/Vis) absorption spectra of the n-type (C13-PTCDI), p-type (C10-DNTT) and n-type/p-type bilayer organic semiconductors. **f**. Ultraviolet photoelectron spectroscopy (UPS) spectra of Au contact with thermally deposited cesium fluoride (CsF) as a function of CsF thickness. **g**. Energy band diagrams of the source-side stack in the dark (solid lines) and under illumination (dashed lines). The photo-induced trapped charge at the CsF/p-type interface enhances the internal electric field in CsF.

(2) Revised Fig. 3c in the revised manuscript (p. 33).

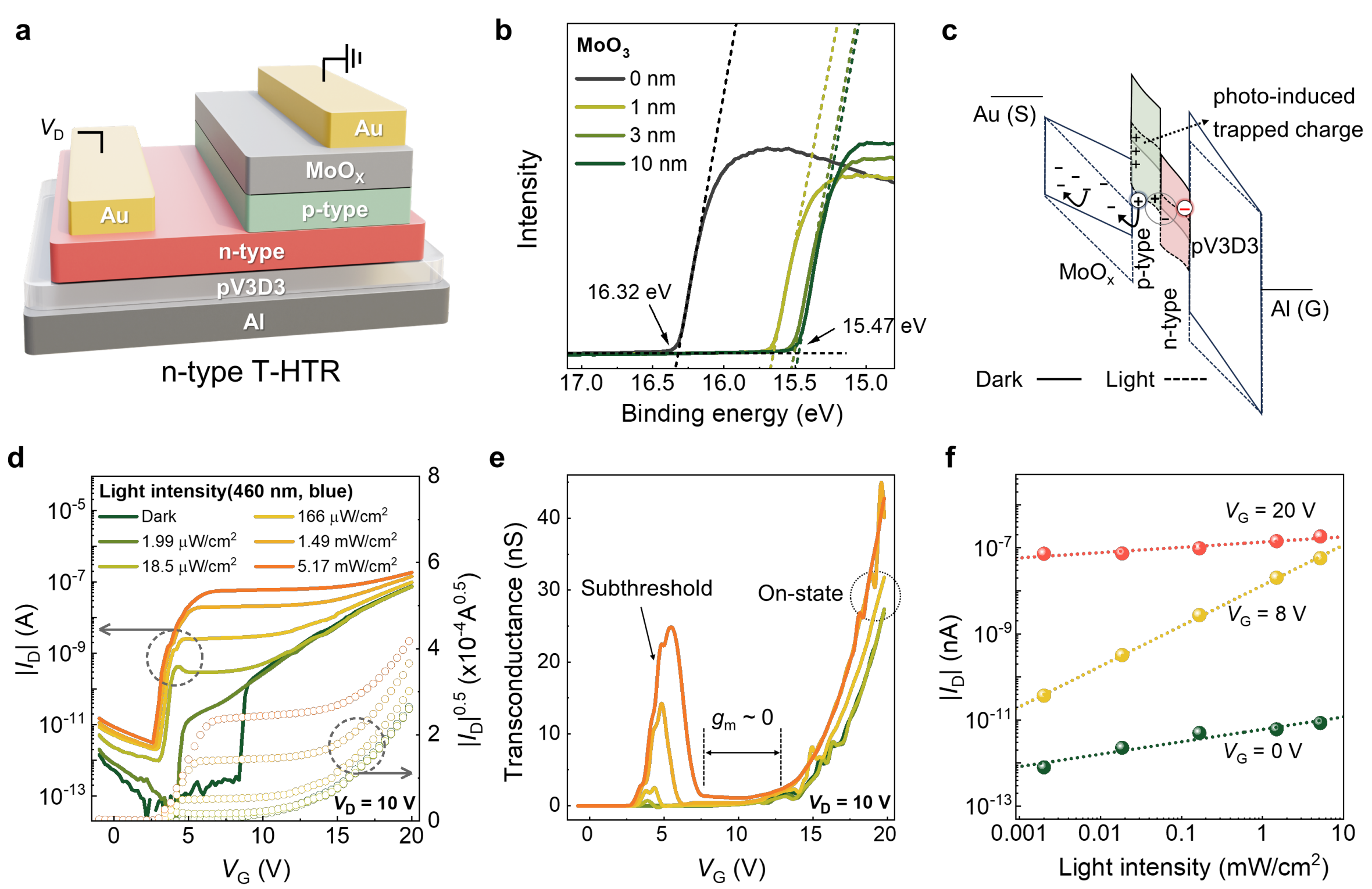

Figure 3 | Versatility of the T-HTR architecture. a. A schematic illustration of the T-HTR with molybdenum oxide (MoO_X) injection barrier. **b**. UPS spectra of Au contact with thermally deposited MoO_X as a function of MoO_X thickness. **c**. Energy band diagrams of the source-side stack in the dark (solid lines) and under illumination (dashed lines). The photo-induced trapped charge at the MoO_X/n-type interface

enhances the internal electric field in MoO_X. **d**. The transfer characteristics, **e**. g_m, and **f**. I_D at the off-, intermediate, and on-state of the T-HTR under 460 nm illumination with respect to intensity.

(3) Replaced Supplementary Figure. 8 with **Fig. R6** in the revised Supplementary Information (p. 9).

(4) Replaced Supplementary Figure. 17 with **Fig. R8** in the revised Supplementary Information (p. 22).

(5) Added the **Table R2** to Supplementary Information (p. 33)

(6) Added the reference [5-8] in the Supplementary Information.

(7) Revised the main text in the revised manuscript (p. 11).

In this configuration, both hole injection from the source and light-induced minority-electron transport from the n-type layer are in principle possible via the trap-assisted Poole-Frenkel process; however, the band alignment strongly favors electron transport while suppressing hole injection. Specifically, the apparent work function of the Au/CsF contact (~3.8 eV) is shallower than the lowest unoccupied molecular orbital (LUMO) level of C13-PTCDI (4.36 eV), whereas the much deeper highest occupied molecular orbital (HOMO) level of the C13-PTCDI (6.42 eV) imposes a large barrier.

Q2) The authors claim that "Although the present proof-of-concept devices employ large shadow-mask-defined geometries, scaling the channel length and minimizing gate–electrode overlap through photolithography would reduce C_gg by ~10^4-fold, potentially extending the cut-off frequency into the hundreds-of-kHz range. Also, combined with high-mobility semiconductors such as oxides or 2D materials, the attainable speed could further approach the MHz regime." However, the authors neither demonstrated the scaling of their devices or ternary transistors based on high-mobility oxides or 2D materials, making this claim vague. Without real demonstration, the concern of integration of their ternary devices with merits over

binary computation is not fully addressed.

Response:

We thank the Reviewer for raising this important point. We fully agree with the reviewer that the statement in the original manuscript might be interpreted as implying a deterministic projection of device performance, which was far from our intention. Our purpose in that discussion was to outline potential directions for device optimization rather than to claim experimentally demonstrated scalability.

In the present work, the devices were fabricated using a shadow-mask process to enable the formation of organic heterostructures for proof-of-concept demonstrations. This approach was adopted strategically because organic semiconductor systems naturally provide well-defined p-type and n-type transport characteristics and allow straightforward heterostructure formation through sequential deposition, which is widely employed in exploratory device studies for facile prototyping and systematic investigation of new device concepts without requiring complex lithographic processing[R5], [R6], [R7], [R8]. As a result, the devices inevitably possess relatively large geometries, including substantial gate–electrode overlap areas. Under such conditions, the estimated cut-off frequency is strongly influenced by the geometrical capacitance associated with these overlap regions. Our original discussion therefore highlighted overlap-area reduction as a plausible and promising strategy to mitigate this capacitance when lithographic patterning becomes available.

However, we fully acknowledge that presenting a quantitative projection of a ~10^4-fold improvement without experimental validation may appear overly speculative. In response to the Reviewer's comment, we have revised the manuscript to remove the explicit numerical projection and replaced it with a more qualitative discussion describing overlap-area minimization as one possible route for improving the operating frequency.

Finally, we would like to clarify the scope of the present work. The primary objective of this study is to introduce and experimentally validate a new device operation principle for light-driven ternary logic. The proof-of-concept T-HTR devices demonstrate optoelectronic ternary logic gates and pixel-level sensory circuits capable of performing multifunctional image-processing operations, such as pixel integration and intersection, through dynamic switching of logic functions. These results illustrate how sensing and logic operations can be integrated

within a single device platform, providing a potential pathway toward compact in-sensor computing architectures. Unlike traditional binary implementations, where such multifunctional operations typically require multiple transistors and additional circuit elements, the proposed T-HTR architecture enables these functions to be realized more compactly within a single device through its intrinsic multi-state operation under optical control. In this context, the discussion of potential scaling was intended only to outline plausible future directions for device optimization rather than to assert realized performance advantages. This architectural feature highlights how multi-state optoelectronic devices may offer an alternative pathway for compact sensory computing systems, where sensing, logic, and signal processing can be co-located within a single device structure.

We appreciate the Reviewer's thoughtful comment, which helped us clarify the scope and presentation of this discussion in the revised manuscript.

We revised the manuscript as follows:

(1) Revised the Supplementary Information (p. 20).

As the CsF thickness increased from 15 nm to 60 nm, the estimated f_T decreased from 10^2-10^3 Hz to below 1 Hz, whereas the on-state (dynamic) power density dropped from the mW cm^{-2} scale to the sub-μW cm^{-2} scale. Such a speed–power compromise is highly relevant for application domains where ultralow static power is prioritized over high operating frequency, including always-on optical sensors, wearable biomedical monitors, and distributed environmental sensor nodes typically operating in the Hz–kHz regime[1~8]. Furthermore, the ternary-logic framework intrinsically reduces the number of computational steps by ≈63 % (log2 / log3), offering additional energy savings[9].

In the present proof-of-concept devices, the channel structures were defined using a shadow-mask process, which inevitably results in relatively large device geometries and substantial gate-electrode overlap areas. Under such conditions, the estimated cut-off frequency is strongly influenced by geometrical capacitance associated with these overlap regions. Therefore, minimizing gate-electrode overlap through

lithographic patterning could provide a practical pathway for reducing parasitic capacitance and improving the operating frequency in future device implementations.

These analyses demonstrate that injection-barrier engineering provides a practical and tunable design handle to balance operation speed and power efficiency in light-driven multi-state logic systems.

(2) Revised the main text in the revised manuscript (p. 4).

Here, this intermediate state is not merely an additional logic level but a light-driven, dynamically adjustable state that can transition toward either the on- or off-states or remain stable between them. Thus, this intermediate state serves as a bridge, preserving optical information that would otherwise be lost in binary logic systems. Therefore, optoelectronic ternary logic circuits ensure that the diverse properties of light can be reflected directly in computational operations, rather than being constrained to rigid binary classifications, introducing a new paradigm for information processing. In particular, a transistor capable of intrinsically supporting multiple logic states can enable circuit functions to be implemented more compactly, as operations that would otherwise require several binary switching elements may be realized within a single multi-state device.

(3) Revised the main text in the revised manuscript (p. 7).

We believe that the T-HTR developed in this study offers a device-level foundation for future optoelectronics, bridging optical input encoding and multi-state logic within a single transistor, thereby providing a pathway toward compact in-sensor computing systems based on multi-state optoelectronic devices.

[R5] *Advanced Materials*, **34**(15), 2109491 (2022).
[R6] *Advanced Materials*, e11018 (2025).
[R7] *Nature Electronics*, **4**(7), 522-529 (2021).
[R8] *Science advances*, **9**(27), eadg5946 (2023).

Response to the Reviewers' Comments

The authors thank the Reviewers for their thorough review of our manuscript and the valuable comments. We have revised the manuscript and supporting information based on the Reviewers' comments. The Reviewers' comments appear in **black**, and the authors' responses are in **blue**. In the revised manuscript, the changes with respect to the previous version are highlighted.

Reviewer (#1)'s COMMENTS:

The authors have addressed most of my previous concerns, and the manuscript has been substantially improved, particularly through the addition of TCAD simulations and the clarification of the operating mechanism. The overall presentation is now significantly clearer and more self-consistent.

I only have two minor comments that, in my opinion, would further strengthen the manuscript.

Q1) 1. The authors attribute the transport in the CsF layer mainly to Poole–Frenkel conduction based on the linear relationship between ln(J/E) and sqrt(E). While this interpretation is reasonable, similar behavior may also arise from Schottky emission or hopping-assisted transport over a limited electric-field range.

To further support the Poole–Frenkel assignment, the authors may consider providing additional evidence, such as temperature-dependent J–E measurements, comparison with Schottky-emission fitting, extraction of the apparent dielectric constant from the fitted slope, or thickness-/electrode-dependence analysis. In the absence of such evidence, I suggest that the authors slightly soften the wording regarding the dominant transport mechanism.

Response:

We thank the Reviewer for this thoughtful and constructive comment. We fully agree that just the linear relationship between ln(J_i/E_i) and $\sqrt{E_i}$ (where J_i and E_i denote the current density and electric field, respectively) should not be regarded as definitive evidence for an ideal Poole-Frenkel mechanism, as Schottky emission, hopping-assisted transport, or other trap-assisted processes may exhibit similar field-dependent behavior over a limited electric-field

range. In particular, considering the thick and wide-bandgap insulating nature of the CsF layer, microscopic charge transport through localized defect states, including hopping-assisted carrier motion, is physically plausible and may naturally contribute to the observed transport behavior.

Following the Reviewer's suggestion, we have therefore revised our interpretation to avoid an overly exclusive assignment of the transport mechanism. In the revised manuscript, we describe the conduction through the CsF layer as "field-assisted trap-mediated transport" rather than simply as "Poole–Frenkel conduction".

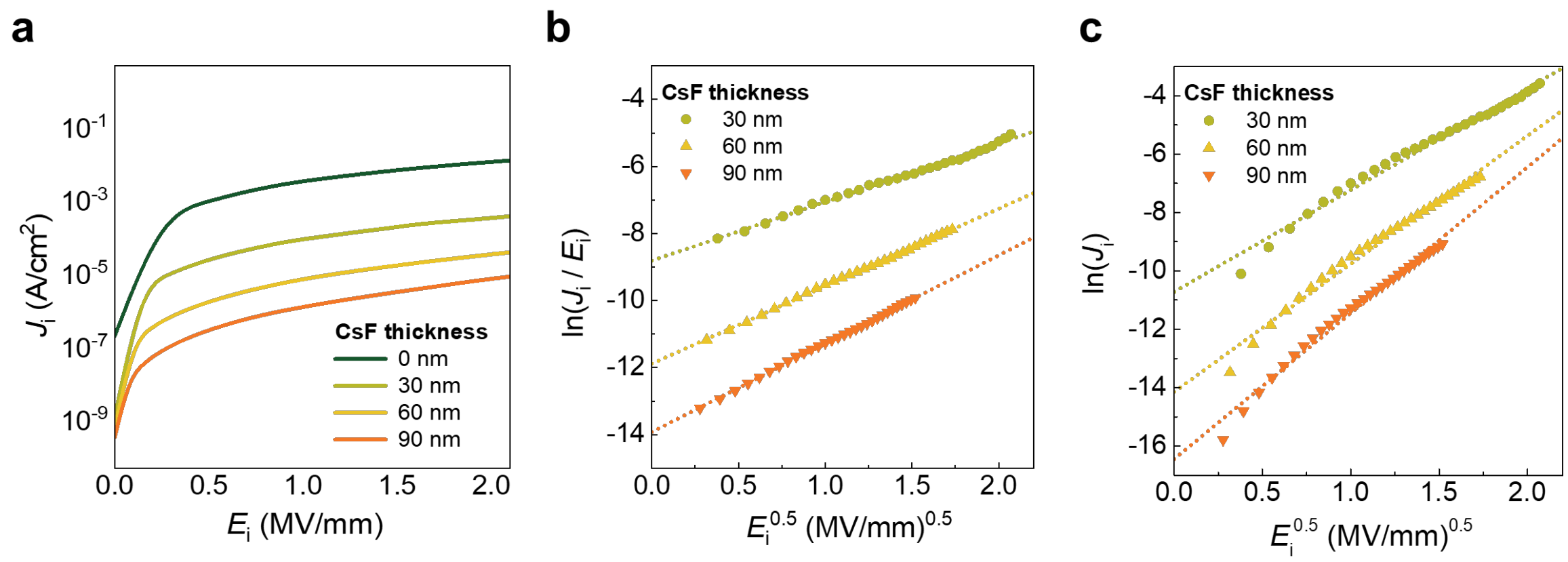

Figure R1. a. Current density–electric field (J_i–E_i) characteristics of the Au/C10-DNTT (20 nm)/C13-PTCDI (20 nm)/CsF (0 to 90 nm)/Au devices with different CsF thicknesses, showing a systematic reduction of current with increasing barrier thickness. **b**. Poole-Frenkel plots and **c**. Schottky plots derived from the J_i–E_i data.

To support this more cautious description, we additionally compared the Poole-Frenkel representation, ln(J_i/E_i) vs $\sqrt{E_i}$, with the Schottky-emission representation, ln(J_i) vs $\sqrt{E_i}$, using the same J_i/E_i data (**Fig. R1**). The Poole-Frenkel representation showed somewhat better linearity than the Schottky representation across the investigated CsF thickness range. We now use this comparison not to exclude all other microscopic pathways, but to support the conclusion that the transport is more consistent with a bulk trap-mediated process than with purely interface-limited Schottky emission.

We sincerely appreciate the reviewer's careful examination and constructive comments. We have thoroughly reviewed the manuscript and supplementary information on these points and have revised the text accordingly to enhance clarity and accuracy.

We revised the manuscript as follows:

(1) Revised the main text in the revised manuscript (p. 10).

The current density (J_i)–electric field (E_i) characteristics of this stacked device showed a systematic decrease in J_i with increasing CsF thickness, confirming that a thicker barrier effectively suppresses carrier transmission (**Supplementary Fig. 5**). Notably, when the experimental data were analyzed in Poole-Frenkel-type [$\ln(J_i/E_i)$ versus $\sqrt{E_i}$] and Schottky-type [$\ln(J_i)$ versus $\sqrt{E_i}$] representations, the Poole-Frenkel representation showed better linearity across the investigated CsF thickness range. These results suggest that charge transport through the thick, wide-bandgap CsF layer is better described as field-assisted trap-mediated transport, rather than injection-limited emission[47,48]. In this mechanism, a thicker CsF layer reduces the average electric field and extends the trap-mediated path, thereby lowering the overall transmission probability.

(2) Revised the main text in the revised manuscript (p. 11).

In this configuration, both hole injection from the source and light-induced minority-electron transport from the n-type layer are in principle possible via the trap-assisted process; however, the band alignment strongly favors electron transport while suppressing hole injection. … This enhanced electric field in CsF lowers the effective barrier for trap-assisted conduction, leading to large threshold voltage (V_{TH}) shift in devices incorporating a CsF injection barrier under illumination, as will be discussed later.

(3) Revised the main text in the revised manuscript (p. 20).

In addition, similar to the CsF, charge transport through the MoO_X layer proceeds via trap-assisted conduction due to the mixed valence states and oxygen-deficiency defects[65,66].

(4) Revised the Supplementary Figure 5 in the Supplementary Information (p. 6).

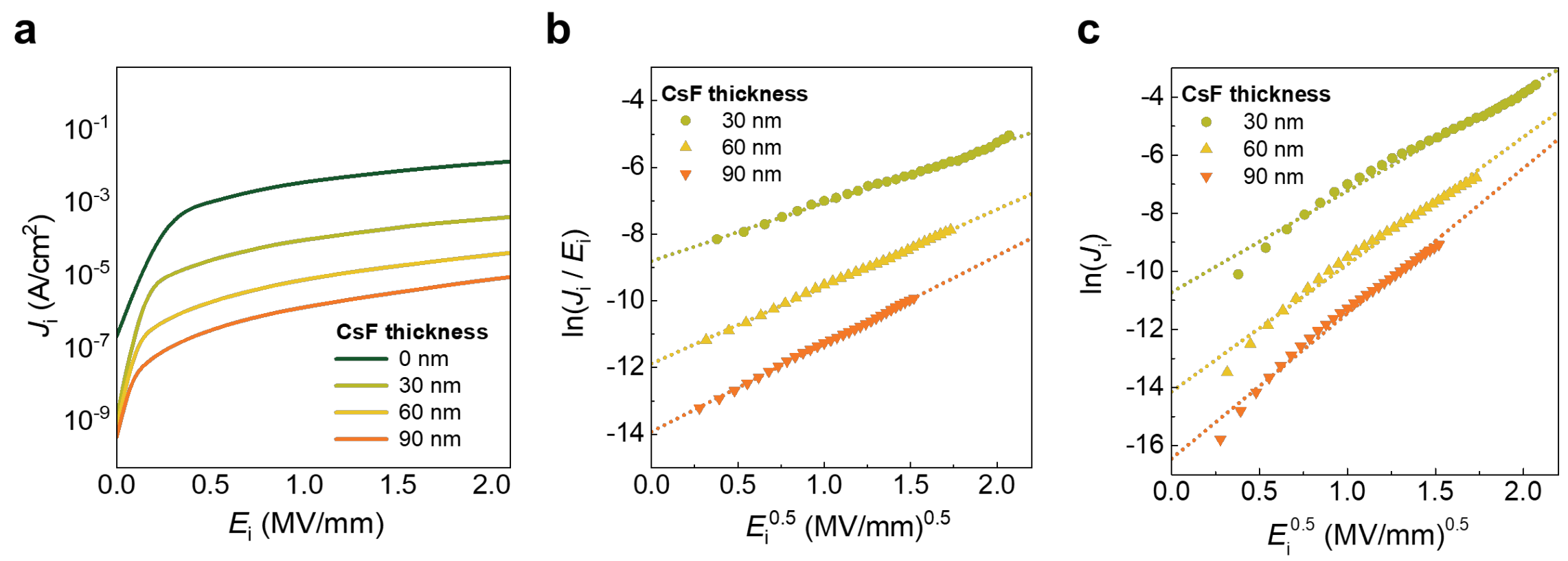

Supplementary Figure 5 | a. Current density–electric field (J_i–E_i) characteristics of the Au/C10-DNTT (20 nm)/C13-PTCDI (20 nm)/CsF (0 to 90 nm)/Au devices with different CsF thicknesses, showing a systematic reduction of current with increasing barrier thickness. **b**. Poole-Frenkel plots and **c**. Schottky plots derived from the J_i–E_i data.

Q2) The authors have appropriately revised the previous discussion regarding frequency scaling and now present the work more carefully as a proof-of-concept demonstration. Nevertheless, the experimentally demonstrated cut-off frequency remains relatively low compared to conventional logic devices. While the proposed architecture is interesting for ultralow-power sensing and in-sensor computing applications, I recommend that the authors further emphasize the current proof-of-concept nature of the platform and briefly discuss the remaining challenges toward practical implementation, such as operating speed, scalability, and large-scale integration compatibility.

Response:

We thank the Reviewer for this thoughtful and constructive comment. We agree that the cut-off frequency of a light-driven ternary heterojunction transistor (T-HTR) demonstrated experimentally in this study still remains relatively low compared with conventional logic devices. We also agree that the manuscript should further emphasize the proof-of-concept nature of the current platform and more clearly discuss the remaining challenges toward practical implementation.

In response to the Reviewer's suggestion, we revised both the Results and Conclusion sections. First, in the Results section, we now explicitly clarify that the relatively low cut-off frequency of the present devices is mainly associated with the use shadow-mask-defined geometry for this proof-of-concept demonstration. In addition, we revised the Conclusion section to more explicitly discuss the remaining challenges toward practical implementation, including operating speed, scalability, and large-scale integration compatibility. Specifically, we now state that future implementation will require lithographic down-scaling for the reduce parasitic capacitance, together with reproducible fabrication of uniform heterojunction/barrier stacks and array-level integration with localized or integrated light sources. We further added that optimization of the injection-barrier thickness and interfacial energy-level alignment to minimize the barrier-associated contact resistance, will be essential for minimizing the speed-power trade-off while preserving a sufficient intermediate-state margin for robust ternary logic operation.

Through these revisions, we have further clarified that the present work should be viewed as a proof-of-concept demonstration of light-driven multi-state logic, while also outlining

the key device-, material-, and system-level challenges that need to be addressed for practical implementation.

We revised the manuscript as follows:

(1) Revised the main text in the revised manuscript (p. 16).

As the CsF thickness increased from 15 nm to 60 nm, the estimated cut-off frequency decreased from 10^2-10^3 Hz to below 1 Hz, whereas the on-state (dynamic) power density dropped from the mW cm^{-2} scale to the sub-μW cm^{-2} scale (**Supplementary Fig. 15**). The relatively low cut-off frequency estimated in the present devices is mainly attributed to the shadow-mask-defined geometry commonly used for proof-of-concept device demonstrations, which results in large device dimensions and substantial gate-overlap induced parasitic capacitance.

(2) Revised the main text in the revised manuscript (p. 27).

Overall, the T-HTR exemplifies a novel optoelectronic logic paradigm that fully exploits the tunable nature of light and bridges ternary logic with sensory data processing. Although the present platform remains at a proof-of-concept stage, particularly with respect to operating speed, its speed-power trade-off is aligned well with ultralow-power sensing and in-sensor computing applications, where reduced static power and operation steps can be more critical than high clock frequency. Further progress toward practical implementation will require lithographic device down-scaling to reduce parasitic capacitance, reproducible fabrication of uniform heterojunction/barrier stacks, and array-level integration with localized or integrated light sources. In parallel, optimizing the injection-barrier thickness and interfacial energy-level alignment, while reducing barrier-associated contact resistance, will be essential to minimize the speed-power trade-off and preserving a sufficient intermediate-state margin for robust ternary logic operation. With these advances, this material-adaptable architecture will provide a new pathway toward compact, energy-efficient optoelectronic systems for reconfigurable in-sensor computing.

Reviewer (#2)'s COMMENTS:

All my concerns have been properly addressed. Therefore, the manuscript is suitable for publication.

Response:

We sincerely thank the Reviewer for the positive evaluation of our manuscript and for the constructive comments provided throughout the review process. We greatly appreciate the Reviewer's time and valuable feedback, which helped us improve the quality and clarity of the manuscript.

Reviewer (#3)'s COMMENTS:

The authors fully addressed my concern. No more revision needed.

Response:

We thank the Reviewer for the careful evaluation of our revised manuscript and for recognizing that the concerns have been satisfactorily addressed. We sincerely appreciate the Reviewer's constructive comments and valuable suggestions throughout the review process.

The manuscript proposed a novel light-driven ternary heterojunction transistor (T-HTR) design, demonstrating three distinct states and serving as key enablers for ternary logic gates. The presence of the PN junction at the source induces a built-in electric field which facilitates the separation of phonon-generated carriers. In addition, its potential in implementing in-sensor computing applications has also been explored. While some results appear interesting, this work still suffer from several critical shortcomings: inadequate physical mechanism analysis and device characterization etc. Before this manuscript can be considered for publication in Nature Communications, substantial revisions are required to meet the journal's rigorous standards.

(1) Several essential device geometry and material property related parameters are missing in this manuscript: the relative dielectric constant of gate oxide layer, the bandgap and electron affinity of the p-type/n-type semiconductor, the mobility of the conducting channel, the length of the gate/source/drain electrodes etc. Moreover, more rigorous band alignments for the heterojunction and the source side that determines carrier injections should be given to present a more convincing physical picture about the operating mechanism. TCAD simulations will be favored.

(2) The insertion of CsF layer serves as blocking barrier for source carrier injection. In this case, do carriers from the source electrode tunnel through the CsF layer? If so, the thickness of the CsF layer will play a pivotal role in determining the tunneling probability. Please clarify on that.

(3) In view of dominating mechanism for the transistor design, the thickness and doping level of both p-type and n-type semiconductors will also have a significant impact on the performance, which determines the magnitude of built-in electric field and the depletion width of the heterojunction. Please clarify on that.

(4) The time-resolved response of the transistor relies on two processes: the separation of phonon-generated carriers and the transit of carriers within the channel. Therefore, the channel mobility and length will have significant impact on the transient response. Whether the scaling of the channel length will improve the time-resolved performance? Please provide additional experimental or simulation results.

(5) In Line 204 of this manuscript, the authors mentioned that the increment of light

intensity leads to the negative shift of threshold voltage. Generally, the threshold voltage is mainly determined by the height of source barrier for a transistor. A more clear description should be given to shed light upon this point.

(6) In Supplementary Table I, the units for the static power consumption of the last two entries should be corrected from 'A' to 'W' for consistency. Additionally, since the table includes devices with varying geometries, a fair comparison of static power consumption should be normalized by device area. Furthermore, power consumption and performance are inherently trade-offs in transistor design. An excessively low current density will lead to poor device performance, such as slow switching speeds. Thus, more detailed discussions and comprehensive figures illustrating this trade-off would strengthen the analysis.
